# Progress toward Global Reduction in Under-Five Mortality: A Bootstrap Analysis of Uncertainty in Millennium Development Goal 4 Estimates

**DOI:** 10.1371/journal.pmed.1001355

**Published:** 2012-12-11

**Authors:** Leontine Alkema, Jin Rou New

**Affiliations:** 1 Department of Statistics and Applied Probability, National University of Singapore, Singapore; 2 Saw Swee Hock School of Public Health, National University of Singapore, Singapore; Umeå Centre for Global Health Research, Umeå University, Sweden

## Abstract

**Background:**

Millennium Development Goal 4 calls for an annual rate of reduction (ARR) of the under-five mortality rate (U5MR) of 4.4% between 1990 and 2015. Progress is measured through the point estimates of the United Nations Inter-agency Group for Child Mortality Estimation (UN IGME). To facilitate evidence-based conclusions about progress toward the goal, we assessed the uncertainty in the estimates arising from sampling errors and biases in data series and the inferior quality of specific data series.

**Methods and Findings:**

We implemented a bootstrap procedure to construct 90% uncertainty intervals (UIs) for the U5MR and ARR to complement the UN IGME estimates. We constructed the bounds for all countries without a generalized HIV epidemic, where a standard estimation approach is carried out (174 countries). In the bootstrap procedure, potential biases in levels and trends of data series of different source types were accounted for. There is considerable uncertainty about the U5MR, particularly for high mortality countries and in recent years. Among 86 countries with a U5MR of at least 40 deaths per 1,000 live births in 1990, the median width of the UI, relative to the U5MR level, was 19% for 1990 and 48% for 2011, with the increase in uncertainty due to more limited data availability. The median absolute width of the 90% UI for the ARR from 1990 to 2011 was 2.2%. Although the ARR point estimate for all high mortality countries was greater than zero, for eight of them uncertainty included the possibility of no improvement between 1990 and 2011. For 13 countries, it is deemed likely that the ARR from 1990 to 2011 exceeded 4.4%.

**Conclusions:**

In light of the upcoming evaluation of Millennium Development Goal 4 in 2015, uncertainty assessments need to be taken into account to avoid unwarranted conclusions about countries' progress based on limited data.

*Please see later in the article for the Editors' Summary*

## Abstract 2

Leontine Alkema and colleagues use a bootstrap procedure to assess the uncertainty around the estimates of the under-five mortality rate produced by the United Nations Inter-Agency Group for Child Mortality Estimation.

## Editors' Summary

**Background:**

In September 2000, world leaders adopted the United Nations Millennium Declaration, committing member states (countries) to a new global partnership to reduce extreme poverty and improve global health by setting out a series of time-bound targets with a deadline of 2015—the Millennium Development Goals (MDGs). There are eight MDGs and the fourth, MDG 4, focuses on reducing the number of deaths in children aged under five years by two-thirds from the 1990 level. Monitoring progress towards meeting all of the MDG targets is of vital importance to measure the effectiveness of interventions and to prioritize slow progress areas. MDG 4 has three specific indicators, and every year, the United Nations Inter-agency Group for Child Mortality Estimation (the UN IGME, which includes the key agencies the United Nations Children's Fund, the World Health Organization, the World Bank, and the United Nations Population Division) produces and publishes estimates of child death rates for all countries.

**Why Was This Study Done?:**

Many poorer countries do not have the infrastructure and the functioning vital registration systems in place to record the number of child deaths. Therefore, it is difficult to accurately assess levels and trends in the rate of child deaths because there is limited information (data) or because the data that exists may be inaccurate or of poor quality. In order to deal with this situation, analyzing trends in under-five child death rates (to show progress towards MDG 4) currently focuses on the “best” estimates from countries, a process that relies on “point” estimates. But this practice can lead to inaccurate results and comparisons. It is therefore important to identify a framework for calculating the uncertainty surrounding these estimates. In this study, the researchers use a statistical method to calculate plausible uncertainty intervals for the estimates of death rates in children aged under five years and the yearly reduction in those rates.

**What Did the Researchers Do and Find?:**

The researchers used the publicly available information from the UN IGME 2012 database, which collates data from a variety of sources, and a statistical method called bootstrapping to construct uncertainty levels for 174 countries out of 195 countries for which the UN IGME published estimates in 2012. This new method improves current practice for estimating the extent of data errors, as it takes into account the structure and (potentially poor) quality of the data. The researchers used 90% as the uncertainty level and categorized countries according to the likelihood of meeting the MDG 4 target.

Using these methods, the researchers found that in countries with high child mortality rates (40 or more deaths per 1,000 children in 1990), there was a lot of uncertainty (wide uncertainty intervals) about the levels and trends of death rates in children aged under five years, especially more recently, because of the limited availability of data. Overall, in 2011 the median width of the uncertainty interval for the child death rate was 48% among the 86 countries with high death rates, compared to 19% in 1990. Using their new method, the researchers found that for eight countries, it is not clear whether any progress had been made in reducing child mortality, but for 13 countries, it is deemed likely that progress exceeded the MDG 4 target.

**What Do These Findings Mean?:**

These findings suggest that new uncertainty assessments constructed by a statistical method called bootstrapping can provide more insights into countries' progress in reducing child mortality and meeting the MDG 4 target. As demonstrated in this study, when data are limited, uncertainty intervals should to be taken into account when estimating progress towards MDG 4 in order to give more accurate assessments on a country' progress, thus allowing for more realistic comparisons and conclusions.

**Additional Information:**

Please access these websites via the online version of this summary at http://dx.doi.org/10.1371/journal.pmed.1001355.

- The UN website has more information about the Millennium Development Goals, including country-specific data
- More information is available from UNICEF's ChildInfo website about the UN IGME and child mortality
- All UN IGME child mortality estimates and data are available via CME Info
- Countdown to 2015 tracks coverage levels for health interventions proven to reduce child mortality and proposes new actions to reach MDG 4

## Introduction

The assessment of levels and trends in child mortality is of key importance for measuring progress toward Millennium Development Goal (MDG) 4, which calls for a reduction of two-thirds in mortality in children under five (the under-five mortality rate [U5MR]), and for measuring the impact of interventions. For countries without well-functioning vital registration (VR) systems (the great majority of developing countries), assessing levels and trends in U5MR is challenging because of limited data availability and/or issues with data quality. The United Nations Inter-agency Group for Child Mortality Estimation (UN IGME, including the United Nations Children's Fund, the World Health Organization, the World Bank, and the United Nations Population Division) produces and publishes estimates of child mortality annually for all UN member states. The most recent set of estimates was published in 2012 [1].

When analyzing trends in U5MR, the focus is generally on the “best” estimates, i.e., the point estimates. This can lead to inaccurate conclusions about countries' progress in reducing the U5MR. For example, the annual rate of reduction (ARR) in Benin between 1990 and 2005 was estimated to be 2.4% in the most recent UN IGME estimates, based on all data available in 2012. Based on the data assumed to have been available in 2006 (i.e., data series from fieldwork conducted prior to 2006), the estimate of the ARR for the same period is only 1.8% (authors' calculation, using the current UN IGME U5MR estimation method); additional data increased the point estimate of the ARR by 33%. Such changes in point estimates of past ARRs are to be expected in many developing countries without well-functioning VR systems because data in such countries are mostly collected retrospectively and are subject to sampling and non-sampling errors. That is, a recent survey can provide data points for the 1990s and 2000s and can potentially further change the point estimate of the ARR for past periods. For an evidence-based analysis of progress in reducing U5MR, an uncertainty assessment of the estimates for U5MR levels and trends, in the form of uncertainty intervals (UIs), is thus required.

UIs are also required for more informed comparisons across countries. For example, even though the UN IGME point estimates for the ARR from 1990 to 2011 are very similar for Ghana and Equatorial Guinea (2.1% and 2.3%, respectively), the estimate for Equatorial Guinea is based on five data points in total and thus highly uncertain. Lower bounds of the UIs for the ARRs that quantify the minimum progress that most likely has been made would allow for a more informative ranking of countries in terms of proven progress in reducing U5MR.

In short, to avoid inaccurate conclusions and comparisons about countries' progress in reducing the U5MR, uncertainty in the estimation of U5MR and its rate of reduction needs to be assessed and taken into account when analyzing trends. The objective of this article is to propose a method to construct plausible UIs for the U5MR as well as for the ARR. In the previous UN IGME publication in 2011 [2], no uncertainty bounds were published because the bounds that were constructed by the default method were deemed implausible, i.e., too narrow. We discuss the default method and its drawbacks and introduce an alternative method to construct bootstrapped uncertainty bounds. We constructed the bounds for all countries without a generalized HIV epidemic and where a standard estimation approach was carried out (174 countries), compared the performance of the proposed method with the existing method, and highlight findings for high mortality countries. The proposed method was incorporated in the most recent UN IGME estimates in 2012 [1].

## Methods

### UN IGME Database and Estimation Approach

The UN IGME 2012 database is publicly available on CME Info (http://www.childmortality.org/). The database includes data on the U5MR for all countries from VR systems, as well as data series constructed from other sources, most commonly surveys or censuses that collected summary or complete birth histories. Birth histories are collected in many developing countries that do not have well-functioning VR systems. They contain records of all births to a woman over her lifetime, as well as information on the survival status of her children. Complete birth histories list detailed information on dates of birth and death, while summary birth histories provide more limited information in the form of the total number of children ever born to a woman and the number of children that are still alive. Birth histories provide U5MR observations for years before the survey/census date based on various U5MR calculation methods. Specifically, so-called direct methods provide U5MR observations from complete birth histories, while indirect methods provide U5MR observations from complete or summary birth histories [3],[4]. Common large-scale household surveys that collect birth histories include Demographic and Health Surveys (DHS) and Multiple Indicator Cluster Surveys (MICS).

**Figure 1:**
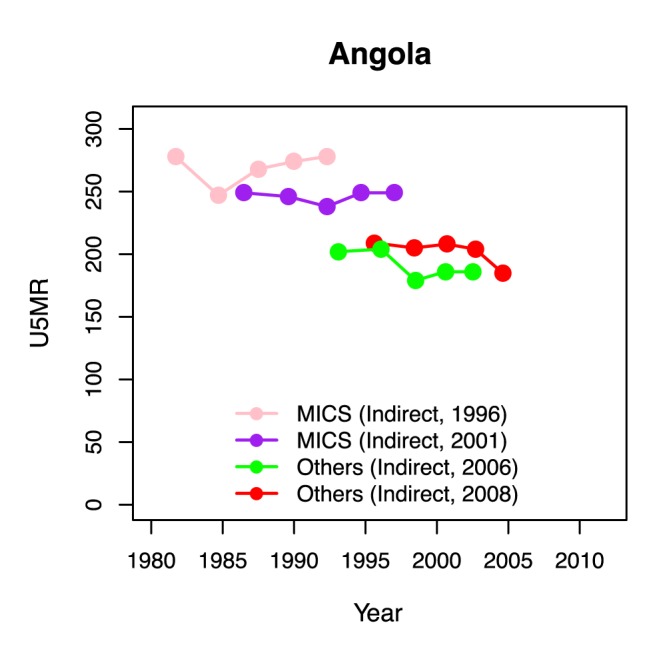
U5MR data series for Angola. Connected dots represent series of indirect estimates of U5MR from birth histories. The source and source date for each series is given in the legend.

Observations of child mortality vary within and between data series because of sampling and non-sampling errors. This is illustrated in Figure 1 for Angola. The four available data series in Angola overlap in time, but not in level. Consequently, some form of smoothing is required to construct U5MR estimates for a country for all years, based on all available data points. The default U5MR curve fitting approach used by the UN IGME in 2012 was loess (locally weighted least squares) regression, where a weighted linear regression model was fitted to the U5MR (on the natural logarithmic scale) for all years of interest. A smoothing parameter α determined the range of points included in each fit and their weights (the flexibility of the fitted trend line decreases with α). The default setting for α was based on the number of surveys/censuses and data points from VR in each country [5]. For a limited number of countries, the smoothing parameter α used in the loess trend fitting procedure was adjusted to better capture more recent trends in the data. An alternative estimation approach was used for countries with a generalized HIV/AIDS epidemic (defined as countries where HIV prevalence in the general population was more than 5% at any point in time since 1980, 17 countries in total) and for countries with conflicts or limited observations of dubious quality (four countries in total) [5].

In 2011, UIs were constructed for the UN IGME estimates for countries where the default method was used, based on the uncertainty in (the parameters of) the local log-linear fits. The resulting UIs were deemed implausible (too narrow) by experts for many countries, and therefore not published. One reason why uncertainty was likely to be underestimated in that approach is the ignorance of the structure of the U5MR data, specifically, potential biases in levels and trends of U5MR data series (explained in more detail in the next section). A second reason is the omission of the possibility that a specific data series is of inferior quality, and a third reason is the lack of inclusion of model uncertainty (i.e., the choice of span parameter α in the loess fit and expert adjustments, and, more generally, the choice of a loess smoother instead of a different curve fitting procedure).

We used a bootstrap method [6] for constructing uncertainty bounds for all countries where a standard loess fitting procedure was used (174 countries out of 195 countries for which the UN IGME published estimates in 2012). The new method improves upon the default method by taking into consideration the U5MR data structure as well as the possibility of data series that are of inferior quality. Model uncertainty is not evaluated (see Discussion).

### Bootstrap Procedure for Constructing Uncertainty Intervals

#### Simplified summary of bootstrap procedure

Because of errors in observed data series, true national child mortality rates are unknown. If there are many data series with small errors available in a given country, the UN IGME point estimates are likely to be very close to the true rates; the fewer the number of data series or the larger the errors in the data series, the further away the UN IGME estimates could be from the truth. Bootstrapped uncertainty bounds are based on the following assessment. Supposing that the UN IGME point estimates for a given country were equal to the truth, which data series could have been obtained in that country (instead of the data series included in the UN IGME database), and, from those, what UN IGME point estimates could have been constructed? For countries with a small number of data series that are deemed to have large errors, the range of data series that could have been obtained will range widely, and, consequently, a wide range of UN IGME point estimates could have been obtained. On the other hand, in countries with many data series of high quality, estimates for a given year will tend to be more similar. Bootstrapped uncertainty bounds are based on the set of estimates that could have been obtained for each country, based on the scenario that the UN IGME estimates are equal to the truth, and reflect the data availability in the country, as well as the likely errors in the available data series, based on an assessment of biases in data series.

#### In-depth explanation of bootstrap procedure

Bootstrapping refers to creating a large number of “new” datasets (that could have been observed instead of the dataset at hand), and then repeating the curve fitting procedure to obtain a large number of point estimates in the form of U5MR trajectories. The set of “bootstrapped” trajectories illustrates the uncertainty associated with the original estimates. Associated 90% uncertainty bounds for the U5MR are obtained by selecting the 5th and 95th percentiles of the bootstrapped trajectories. Similarly, the UI for the ARR for a country in a given period is obtained by selecting the 5th and 95th percentiles of the bootstrapped ARR estimates, where each bootstrapped ARR estimate is calculated from one bootstrapped U5MR trajectory.

The difficulty in the bootstrap procedure is the first step: how to generate “new” datasets that lead to a similar variability in point estimates as the dataset at hand. Non-parametric and parametric bootstrapping procedures incorporate different approaches to the sampling step. In the non-parametric bootstrap procedure, data are resampled from the original dataset with replacement. This procedure is not easily applicable in the U5MR estimation context because U5MR observations are organized in series, or distinct sampling units. That is, surveys and censuses that collect birth histories provide a number of U5MR data points, as illustrated in Figure 1 for Angola. If we were to resample from the original dataset with replacement, we would need to resample the data series (instead of the observations). This approach is not feasible for U5MR estimation for most countries because of the limited number of data series in each country (e.g., there are only four series in Angola).

In this study, we used a parametric bootstrap procedure to generate new datasets [6]. In this approach, data are sampled based on a probability model for the data (hereafter referred to as the “data model”), replacing the unknown parameters in the data model by point estimates based on the sample at hand. The approach is most easily understood in a simplified example: in a patient study of disease status, new data (measurements of the disease outcome) are generated in a parametric bootstrap by simulating data from a Bernoulli distribution where the probability of having the disease is given by the point estimate from the current sample at hand.

Potential biases in trends and levels of U5MR data series need to be accounted for in the data model for the U5MR, as illustrated in Figure 1 for Angola. The four data series in Angola overlap in time, but not in level. The illustration suggests that some data series might systematically over- or underestimate the true U5MR with respect to the mortality level and/or trend. We used a multilevel modeling approach to estimate the mean and variability in biases in levels and trends, as well as additional error variance. Based on potential differences in biases, non-VR data were categorized into eight different “source types” by data source and U5MR calculation method (namely, DHS Direct with reported sampling errors, DHS Direct without reported sampling errors, DHS Indirect, MICS Indirect, Census Indirect, Others Direct [including MICS and Census Direct], Others Indirect, and Other Source Types). For each source type, for each data series, biases in the trend were modeled as a linear function of the retrospective period of the observation in the survey (the difference between the observation reference date and the date of the survey/census). This approach was motivated by known problems with retrospective data, such as the occurrence of recall biases and violations of modeling assumptions when calculating indirect U5MR observations. Details of the U5MR data model are given in Text S1. In short, the data model for observation *i* is given as follows:
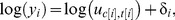
(1)where *y_i_* is the observed U5MR, 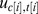 is the true U5MR in country *c*[*i*] and year *t*[*i*], and exp(δ*_i_*) represents the relative difference between the true and observed U5MR. The log-difference δ*_i_* for non-VR data is modeled as follows:
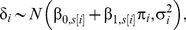
(2)where the mean function 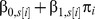 represents the bias in level and trend as a function of the retrospective period π*_i_* for observation *i* in data series *s*[*i*], and 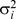 represents the error variance (a combination of sampling and non-sampling variance, where sampling variance is given for a large subset of the DHS Direct series). Using the UN IGME 2012 estimates for the true U5MR, we obtained parameter estimates for the distribution of the random intercept β_0,*s*_ and slope β_1,*s*_ for each data series by source type, and for 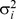.

For countries with data from VR systems, the log-differences δ*_i_* for VR observations are modeled as either random draws from a normal distribution or realizations from a time series process (for countries where the loess smoother did not adequately capture temporal fluctuations).

Based on the estimates of mean bias in levels and trends and error variance by source type, as well as the variability of biases across data series, “new” data series were sampled around the current UN IGME estimates in the first step of the bootstrap procedure, after which the loess smoother was fitted to the bootstrapped dataset. Instead of resampling all observed non-VR data series in the country in each bootstrap, one randomly selected data series was left out for countries with at least three data series. The leave-one-out step was motivated by the issue that an included data series could have been of low quality. By leaving out one series at a time at random, the influence of any one series on the resulting curve fit is reduced.

The dataset consisted of 867 data series and 8,336 observations for 174 countries where the UN IGME loess estimation procedure was used. An overview of the number of data series and observations is given in Table 1. Additional details on the bootstrap procedure are given in Text S1.

**Table 1:** Overview of the number of data series and observations by source type.

Source Type	Number of Data Series (Number of Observations)
VR	96 (3,209)
DHS Direct (with reported sampling errors)	185 (2,580)
DHS Direct (without reported sampling errors)	29 (94)
Others Direct (including MICS and Census Direct)	118 (355)
DHS Indirect	10 (50)
MICS Indirect	66 (318)
Census Indirect	178 (887)
Others Indirect	127 (623)
Other Source Types	58 (220)

### Validation of Uncertainty Bounds

Model performance for the default method and the proposed bootstrap method of constructing UIs was assessed based on the UN IGME 2011 dataset [2] by applying the modeling approach to a subset of the observations (called the training set) and then verifying how accurate the U5MR/ARR bounds were by comparing them to those obtained with the complete dataset. Given the retrospective nature of U5MR data and the occurrence of data in data series, the training set was not constructed by leaving out observations at random, but based on all data available in some year in the past, here 2006 [7]. To construct the training set, all data that were collected in or after 2006 were removed. For example, if a DHS survey was carried out in 2006, all (retrospective) observations from that DHS survey were left out of the training set. The observations that were left out of the training set formed the test set. To construct UIs using data in the training set only, we fitted the loess smoother to the data from the training set and carried out the bootstrap procedure. This approach resulted in UIs that would have been constructed in 2006 based on the proposed method. To validate the bounds, we calculated how often the UN IGME 2011 estimates [2] were inside previously constructed UIs for the U5MR in 1990, 2000, and 2005, and for the ARR from 1990 to 2005.

### Interpreting UIs for the ARR

The goal of constructing UIs is to enable and promote evidence-based assessments of progress toward reducing U5MR. We propose to categorize countries based on the UI for their ARR for 1990 to 2011 into five categories, as summarized in Table 2. In this categorization, category 1 includes all countries for which no conclusion at all can be drawn about progress made in reducing U5MR (the UI for the ARR from 1990 to 2011 is very wide, ranging from less than 0% to more than 4.4%). For countries in category 2, it is not clear whether any progress has been made in reducing U5MR, and it is deemed unlikely that the country is on track for MDG 4. For countries in categories 3 and 4, there is evidence that progress has been made, but the categories differ based on the upper bound for the ARR: in category 3 it is deemed unlikely that the countries are on track for MDG 4 based on available data, while this is yet to be determined for the countries in category 4. Category 5 includes all countries for which it is deemed likely that the country is on track to reach the MDG 4 target (the lower bound for the ARR from 1990 to 2011 is above 4.4%).

**Table 2:** Categorization of countries based on evidence for progress in reducing U5MR and accomplishing the MDG 4 target of an ARR of 4.4% from 1990 to 2011.

Category	ARR 1990–2011		Evidence of Progress?	
	Lower Bound (L)	Upper Bound (U)	Progress in Reducing U5MR?	Progress at ARR of 4.4% or Above?
1	L≤0%	U≥4.4%	Not clear (estimate of ARR is highly uncertain)	
2	L≤0%	0%≤U<4.4%	Not clear	Unlikely
3	0%<L<4.4%	U<4.4%	Likely	Unlikely
4	0%<L<4.4%	U≥4.4%	Likely	Not clear
5	L≥4.4%	U≥4.4%	Likely	Likely

The analysis was carried out in open source software R [8] and WinBUGS [9].

## Results

### Analysis of Biases in U5MR Data Series

**Figure 2:**
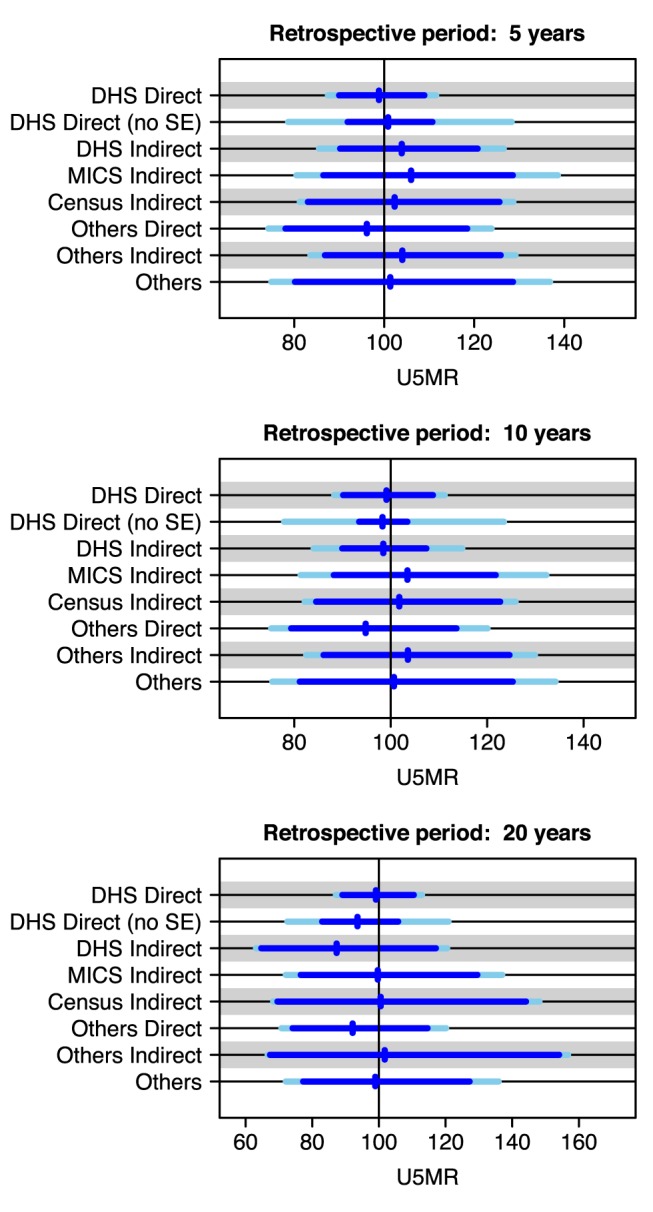
Visualization of 90% prediction intervals for new data points by source type and retrospective period. For a “true” U5MR of 100 deaths per 1,000 live births (represented by the black line), the 90% prediction interval for a U5MR observation is shown in light blue (for DHS direct series, this excludes the sampling variability), and the predicted mean observed U5MR is represented by the blue vertical line (the difference between the mean U5MR and 100 represents the mean bias). The blue horizontal line represents the 90% prediction interval for an observation based on uncertainty in the bias parameters only (excluding sampling and non-sampling variability). SE, sampling error.

Mean biases in U5MR levels and trends, as well as 90% prediction intervals for the expected range of U5MR values for a “new” U5MR data series, given a “true” U5MR of 100 deaths per 1,000 live births, are shown in Figure 2 for the different source types, for various retrospective periods. These plots were obtained based on parameter estimates from multilevel modeling. In general, the mean biases are considerable, but, more notably, the 90% prediction intervals based on uncertainty in biases alone (the blue horizontal lines) are wide, indicating substantial variability in biases across data series. For example, the prediction interval ranges from 88 to 122 deaths per 1,000 live births for an observation from a MICS Indirect series, with a retrospective period of 10 y. The error variance tends to contribute less to the width of the 90% prediction intervals, implying that there is significant variability in data series that is not attributed to random error. Half-widths of 90% prediction intervals for new observations tend to be at least 20% of the U5MR level for most source types. Across source types, there are considerable differences in the mean and variability of biases, confirming the need to distinguish data series by their source types for the generation of UIs. For example, a new data point with a retrospective period of 5 y is more likely to overestimate the true U5MR if it is from an indirect data series than if it is from a direct data series. Also, the 90% prediction intervals for a new data point of source type DHS Direct based on uncertainty in biases alone are narrower than those of other source types, indicating lower variability in biases across data series of source type DHS Direct than across data series of other source types.

### Uncertainty Intervals

**Figure 3:**
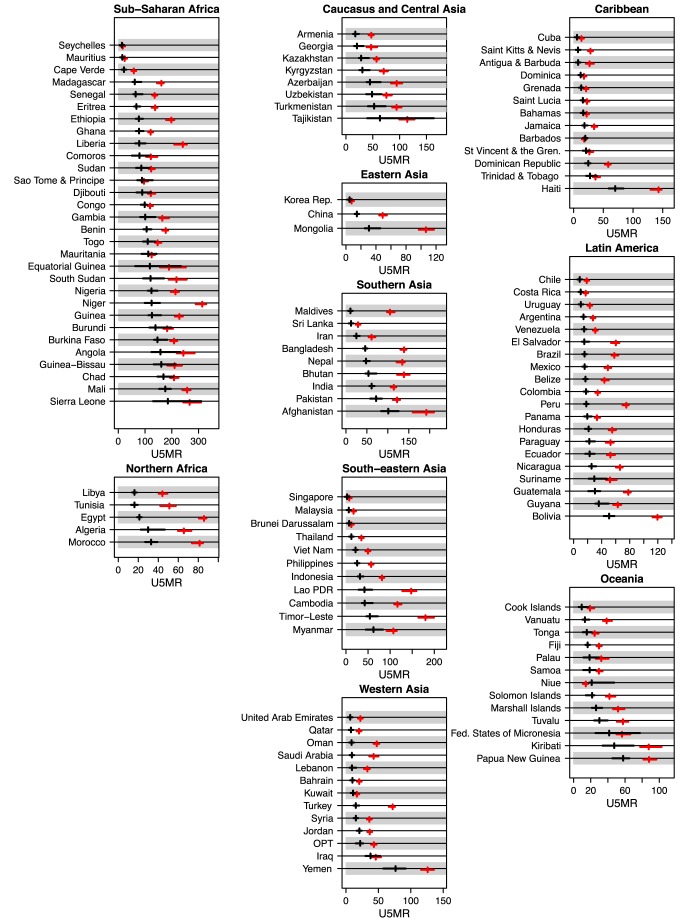
Point estimates and UIs for the U5MR in 1990 and 2011 for all developing countries, summarized by MDG region. Point estimates and UIs for the U5MR in 1990 (red) and 2011 (black). Within regions, countries are ordered by the point estimate of the U5MR for 2011. Lao PDR, Lao People's Democratic Republic; Korea Rep., Republic of Korea; OPT, Occupied Palestinian Territory; St Vincent & the Gren., Saint Vincent and the Grenadines.

The UIs for all 174 countries are given in Figure S1 and Table S1. The U5MR UIs for 1990 and 2011 are given in Figure 3 for all developing countries (grouped by MDG region). We focus our discussion on the 90% UIs for 86 high mortality countries, here defined as countries with an estimated U5MR of at least 40 deaths per 1,000 live births in 1990, where the loess estimation method was used to construct the estimates (i.e., excluding the countries with a generalized HIV/AIDS epidemic and other countries where an alternative estimation approach was used). On average, there is more uncertainty about the U5MR in 2011 compared to 1990, both in absolute levels as well as relative to the level of U5MR. This is summarized in Table 3; the median absolute width of the UIs increased from 16 deaths per 1,000 live births in 1990 to 23 deaths in 2011. The median relative width of the UI is 48% for the U5MR in 2011, compared to 19% for 1990 (and 24% in 2000). The median width for the ARR from 1990 to 2011 is 2.2%.

**Table 3:** Overview of width of uncertainty bounds for U5MR in 1990, 2000, and 2011 for 86 high mortality countries.

Year	UI Width (U5MR)		UI Width Relative to U5MR (Percent)	
	Mean	Median	Mean	Median
1990	21	16	20	19
2000	21	16	25	24
2010	31	23	52	48

High mortality countries are defined by a U5MR of more than 40 deaths per 1,000 live births in 1990, where U5MR estimates were constructed using the standard UN IGME method. The width is reported in number of deaths per 1,000 live births. Relative width is reported as a percentage of the U5MR point estimates.

The increase in uncertainty from 1990 to 2011 in the U5MR can be explained by more limited data availability for recent years. For the 86 high mortality countries, out of a total of 4,528 observations used for the UN IGME loess estimation procedure, 1,283 observations have reference dates in the 1980s, and 1,148 observations have reference dates in the 1990s. In contrast, only 483 observations were available since the year 2000. The median extrapolation period (number of years between the last available observation and the year 2011.5) for these countries is 5.2 y.

**Figure 4:**
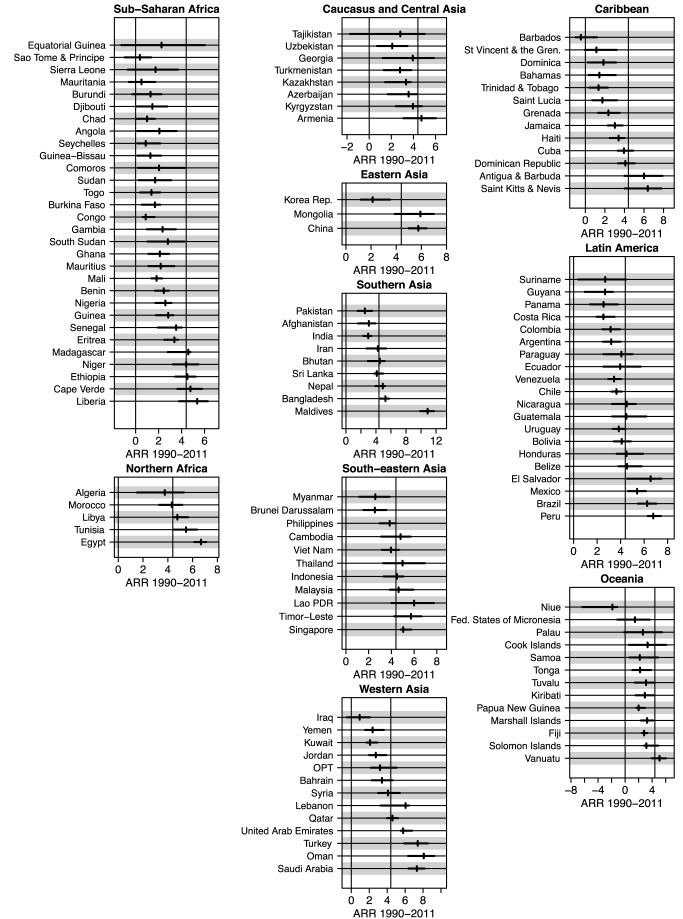
Point estimates and UIs for the ARR from 1990 to 2011 for all developing countries, summarized by MDG region. Within regions, countries are ordered by the lower bound of the UI. Lao PDR, Lao People's Democratic Republic; Korea Rep., Republic of Korea; OPT, Occupied Palestinian Territory; St Vincent & the Gren., Saint Vincent and the Grenadines.

**Figure 5:**
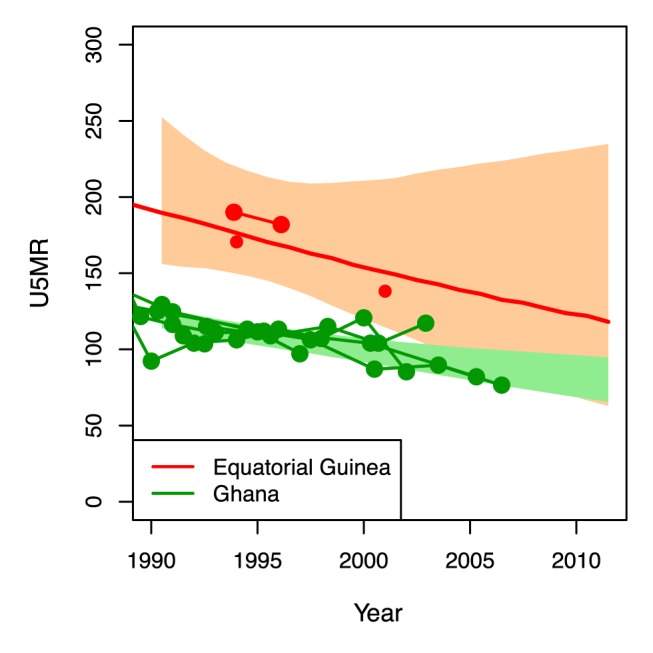
U5MR from 1990 to 2011 for Ghana and Equatorial Guinea. Point estimates (lines), UIs (shaded areas), and data series (connected dots) for Ghana (green) and Equatorial Guinea (red).

Figure 4 shows UIs for the ARR from 1990 to 2011 for all developing countries (grouped by MDG region), sorted by the lower bound of the ARR. The widths of the UIs vary greatly across countries. As alluded to in the Introduction, the figure illustrates that while the point estimates of the ARR may be similar for countries like Ghana and Equatorial Guinea (2.1% for Ghana and 2.3% for Equatorial Guinea), there is much greater uncertainty about the ARR in Equatorial Guinea. The lower bounds of these countries' respective UIs (1.1% for Ghana and −1.3% for Equatorial Guinea) show evidence of progress in Ghana compared to lack of information about the rate of reduction in Equatorial Guinea. The difference in data availability, as illustrated in Figure 5, is the main driver of the difference in uncertainty in ARR between the two countries.

**Figure 6:**
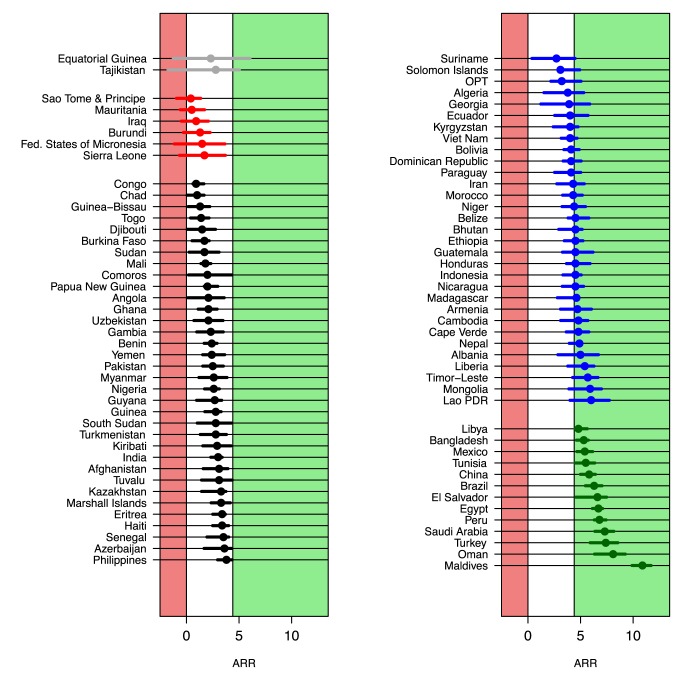
ARR from 1990 to 2011 for 86 high mortality countries. Countries are grouped based on the categories from Table 2 (lines colored grey, red, black, blue, and green for categories 1 to 5, respectively). Within each category, countries are sorted by the point estimate of the ARR. Lao PDR, Lao People's Democratic Republic; OPT, Occupied Palestinian Territory.

The categorization of Table 2 is illustrated in Figure 6 for the high mortality countries. For Equatorial Guinea and Tajikistan, in category 1, the estimate for the ARR is highly uncertain: there is insufficient evidence both to conclude that any progress has been made and to exclude the possibility that the country is on track to achieve MDG 4. For the six countries in category 2 (7% of the high mortality countries), there is also insufficient evidence to conclude that any progress has been made. In all countries in the remaining categories, there is evidence to conclude that progress has been made in reducing U5MR. In particular, while several countries in category 3 (34 countries, 40%) have ARR point estimates that are comparable to the point estimates to countries in categories 1 and 2, the lower bounds of the UIs for the category 3 countries do highlight that there is evidence of progress in reducing child mortality.

Based on the upper bounds of the UIs, the countries in categories 2 and 3 are not considered to be on track for meeting MDG 4. For the 31 countries in category 4 (36%), it is not clear whether they are on track for meeting the MDG 4 target or not. This category includes countries such as Suriname, with a point estimate for the ARR of 2.7%, as well as Lao People's Democratic Republic, with a much higher point estimate of 6.0%. The great uncertainty in the ARR in these two countries around the point estimate is explained by the scarcity of the data; in both countries, there are only two observations since 2000. Finally, for the 13 countries in category 5 (15%), there is evidence that the ARR has already exceeded the MDG 4 target of 4.4%. Maldives, the country with the highest point estimate for the ARR, 10.9%, is also the country with the highest lower bound for the ARR, 9.9%. The UI for the Maldives is relatively narrow (compared to other high mortality countries) because of greater data availability; the Maldives dataset includes a number of data series in the past decades, going back as far as the 1960s, and VR data from 2006 to 2011.

### Validation and Comparison of Uncertainty Methods

Validation measures for the U5MR and ARR (from 1990 to 2005) based on the UN IGME 2011 dataset are summarized in Table 4 for the bootstrap method and the default method to construct UIs. The results are presented for the 70 high mortality countries that were included in the validation exercise (for which observations were included in both the training as well as the test sets). In total, 18% of the data were left out for these countries for the training set (21% on average per country). For the bootstrap method, the updated ARR based on the UN IGME 2011 dataset ended up above the 90% UI that would have been constructed in 2006 for 16% of the countries (11 countries), while only 6% of the updated ARRs ended up below the previously constructed UIs. This suggests that the upper bound for the ARR was too low (too conservative with respect to estimating accelerated progress) in 2006, while the lower bound was reasonable. This is in line with the findings for the UIs for the U5MR in 1990, 2000, and 2005. The proportion of updated estimates for 2005 that fell below the UIs (17%) was higher than expected; the recent U5MR would have been overestimated in 2006 based on all data available at that time for a subset of countries. The results for the default method were qualitatively the same (the ARR would have been underestimated and recent U5MR would have been overestimated in 2006), but the results were substantially worse for the default method compared to the bootstrap method: the default UI for the ARR would not have contained the updated estimate for 40% of all high mortality countries.

**Table 4:** Validation results for the bootstrap method and the default method for high mortality countries based on the 2011 dataset.

Indicator	Bootstrap Method		Default Method	
	Below	Above	Below	Above
U5MR 1990	3	3	14	10
U5MR 2000	10	6	31	17
U5MR 2005	17	7	37	14
ARR 1990–2005	6	16	11	29

The percentage of high mortality countries for which the UN IGME 2011 estimate of the U5MR in 1990, 2000, and 2005, and the ARR for 1990–2005, falls below or above the 90% UI that would have been constructed in 2006 with all available data at that time. High mortality countries are defined by a U5MR of at least 40 deaths per 1,000 live births in 1990, where U5MR estimates were constructed using the standard UN IGME method; 70 high mortality countries were included in the exercise (with data in both training and test sets).

## Discussion

We used a bootstrap procedure to construct uncertainty bounds for the U5MR and the ARR for all countries, to provide more information about the evidence (or lack thereof) of countries' progress in reducing the U5MR. Our analysis was based on estimates of biases in levels and trends for different source types, and the variability therein. We found that there is substantial across-survey variation in biases and that half-widths of 90% prediction intervals for “new observations” tend to be at least 20% of the U5MR level for most source types. Ignoring these biases can lead to an underestimate of uncertainty in U5MR. We found that substantial uncertainty exists about U5MR levels and trends for high mortality countries, especially in more recent years; the median relative width of the UI compared to the U5MR level is 48% among 86 high mortality countries for 2011, compared to 19% in 1990. The greater uncertainty for recent years is explained by more limited data availability.

### Accuracy of Uncertainty Intervals

The validation of the UIs for the UN IGME 2011 dataset suggested that bounds that would have been constructed in 2006 based on all available data at that time are a substantial improvement upon the default method. In particular, the default bounds for the ARR that would have been constructed in 2006 would have been problematic (i.e., in categorizing countries as in Figure 6) because they would not have contained the updated point estimate for the ARR for 40% of the high mortality countries. The 90% bootstrapped bounds were more satisfactory (included an additional 13 out of 70 countries), but some drawbacks remain. Upper bounds for the ARR as constructed by the bootstrap method were too low for 16% of the high mortality countries (11 out of 70 countries); more recent estimates for the U5MR back in 2006 would have been too high for this set of countries. This set included countries such as Niger and Madagascar, where more recently collected data series suggest that progress toward reducing U5MR is accelerating. Given that projections are based on an extrapolation of more recent trends, such accelerations are challenging to project. While this finding implies that the upper bounds of the ARR might have to be treated with care, the validation exercise did not reveal any issues with the lower bounds for the estimated ARR.

We acknowledge that the bootstrapped bounds do not include the uncertainty associated with various steps in the UN IGME fitting procedure and are therefore likely to represent an underestimate of the uncertainty associated with the UN IGME estimates. A full uncertainty assessment is complicated because of set rules and expert adjustments in the UN IGME fitting procedure (the exclusion of outlying data, the rule for setting the span parameter α, and the adjustment of α for selected countries if the fit is deemed inappropriate). Instead of focusing attention and resources on trying to incorporate this uncertainty associated with various steps in the UN IGME fitting procedure into the uncertainty assessment, we favor exploration of alternative methods for estimating child mortality that incorporate an appropriate data model to reduce potential biases in the point estimates as well as the UIs.

### Assessing Uncertainty in Measures of Child Mortality

The main objective of our proposed method was to assess the uncertainty in countries with high U5MR, where point estimates can be volatile because of great uncertainty. We focused on countries where a standard U5MR estimation method was used. The UN IGME 2012 estimates of child mortality levels and trends [1] include UIs for all countries, based on our proposed method and additional assumptions for the construction of bootstrapped UIs for countries with nonstandard U5MR estimation methods (in particular, countries with high HIV prevalence or countries with adjustments related to crises or natural disasters). UIs for related child mortality indicators (the infant mortality rate, number of deaths, and aggregate estimates) were also included in the UN IGME 2012 estimates and were derived from the bootstrapped U5MR trajectories. Details on the additional methods for the construction of these UIs and the UIs for countries with high HIV prevalence and adjustments are given in Text S1. For neonatal mortality estimates, point estimates and associated uncertainty bounds in [1] were not obtained through the bootstrap approach; instead, the multilevel modeling approach proposed by Oestergaard and colleagues was used [10].

### Alternative Methods and Estimates

Alternative estimation methods and estimates of levels and trends in U5MR exist. In 2011, the Institute for Health Metrics and Evaluation (IHME) published national and global estimates of U5MR [11]. Differences between the 2011 UN IGME and IHME estimation approaches and resulting estimates are discussed in [12]. In short, the UN IGME and IHME estimation approaches differ with respect to data collection and the preprocessing of data, trend fitting procedures, inclusion and exclusion of data series, and additional adjustment procedures. Because of these differences in data and methods used, differences in point estimates occur (the difference in estimated U5MR between the two groups was more than 10% and corresponded to more than ten deaths per 1,000 live births for 10% of all countries in 1990 and 20% of all countries in 2010). The two main differences between the IHME approach to constructing uncertainty bounds and our bootstrap approach are the method used and the assumptions made about biases and error variance of observations. The IHME uncertainty bounds are obtained using Gaussian process regression, whereby trajectories of the log_10_–transformed U5MR over time are assumed to be realizations of a Gaussian process. These trajectories are constructed based on the assumption that observations from non-VR data series are conditionally independent (loosely interpreted, that the observations are randomly scattered around the U5MR trend line), which is different from our approach, in which we take account of potential biases in levels and trends in data series. A validation exercise of the uncertainty bounds that IHME constructed in 2010 (based on similar fitting methods and similar assumptions about data series) showed that while the IHME's point estimates were not biased, the IHME uncertainty bounds may be too narrow, which is potentially explained by their approach, which does not account for substantial across-survey variation in biases [7].

Country studies have been carried out using estimates of U5MR or ARR based on a single survey [13] or with estimates of ARR calculated from estimates of U5MR in two different years derived from two separate surveys [14]. Given sampling and non-sampling errors in data series, generally, we would caution against the use of point estimates based on a single study.

### Conclusions

Point estimates on child mortality based on limited information may substantially under- or overestimate the truth. Uncertainty assessments can and should be used to complement point estimates to avoid unwarranted conclusions about levels or trends in child mortality and to reduce confusion about differences in estimates, such as estimates from different groups such as the UN IGME and IHME, or after updating point estimates in light of new data. The new uncertainty assessments provide more insights into countries' progress in reducing child mortality. In particular, a comparison of the lower bound of the UI for the ARR across countries is more informative to pinpoint countries where we are confident that U5MR has declined since 1990, compared to ranking countries by their point estimates of the ARR (which can be highly uncertain, and thus arbitrarily high or low, for countries with limited data). In the coming years, including in 2015, UIs can be used to assess countries' progress toward MDG 4, as illustrated here (Table 2; Figure 6), and to evaluate whether the accomplishment of the target of an ARR of 4.4% is deemed unlikely, not clear, or likely based on all available data.

## Supporting Information

Figure S1 **U5MR from 1990 to 2011 for all 174 countries.** Data (colored connected dots), UN IGME 2012 point estimates (black dashed lines), UIs constructed using the bootstrap method (red shaded areas, labeled “new UIs”), and UIs constructed using the previous method (blue shaded areas, labeled “old UIs”). The source and source date for each data series are given in the legend.
(PDF)

Table S1 **Estimates and uncertainty intervals for all countries for the U5MR in 1990, 2000, and 2011, and the ARR from 1990 to 2011.**
(DOCX)

Text S1 **Supplementary information on the construction of the uncertainty intervals.**
(PDF)

## Abbreviations

ARR: annual rate of reduction
DHS: Demographic and Health Surveys
IHME: Institute for Health Metrics and Evaluation
MDG: Millennium Development Goal
MICS: Multiple Indicator Cluster Surveys
U5MR: under-five mortality rate
UI: uncertainty interval
UN IGME: United Nations Inter-agency Group for Child Mortality Estimation
VR: vital registration
