# Supplementary material for: Progress toward Global Reduction in Under-Five Mortality: A Bootstrap Analysis of Uncertainty in Millennium Development Goal 4 Estimates
Source: PLoS Med. 2012 Dec 11;9(12):e1001355. doi: 10.1371/journal.pmed.1001355 (PMC3519895; doi:10.1371/journal.pmed.1001355)
Supplement: Figure S1 — U5MR from 1990 to 2011 for all 174 countries. Data (colored connected dots), UN IGME 2012 point estimates (black dashed lines), UIs constructed using the bootstrap method (red shaded areas, labeled “new UIs”), and UIs constructed using the previous method (blue shaded areas, labeled “old UIs”). The source and source date for each data series are given in the legend. (PDF) [file pmed.1001355.s001.pdf]

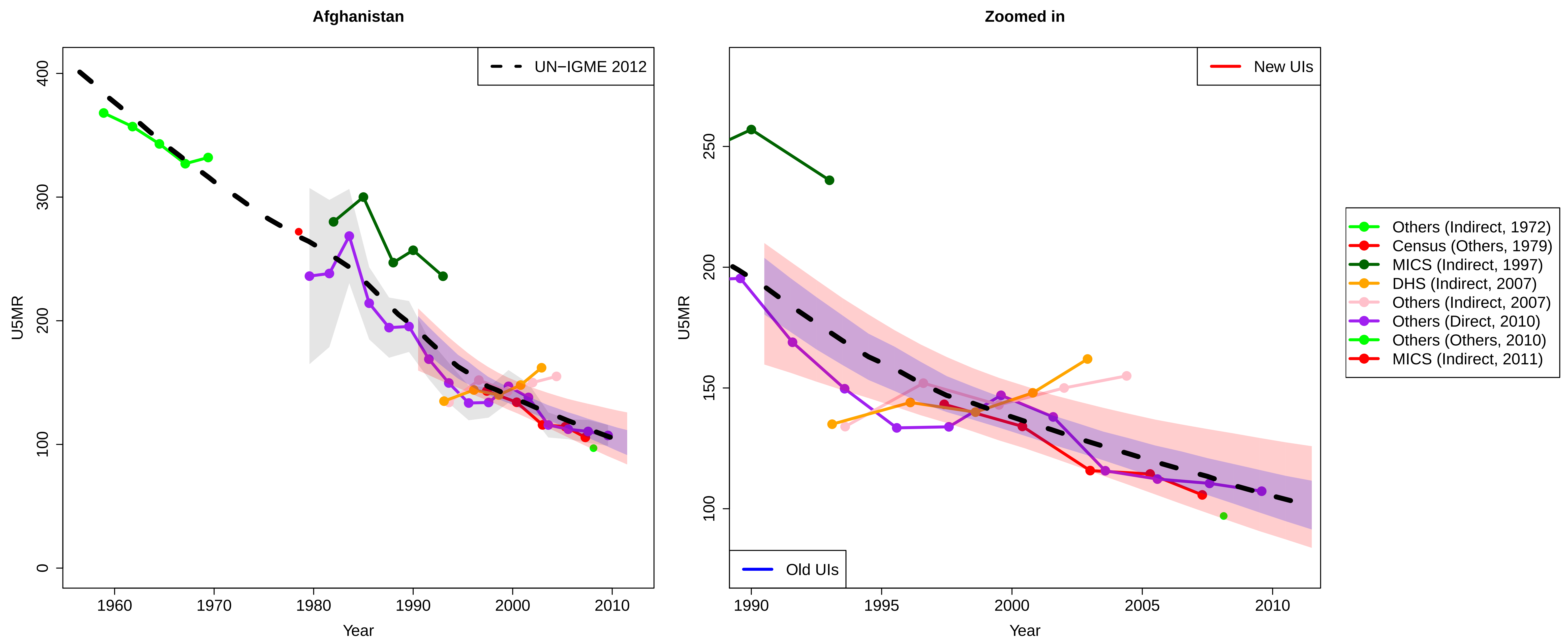

Afghanistan
Zoomed in
UN−IGME 2012
New UIs
Old UIs
U5MR
Year
Others (Indirect, 1972)
Census (Others, 1979)
MICS (Indirect, 1997)
DHS (Indirect, 2007)
Others (Indirect, 2007)
Others (Direct, 2010)
Others (Others, 2010)
MICS (Indirect, 2011)

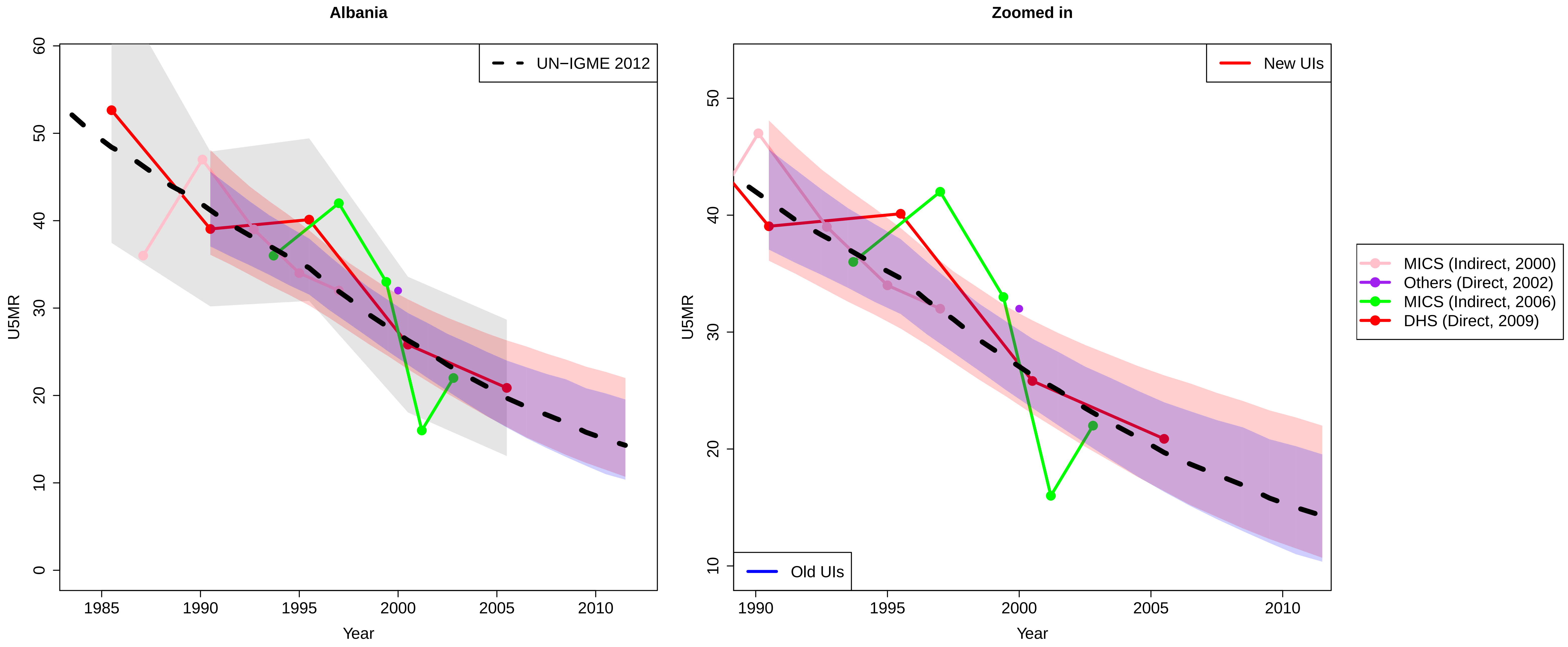

Albania
UN−IGME 2012
U5MR
Year
Zoomed in
New UIs
Old UIs
MICS (Indirect, 2000)
Others (Direct, 2002)
MICS (Indirect, 2006)
DHS (Direct, 2009)

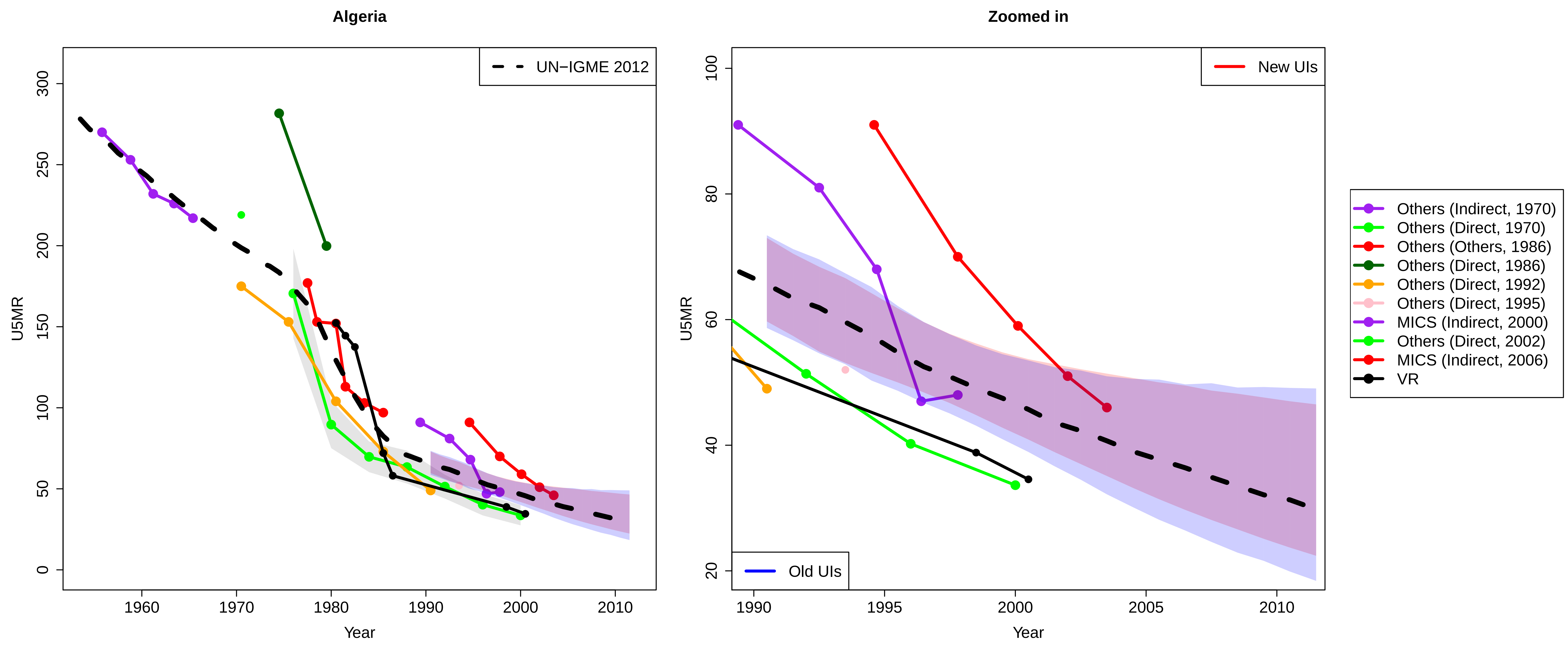
Algeria
UN−IGME 2012
U5MR
Year
Zoomed in
New UIs
Old UIs
Others (Indirect, 1970)
Others (Direct, 1970)
Others (Others, 1986)
Others (Direct, 1986)
Others (Direct, 1992)
Others (Direct, 1995)
MICS (Indirect, 2000)
Others (Direct, 2002)
MICS (Indirect, 2006)
VR

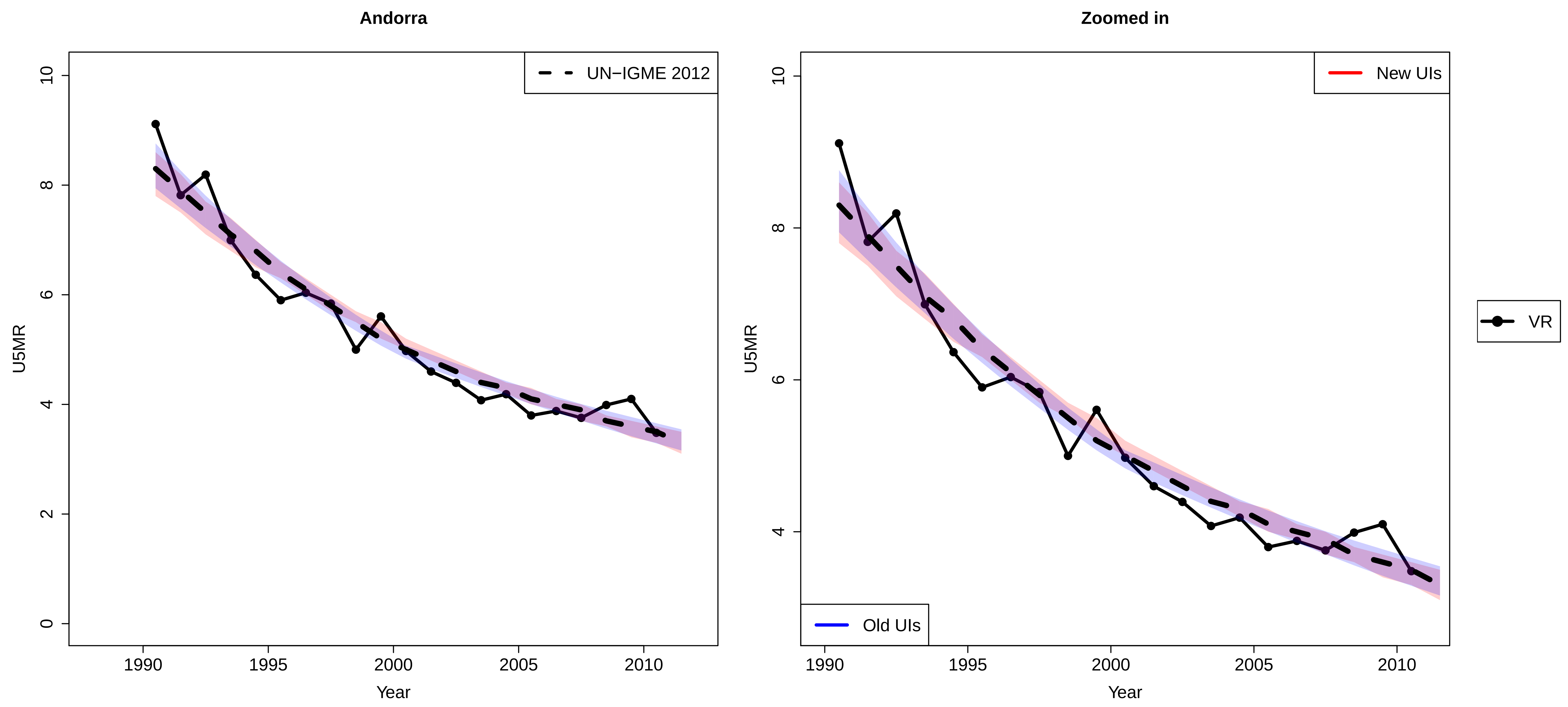
Andorra
UN−IGME 2012
U5MR
Year
Zoomed in
New UIs
Old UIs
VR
0
2
4
6
8
10
1990
1995
2000
2005
2010

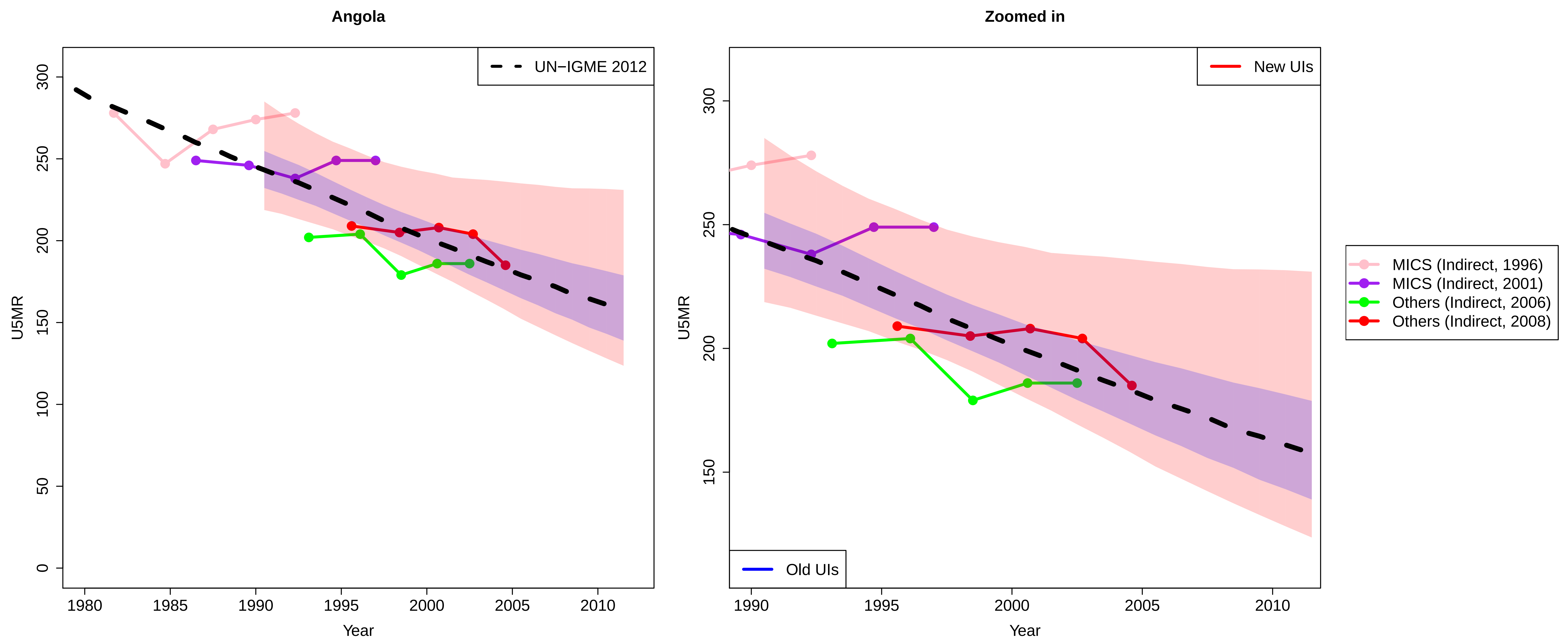
Angola
Zoomed in
UN−IGME 2012
New UIs
Old UIs
MICS (Indirect, 1996)
MICS (Indirect, 2001)
Others (Indirect, 2006)
Others (Indirect, 2008)
U5MR
Year

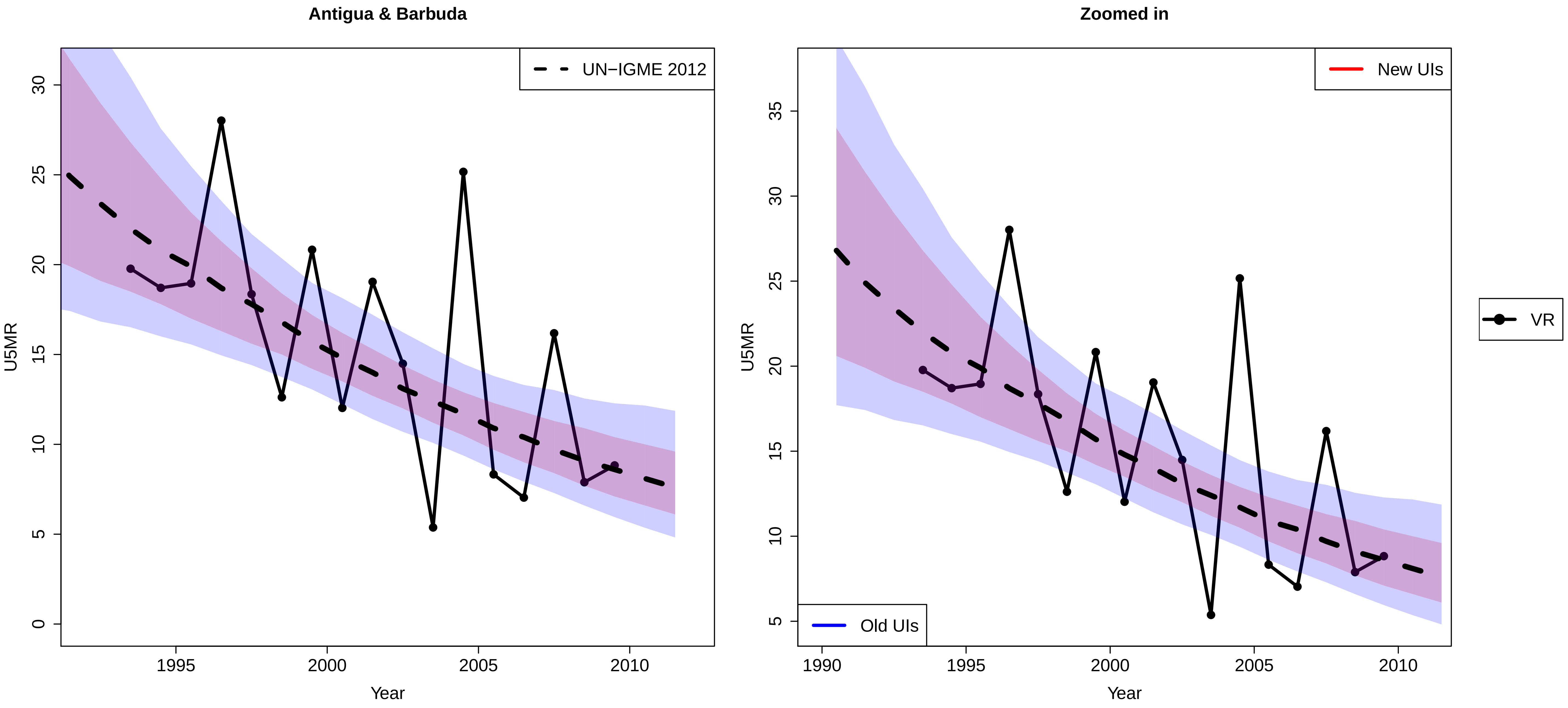
Antigua & Barbuda
UN−IGME 2012
U5MR
Year
Zoomed in
New UIs
Old UIs
VR

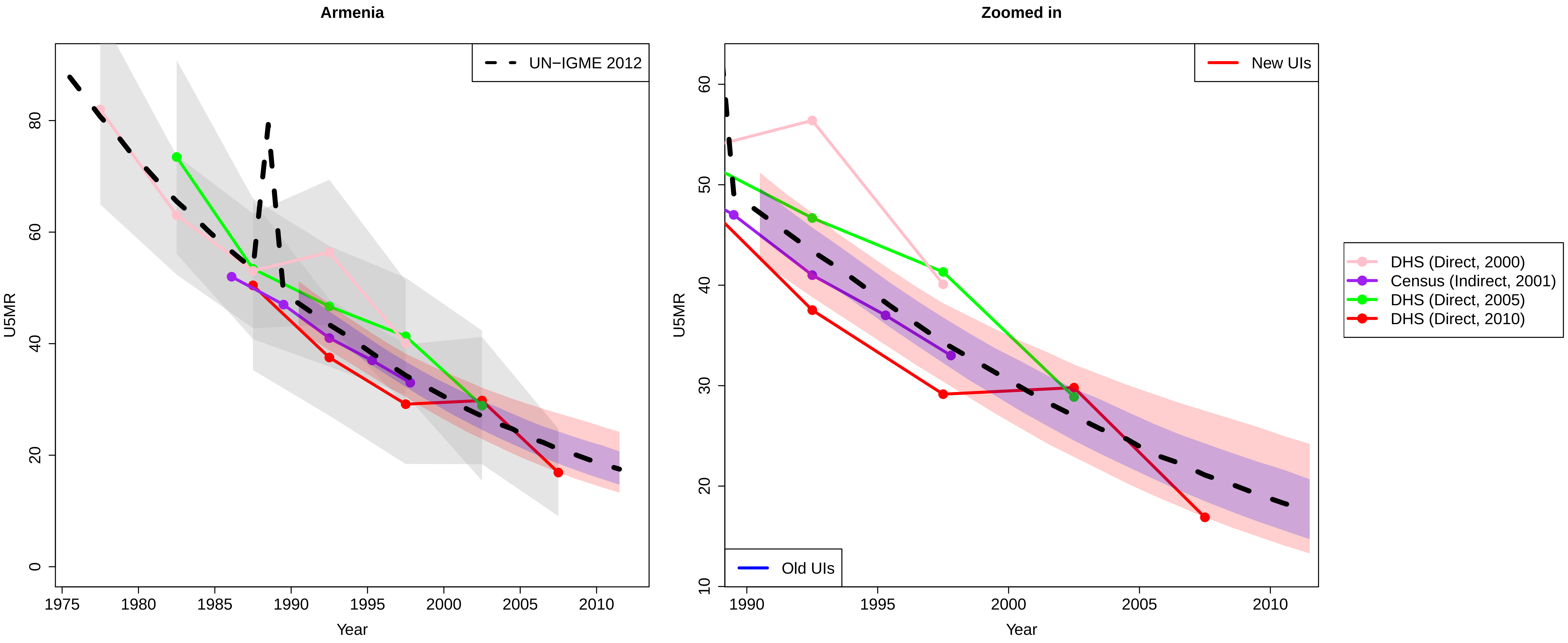

Armenia
UN−IGME 2012
U5MR
Year
Zoomed in
New UIs
Old UIs
DHS (Direct, 2000)
Census (Indirect, 2001)
DHS (Direct, 2005)
DHS (Direct, 2010)

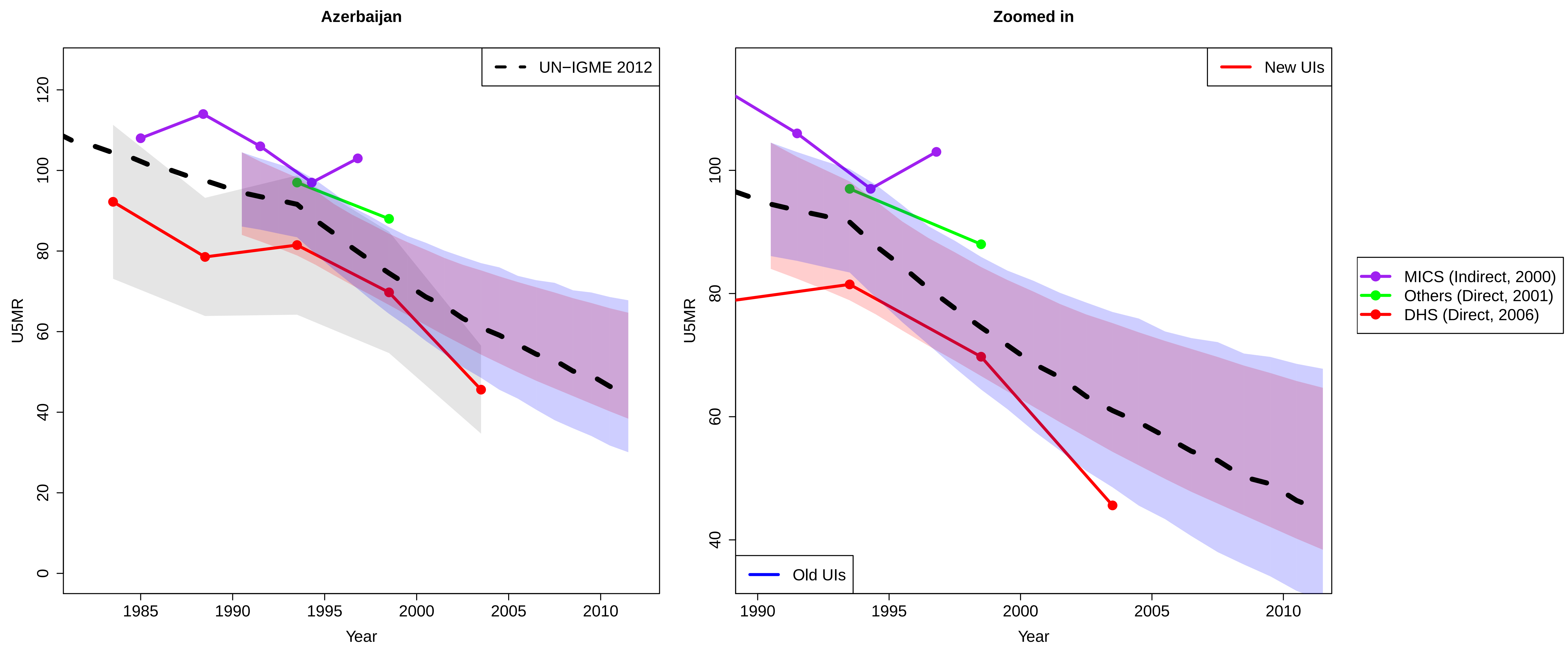

Azerbaijan
Zoomed in
UN−IGME 2012
New UIs
Old UIs
MICS (Indirect, 2000)
Others (Direct, 2001)
DHS (Direct, 2006)
U5MR
Year

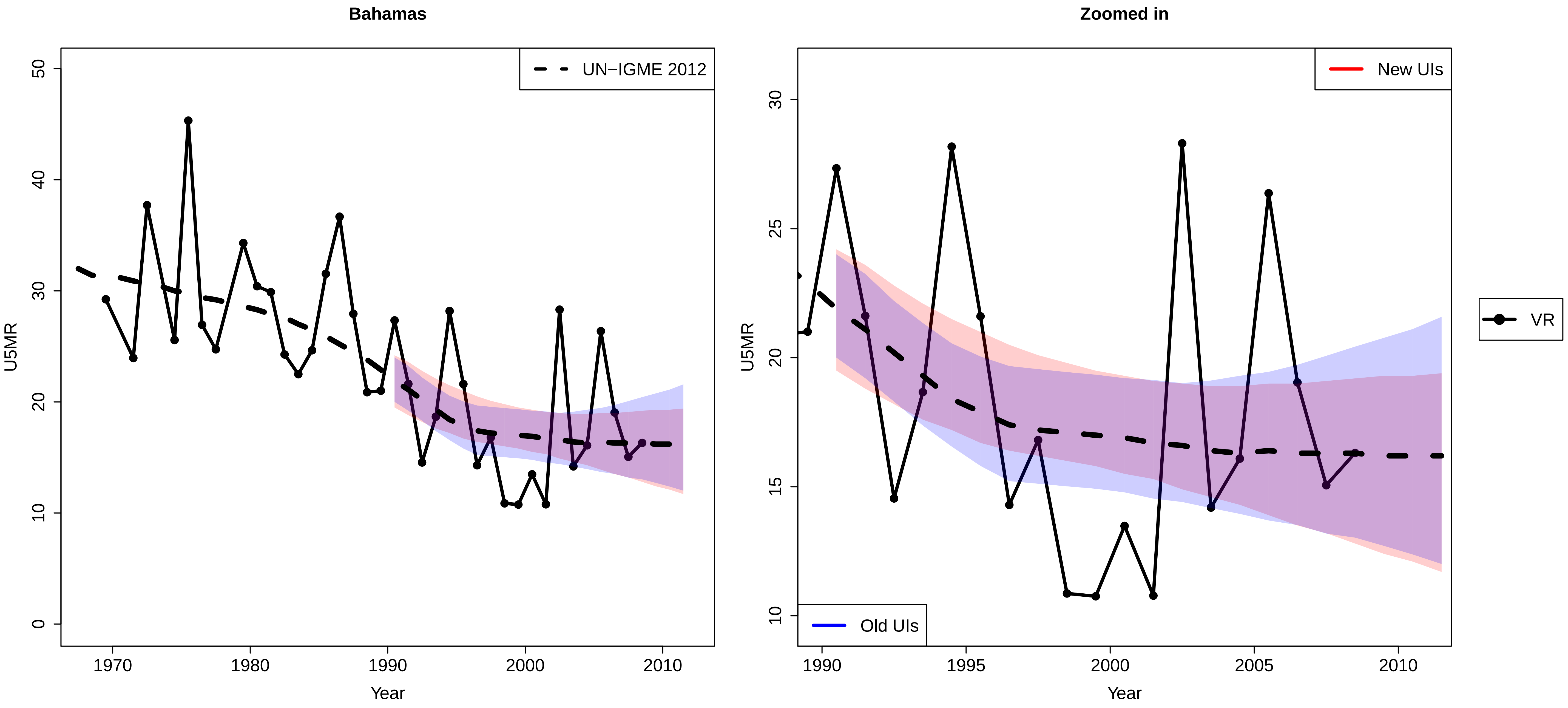

Bahamas
Zoomed in
UN−IGME 2012
New UIs
Old UIs
VR
U5MR
Year
0
10
20
30
40
50
1970
1980
1990
2000
2010
10
15
20
25
30
1990
1995
2000
2005
2010

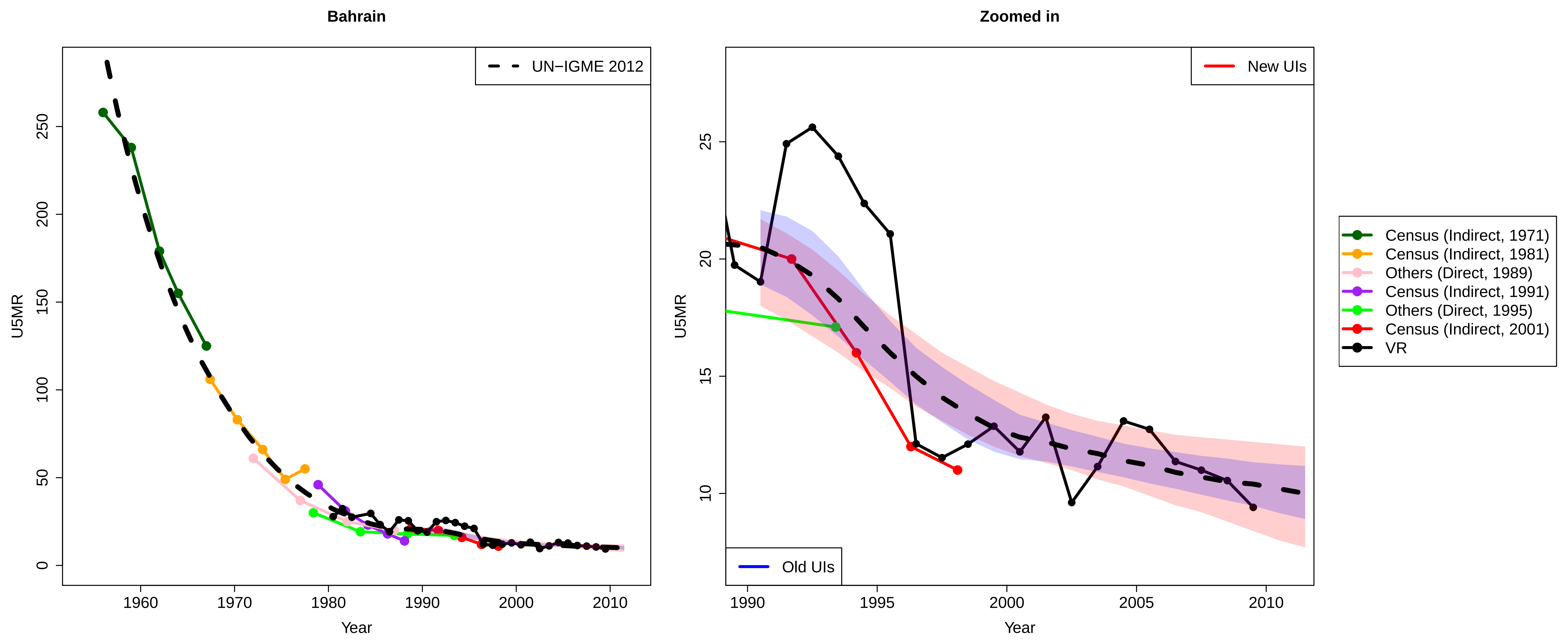

Bahrain
Zoomed in
UN−IGME 2012
New UIs
Old UIs
U5MR
Year
Census (Indirect, 1971)
Census (Indirect, 1981)
Others (Direct, 1989)
Census (Indirect, 1991)
Others (Direct, 1995)
Census (Indirect, 2001)
VR

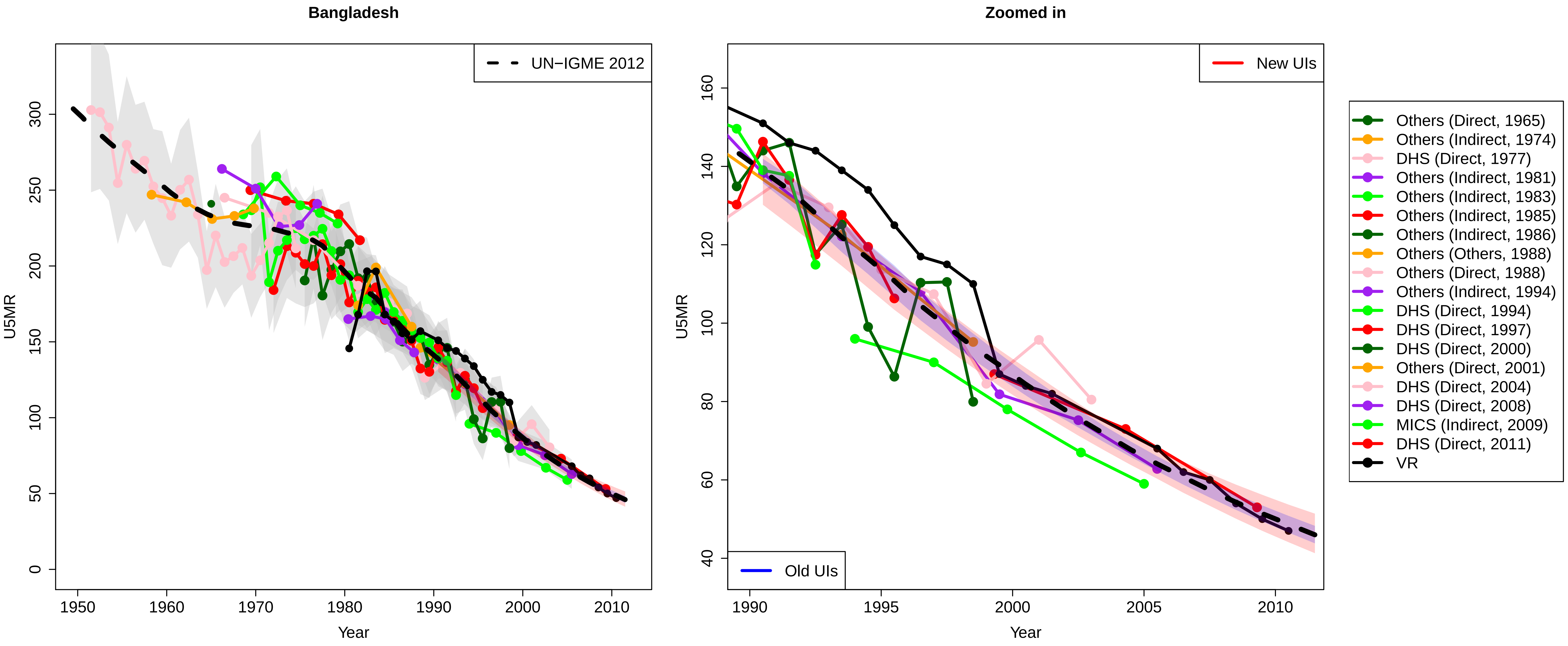
Bangladesh
Zoomed in
UN−IGME 2012
New UIs
Old UIs
U5MR
Year
Others (Direct, 1965)
Others (Indirect, 1974)
DHS (Direct, 1977)
Others (Indirect, 1981)
Others (Indirect, 1983)
Others (Indirect, 1985)
Others (Indirect, 1986)
Others (Others, 1988)
Others (Direct, 1988)
Others (Indirect, 1994)
DHS (Direct, 1994)
DHS (Direct, 1997)
DHS (Direct, 2000)
Others (Direct, 2001)
DHS (Direct, 2004)
DHS (Direct, 2008)
MICS (Indirect, 2009)
DHS (Direct, 2011)
VR

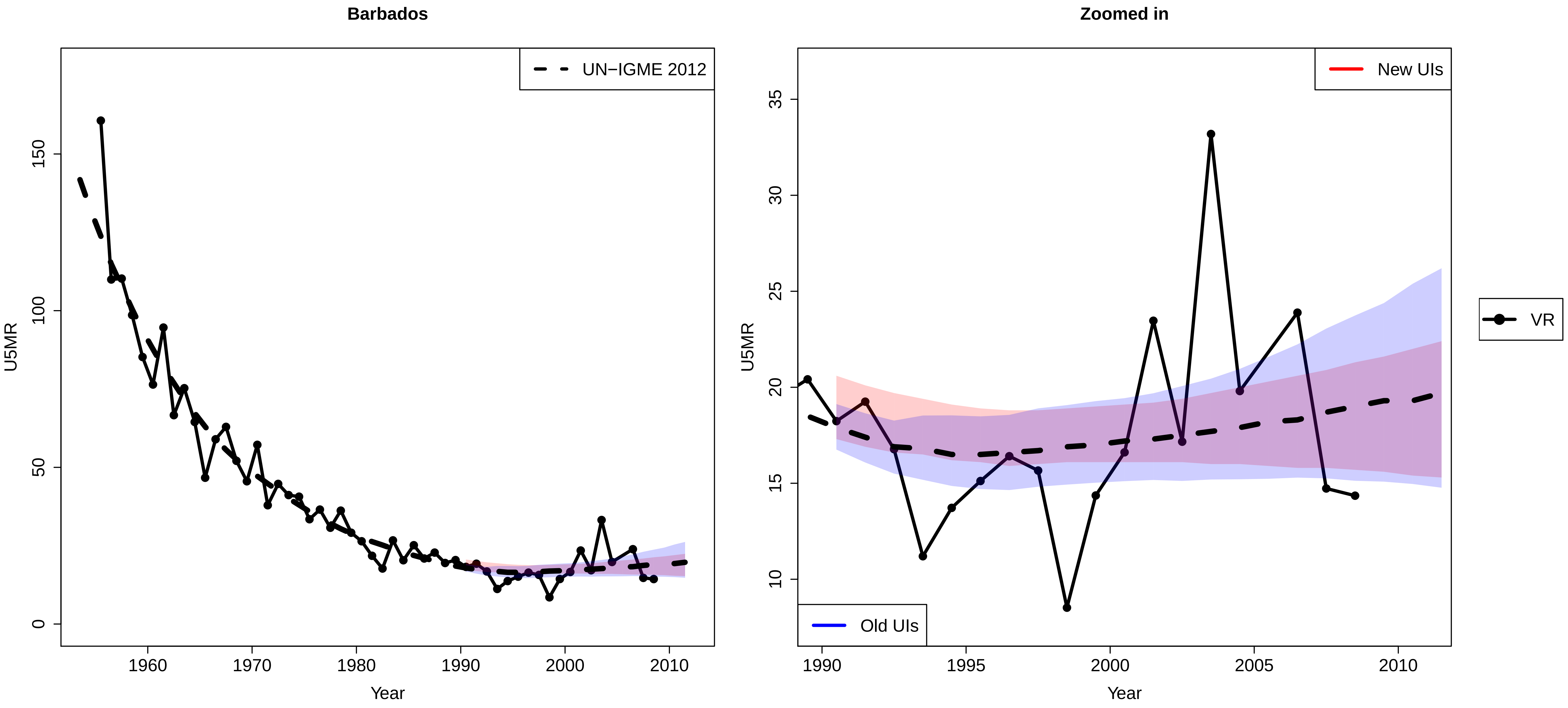
Barbados
Zoomed in
UN−IGME 2012
New UIs
Old UIs
VR
U5MR
Year
0
50
100
150
1960
1970
1980
1990
2000
2010
10
15
20
25
30
35
1990
1995
2000
2005
2010

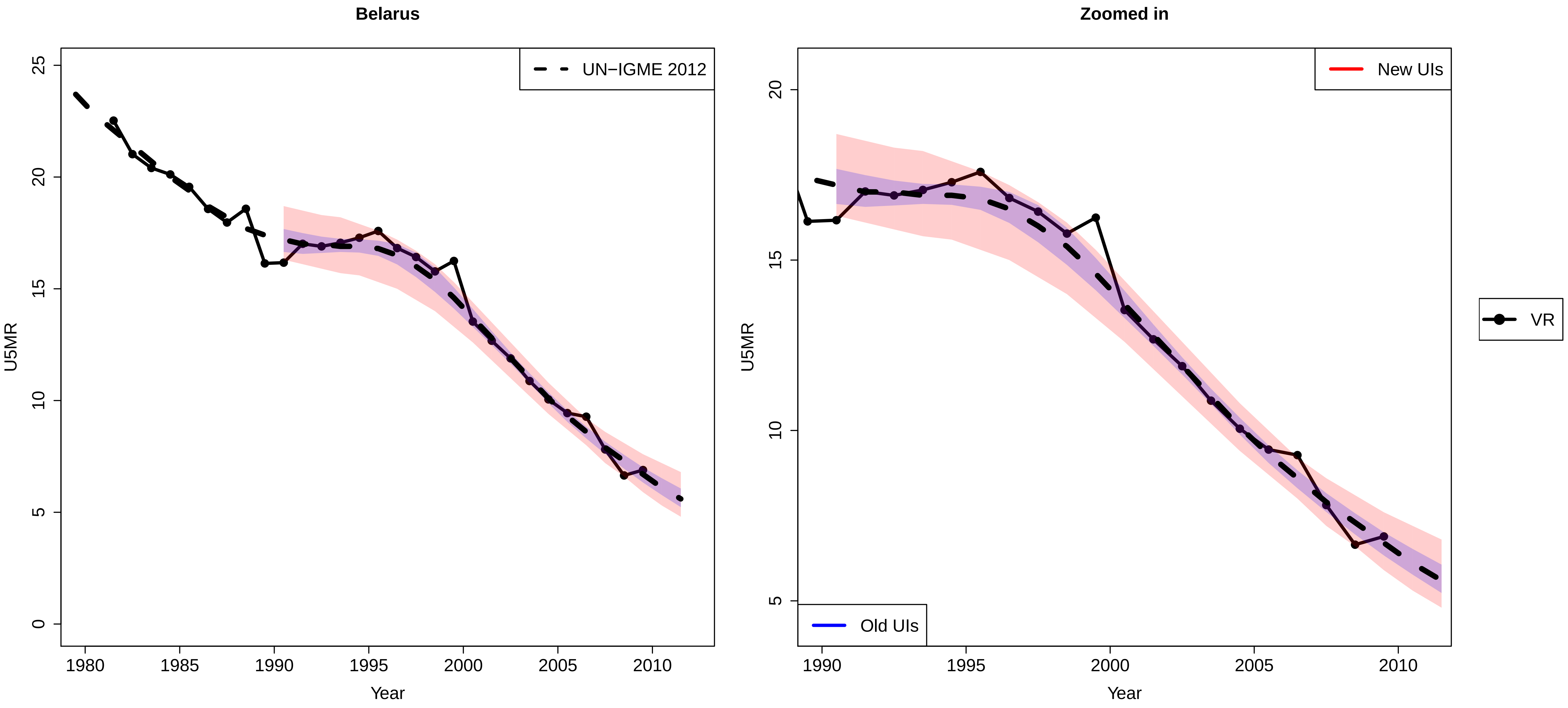

Belarus
Zoomed in
UN−IGME 2012
New UIs
Old UIs
VR
U5MR
Year
0
5
10
15
20
25
1980
1985
1990
1995
2000
2005
2010

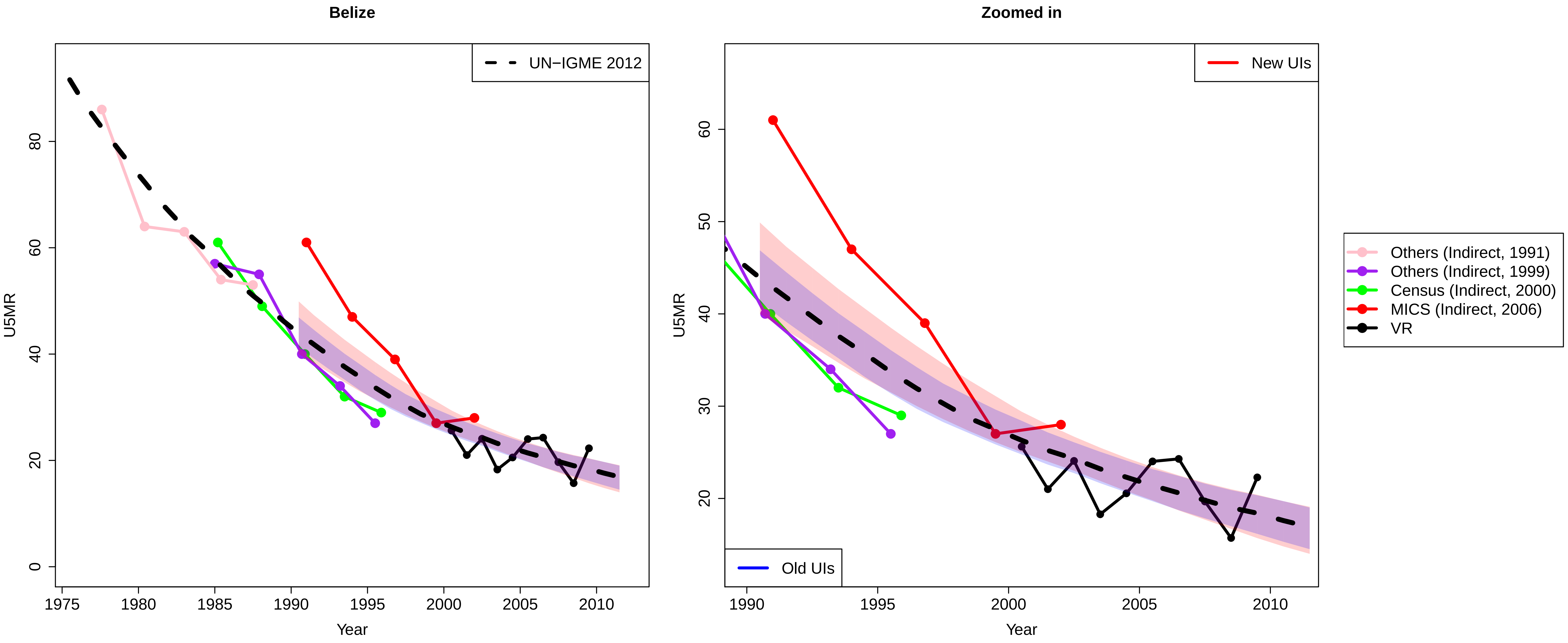

Belize
Zoomed in
UN−IGME 2012
New UIs
Old UIs
Others (Indirect, 1991)
Others (Indirect, 1999)
Census (Indirect, 2000)
MICS (Indirect, 2006)
VR
U5MR
Year

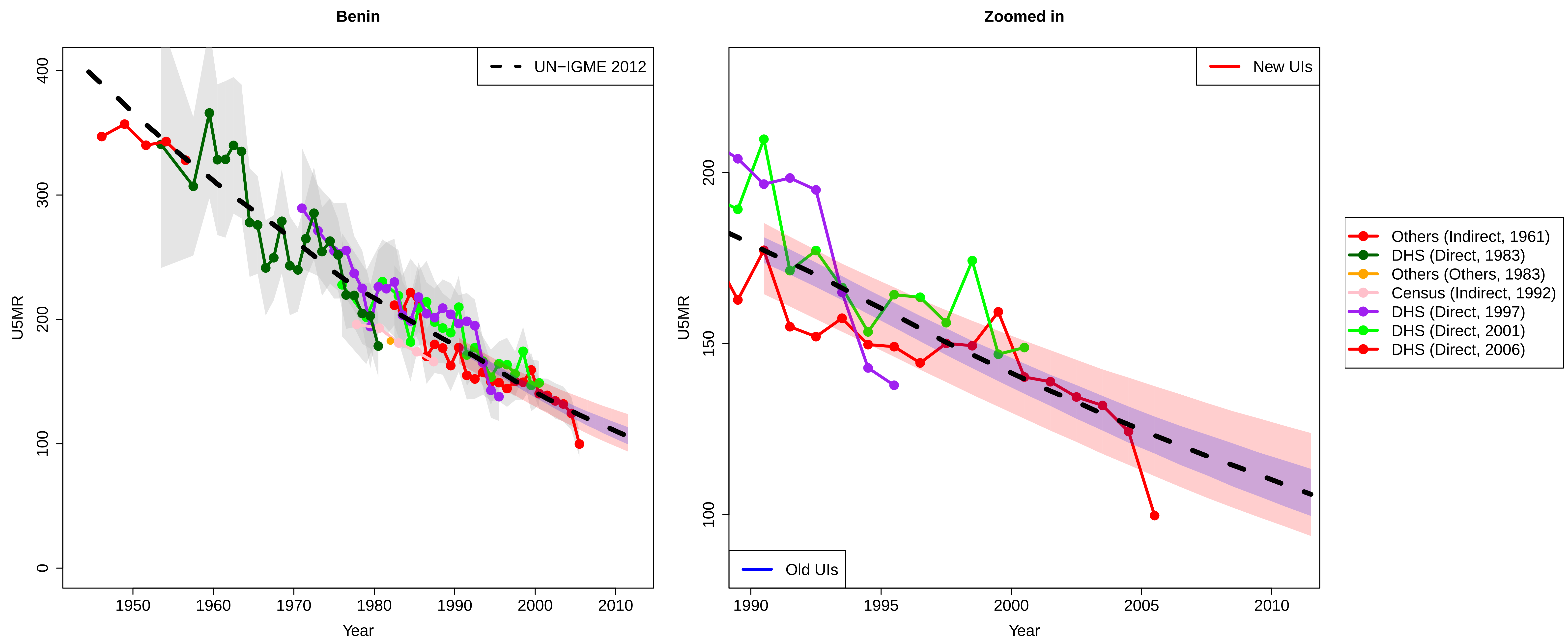

Benin
Zoomed in
UN−IGME 2012
New UIs
Old UIs
U5MR
Year
Others (Indirect, 1961)
DHS (Direct, 1983)
Others (Others, 1983)
Census (Indirect, 1992)
DHS (Direct, 1997)
DHS (Direct, 2001)
DHS (Direct, 2006)

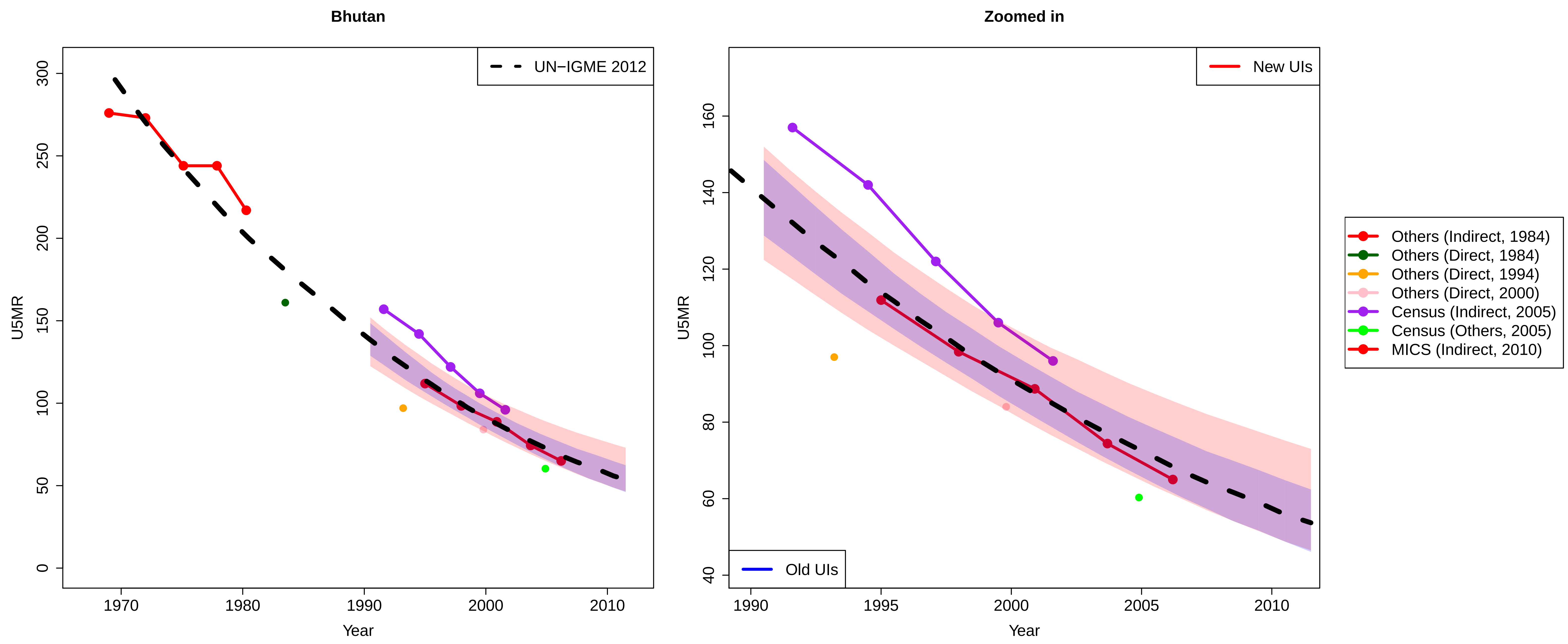
Bhutan
Zoomed in
UN−IGME 2012
New UIs
Old UIs
U5MR
Year
Others (Indirect, 1984)
Others (Direct, 1984)
Others (Direct, 1994)
Others (Direct, 2000)
Census (Indirect, 2005)
Census (Others, 2005)
MICS (Indirect, 2010)

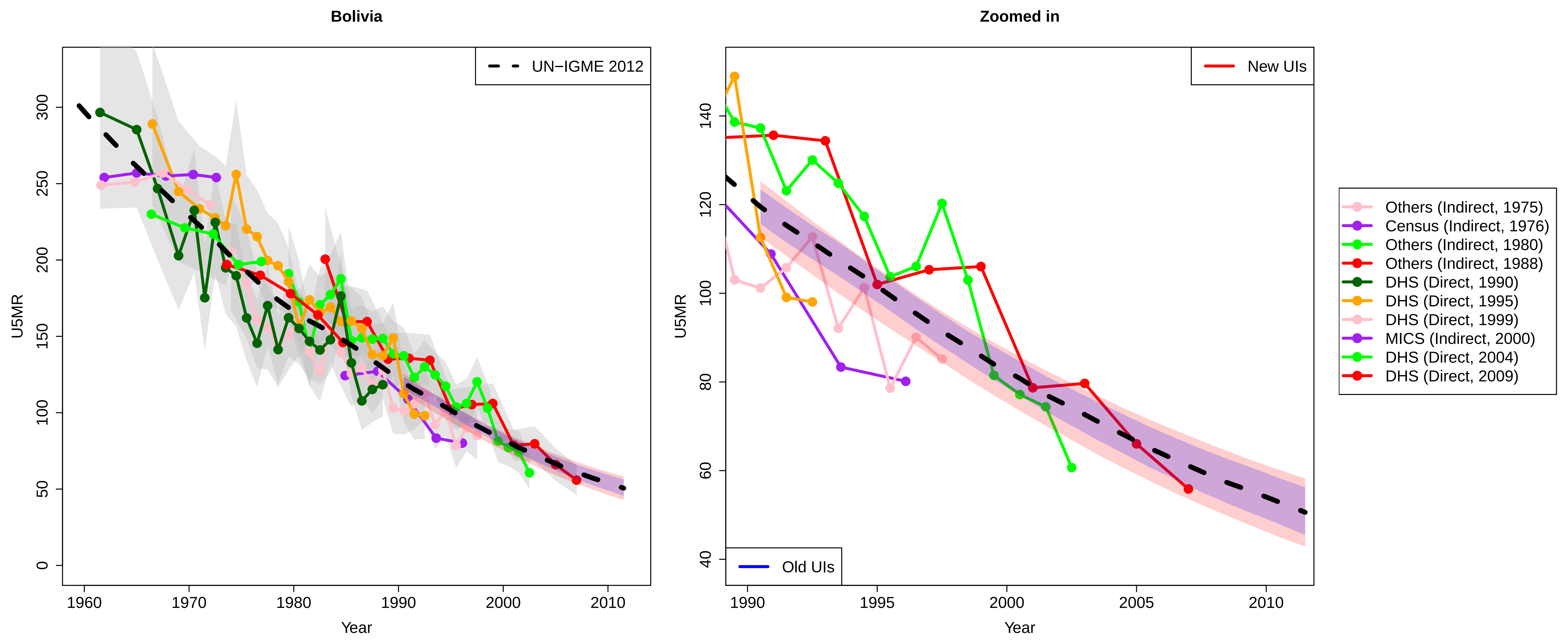
Bolivia
Zoomed in
UN−IGME 2012
New UIs
Old UIs
U5MR
Year
Others (Indirect, 1975)
Census (Indirect, 1976)
Others (Indirect, 1980)
Others (Indirect, 1988)
DHS (Direct, 1990)
DHS (Direct, 1995)
DHS (Direct, 1999)
MICS (Indirect, 2000)
DHS (Direct, 2004)
DHS (Direct, 2009)

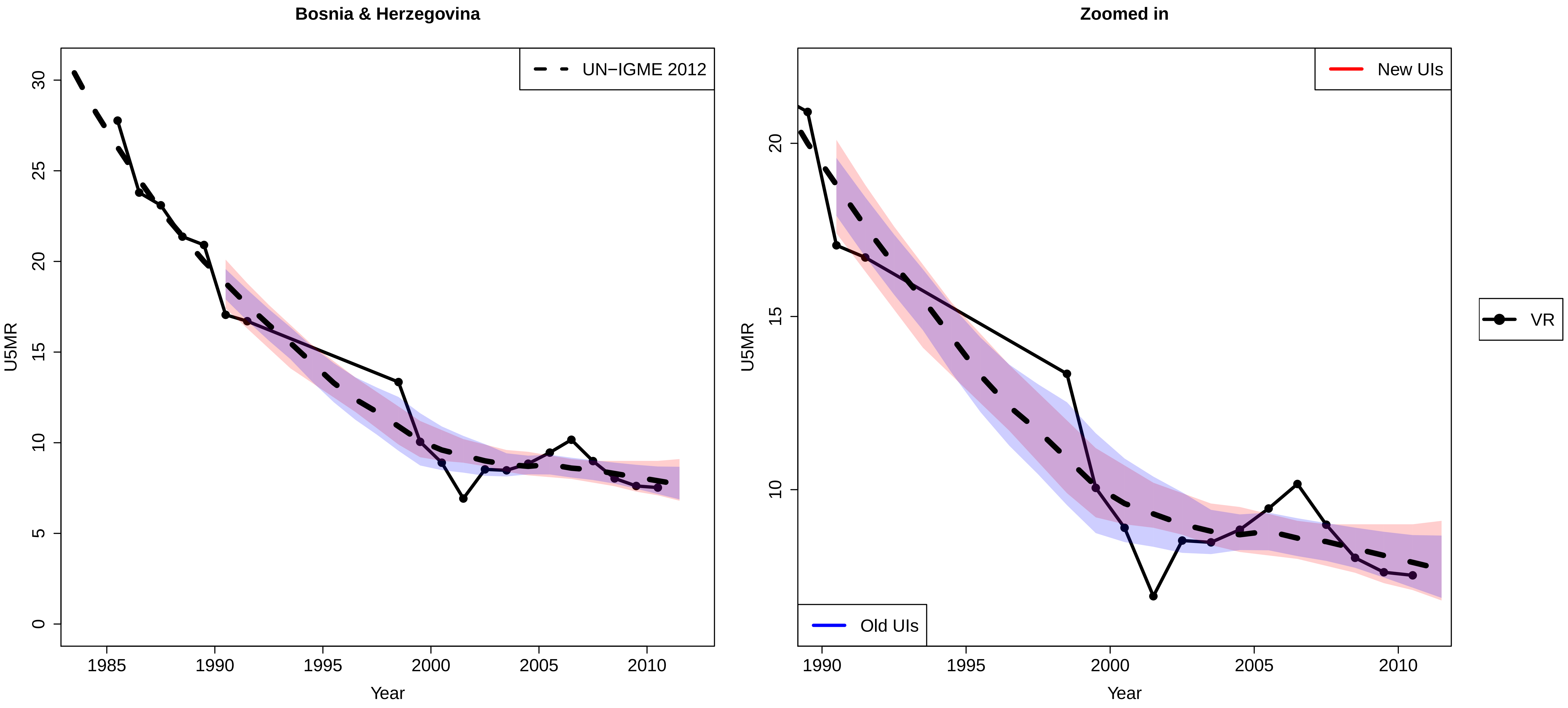
Bosnia & Herzegovina
UN−IGME 2012
U5MR
Year
Zoomed in
New UIs
Old UIs
VR
0
5
10
15
20
25
30
1985
1990
1995
2000
2005
2010

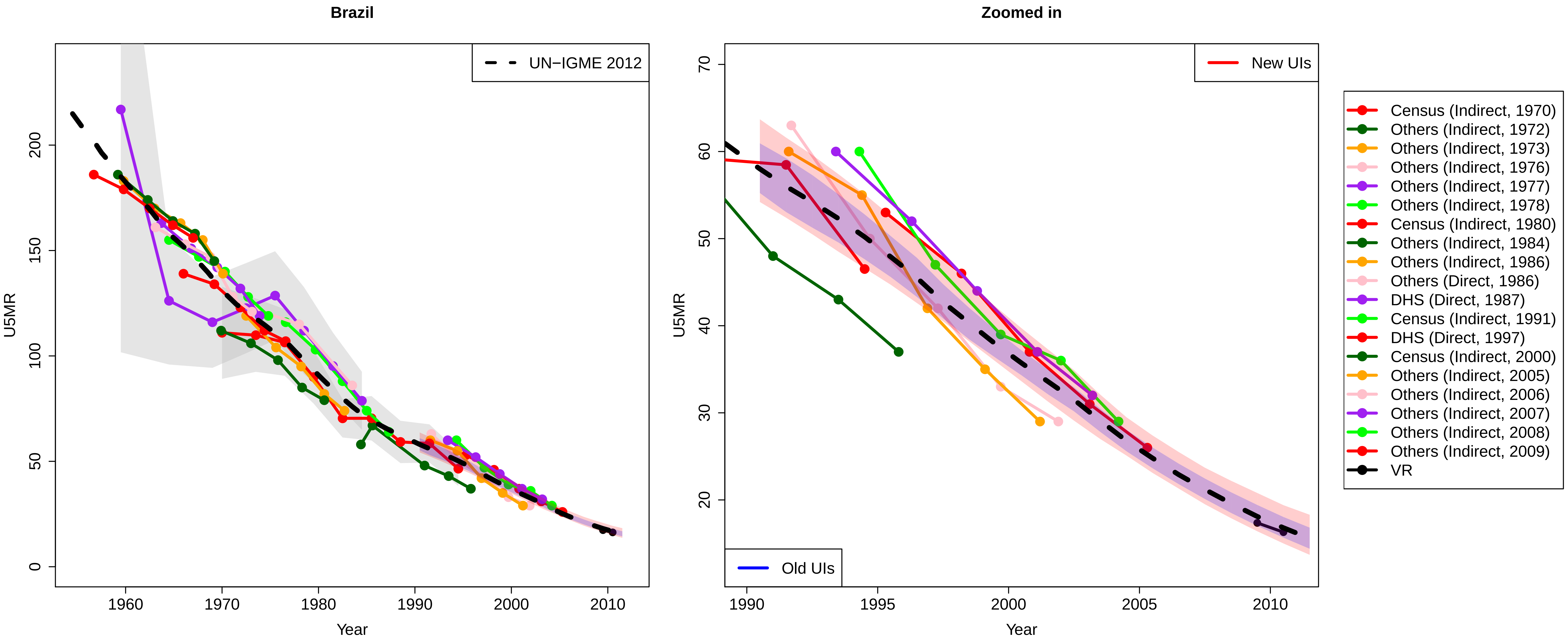

Brazil
UN−IGME 2012
U5MR
Year
Zoomed in
New UIs
Old UIs
Census (Indirect, 1970)
Others (Indirect, 1972)
Others (Indirect, 1973)
Others (Indirect, 1976)
Others (Indirect, 1977)
Others (Indirect, 1978)
Census (Indirect, 1980)
Others (Indirect, 1984)
Others (Indirect, 1986)
Others (Direct, 1986)
DHS (Direct, 1987)
Census (Indirect, 1991)
DHS (Direct, 1997)
Census (Indirect, 2000)
Others (Indirect, 2005)
Others (Indirect, 2006)
Others (Indirect, 2007)
Others (Indirect, 2008)
Others (Indirect, 2009)
VR

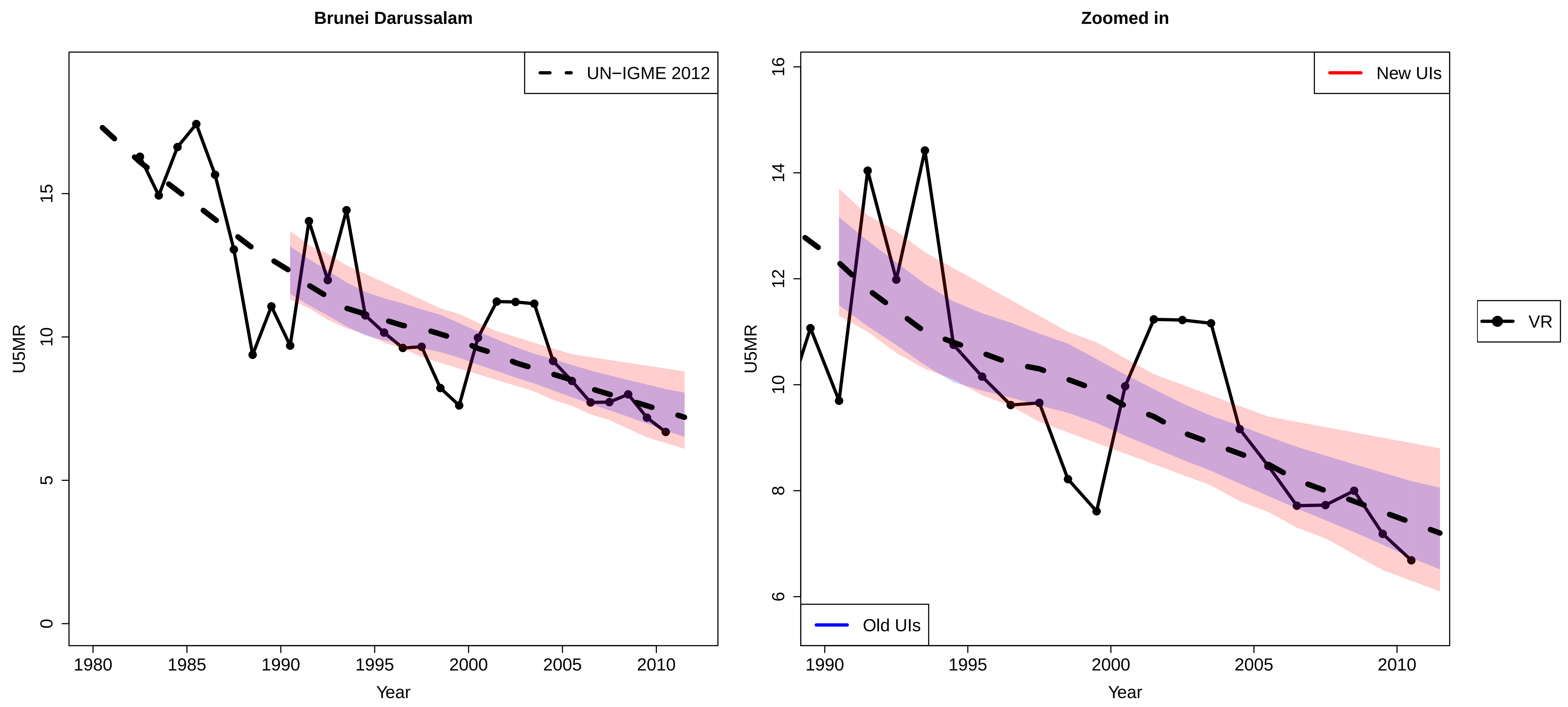

Brunei Darussalam
Zoomed in
UN−IGME 2012
New UIs
Old UIs
VR
U5MR
Year
0
5
10
15
6
8
10
12
14
16
1980
1985
1990
1995
2000
2005
2010

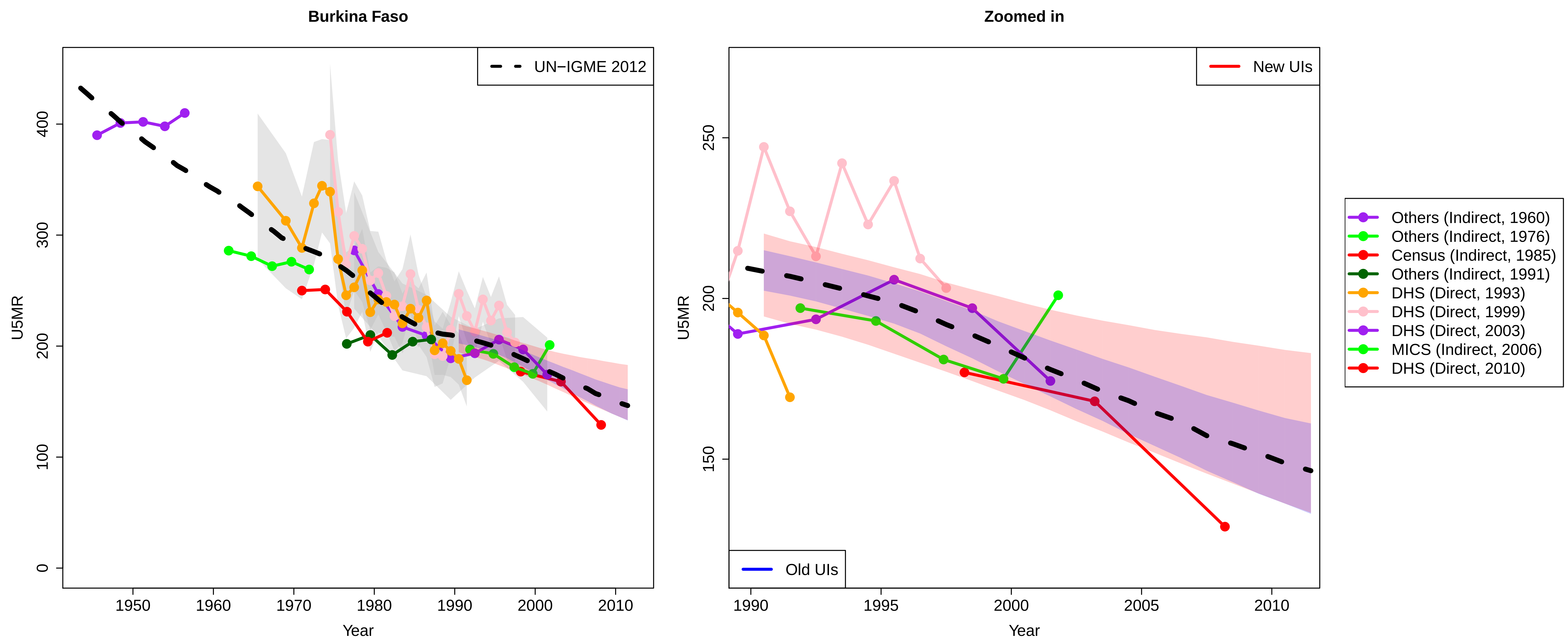

Burkina Faso
Zoomed in
UN−IGME 2012
New UIs
Old UIs
U5MR
Year
Others (Indirect, 1960)
Others (Indirect, 1976)
Census (Indirect, 1985)
Others (Indirect, 1991)
DHS (Direct, 1993)
DHS (Direct, 1999)
DHS (Direct, 2003)
MICS (Indirect, 2006)
DHS (Direct, 2010)

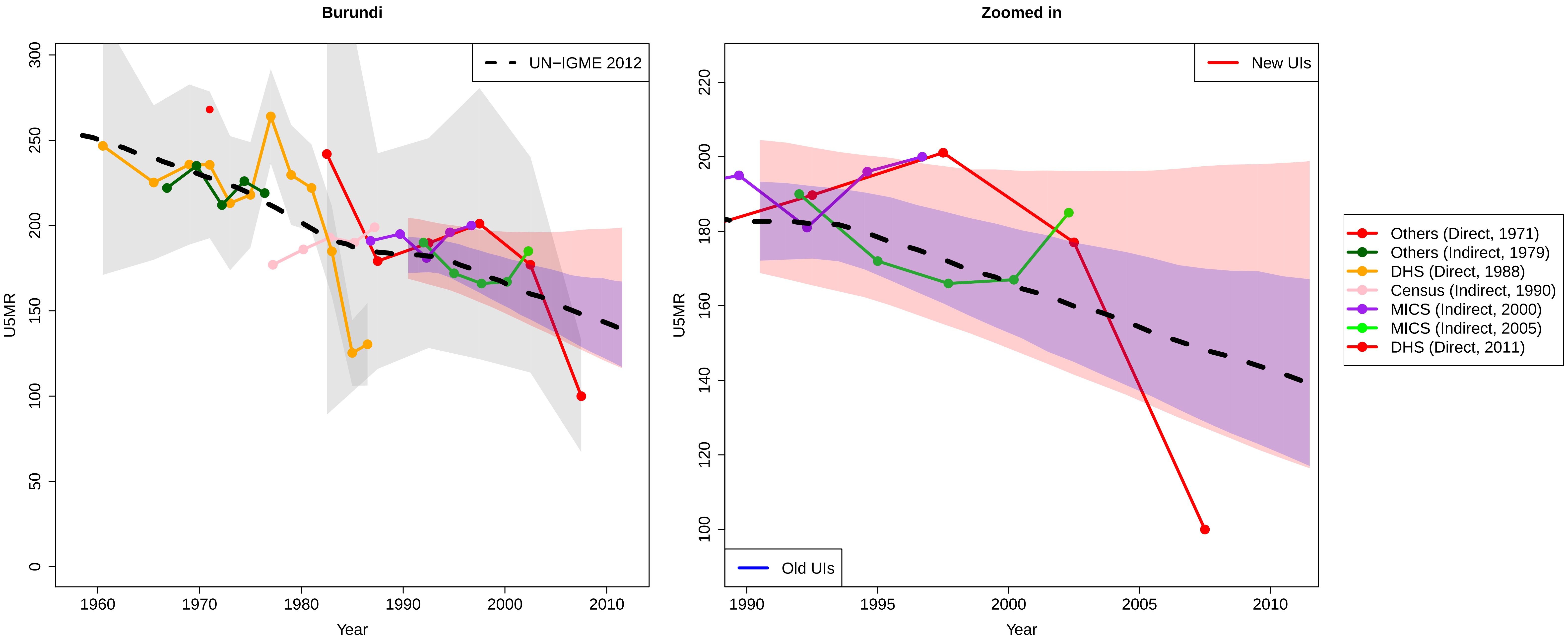

Burundi
Zoomed in
UN−IGME 2012
New UIs
Old UIs
U5MR
Year
Others (Direct, 1971)
Others (Indirect, 1979)
DHS (Direct, 1988)
Census (Indirect, 1990)
MICS (Indirect, 2000)
MICS (Indirect, 2005)
DHS (Direct, 2011)

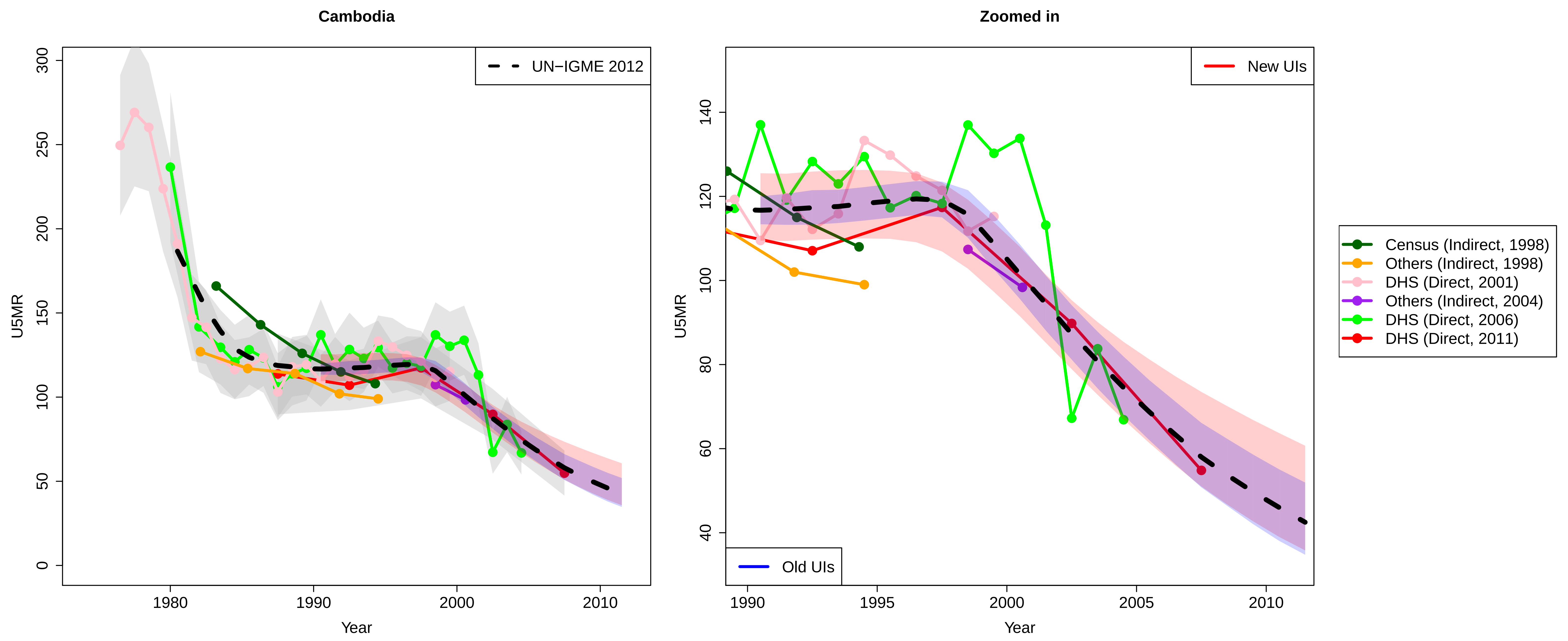

Cambodia
UN−IGME 2012
U5MR
Year
Zoomed in
New UIs
Old UIs
Census (Indirect, 1998)
Others (Indirect, 1998)
DHS (Direct, 2001)
Others (Indirect, 2004)
DHS (Direct, 2006)
DHS (Direct, 2011)

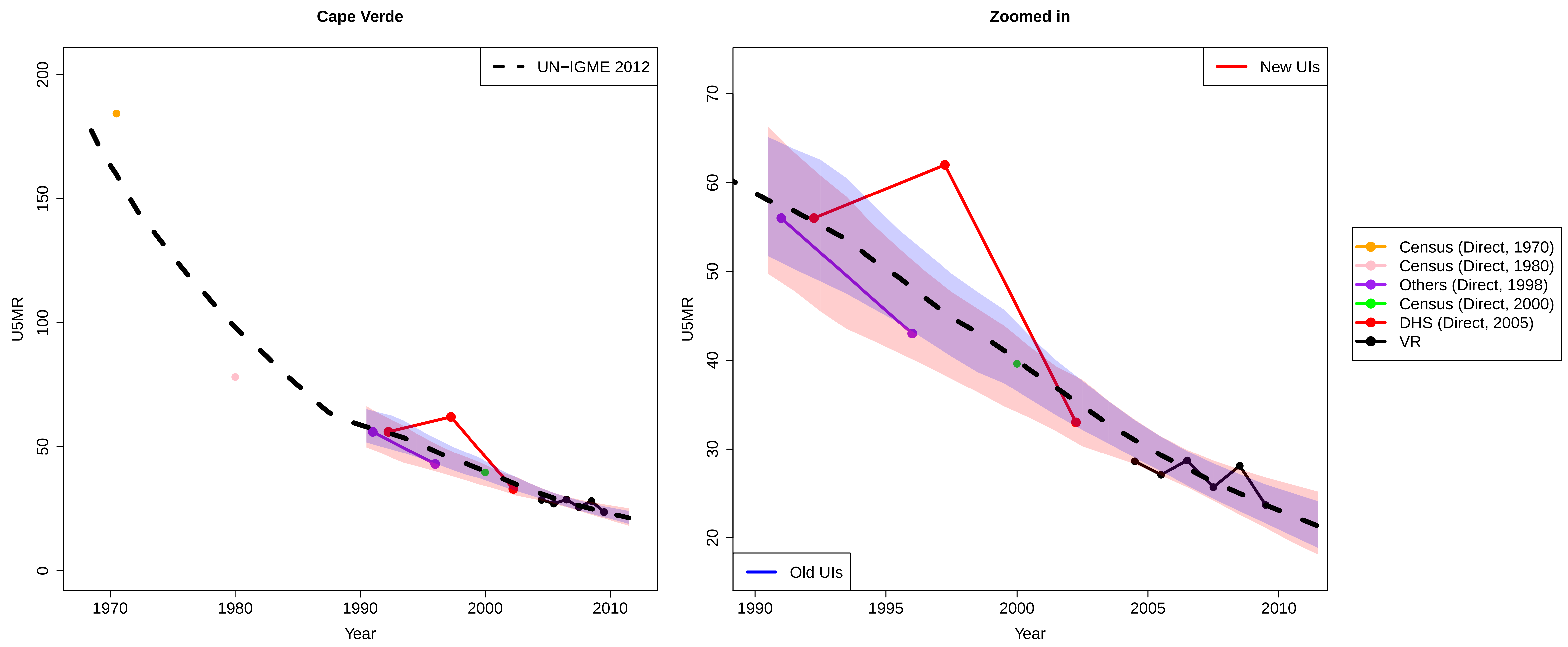
Cape Verde
Zoomed in
UN−IGME 2012
New UIs
Old UIs
Census (Direct, 1970)
Census (Direct, 1980)
Others (Direct, 1998)
Census (Direct, 2000)
DHS (Direct, 2005)
VR
U5MR
Year
0
50
100
150
200
20
30
40
50
60
70
1970
1980
1990
2000
2010
1995
2005

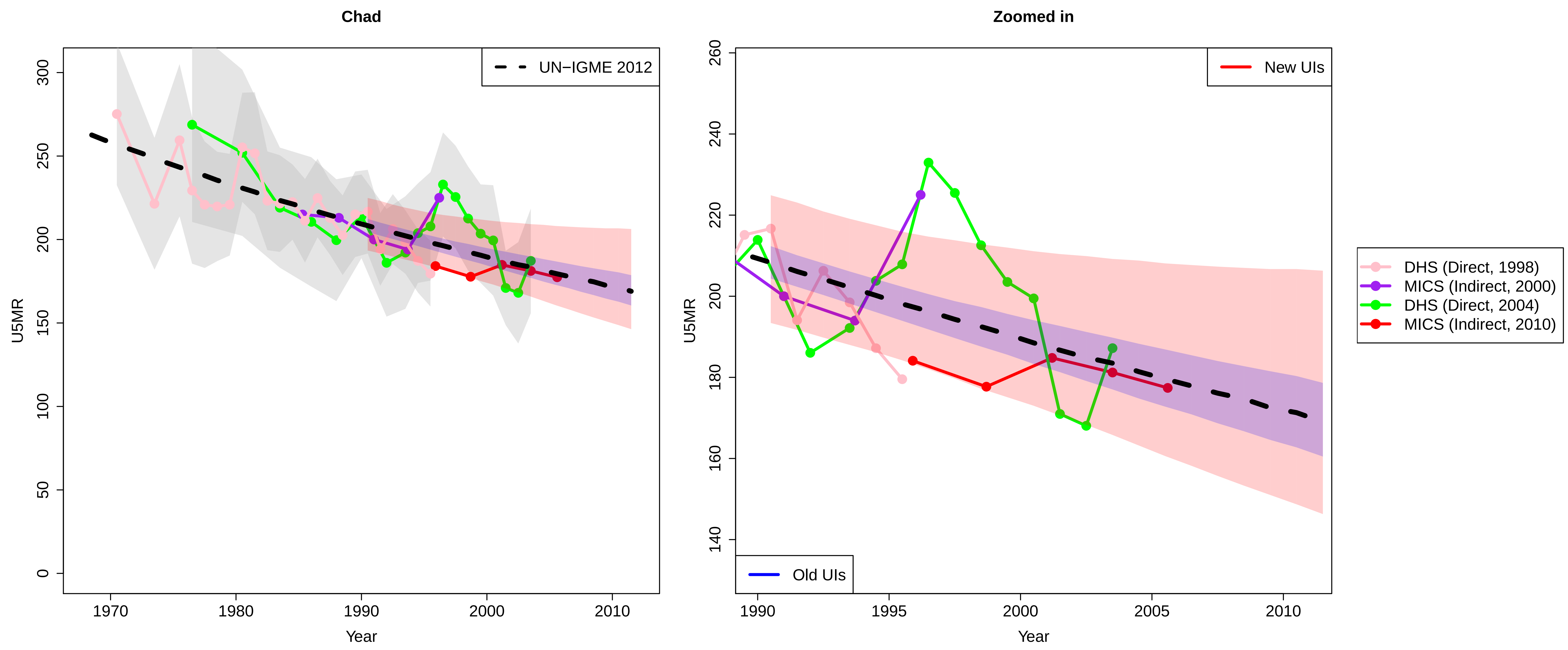

Chad
UN−IGME 2012
U5MR
Year
Zoomed in
New UIs
Old UIs
DHS (Direct, 1998)
MICS (Indirect, 2000)
DHS (Direct, 2004)
MICS (Indirect, 2010)

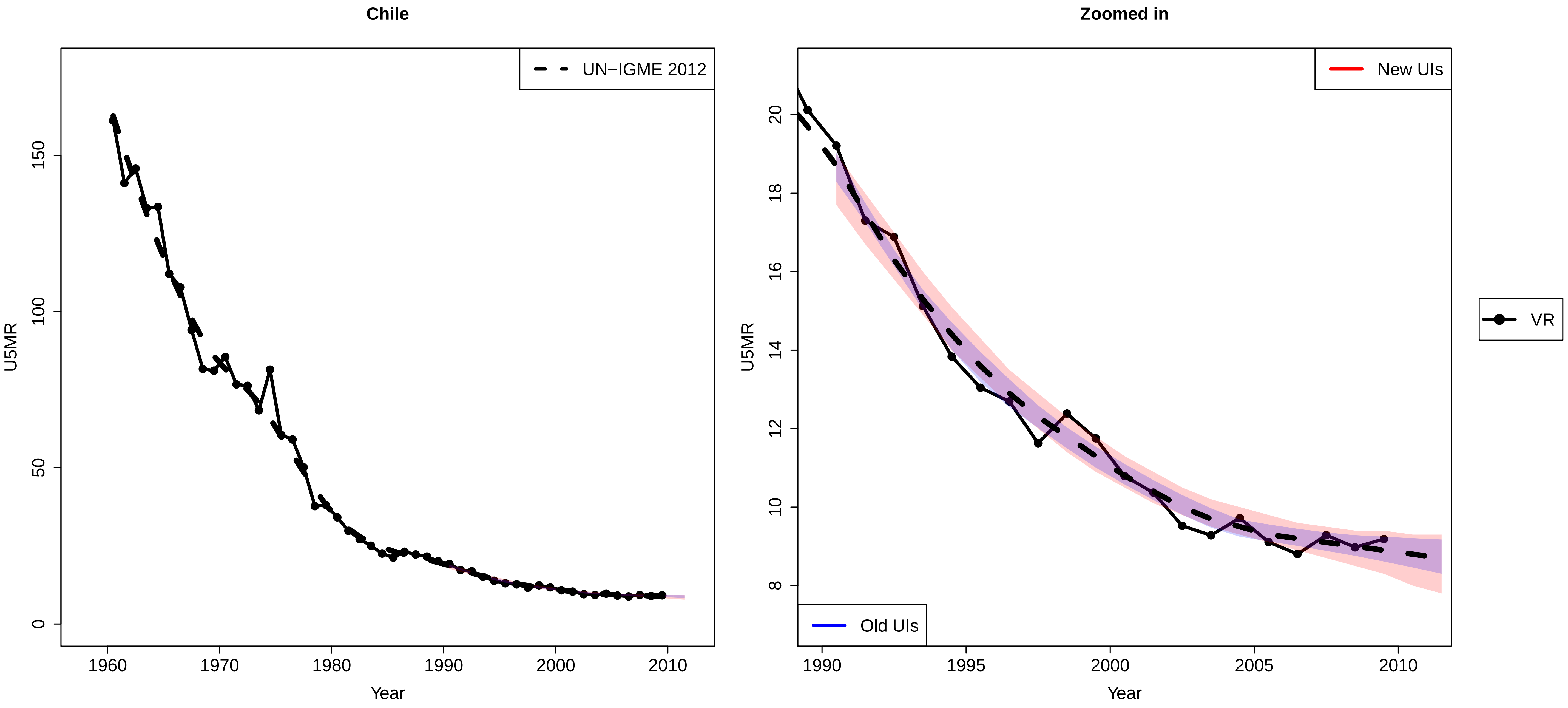

Chile
UN−IGME 2012
U5MR
Year
0
50
100
150
1960
1970
1980
1990
2000
2010
Zoomed in
New UIs
Old UIs
VR
8
10
12
14
16
18
20
1990
1995
2000
2005
2010

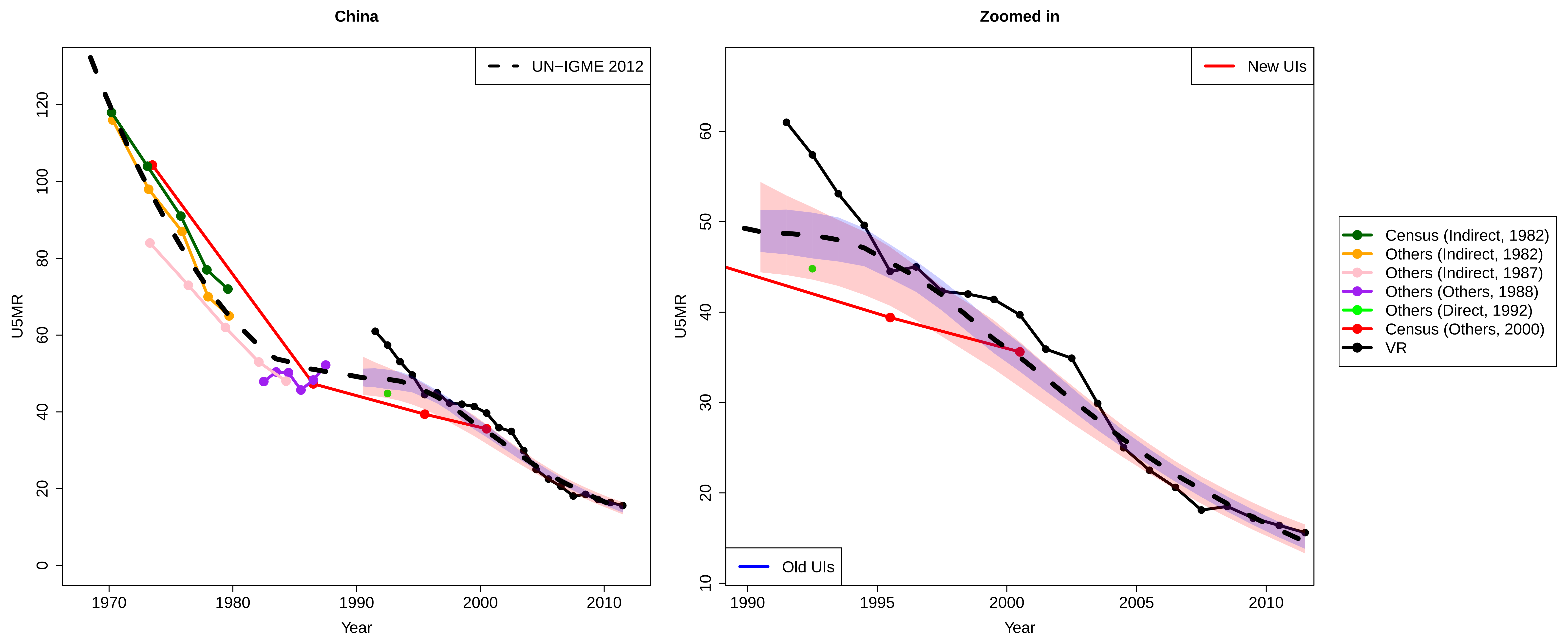
China
Zoomed in
UN−IGME 2012
New UIs
Old UIs
U5MR
Year
Census (Indirect, 1982)
Others (Indirect, 1982)
Others (Indirect, 1987)
Others (Others, 1988)
Others (Direct, 1992)
Census (Others, 2000)
VR

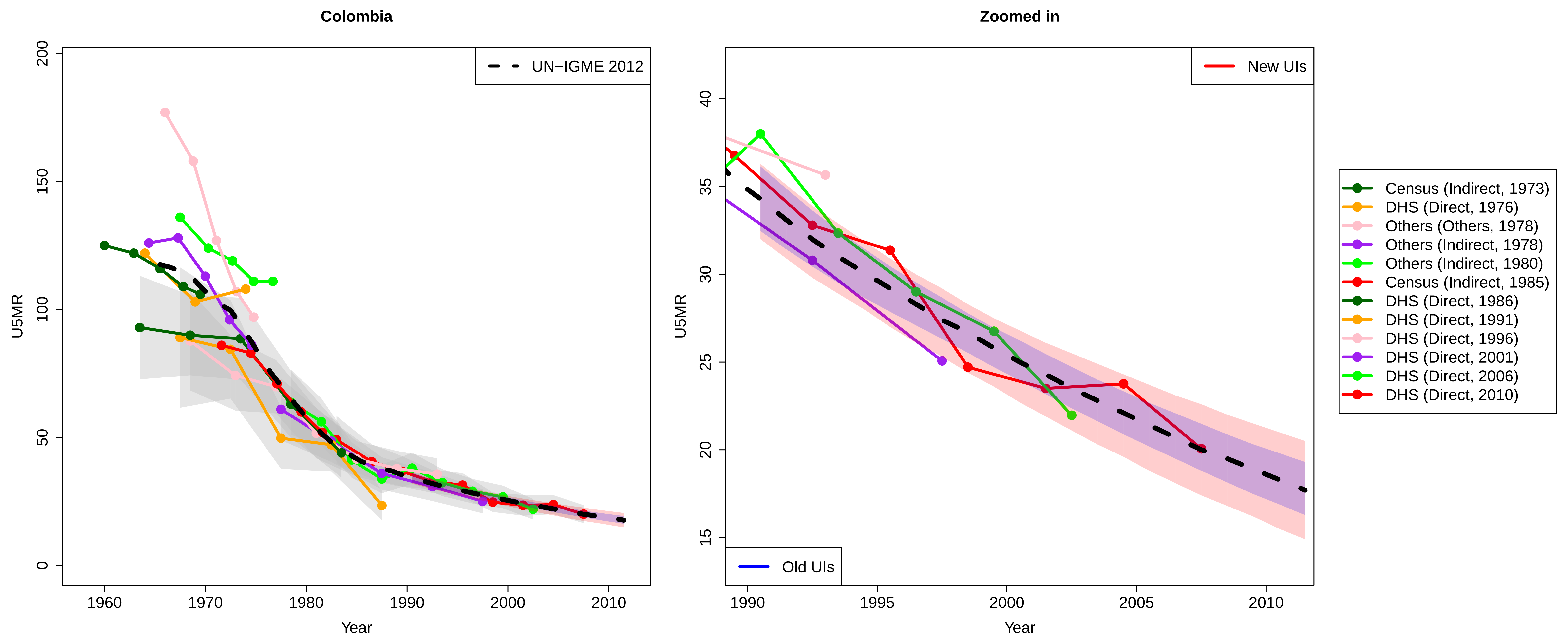

Colombia
UN−IGME 2012
U5MR
Year
Zoomed in
New UIs
Old UIs
Census (Indirect, 1973)
DHS (Direct, 1976)
Others (Others, 1978)
Others (Indirect, 1978)
Others (Indirect, 1980)
Census (Indirect, 1985)
DHS (Direct, 1986)
DHS (Direct, 1991)
DHS (Direct, 1996)
DHS (Direct, 2001)
DHS (Direct, 2006)
DHS (Direct, 2010)

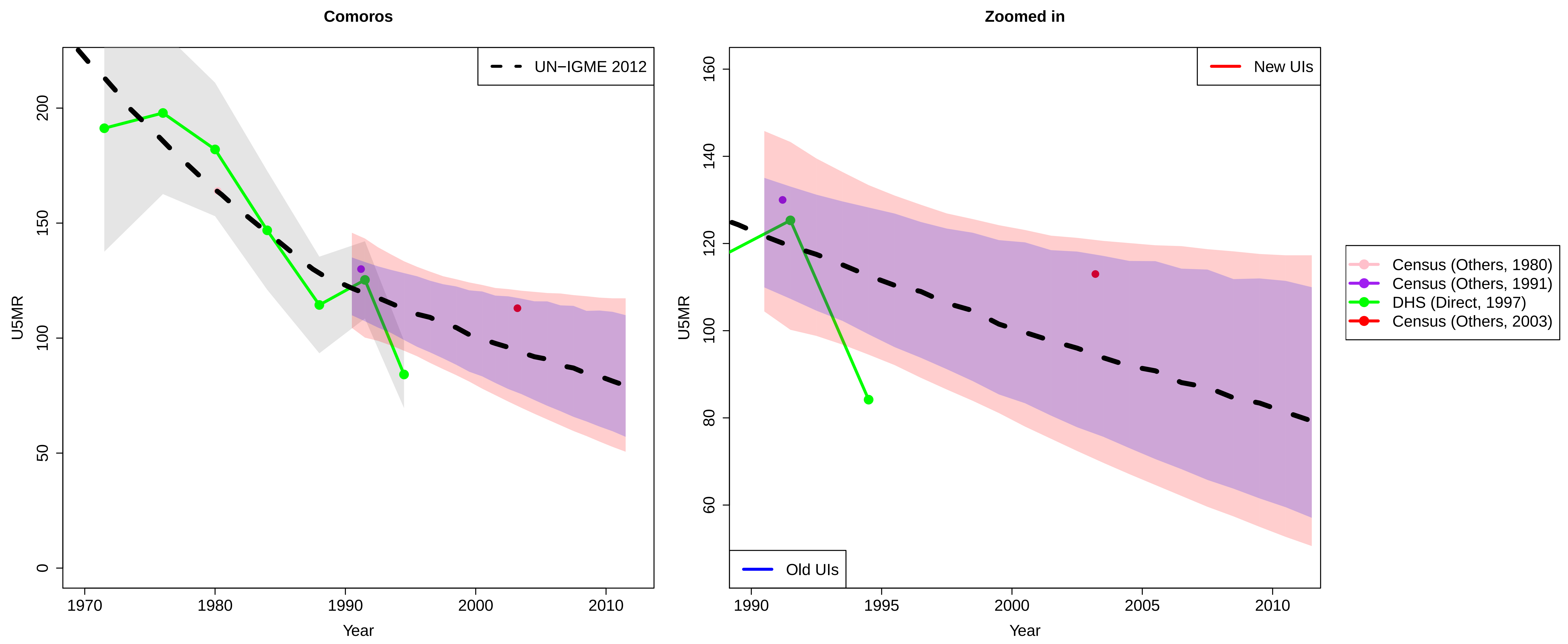
Comoros
UN−IGME 2012
U5MR
Year
Zoomed in
New UIs
Old UIs
Census (Others, 1980)
Census (Others, 1991)
DHS (Direct, 1997)
Census (Others, 2003)

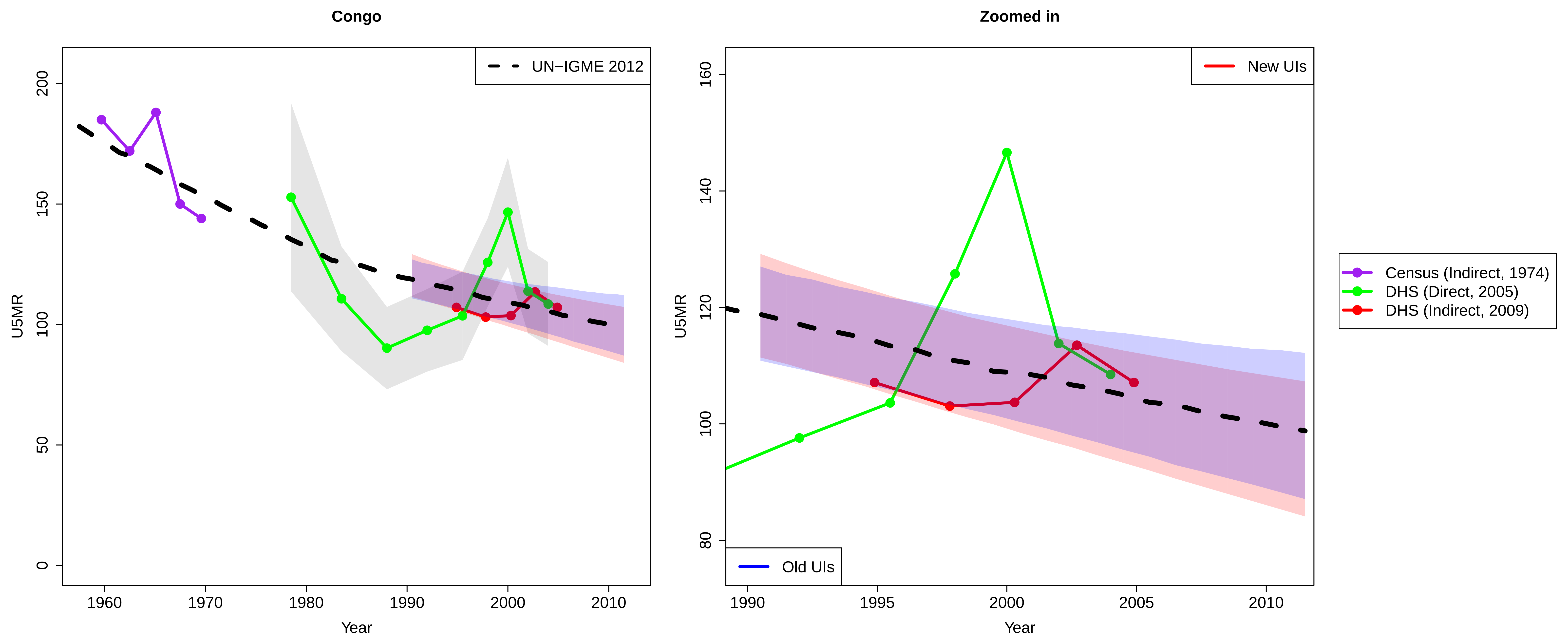
Congo
Zoomed in
UN−IGME 2012
New UIs
Old UIs
Census (Indirect, 1974)
DHS (Direct, 2005)
DHS (Indirect, 2009)
U5MR
Year
0
50
100
150
200
80
100
120
140
160
1960
1970
1980
1990
2000
2010
1990
1995
2000
2005
2010

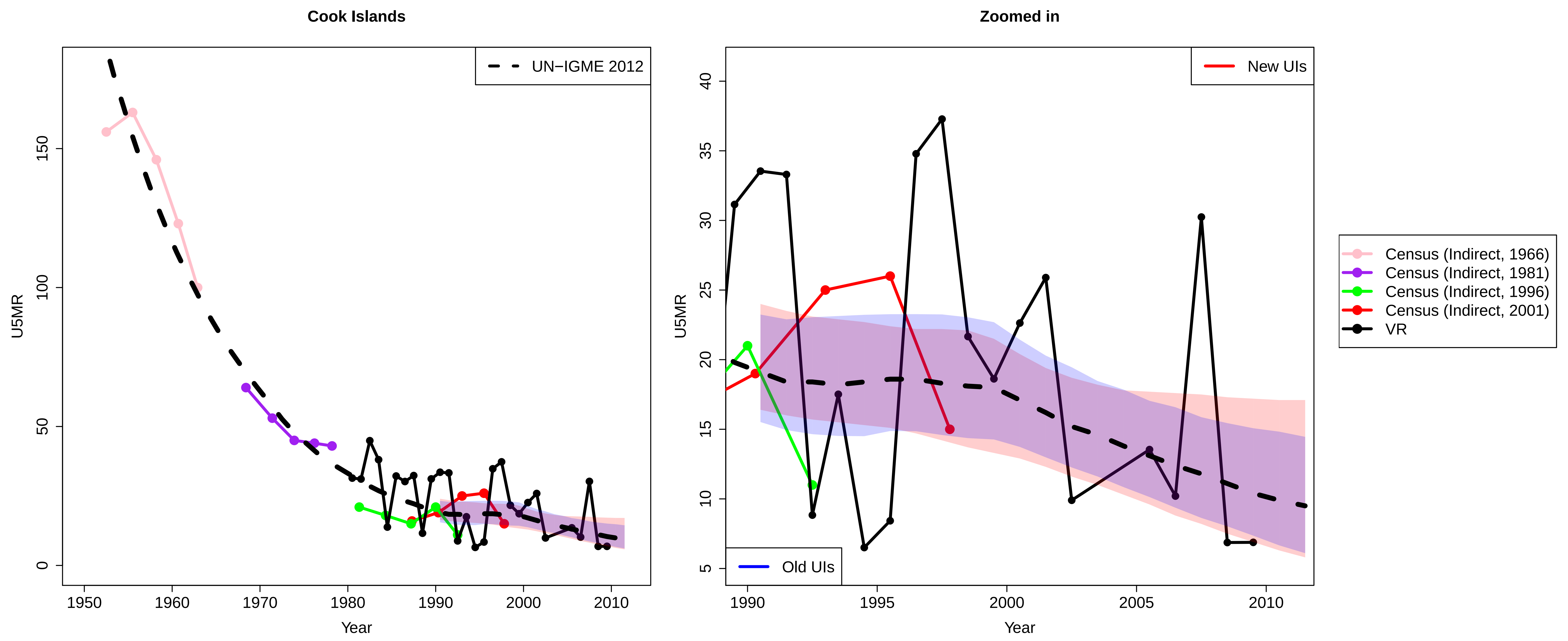
Cook Islands
Zoomed in
UN−IGME 2012
New UIs
Old UIs
Census (Indirect, 1966)
Census (Indirect, 1981)
Census (Indirect, 1996)
Census (Indirect, 2001)
VR
U5MR
Year
0
50
100
150
5
10
15
20
25
30
35
40
1950
1960
1970
1980
1990
2000
2010
1995
2005

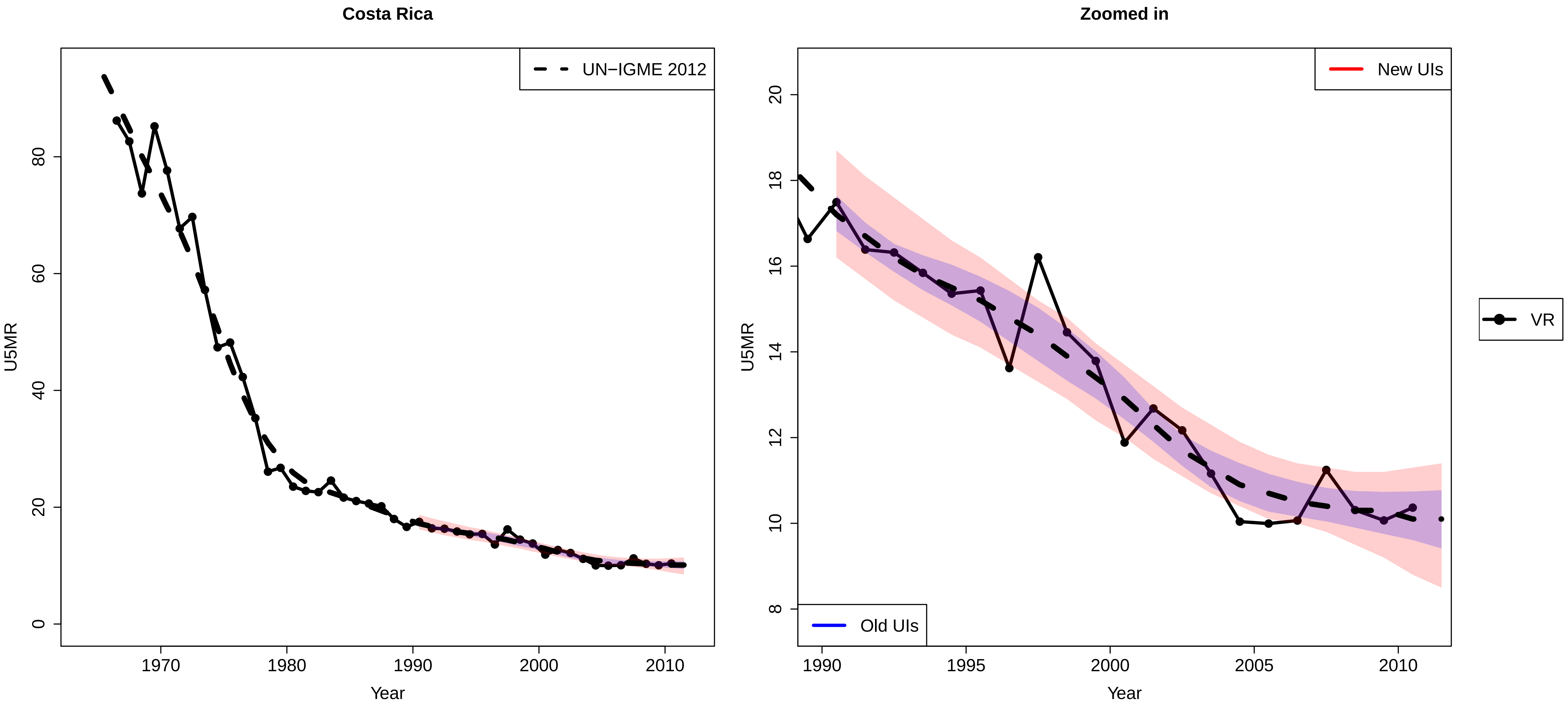

Costa Rica
UN−IGME 2012
U5MR
0
20
40
60
80
1970
1980
1990
2000
2010
Year
Zoomed in
New UIs
Old UIs
U5MR
8
10
12
14
16
18
20
1990
1995
2000
2005
2010
Year
VR

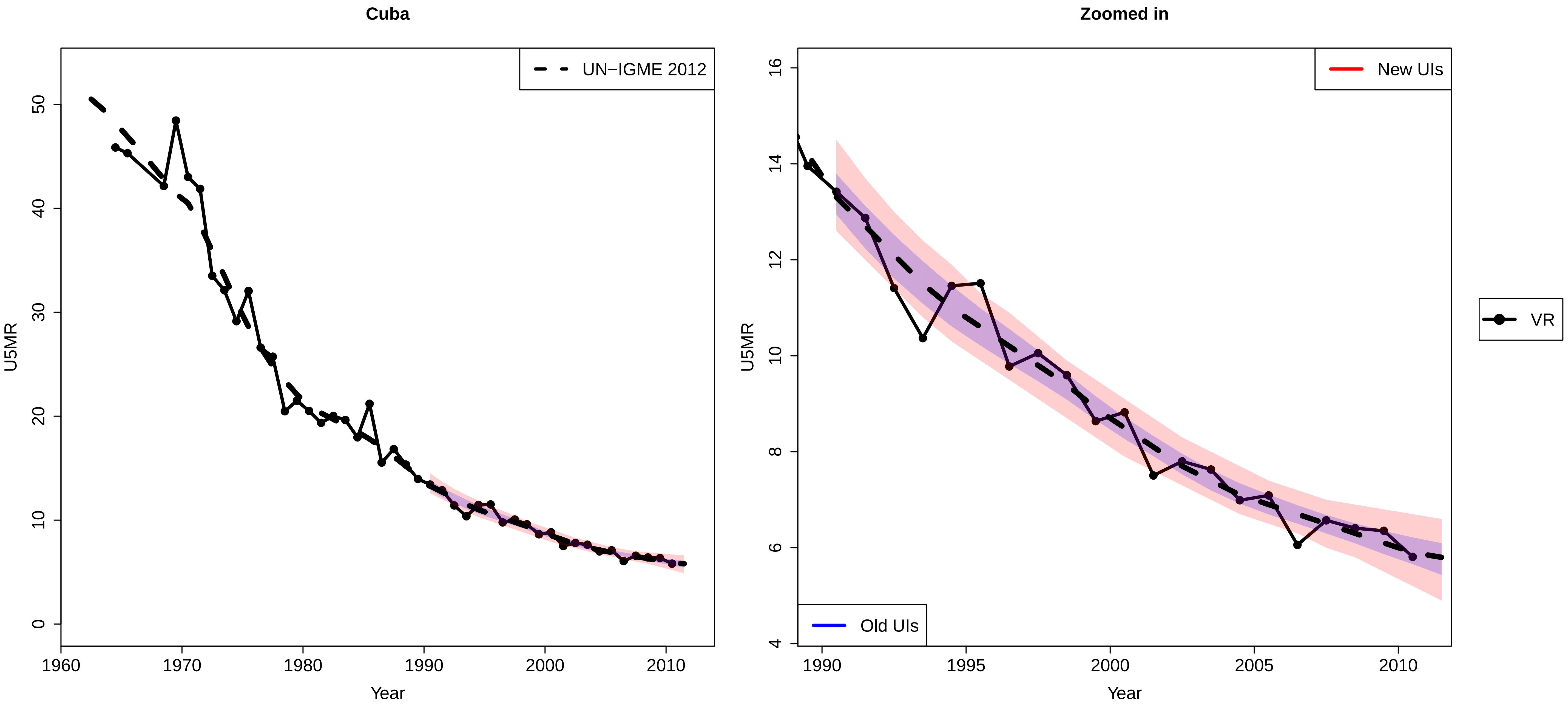
Cuba
UN−IGME 2012
U5MR
Year
Zoomed in
New UIs
Old UIs
VR
0
10
20
30
40
50
1960
1970
1980
1990
2000
2010
4
6
8
10
12
14
16
1995
2005

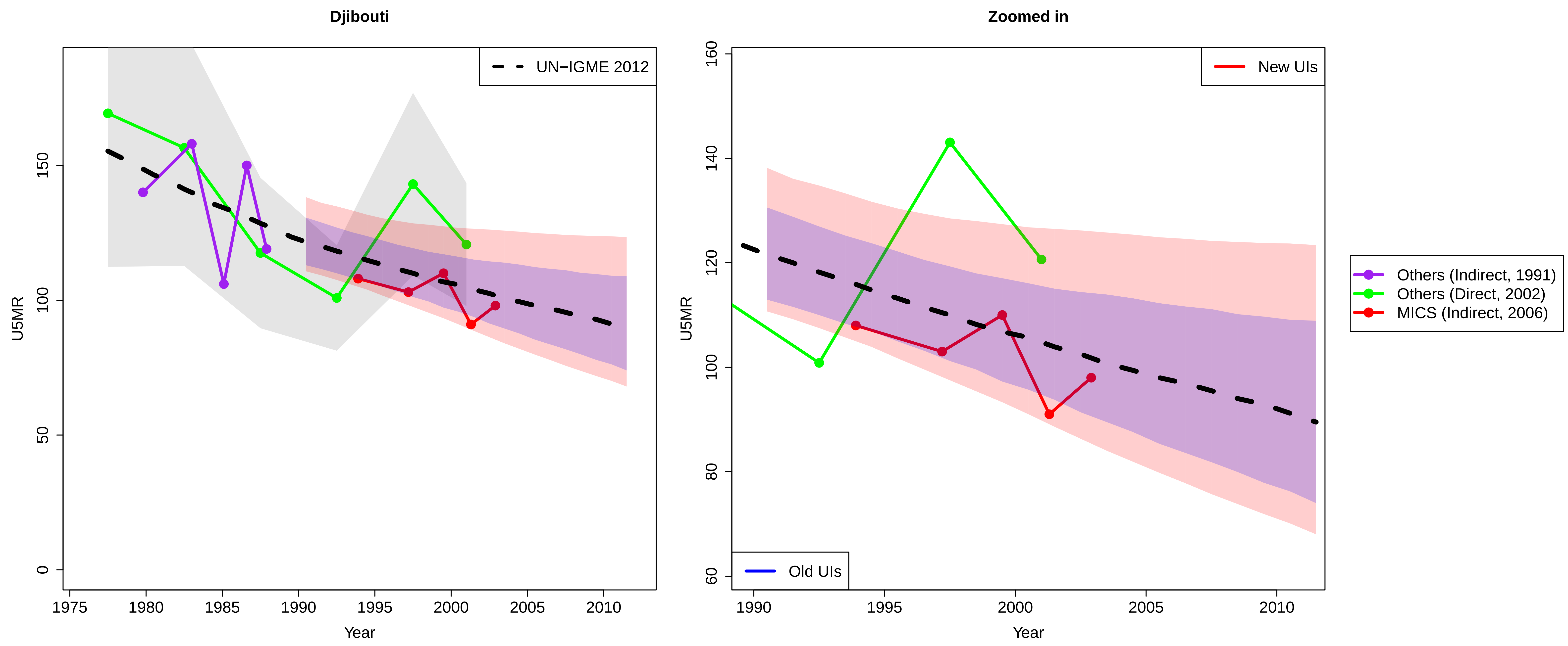

Djibouti
UN−IGME 2012
U5MR
0
50
100
150
1975
1980
1985
1990
1995
2000
2005
2010
Year
Zoomed in
New UIs
Old UIs
60
80
100
120
140
160
Others (Indirect, 1991)
Others (Direct, 2002)
MICS (Indirect, 2006)

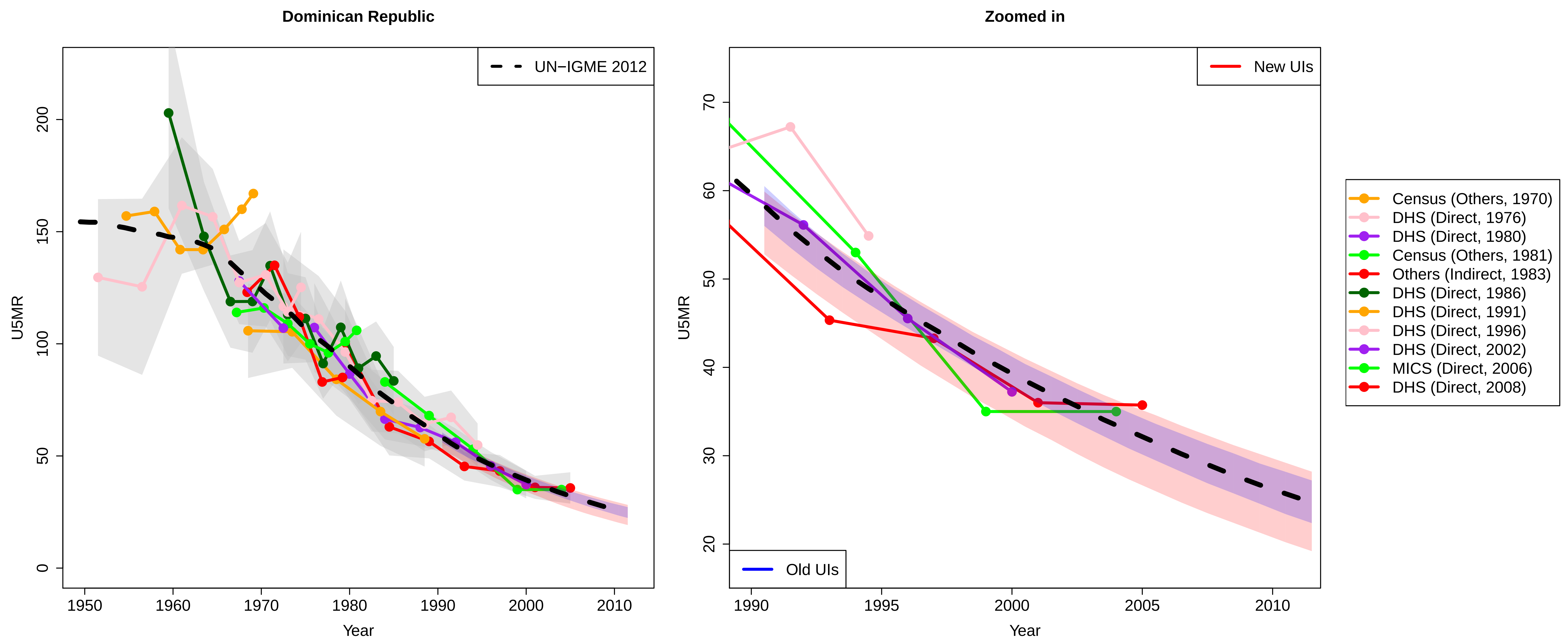
Dominican Republic
Zoomed in
UN−IGME 2012
New UIs
Old UIs
U5MR
Year
Census (Others, 1970)
DHS (Direct, 1976)
DHS (Direct, 1980)
Census (Others, 1981)
Others (Indirect, 1983)
DHS (Direct, 1986)
DHS (Direct, 1991)
DHS (Direct, 1996)
DHS (Direct, 2002)
MICS (Direct, 2006)
DHS (Direct, 2008)

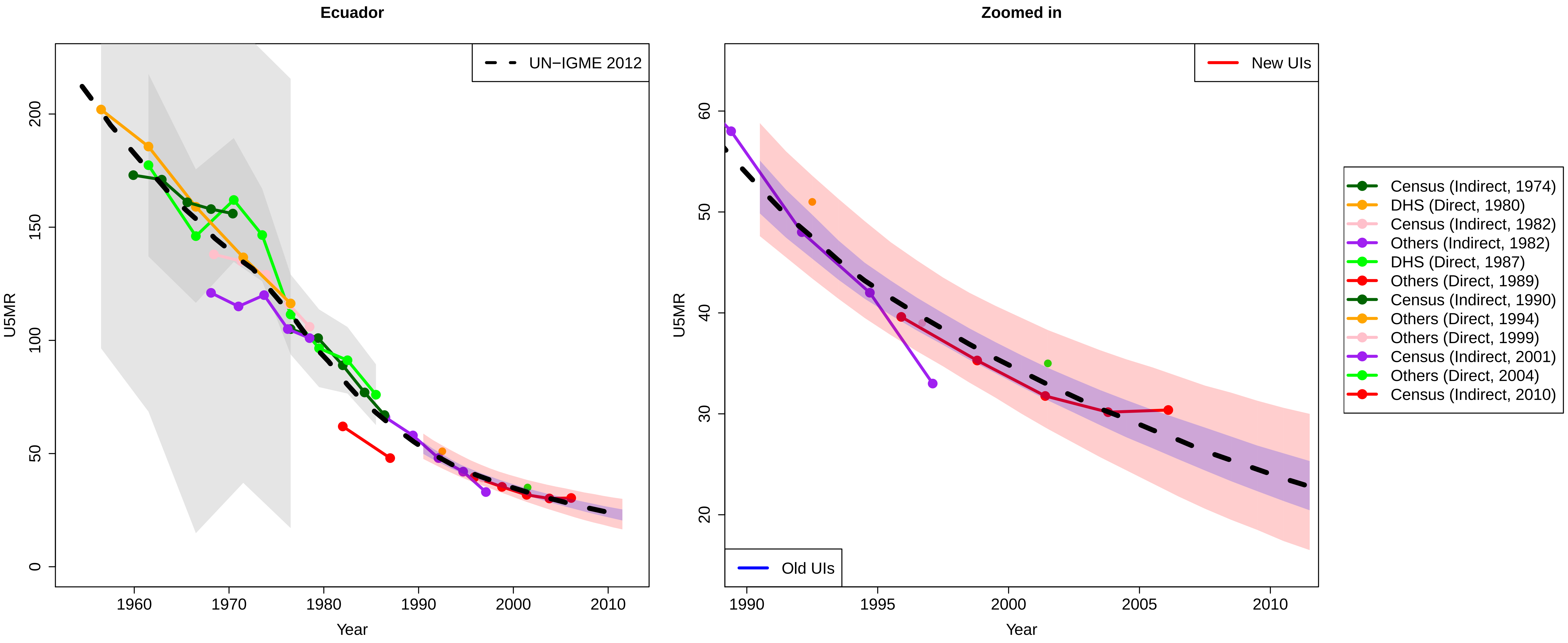

Ecuador
Zoomed in
UN−IGME 2012
New UIs
Old UIs
U5MR
Year
Census (Indirect, 1974)
DHS (Direct, 1980)
Census (Indirect, 1982)
Others (Indirect, 1982)
DHS (Direct, 1987)
Others (Direct, 1989)
Census (Indirect, 1990)
Others (Direct, 1994)
Others (Direct, 1999)
Census (Indirect, 2001)
Others (Direct, 2004)
Census (Indirect, 2010)

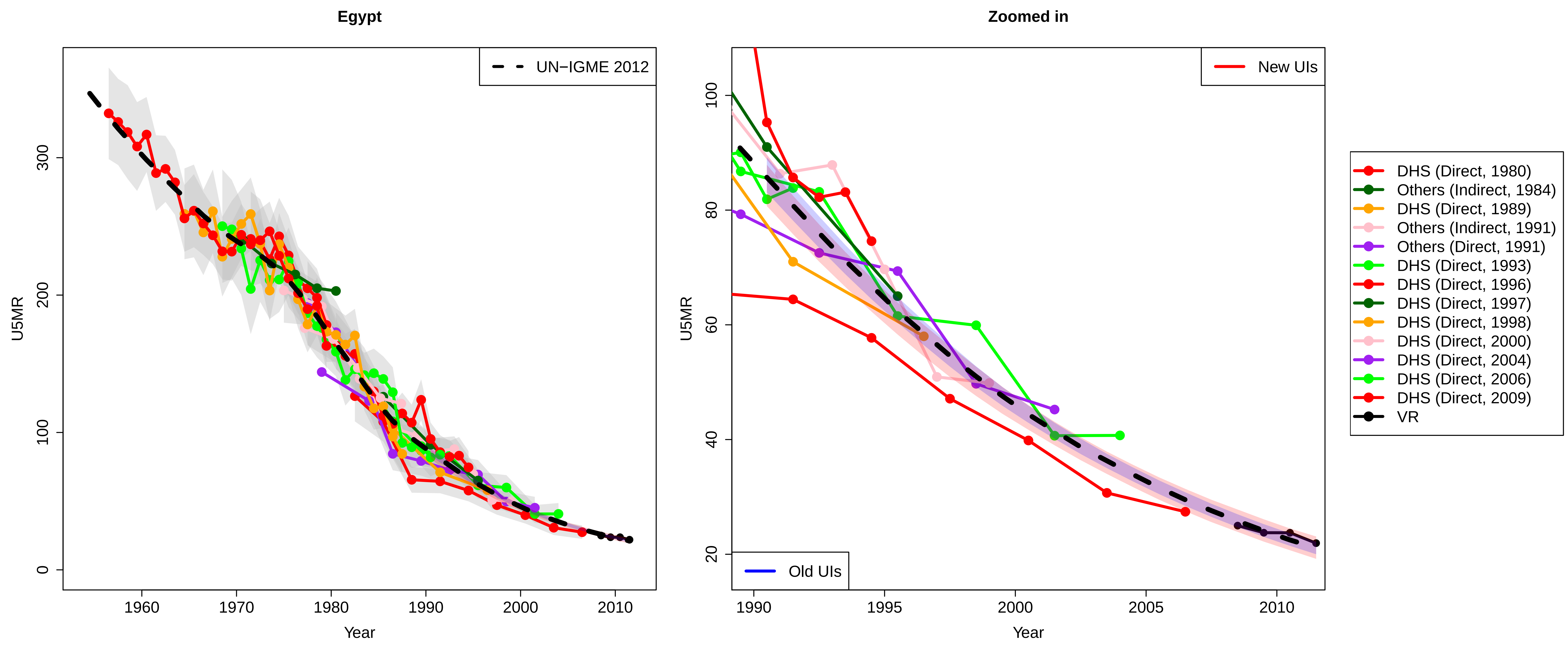

Egypt
UN−IGME 2012
U5MR
Year
Zoomed in
New UIs
Old UIs
DHS (Direct, 1980)
Others (Indirect, 1984)
DHS (Direct, 1989)
Others (Indirect, 1991)
Others (Direct, 1991)
DHS (Direct, 1993)
DHS (Direct, 1996)
DHS (Direct, 1997)
DHS (Direct, 1998)
DHS (Direct, 2000)
DHS (Direct, 2004)
DHS (Direct, 2006)
DHS (Direct, 2009)
VR

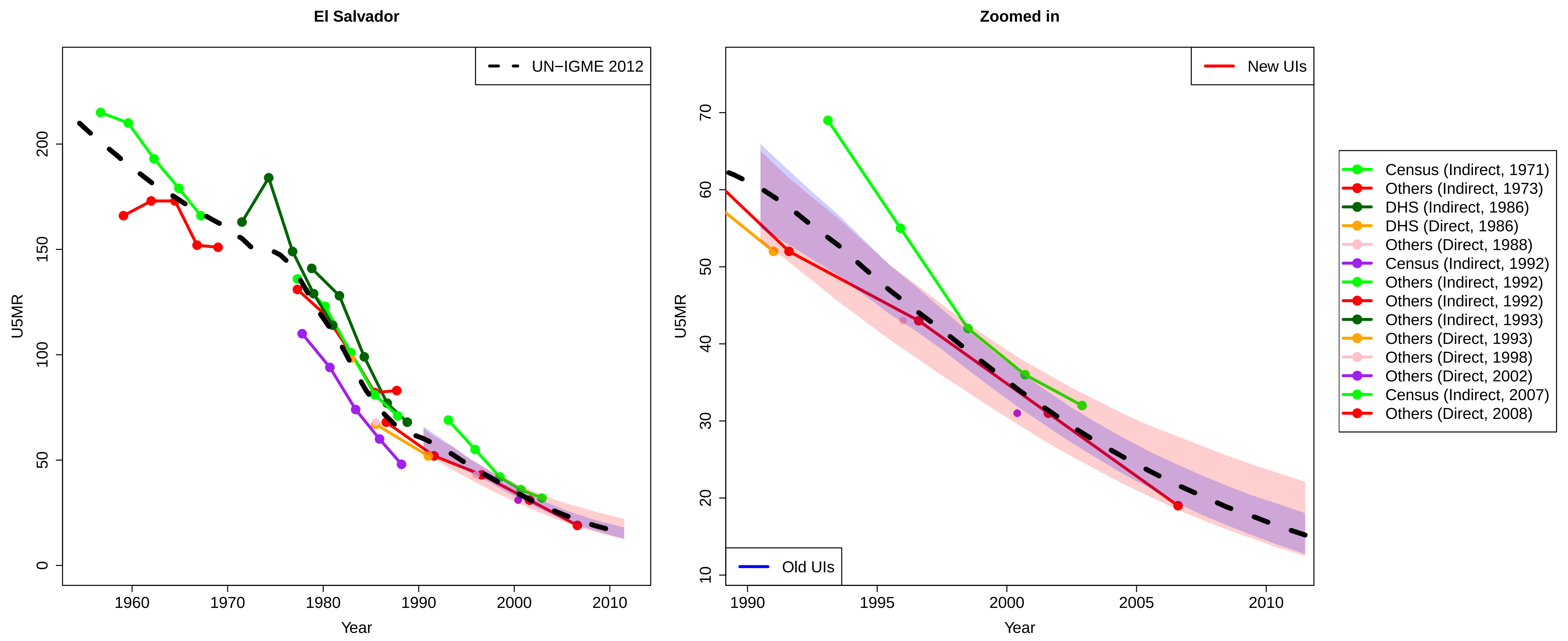
El Salvador
Zoomed in
UN−IGME 2012
New UIs
Old UIs
U5MR
Year
Census (Indirect, 1971)
Others (Indirect, 1973)
DHS (Indirect, 1986)
DHS (Direct, 1986)
Others (Direct, 1988)
Census (Indirect, 1992)
Others (Indirect, 1992)
Others (Indirect, 1992)
Others (Indirect, 1993)
Others (Direct, 1993)
Others (Direct, 1998)
Others (Direct, 2002)
Census (Indirect, 2007)
Others (Direct, 2008)

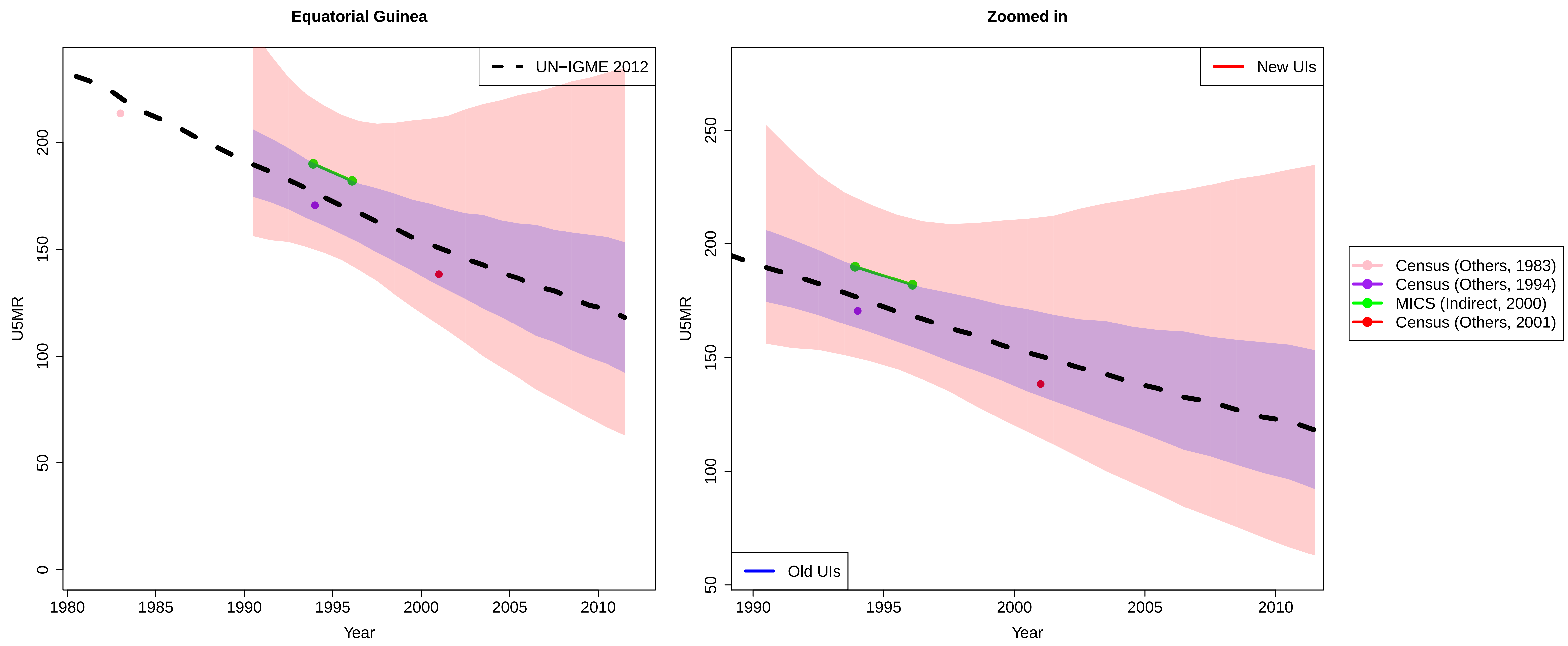

Equatorial Guinea
Zoomed in
UN−IGME 2012
New UIs
Old UIs
Census (Others, 1983)
Census (Others, 1994)
MICS (Indirect, 2000)
Census (Others, 2001)
U5MR
Year

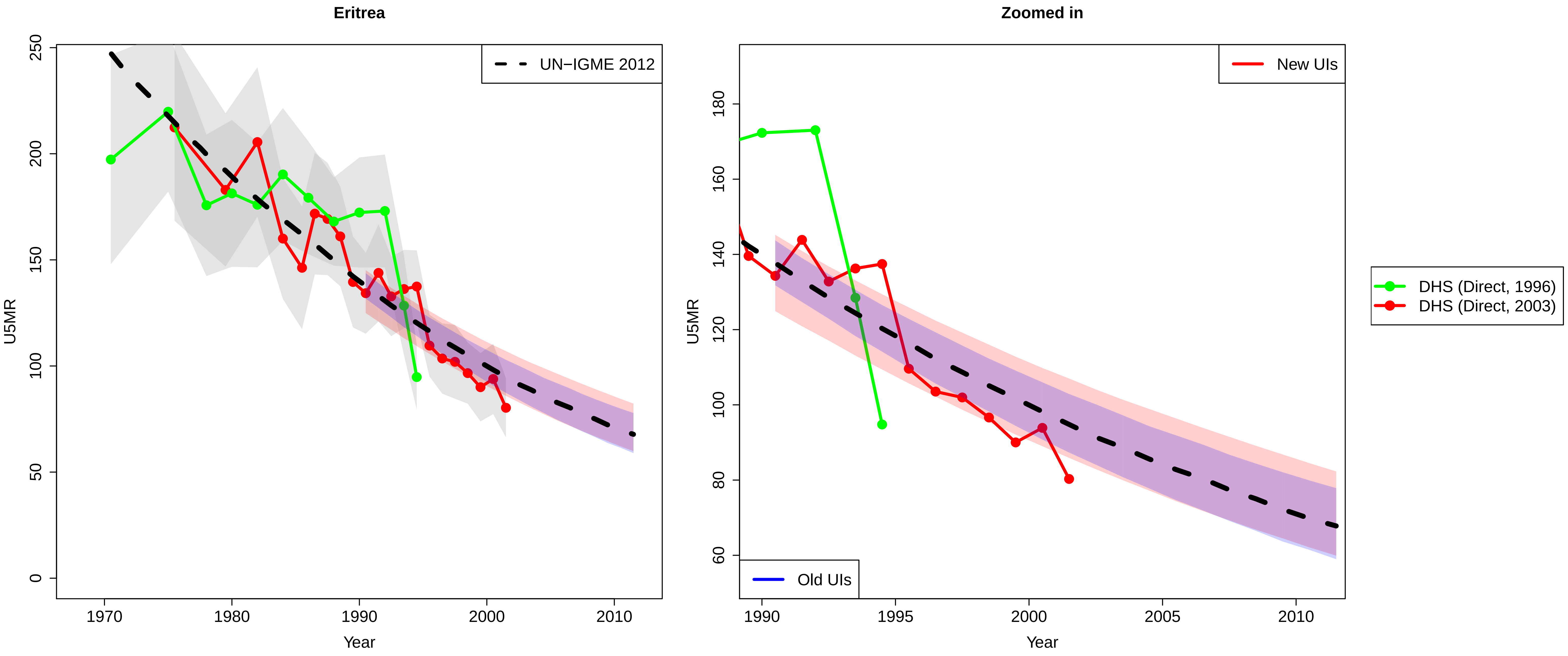

Eritrea
UN−IGME 2012
U5MR
Year
Zoomed in
New UIs
Old UIs
DHS (Direct, 1996)
DHS (Direct, 2003)

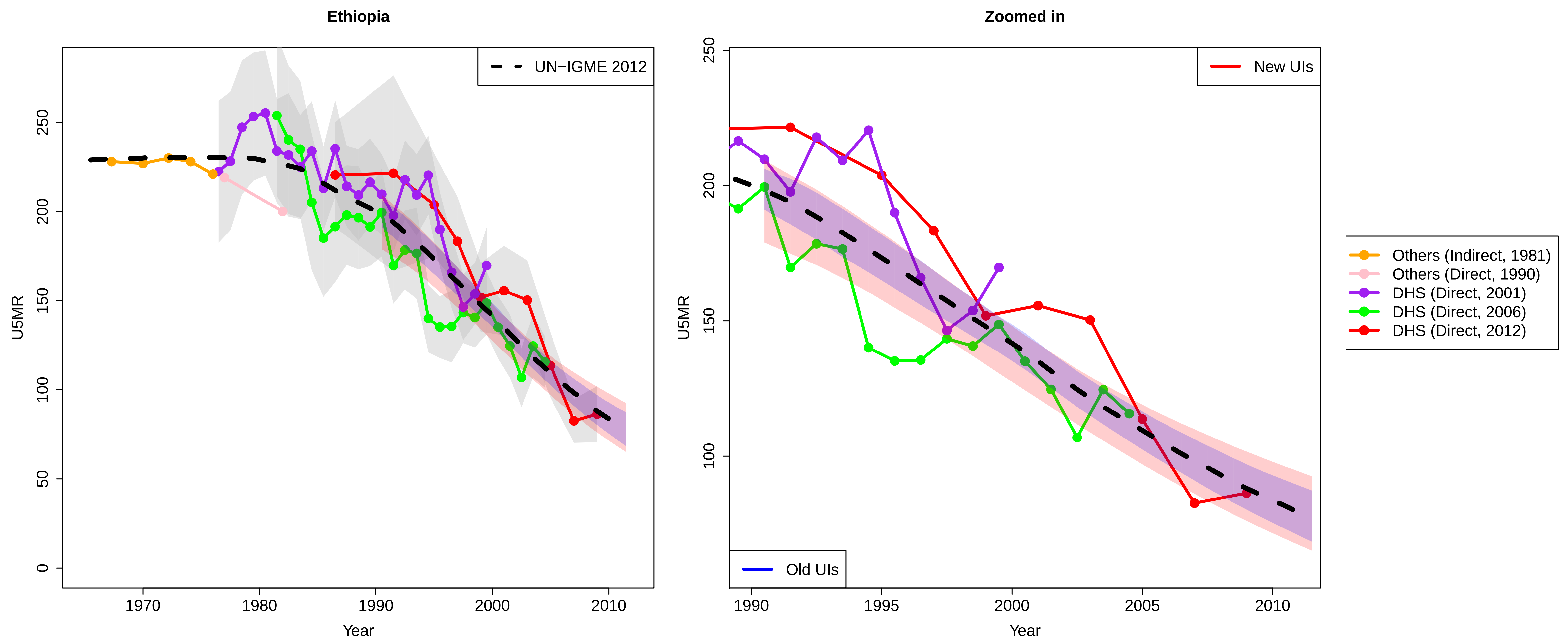

Ethiopia
UN−IGME 2012
U5MR
0
50
100
150
200
250
1970
1980
1990
2000
2010
Year
Zoomed in
New UIs
Old UIs
U5MR
100
150
200
250
1990
1995
2000
2005
2010
Year
Others (Indirect, 1981)
Others (Direct, 1990)
DHS (Direct, 2001)
DHS (Direct, 2006)
DHS (Direct, 2012)

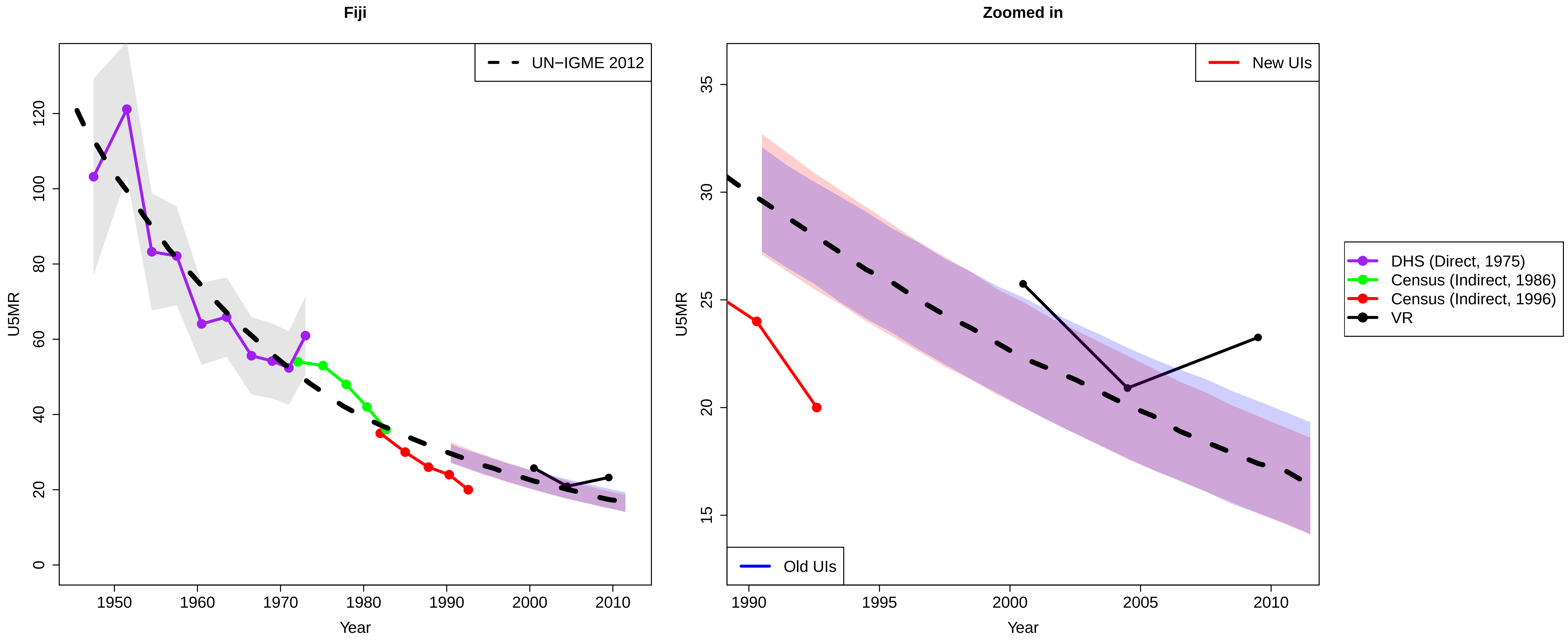

Fiji
Zoomed in
UN−IGME 2012
New UIs
Old UIs
DHS (Direct, 1975)
Census (Indirect, 1986)
Census (Indirect, 1996)
VR
U5MR
Year
0
20
40
60
80
100
120
1950
1960
1970
1980
1990
2000
2010
15
20
25
30
35
1995
2005

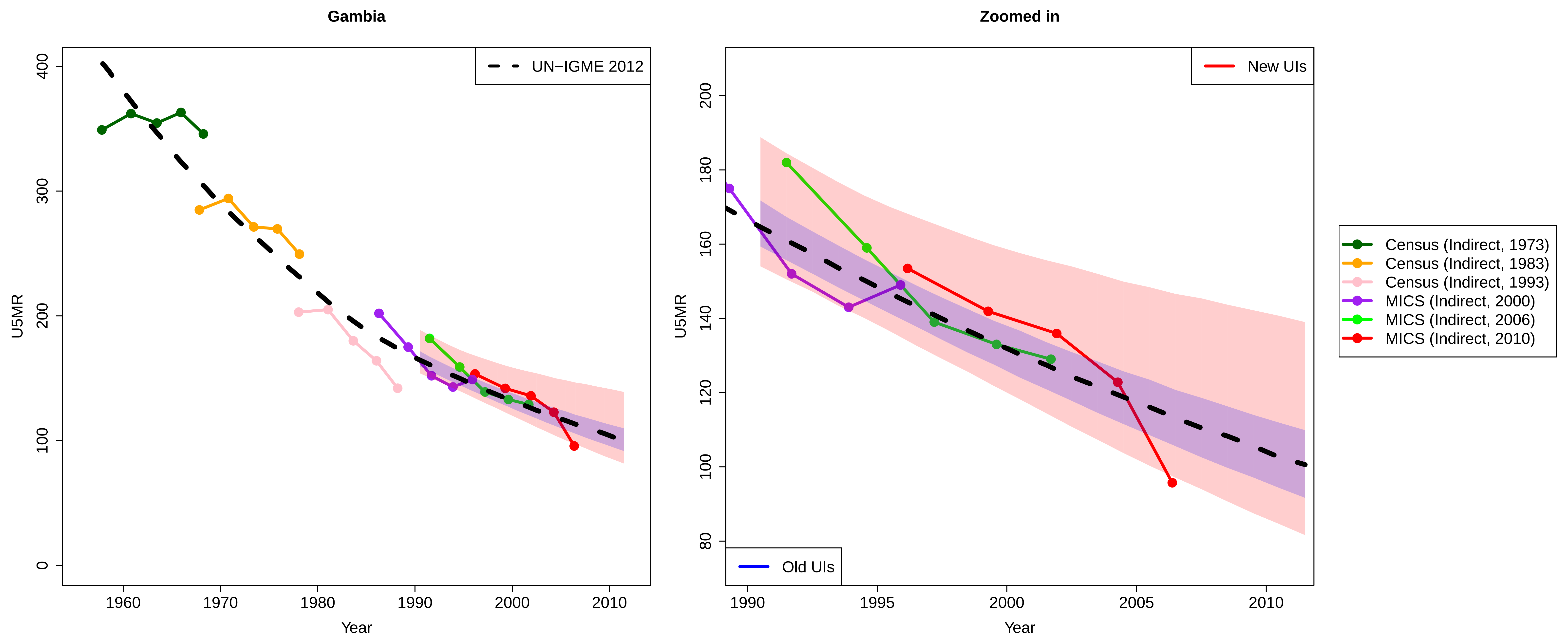

Gambia
UN−IGME 2012
U5MR
Year
Zoomed in
New UIs
Old UIs
Census (Indirect, 1973)
Census (Indirect, 1983)
Census (Indirect, 1993)
MICS (Indirect, 2000)
MICS (Indirect, 2006)
MICS (Indirect, 2010)

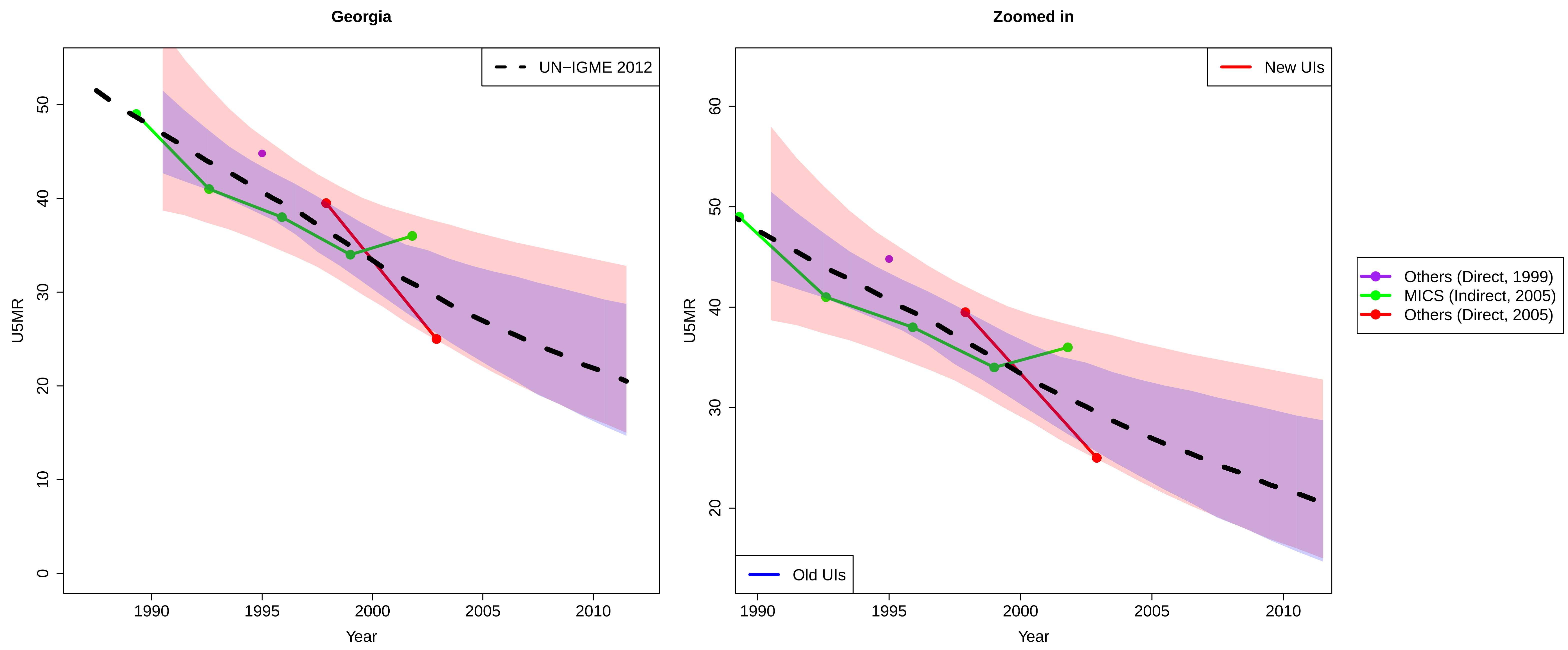
Georgia
UN−IGME 2012
U5MR
Year
Zoomed in
New UIs
Old UIs
Others (Direct, 1999)
MICS (Indirect, 2005)
Others (Direct, 2005)

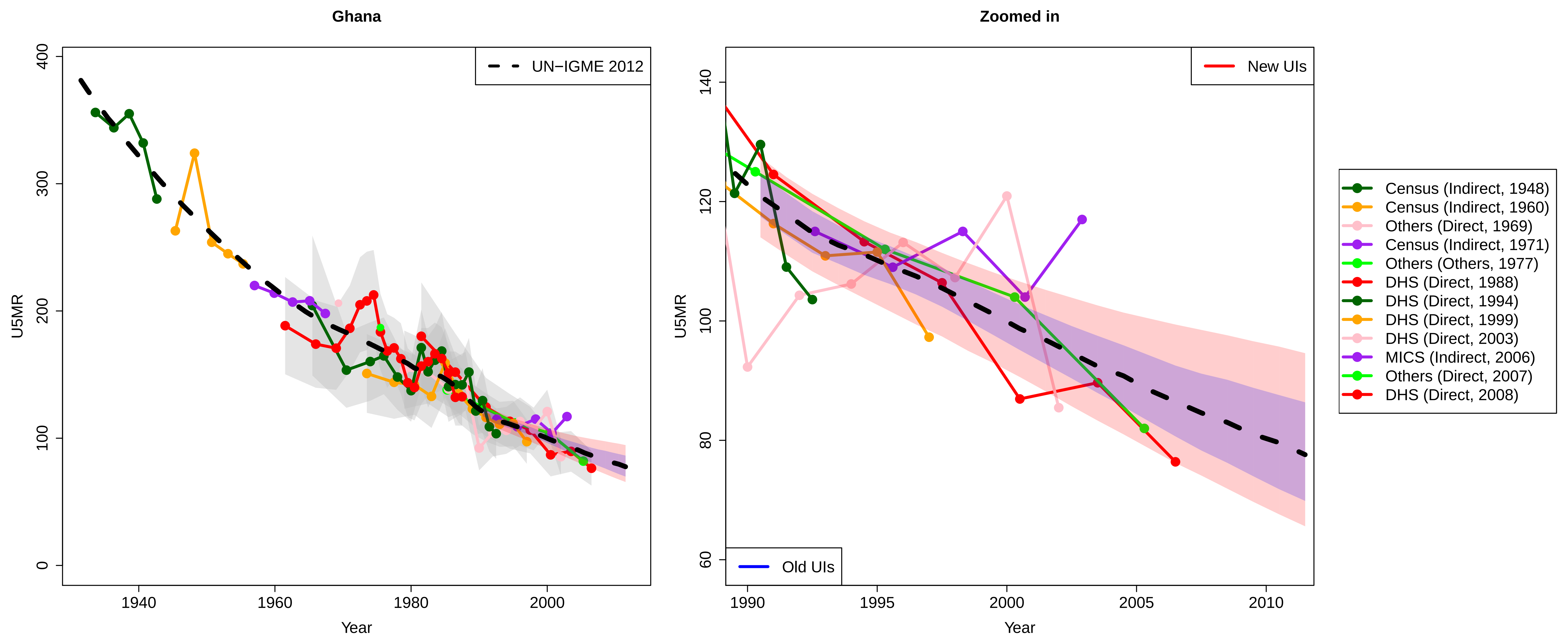

Ghana
Zoomed in
UN−IGME 2012
New UIs
Old UIs
U5MR
Year
Census (Indirect, 1948)
Census (Indirect, 1960)
Others (Direct, 1969)
Census (Indirect, 1971)
Others (Others, 1977)
DHS (Direct, 1988)
DHS (Direct, 1994)
DHS (Direct, 1999)
DHS (Direct, 2003)
MICS (Indirect, 2006)
Others (Direct, 2007)
DHS (Direct, 2008)

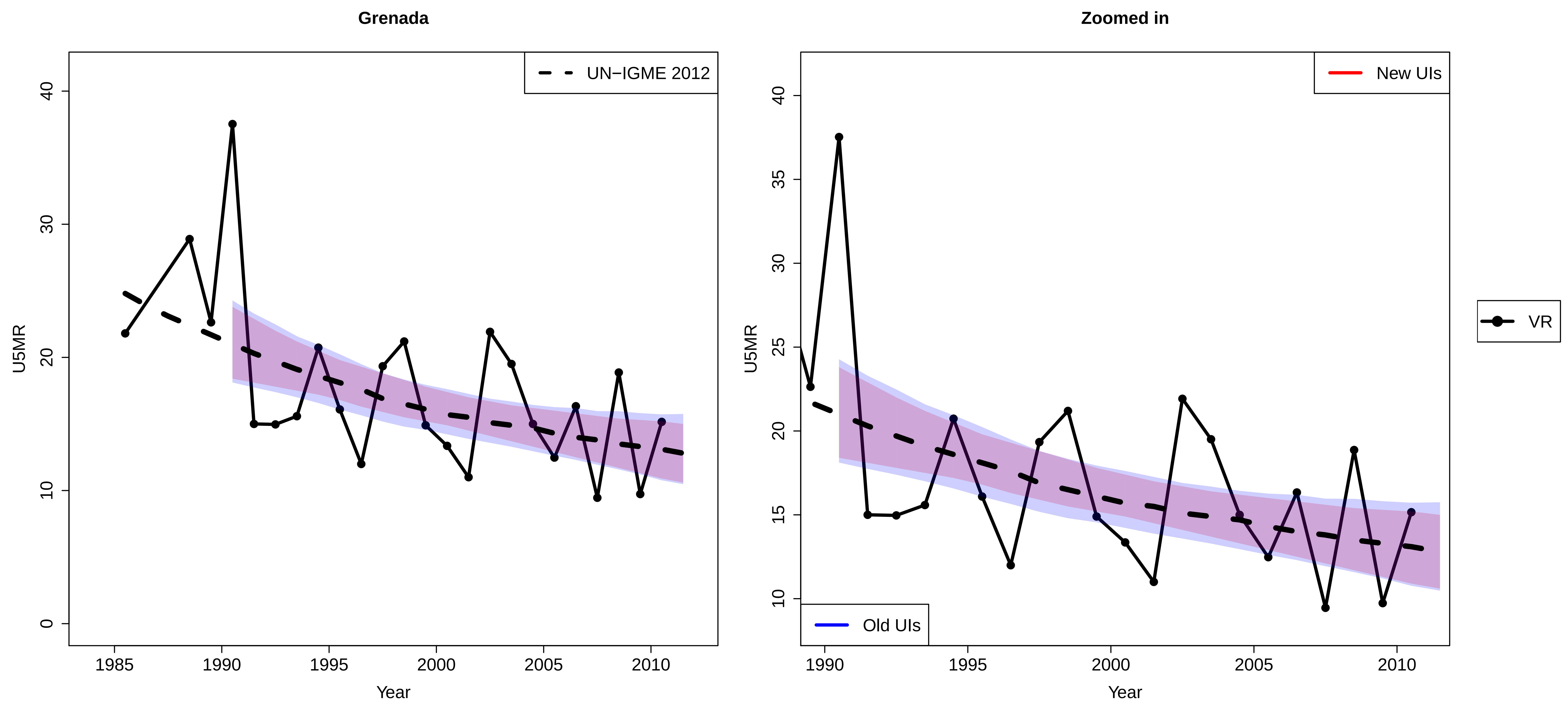

Grenada
UN−IGME 2012
U5MR
Year
Zoomed in
New UIs
Old UIs
VR

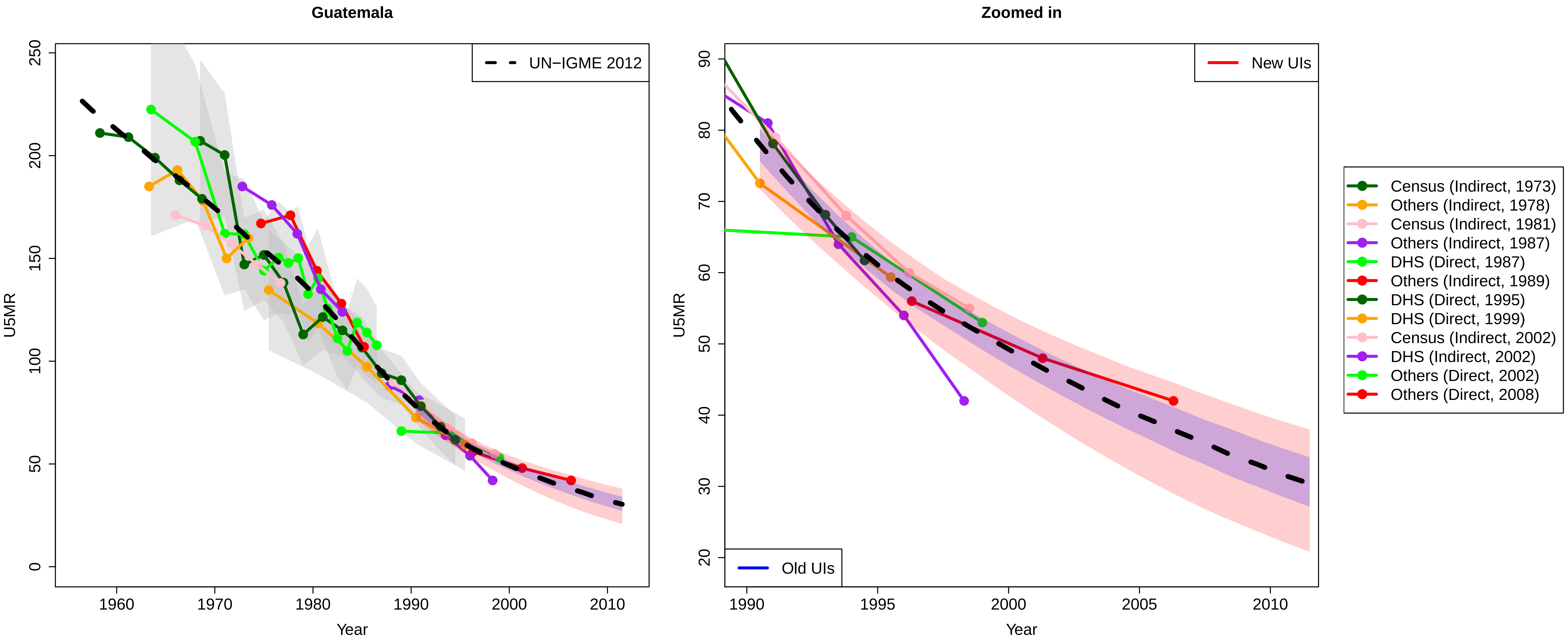

Guatemala
Zoomed in
UN−IGME 2012
New UIs
Old UIs
U5MR
Year
Census (Indirect, 1973)
Others (Indirect, 1978)
Census (Indirect, 1981)
Others (Indirect, 1987)
DHS (Direct, 1987)
Others (Indirect, 1989)
DHS (Direct, 1995)
DHS (Direct, 1999)
Census (Indirect, 2002)
DHS (Indirect, 2002)
Others (Direct, 2002)
Others (Direct, 2008)

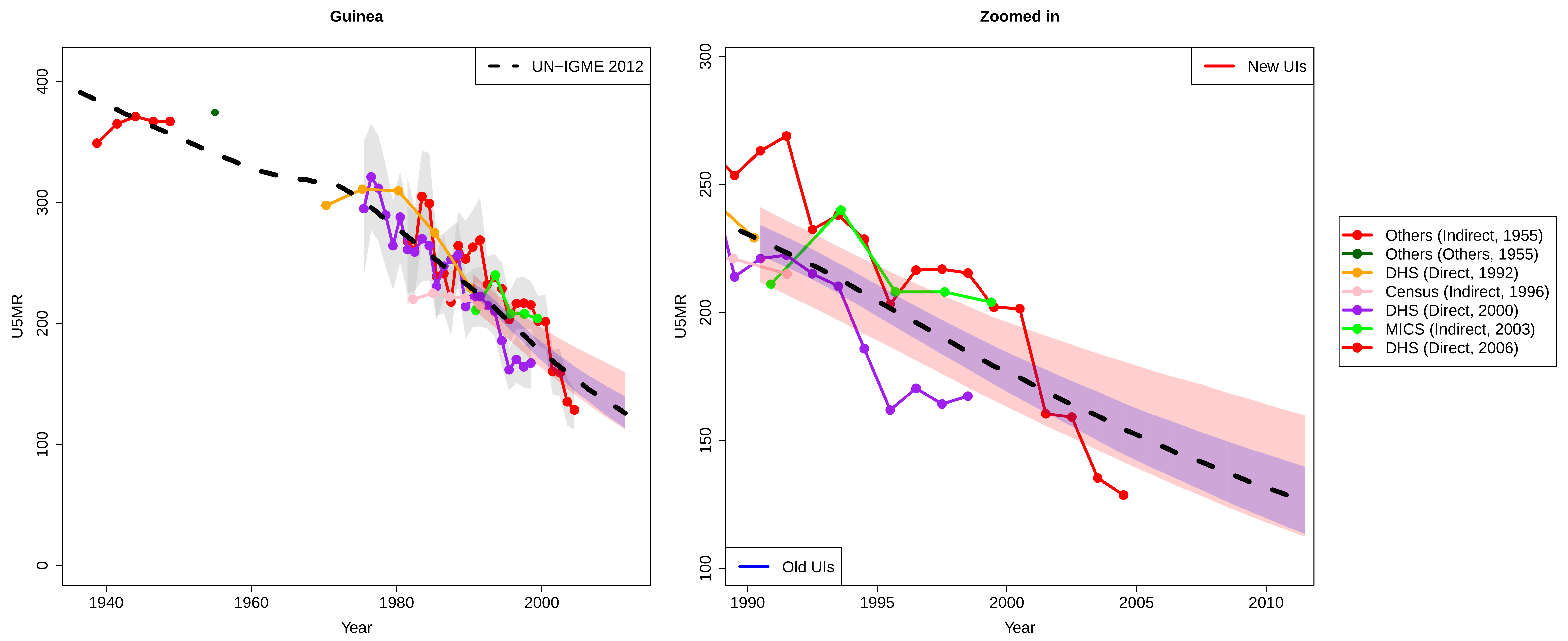

Guinea
UN−IGME 2012
U5MR
Year
0
100
200
300
400
1940
1960
1980
2000
Zoomed in
New UIs
Old UIs
U5MR
Year
100
150
200
250
300
1990
1995
2000
2005
2010
Others (Indirect, 1955)
Others (Others, 1955)
DHS (Direct, 1992)
Census (Indirect, 1996)
DHS (Direct, 2000)
MICS (Indirect, 2003)
DHS (Direct, 2006)

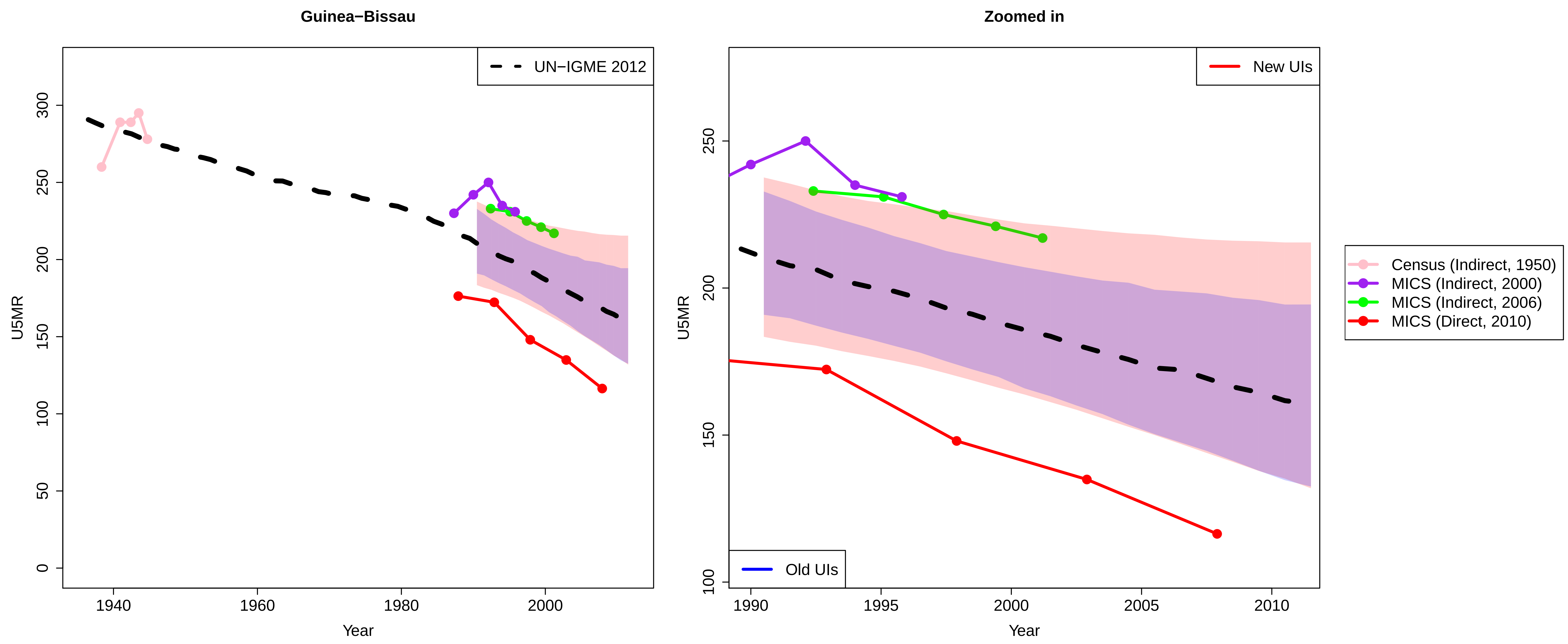

Guinea−Bissau
UN−IGME 2012
U5MR
Year
Zoomed in
New UIs
Old UIs
Census (Indirect, 1950)
MICS (Indirect, 2000)
MICS (Indirect, 2006)
MICS (Direct, 2010)

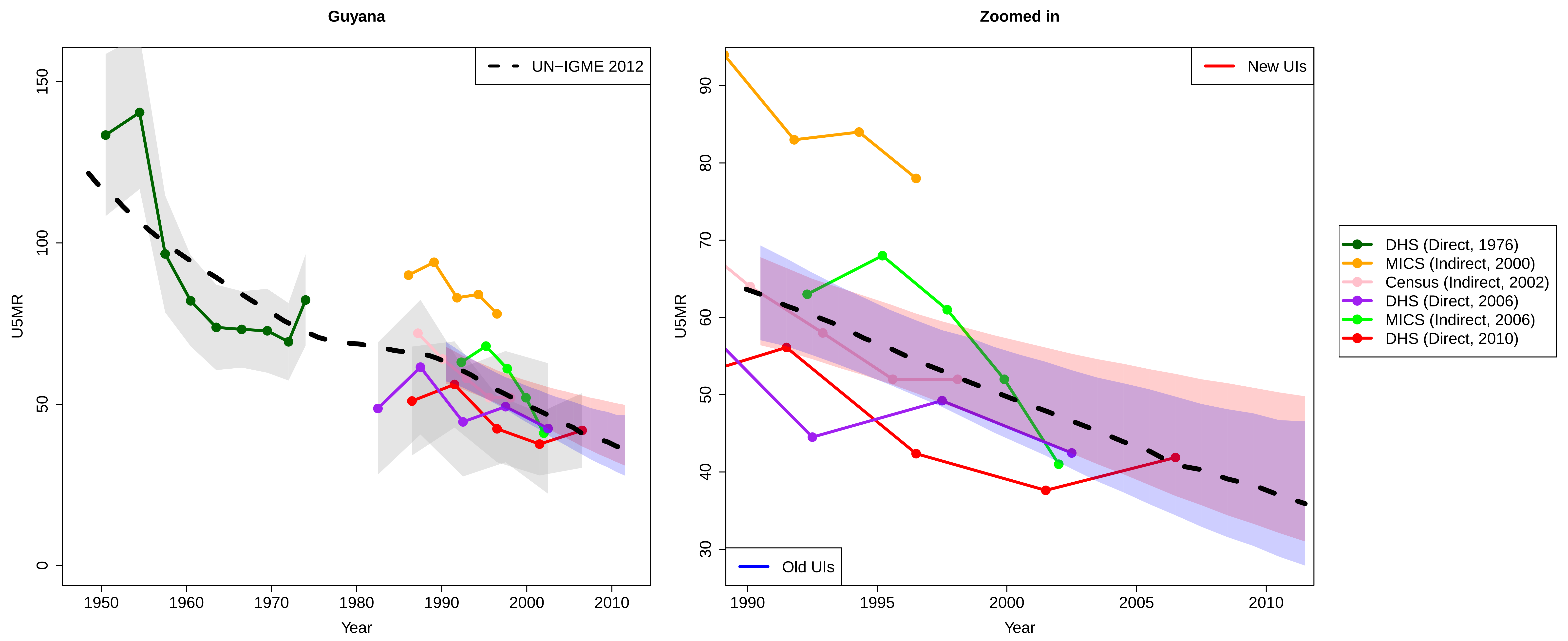
Guyana
UN−IGME 2012
U5MR
Year
Zoomed in
New UIs
Old UIs
DHS (Direct, 1976)
MICS (Indirect, 2000)
Census (Indirect, 2002)
DHS (Direct, 2006)
MICS (Indirect, 2006)
DHS (Direct, 2010)

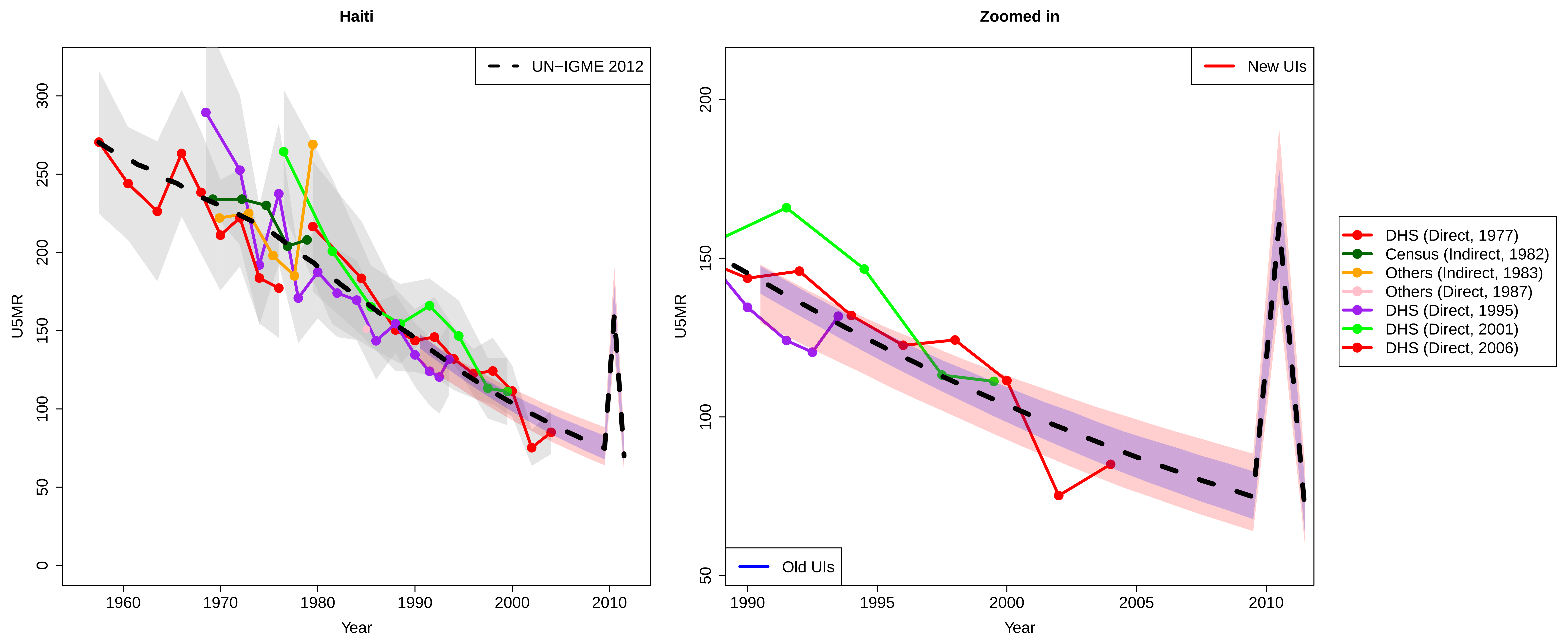

Haiti
Zoomed in
UN−IGME 2012
New UIs
Old UIs
DHS (Direct, 1977)
Census (Indirect, 1982)
Others (Indirect, 1983)
Others (Direct, 1987)
DHS (Direct, 1995)
DHS (Direct, 2001)
DHS (Direct, 2006)
U5MR
Year

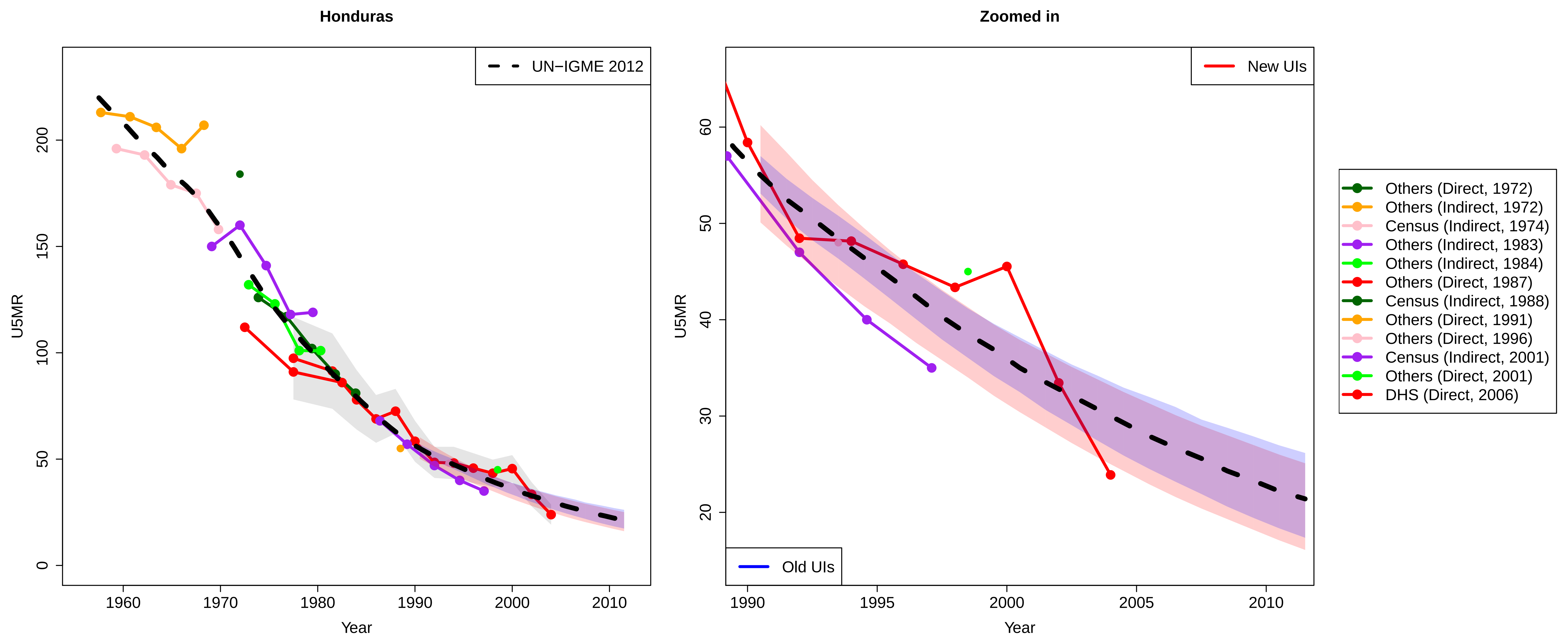

Honduras
UN−IGME 2012
U5MR
Year
Zoomed in
New UIs
Old UIs
U5MR
Year
Others (Direct, 1972)
Others (Indirect, 1972)
Census (Indirect, 1974)
Others (Indirect, 1983)
Others (Indirect, 1984)
Others (Direct, 1987)
Census (Indirect, 1988)
Others (Direct, 1991)
Others (Direct, 1996)
Census (Indirect, 2001)
Others (Direct, 2001)
DHS (Direct, 2006)

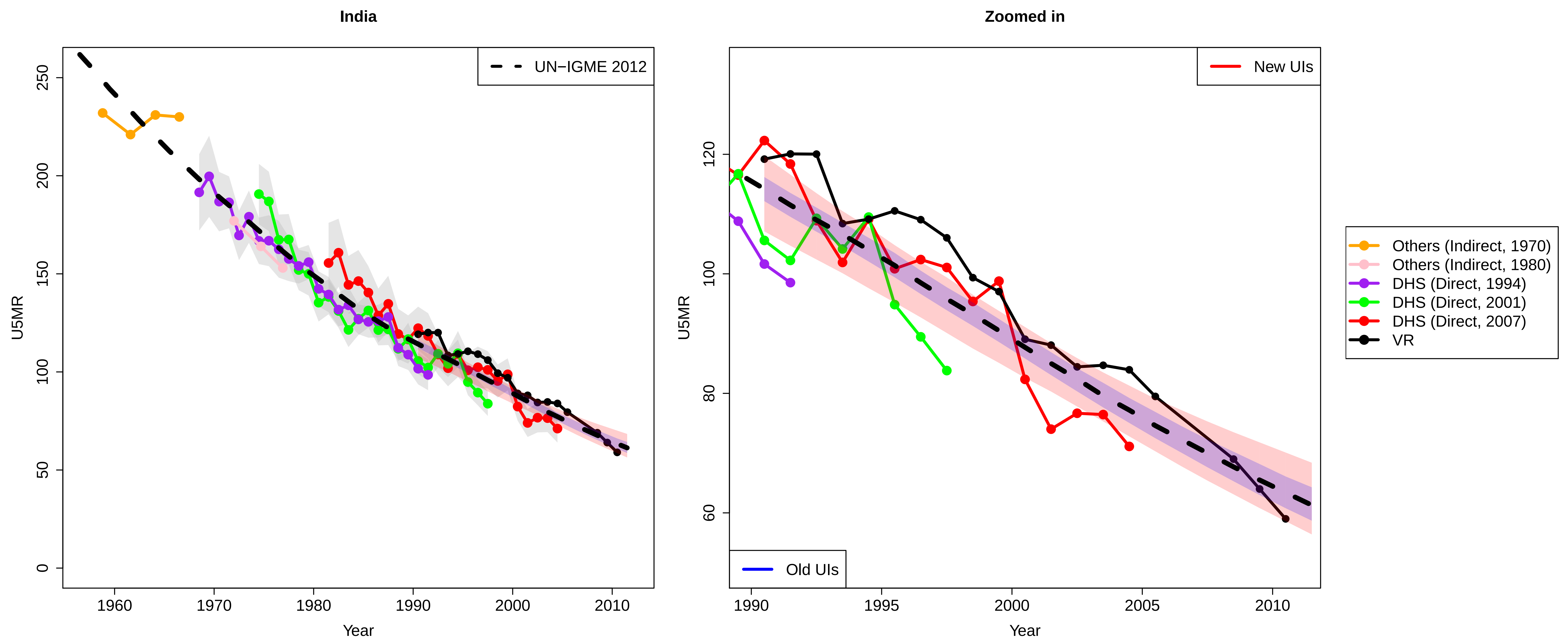
India
Zoomed in
UN−IGME 2012
New UIs
Old UIs
Others (Indirect, 1970)
Others (Indirect, 1980)
DHS (Direct, 1994)
DHS (Direct, 2001)
DHS (Direct, 2007)
VR
U5MR
Year
0
50
100
150
200
250
60
80
100
120
1960
1970
1980
1990
2000
2010
1990
1995
2000
2005
2010

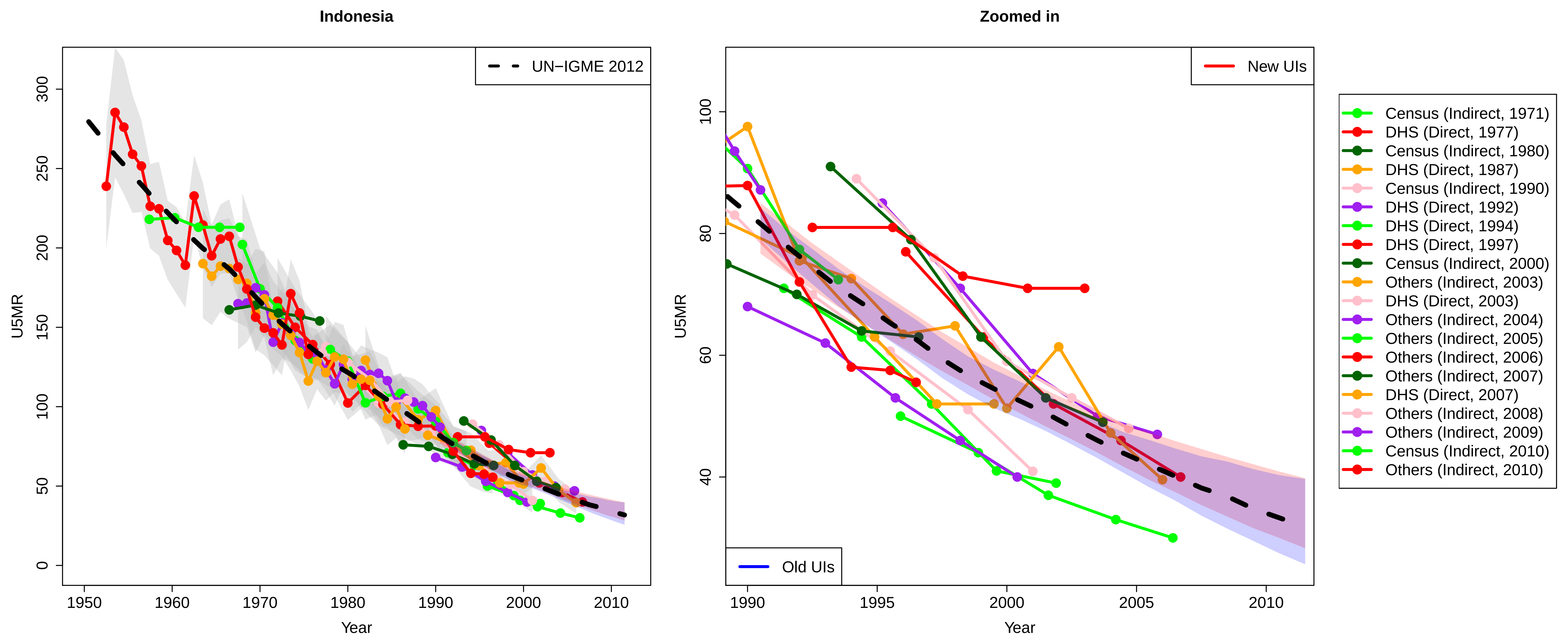
Indonesia
Zoomed in
UN–IGME 2012
New UIs
Old UIs
U5MR
Year
Census (Indirect, 1971)
DHS (Direct, 1977)
Census (Indirect, 1980)
DHS (Direct, 1987)
Census (Indirect, 1990)
DHS (Direct, 1992)
DHS (Direct, 1994)
DHS (Direct, 1997)
Census (Indirect, 2000)
Others (Indirect, 2003)
DHS (Direct, 2003)
Others (Indirect, 2004)
Others (Indirect, 2005)
Others (Indirect, 2006)
Others (Indirect, 2007)
DHS (Direct, 2007)
Others (Indirect, 2008)
Others (Indirect, 2009)
Census (Indirect, 2010)
Others (Indirect, 2010)

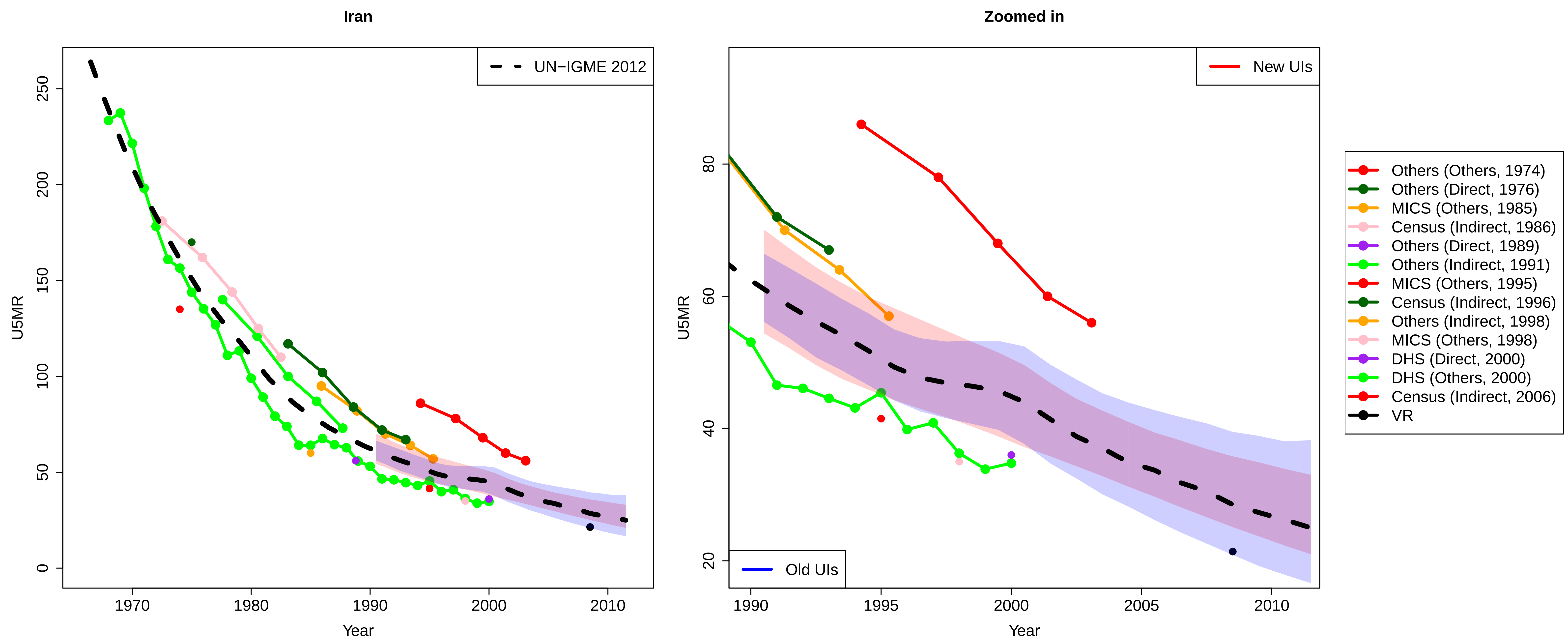

Iran
Zoomed in
UN−IGME 2012
New UIs
Old UIs
U5MR
Year
Others (Others, 1974)
Others (Direct, 1976)
MICS (Others, 1985)
Census (Indirect, 1986)
Others (Direct, 1989)
Others (Indirect, 1991)
MICS (Others, 1995)
Census (Indirect, 1996)
Others (Indirect, 1998)
MICS (Others, 1998)
DHS (Direct, 2000)
DHS (Others, 2000)
Census (Indirect, 2006)
VR

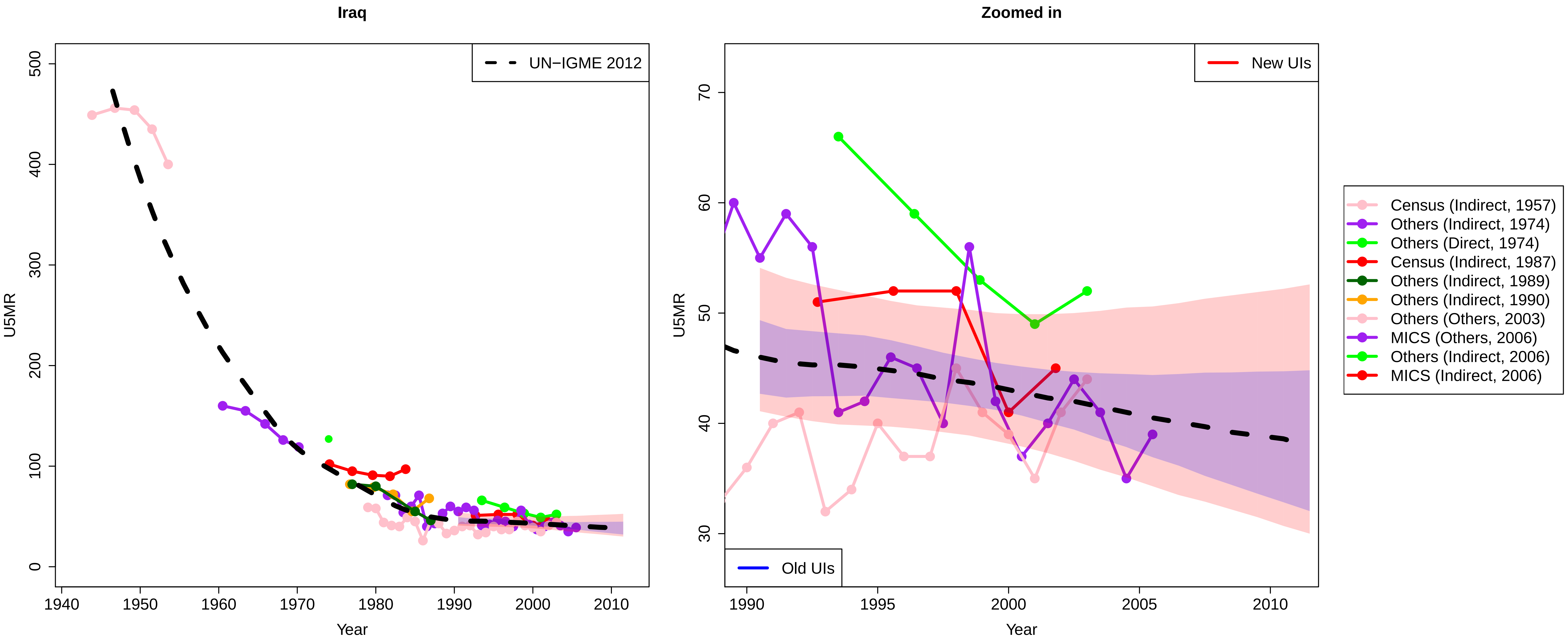

Iraq
UN−IGME 2012
U5MR
0
100
200
300
400
500
1940
1950
1960
1970
1980
1990
2000
2010
Year
Zoomed in
New UIs
Old UIs
U5MR
30
40
50
60
70
1990
1995
2000
2005
2010
Year
Census (Indirect, 1957)
Others (Indirect, 1974)
Others (Direct, 1974)
Census (Indirect, 1987)
Others (Indirect, 1989)
Others (Indirect, 1990)
Others (Others, 2003)
MICS (Others, 2006)
Others (Indirect, 2006)
MICS (Indirect, 2006)

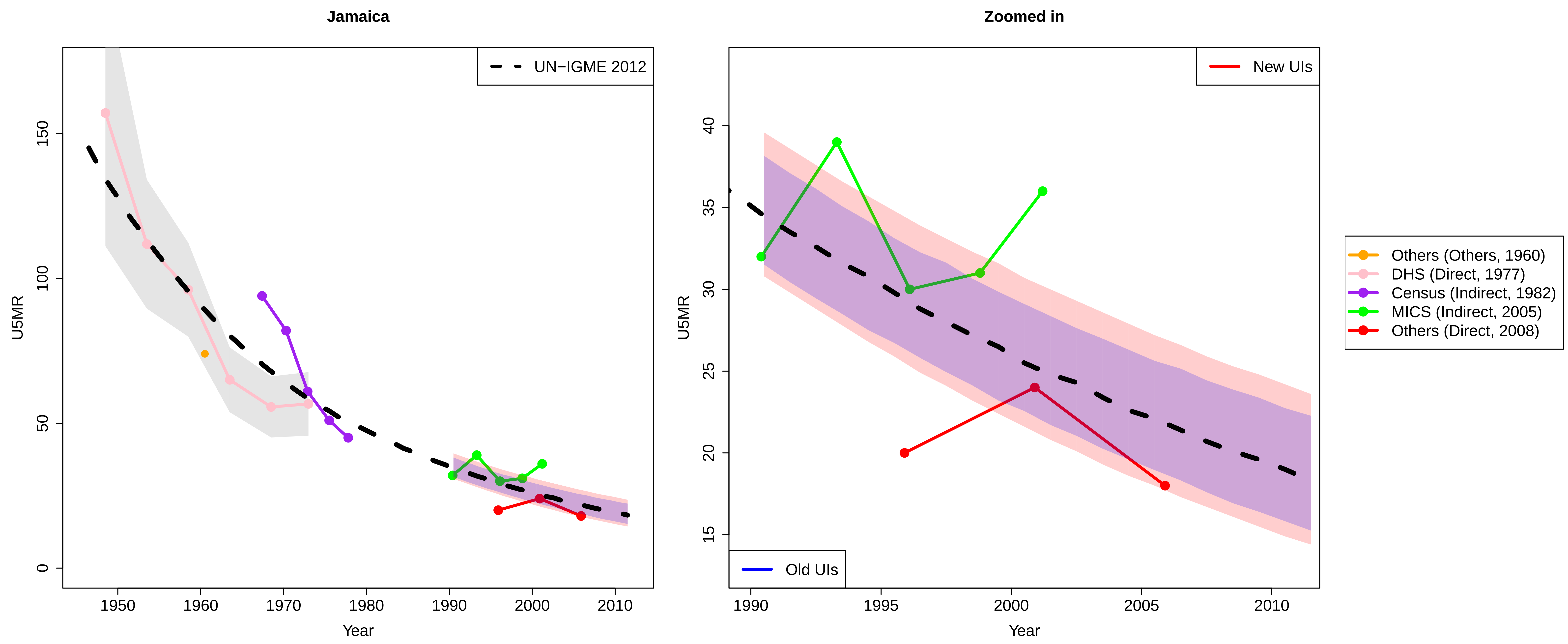

Jamaica
Zoomed in
UN−IGME 2012
New UIs
Old UIs
U5MR
Year
0
50
100
150
1950
1960
1970
1980
1990
2000
2010
15
20
25
30
35
40
1990
1995
2000
2005
2010
Others (Others, 1960)
DHS (Direct, 1977)
Census (Indirect, 1982)
MICS (Indirect, 2005)
Others (Direct, 2008)

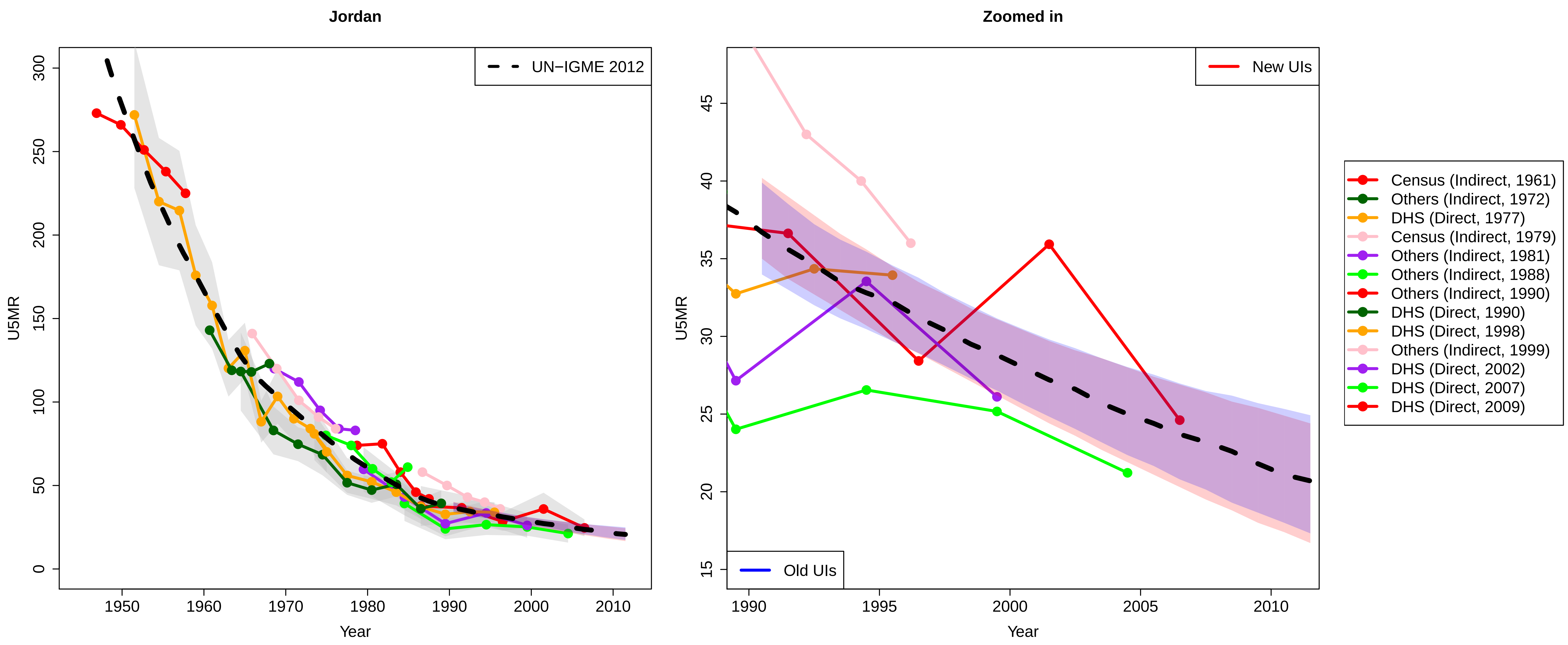
Jordan
UN−IGME 2012
U5MR
0
50
100
150
200
250
300
1950
1960
1970
1980
1990
2000
2010
Year
Zoomed in
New UIs
Old UIs
U5MR
15
20
25
30
35
40
45
1990
1995
2000
2005
2010
Year
Census (Indirect, 1961)
Others (Indirect, 1972)
DHS (Direct, 1977)
Census (Indirect, 1979)
Others (Indirect, 1981)
Others (Indirect, 1988)
Others (Indirect, 1990)
DHS (Direct, 1990)
DHS (Direct, 1998)
Others (Indirect, 1999)
DHS (Direct, 2002)
DHS (Direct, 2007)
DHS (Direct, 2009)

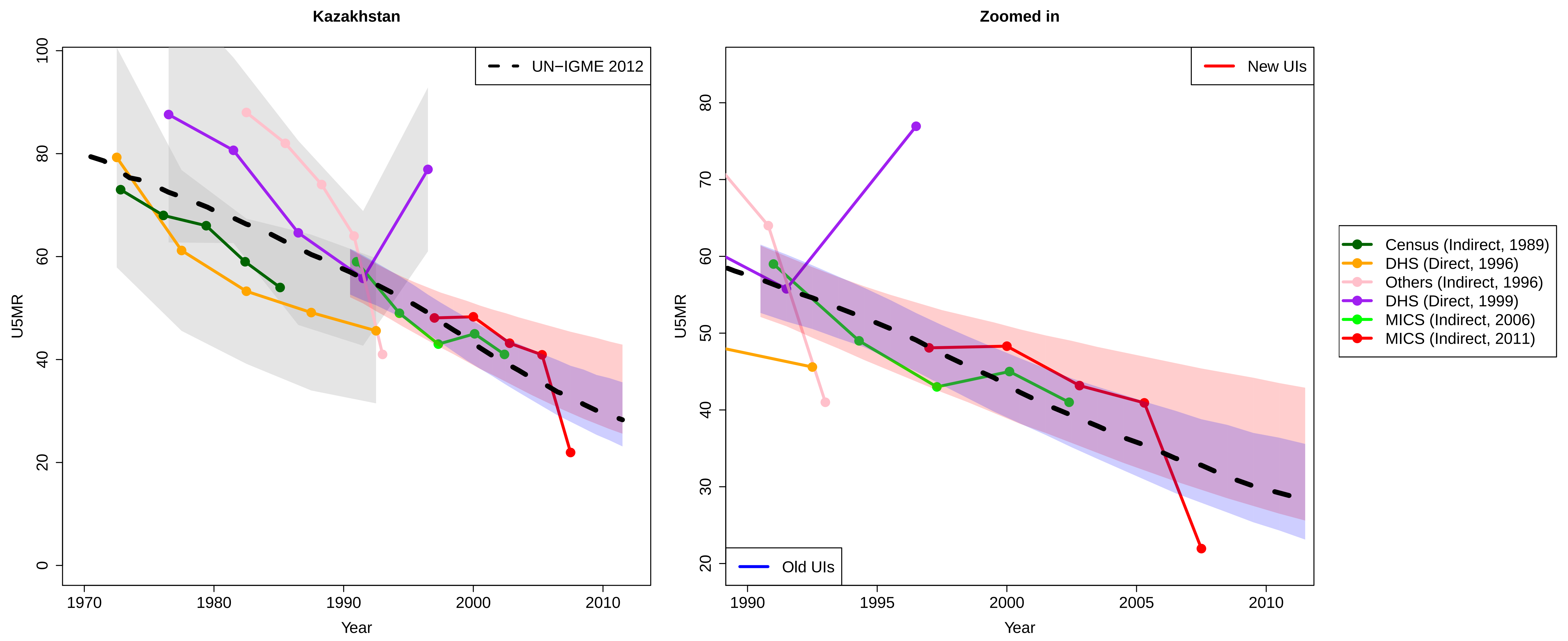
Kazakhstan
Zoomed in
UN−IGME 2012
New UIs
Old UIs
Census (Indirect, 1989)
DHS (Direct, 1996)
Others (Indirect, 1996)
DHS (Direct, 1999)
MICS (Indirect, 2006)
MICS (Indirect, 2011)
U5MR
Year

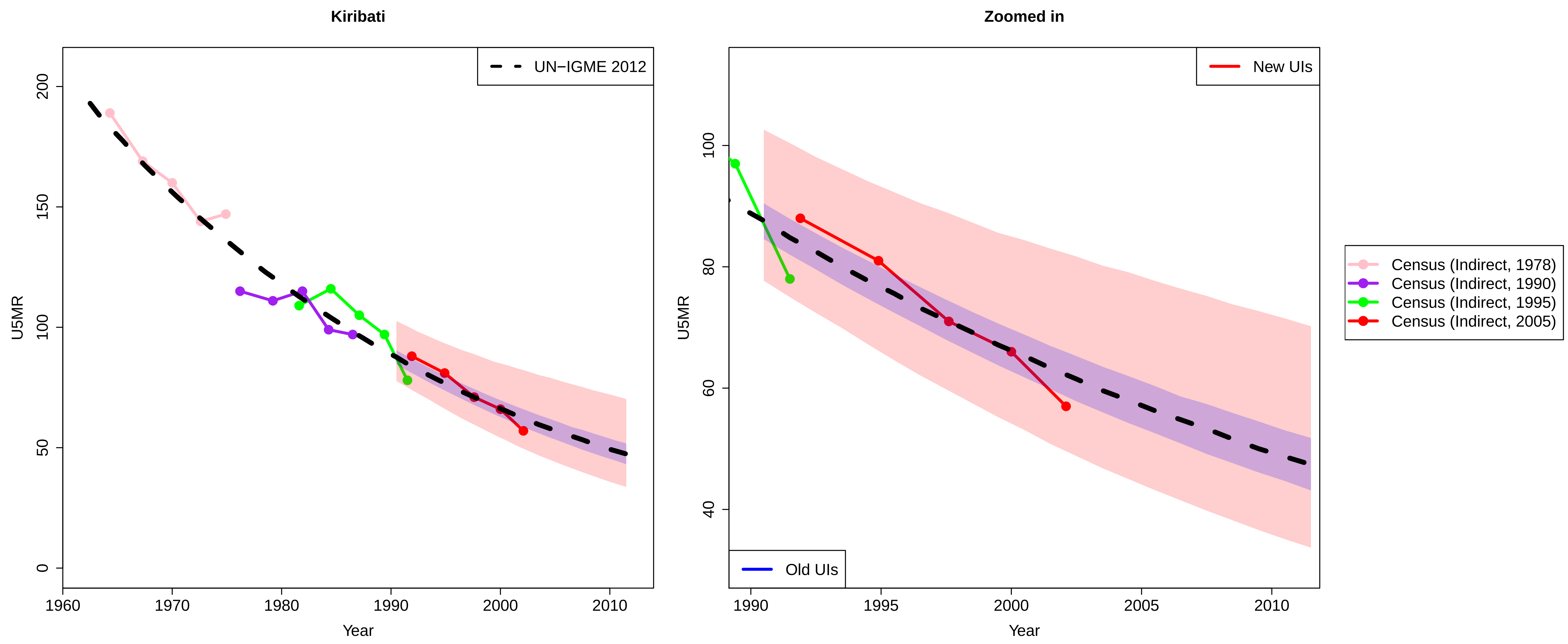

Kiribati
Zoomed in
UN−IGME 2012
New UIs
Old UIs
U5MR
Year
Census (Indirect, 1978)
Census (Indirect, 1990)
Census (Indirect, 1995)
Census (Indirect, 2005)

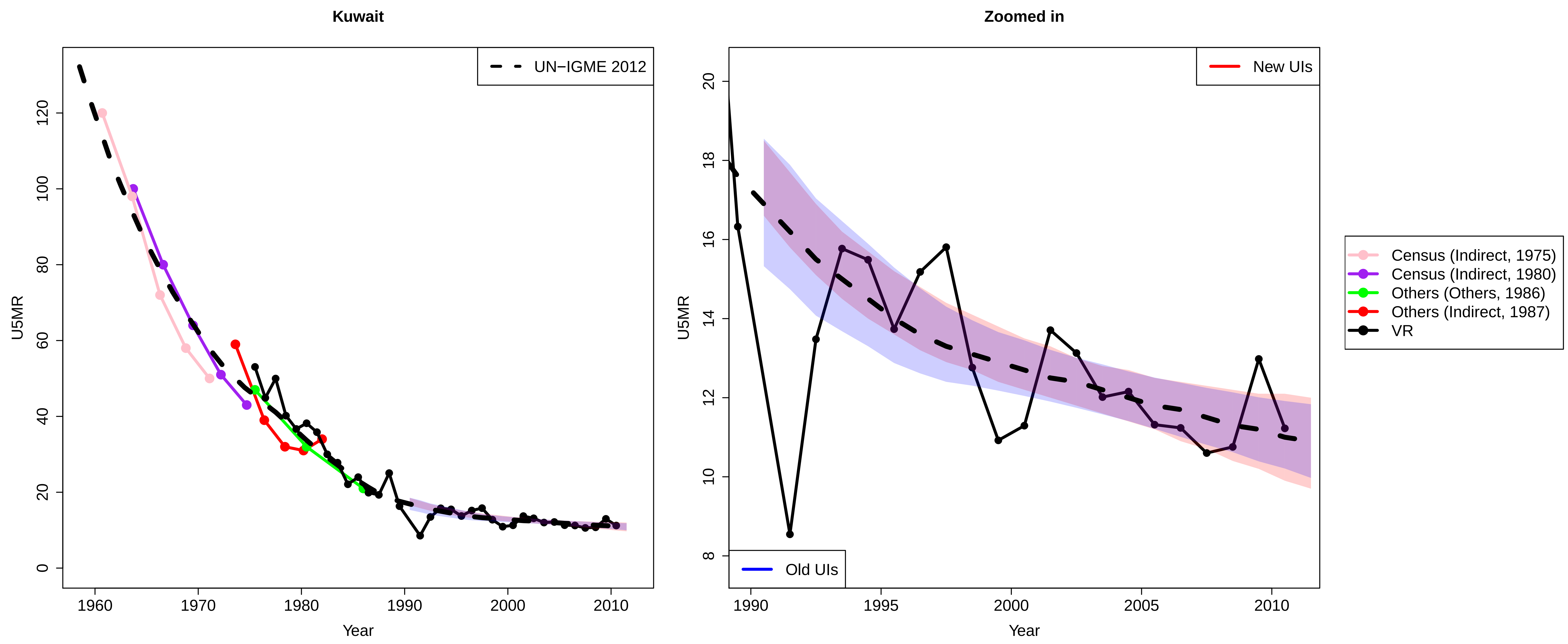

Kuwait
UN−IGME 2012
U5MR
Year
Zoomed in
New UIs
Old UIs
Census (Indirect, 1975)
Census (Indirect, 1980)
Others (Others, 1986)
Others (Indirect, 1987)
VR

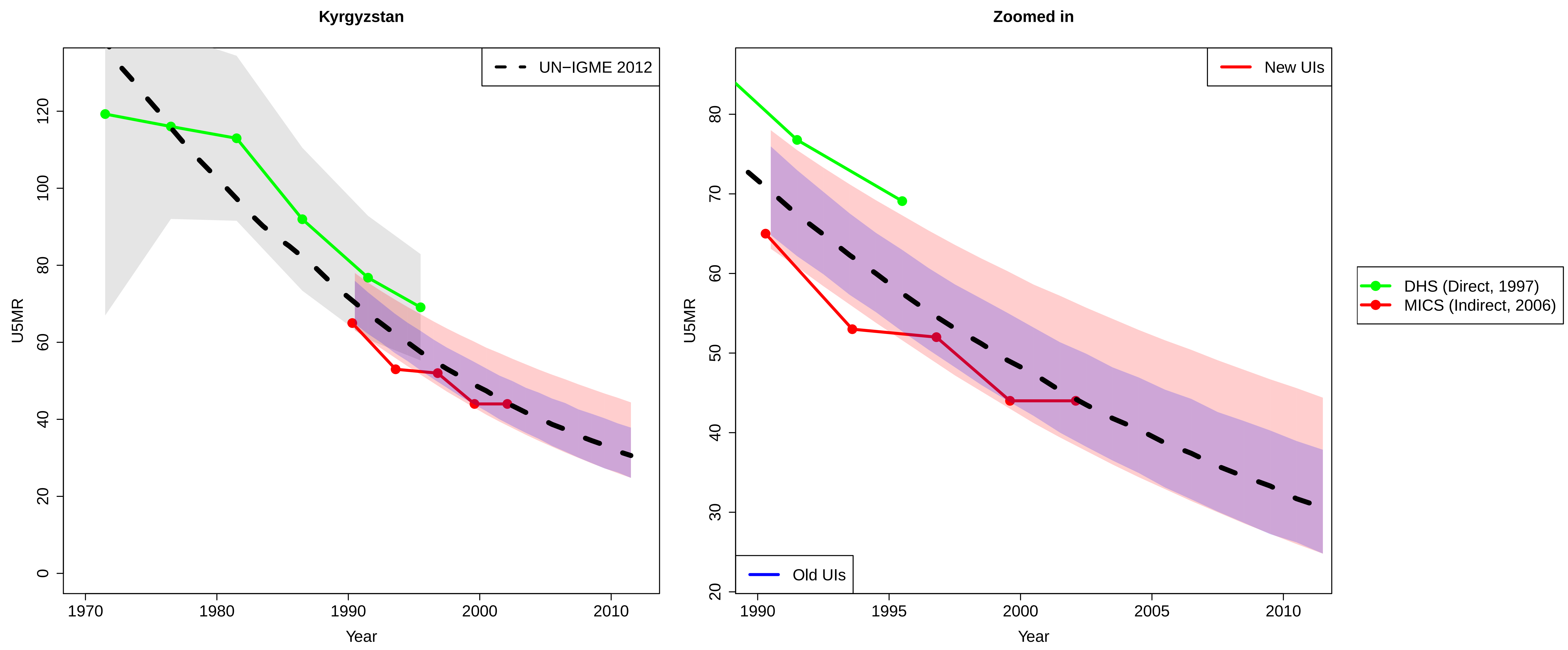
Kyrgyzstan
Zoomed in
UN−IGME 2012
New UIs
Old UIs
DHS (Direct, 1997)
MICS (Indirect, 2006)
U5MR
Year
0
20
40
60
80
100
120
30
50
70
1970
1980
1990
2000
2010
1995
2005

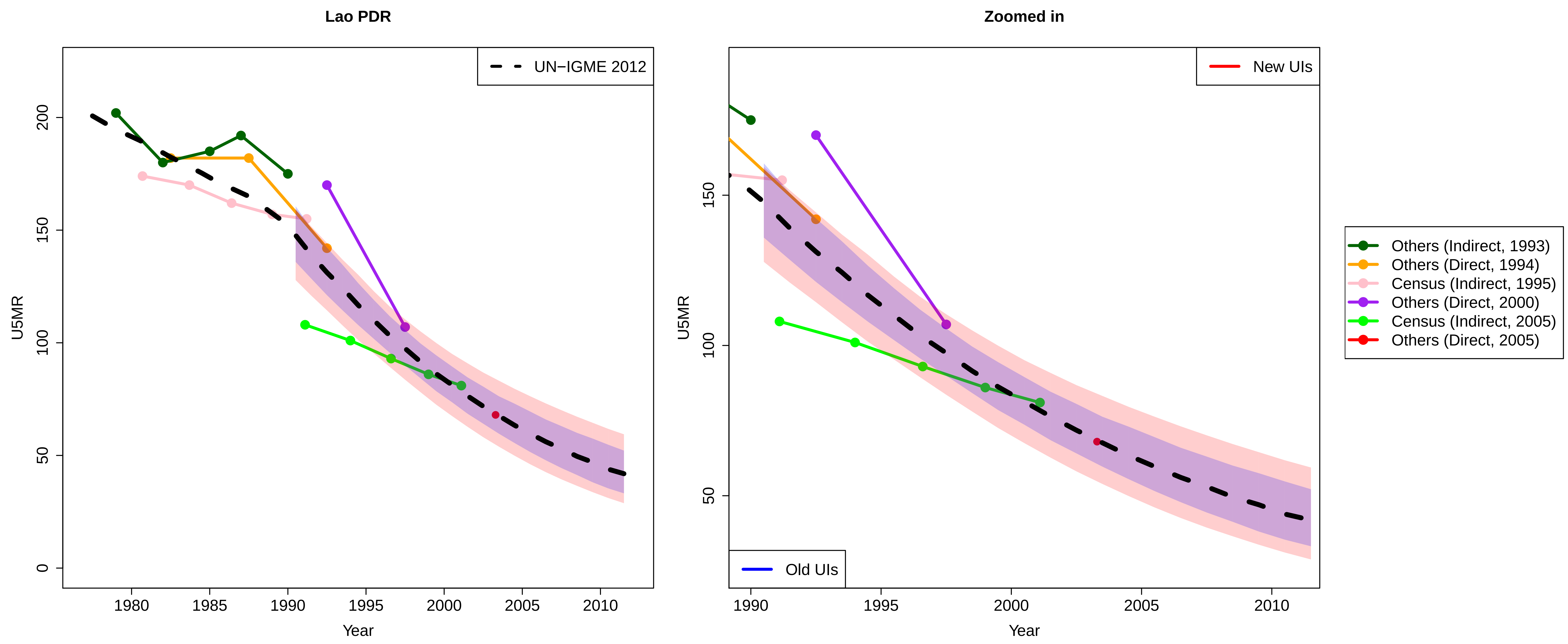

Lao PDR
Zoomed in
UN−IGME 2012
New UIs
Old UIs
U5MR
Year
Others (Indirect, 1993)
Others (Direct, 1994)
Census (Indirect, 1995)
Others (Direct, 2000)
Census (Indirect, 2005)
Others (Direct, 2005)

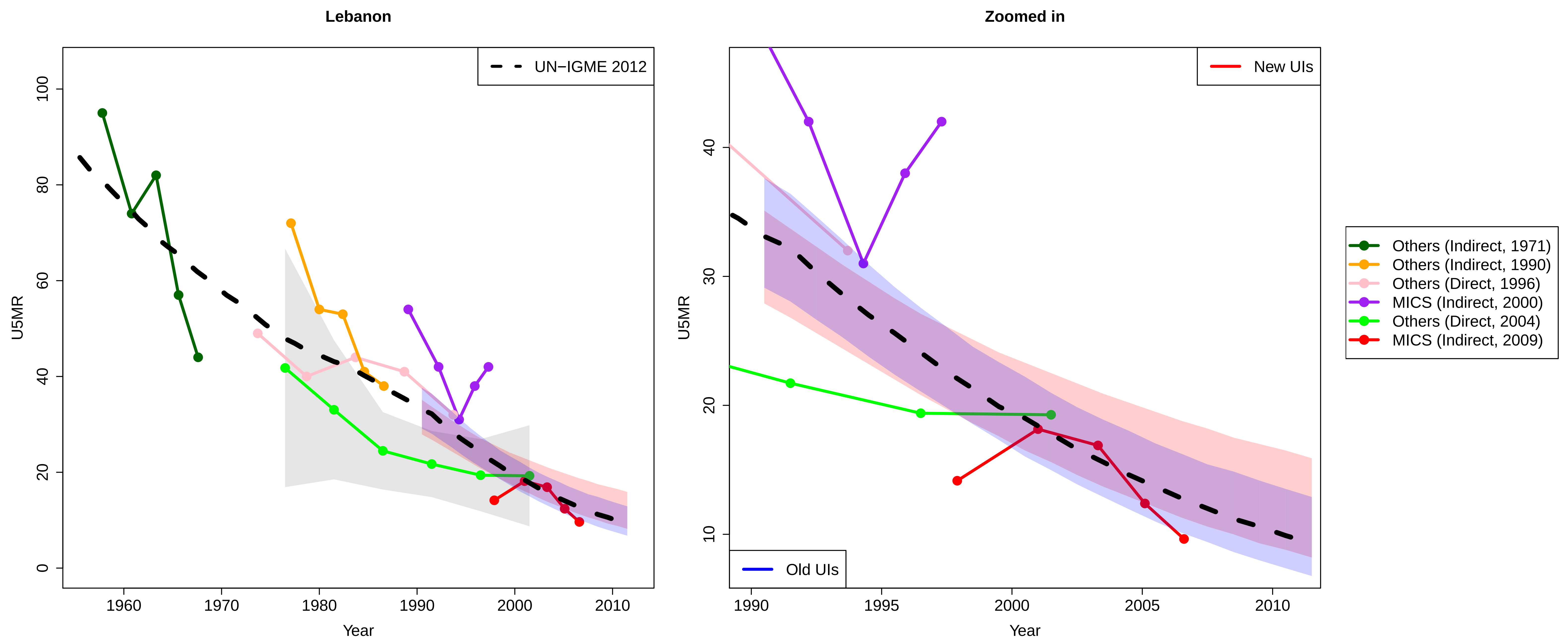

Lebanon
Zoomed in
UN−IGME 2012
New UIs
Old UIs
U5MR
Year
Others (Indirect, 1971)
Others (Indirect, 1990)
Others (Direct, 1996)
MICS (Indirect, 2000)
Others (Direct, 2004)
MICS (Indirect, 2009)

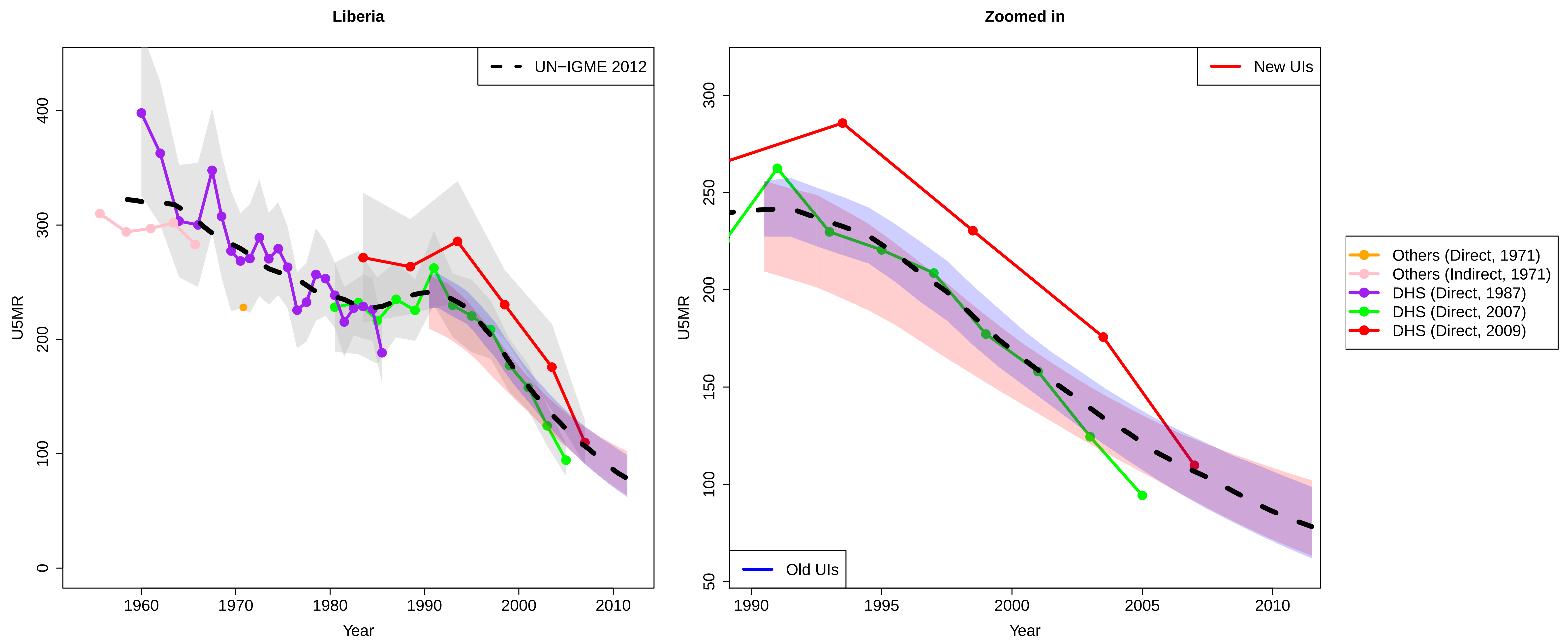
Liberia
Zoomed in
UN−IGME 2012
New UIs
Old UIs
Others (Direct, 1971)
Others (Indirect, 1971)
DHS (Direct, 1987)
DHS (Direct, 2007)
DHS (Direct, 2009)
U5MR
Year

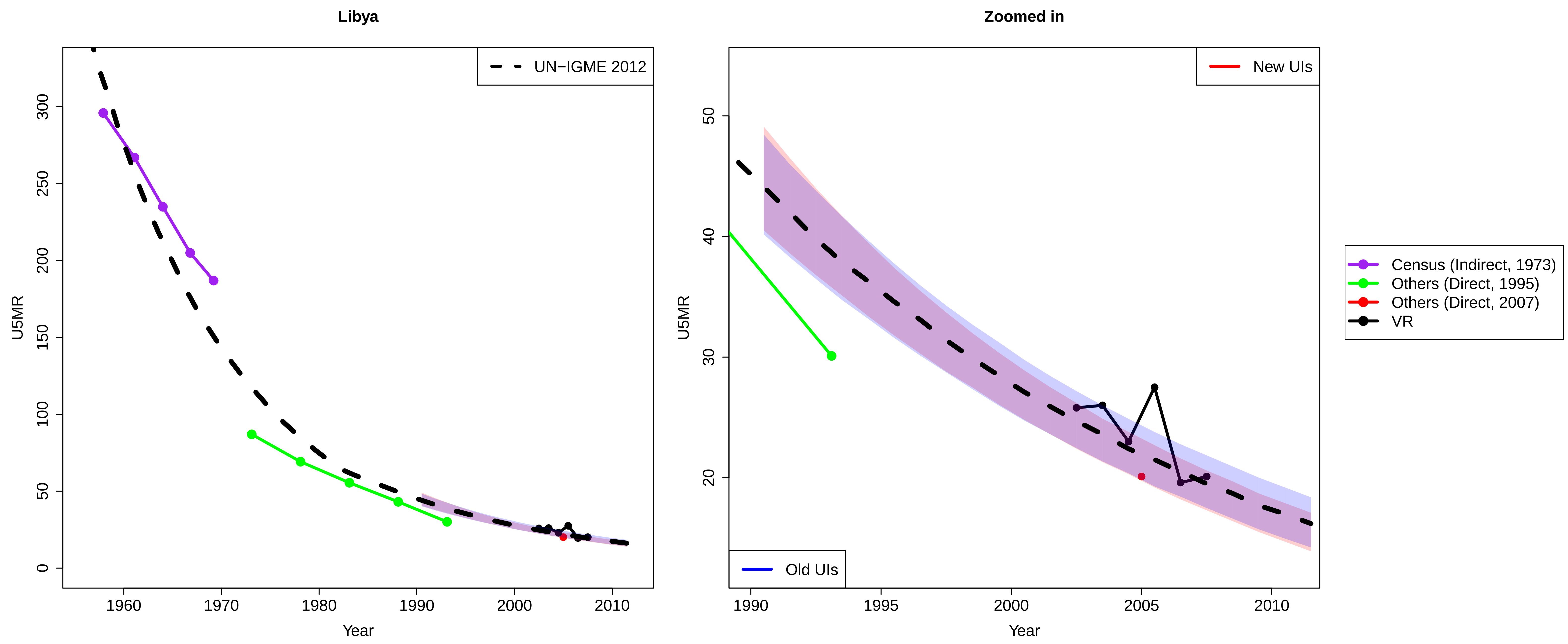
Libya
UN−IGME 2012
U5MR
Year
Zoomed in
New UIs
Old UIs
Census (Indirect, 1973)
Others (Direct, 1995)
Others (Direct, 2007)
VR

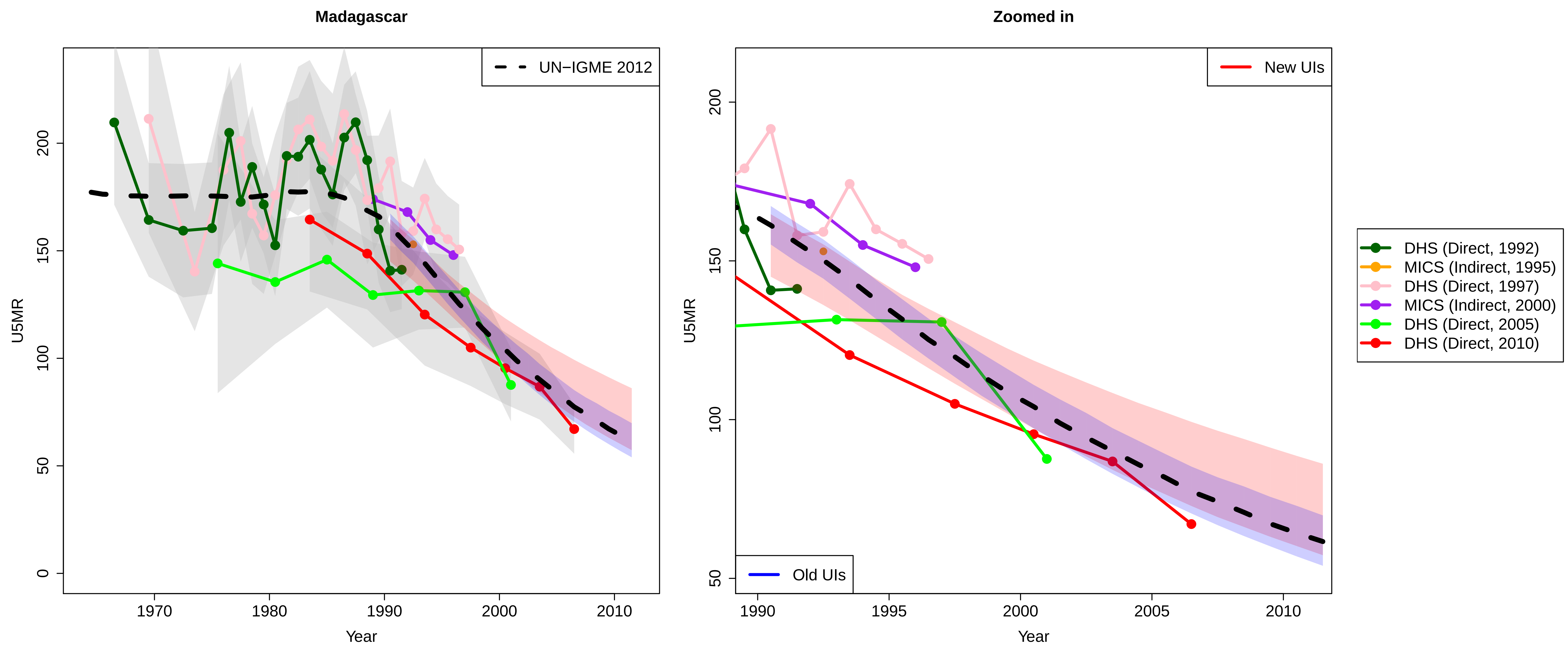
Madagascar
Zoomed in
UN−IGME 2012
New UIs
Old UIs
U5MR
Year
0
50
100
150
200
1970
1980
1990
2000
2010
1995
2005
DHS (Direct, 1992)
MICS (Indirect, 1995)
DHS (Direct, 1997)
MICS (Indirect, 2000)
DHS (Direct, 2005)
DHS (Direct, 2010)

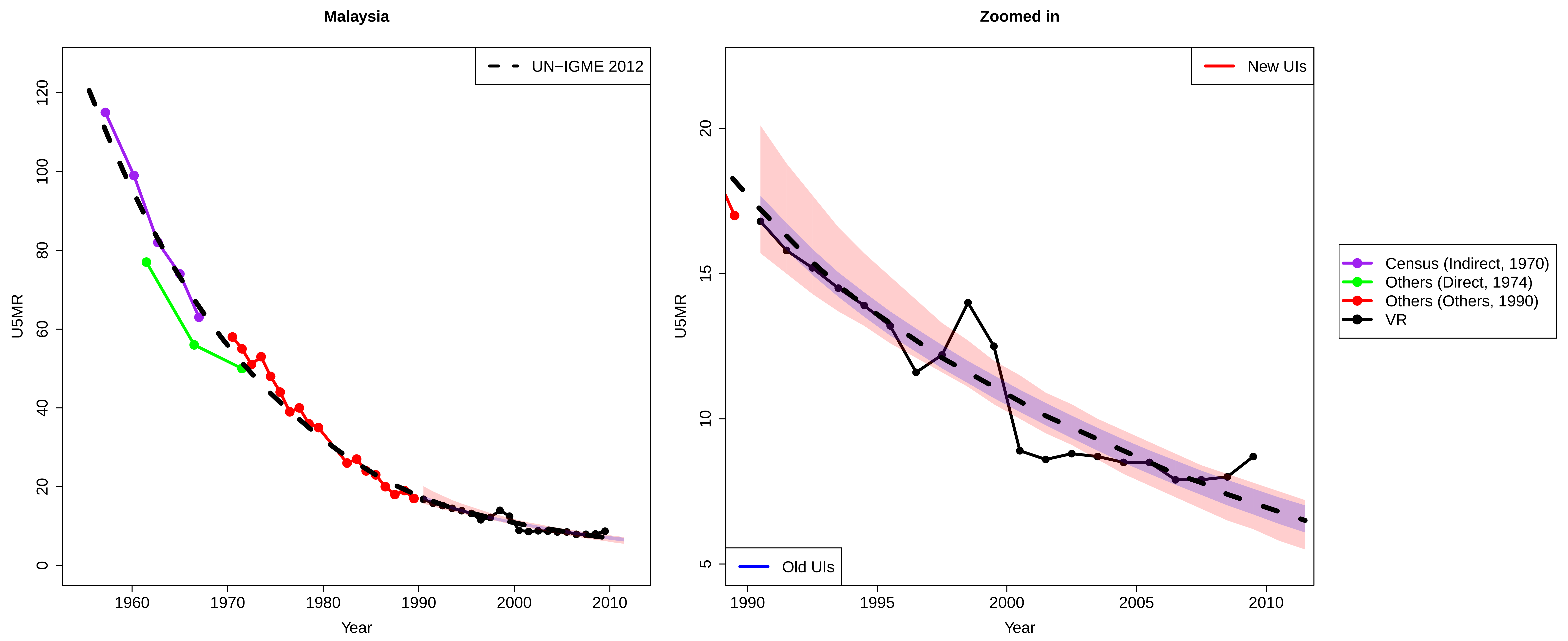
Malaysia
Zoomed in
UN−IGME 2012
New UIs
Old UIs
Census (Indirect, 1970)
Others (Direct, 1974)
Others (Others, 1990)
VR
U5MR
Year
0
20
40
60
80
100
120
1960
1970
1980
1990
2000
2010
5
10
15
20
1995
2005

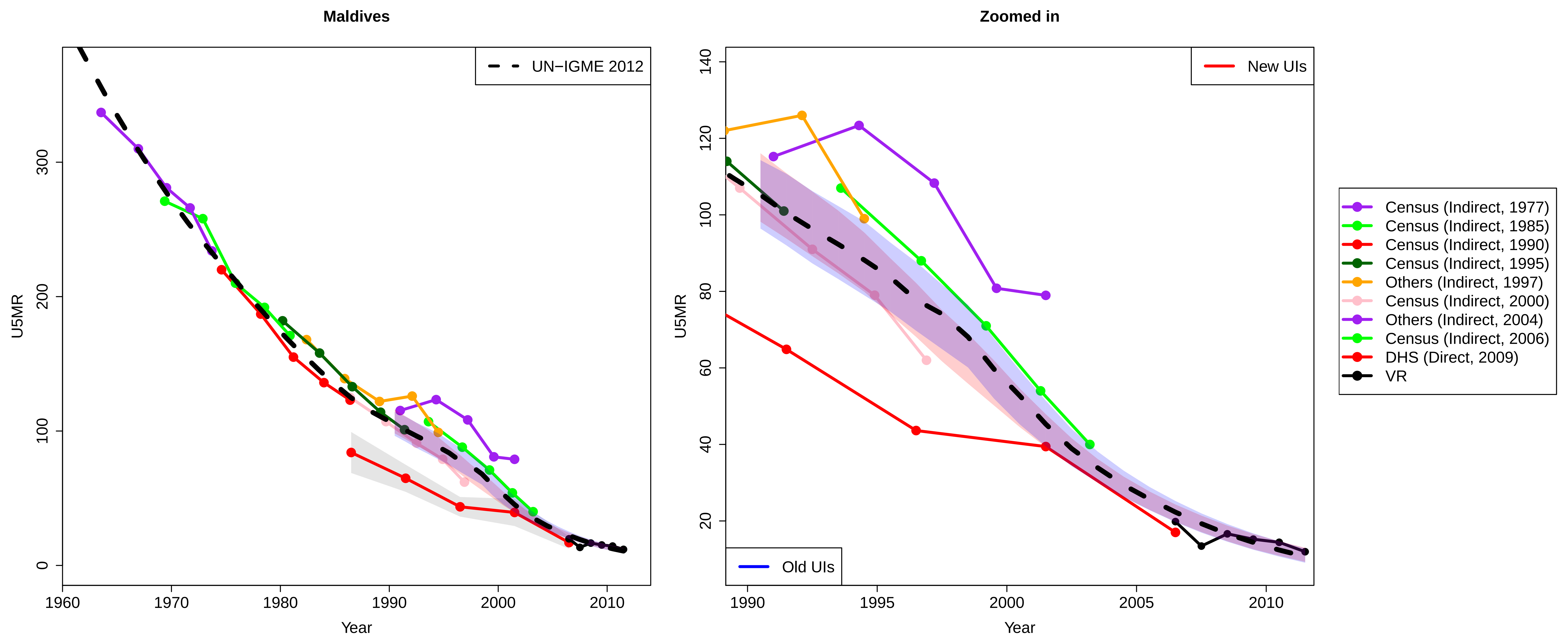
Maldives
UN−IGME 2012
U5MR
Year
Zoomed in
New UIs
Old UIs
Census (Indirect, 1977)
Census (Indirect, 1985)
Census (Indirect, 1990)
Census (Indirect, 1995)
Others (Indirect, 1997)
Census (Indirect, 2000)
Others (Indirect, 2004)
Census (Indirect, 2006)
DHS (Direct, 2009)
VR

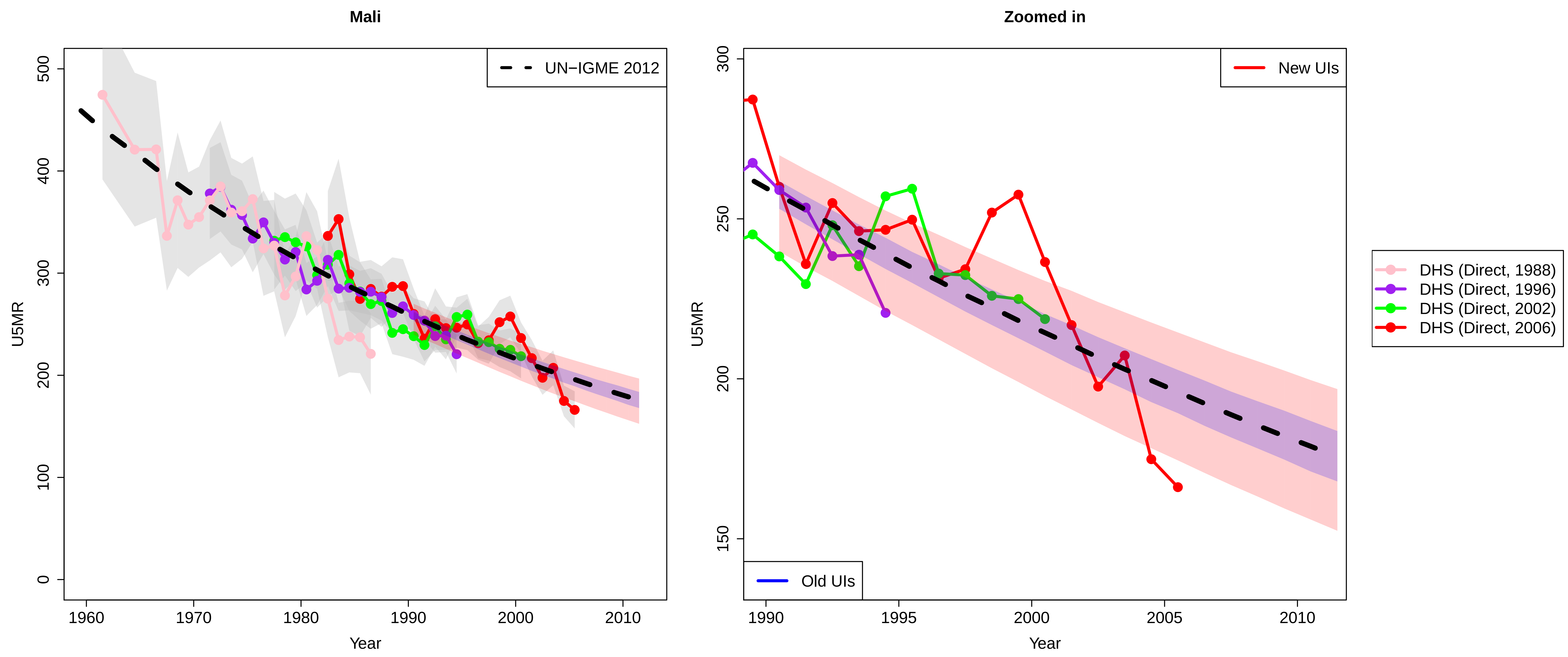
Mali
Zoomed in
UN−IGME 2012
New UIs
Old UIs
DHS (Direct, 1988)
DHS (Direct, 1996)
DHS (Direct, 2002)
DHS (Direct, 2006)
U5MR
Year
0
100
200
300
400
500
150
250
300
1960
1970
1980
1990
2000
2010
1995
2005

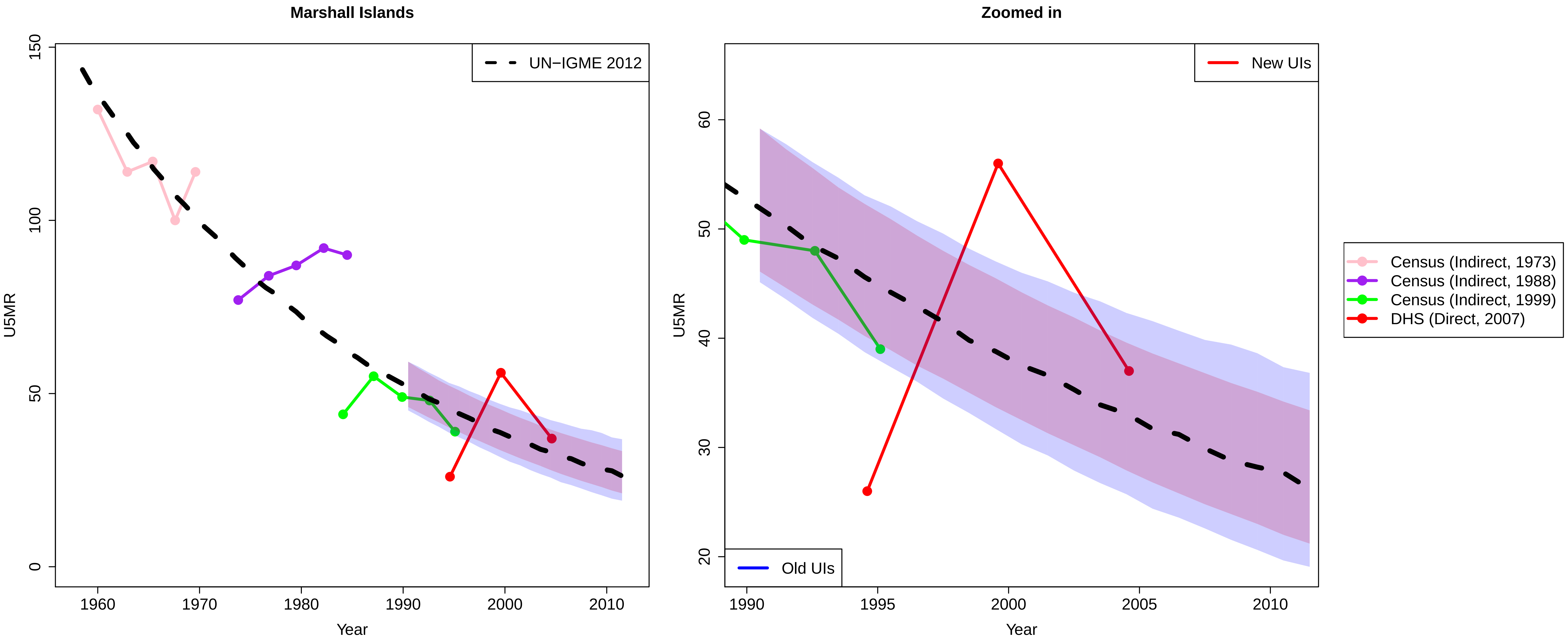

Marshall Islands
Zoomed in
UN−IGME 2012
New UIs
Old UIs
Census (Indirect, 1973)
Census (Indirect, 1988)
Census (Indirect, 1999)
DHS (Direct, 2007)
U5MR
Year
0
50
100
150
20
30
40
50
60
1960
1970
1980
1990
2000
2010
1995
2005

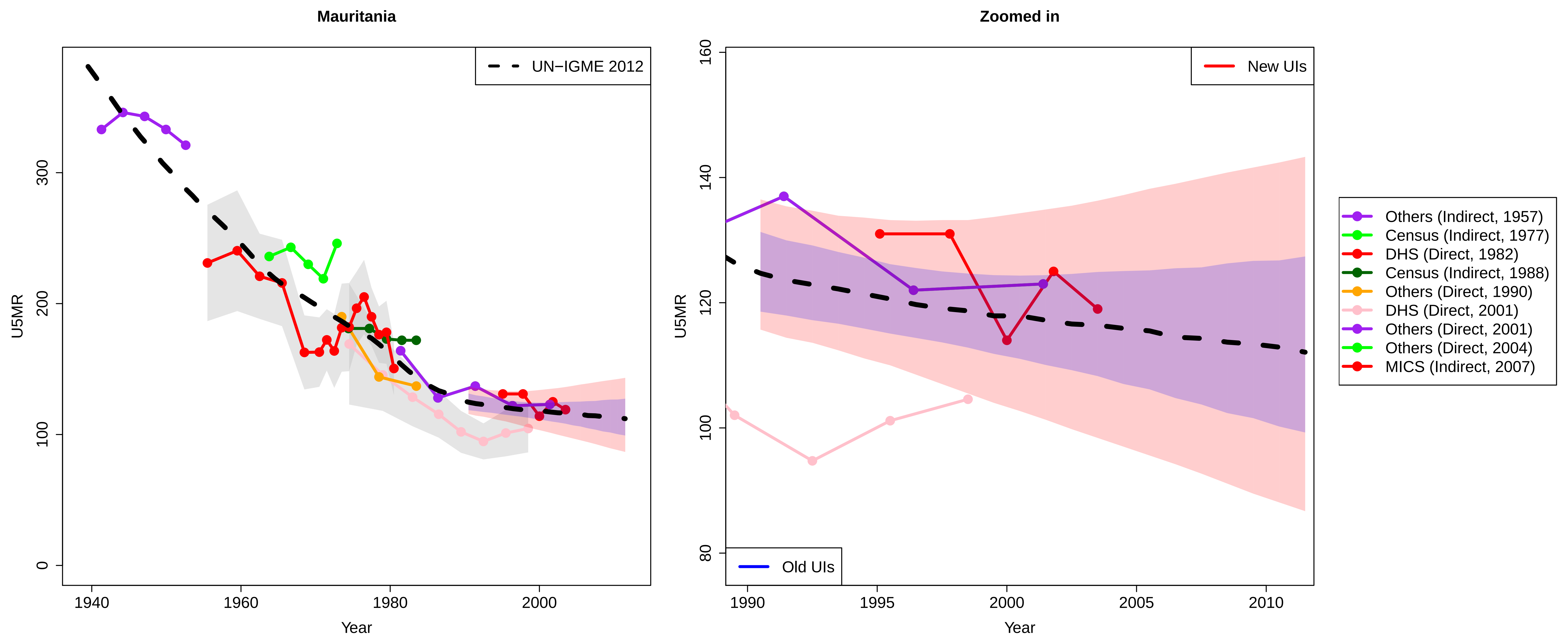

Mauritania
UN−IGME 2012
U5MR
Year
0
100
200
300
1940
1960
1980
2000
Zoomed in
New UIs
Old UIs
80
100
120
140
160
1990
1995
2000
2005
2010
Others (Indirect, 1957)
Census (Indirect, 1977)
DHS (Direct, 1982)
Census (Indirect, 1988)
Others (Direct, 1990)
DHS (Direct, 2001)
Others (Direct, 2001)
Others (Direct, 2004)
MICS (Indirect, 2007)

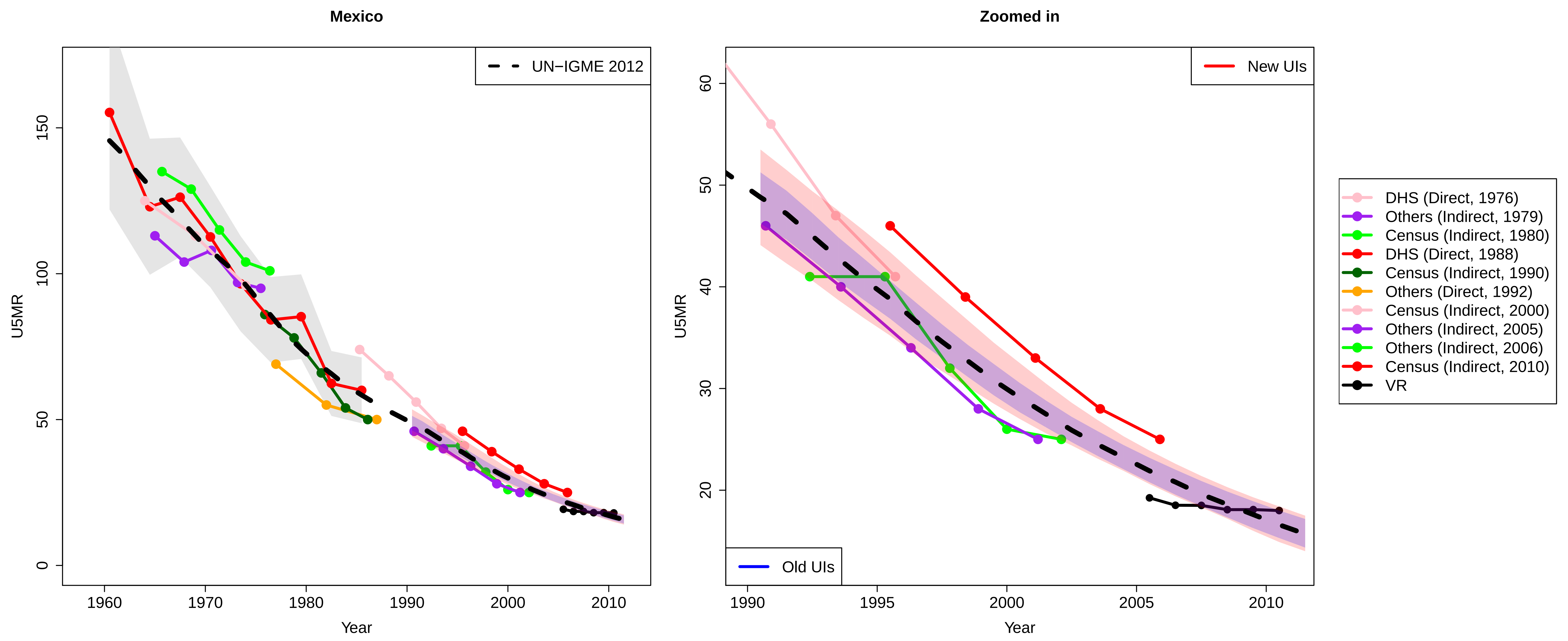

Mexico
Zoomed in
UN−IGME 2012
New UIs
Old UIs
U5MR
Year
0
50
100
150
1960
1970
1980
1990
2000
2010
20
30
40
50
60
1995
2005
DHS (Direct, 1976)
Others (Indirect, 1979)
Census (Indirect, 1980)
DHS (Direct, 1988)
Census (Indirect, 1990)
Others (Direct, 1992)
Census (Indirect, 2000)
Others (Indirect, 2005)
Others (Indirect, 2006)
Census (Indirect, 2010)
VR

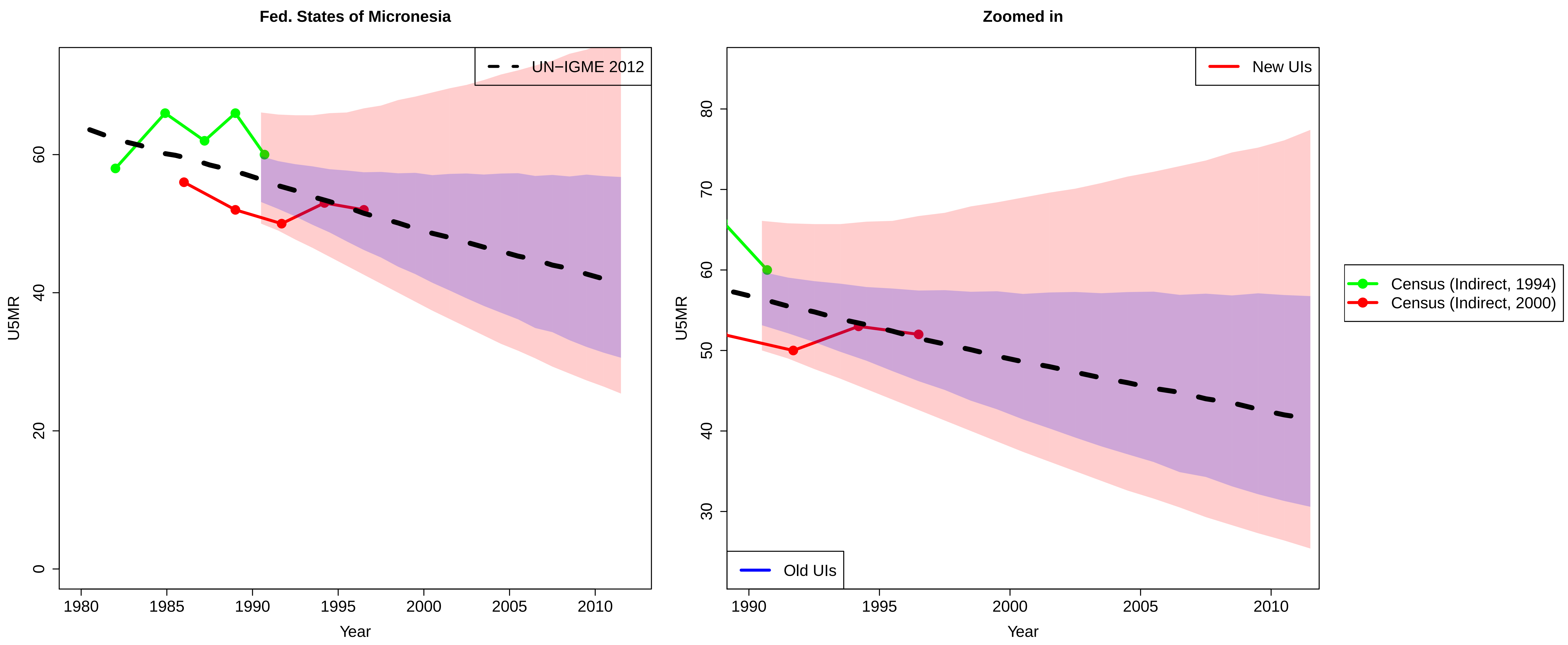
Fed. States of Micronesia
Zoomed in
UN−IGME 2012
New UIs
Old UIs
Census (Indirect, 1994)
Census (Indirect, 2000)
U5MR
Year
0
20
40
60
30
40
50
60
70
80
1980
1985
1990
1995
2000
2005
2010

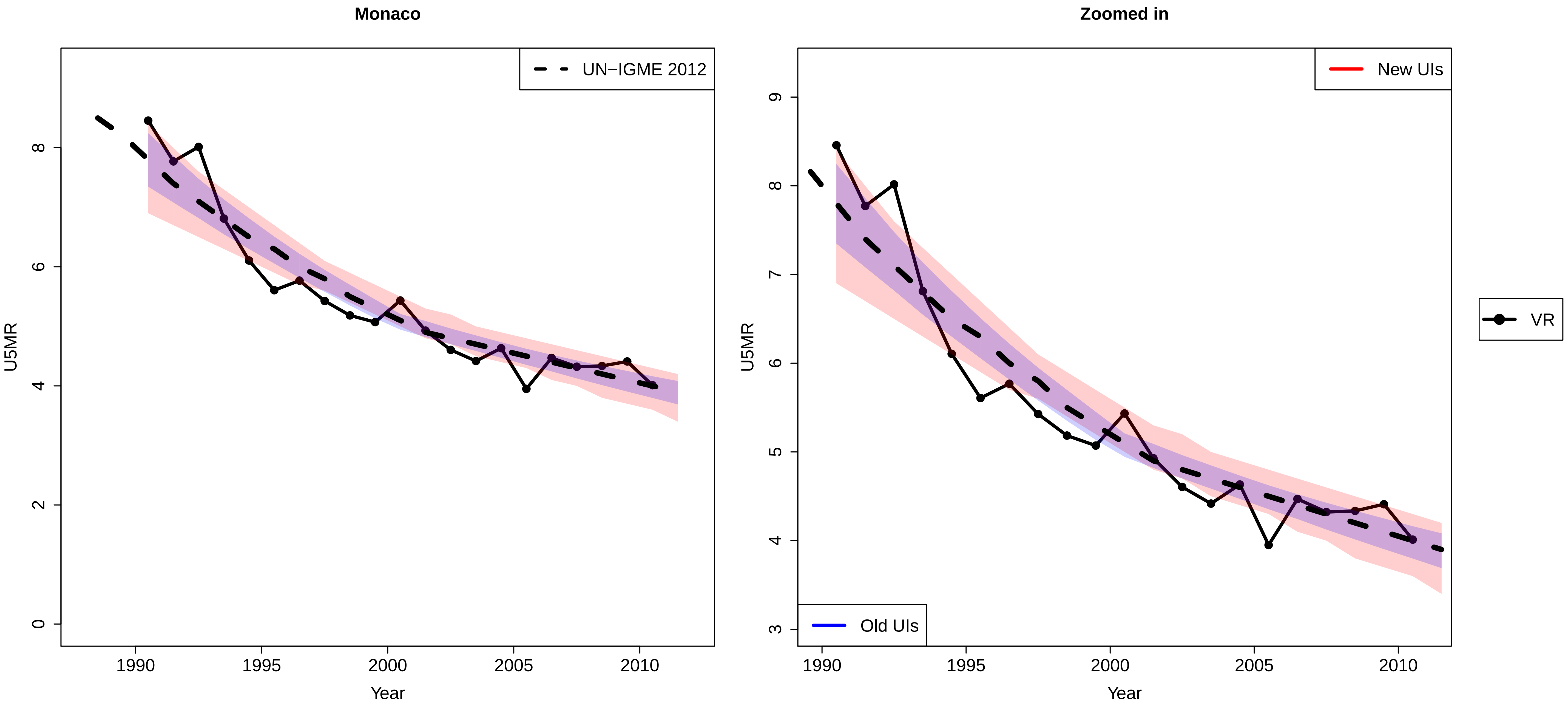
Monaco
UN−IGME 2012
U5MR
Year
Zoomed in
New UIs
Old UIs
VR

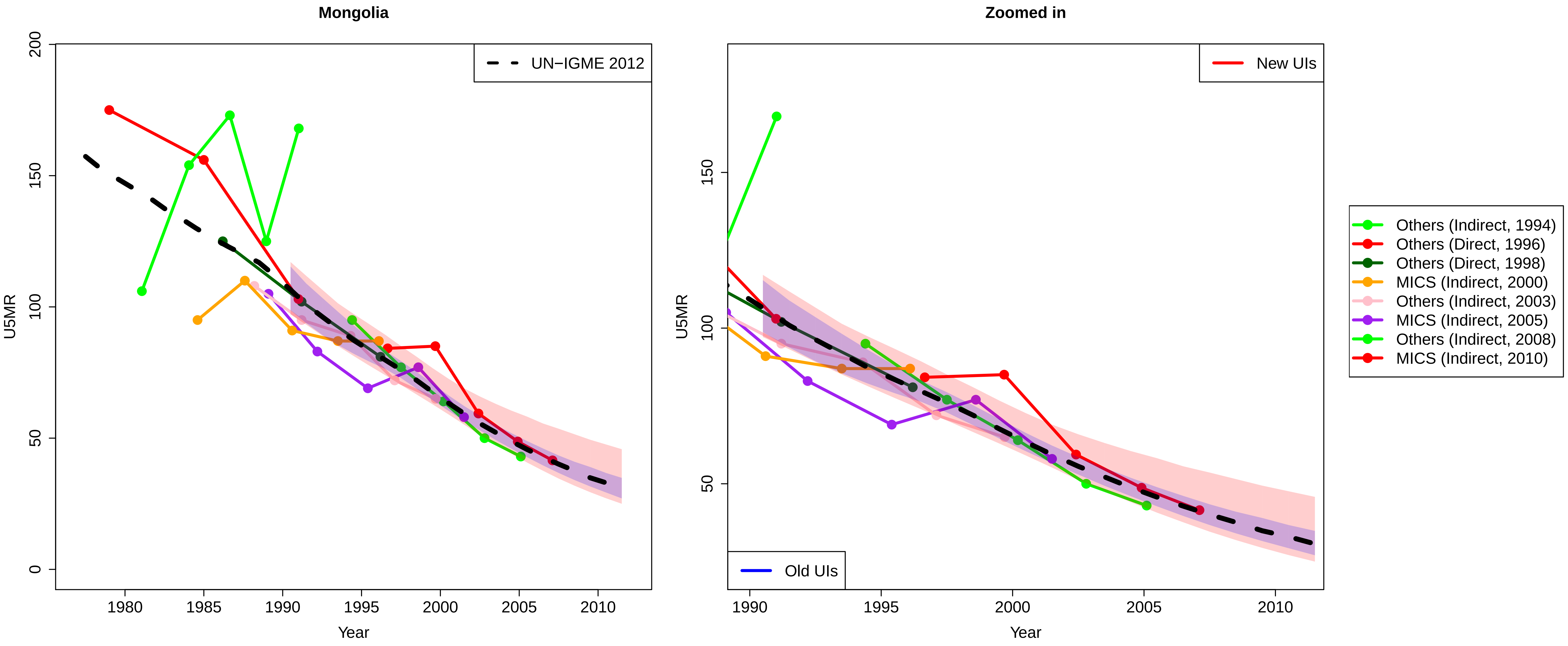

Mongolia
Zoomed in
UN−IGME 2012
New UIs
Old UIs
U5MR
Year
Others (Indirect, 1994)
Others (Direct, 1996)
Others (Direct, 1998)
MICS (Indirect, 2000)
Others (Indirect, 2003)
MICS (Indirect, 2005)
Others (Indirect, 2008)
MICS (Indirect, 2010)

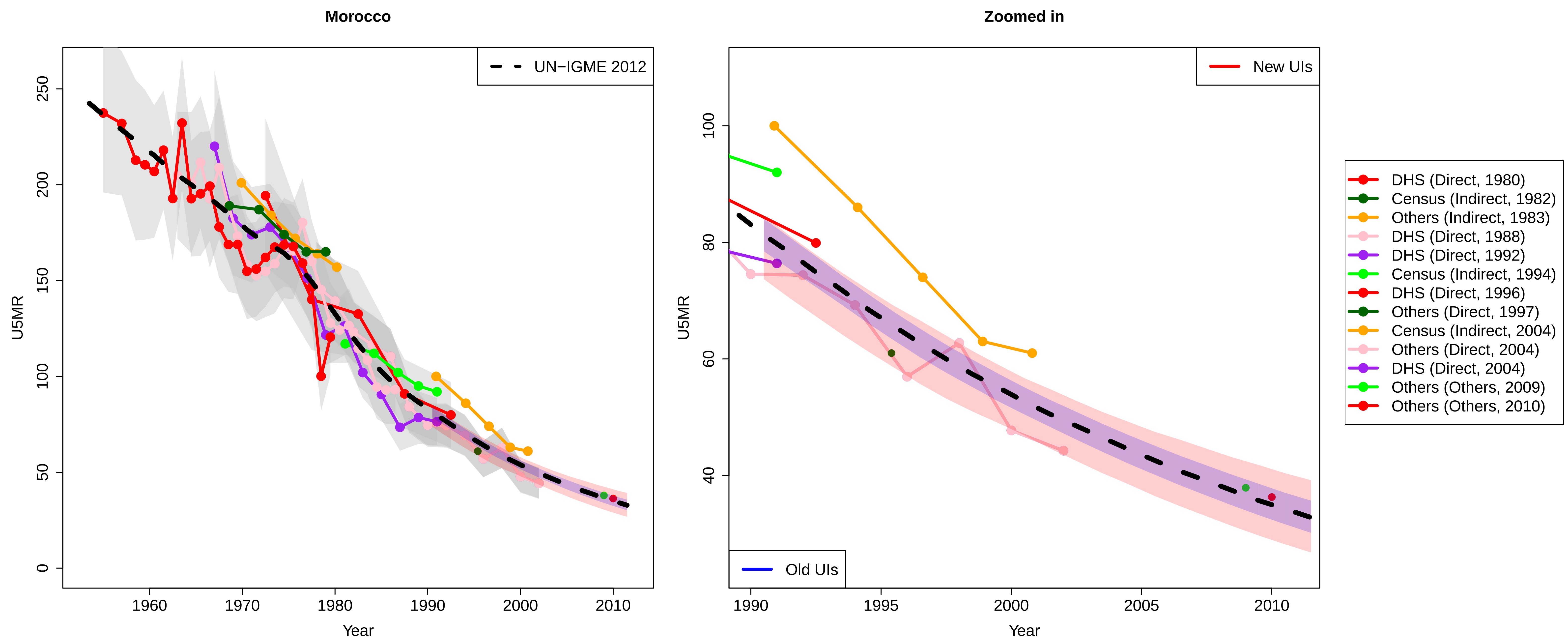

Morocco
UN−IGME 2012
U5MR
Year
0
50
100
150
200
250
1960
1970
1980
1990
2000
2010
Zoomed in
New UIs
Old UIs
40
60
80
100
1990
1995
2000
2005
2010
DHS (Direct, 1980)
Census (Indirect, 1982)
Others (Indirect, 1983)
DHS (Direct, 1988)
DHS (Direct, 1992)
Census (Indirect, 1994)
DHS (Direct, 1996)
Others (Direct, 1997)
Census (Indirect, 2004)
Others (Direct, 2004)
DHS (Direct, 2004)
Others (Others, 2009)
Others (Others, 2010)

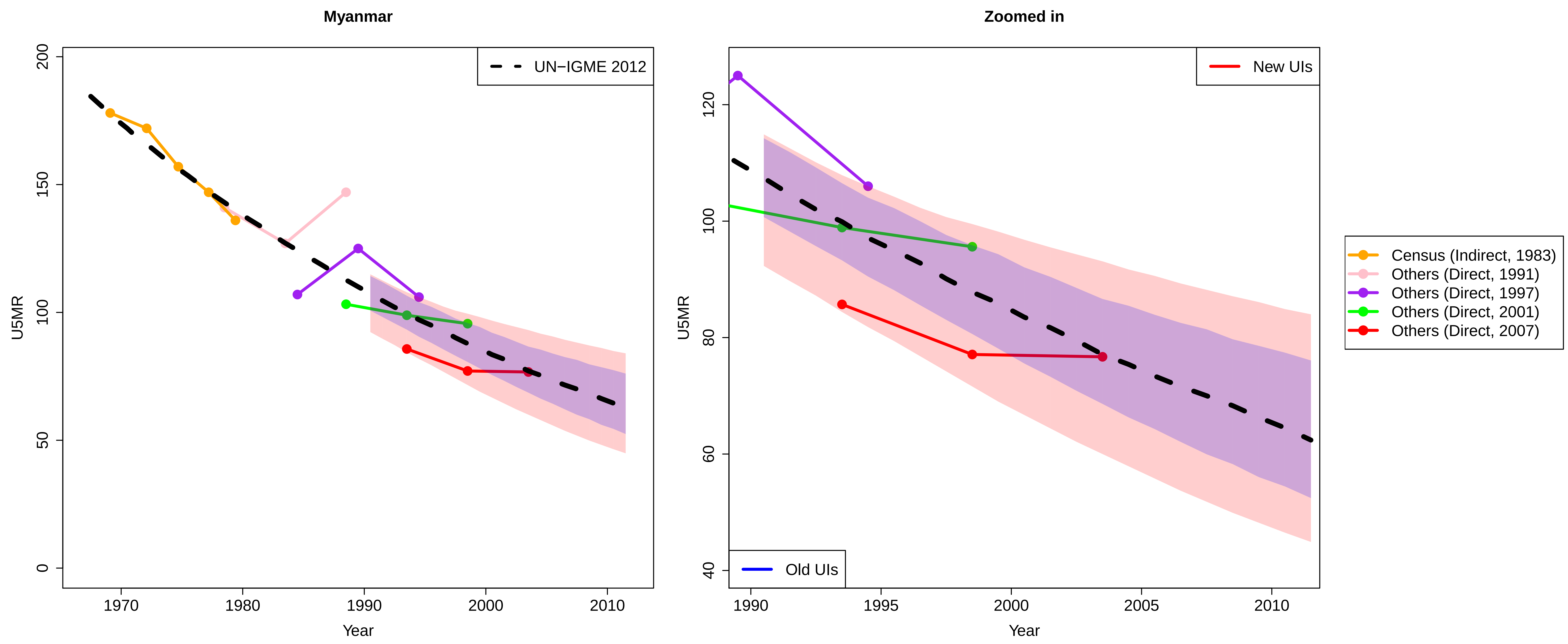

Myanmar
UN−IGME 2012
U5MR
Year
Zoomed in
New UIs
Old UIs
Census (Indirect, 1983)
Others (Direct, 1991)
Others (Direct, 1997)
Others (Direct, 2001)
Others (Direct, 2007)

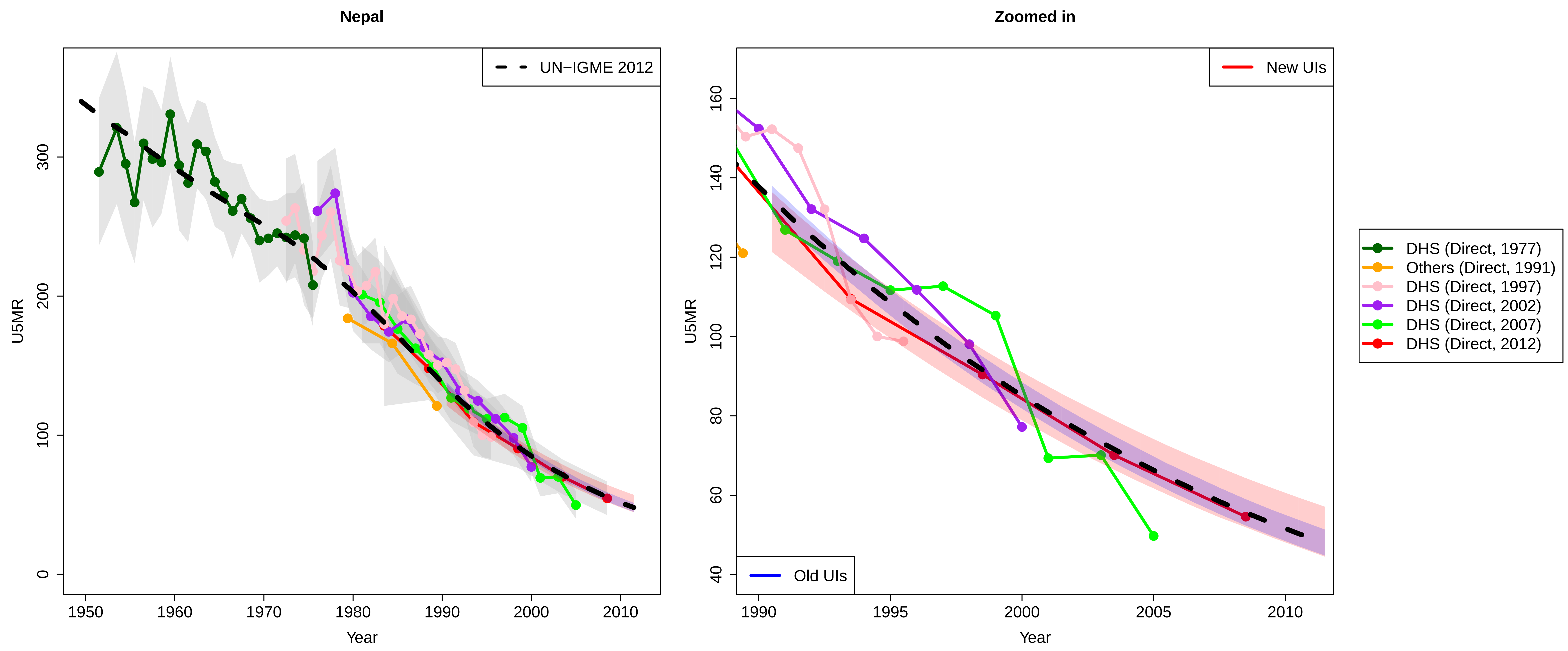
Nepal
Zoomed in
UN−IGME 2012
New UIs
Old UIs
DHS (Direct, 1977)
Others (Direct, 1991)
DHS (Direct, 1997)
DHS (Direct, 2002)
DHS (Direct, 2007)
DHS (Direct, 2012)
U5MR
Year
0
100
200
300
40
60
80
100
120
140
160
1950
1960
1970
1980
1990
2000
2010
1990
1995
2000
2005
2010

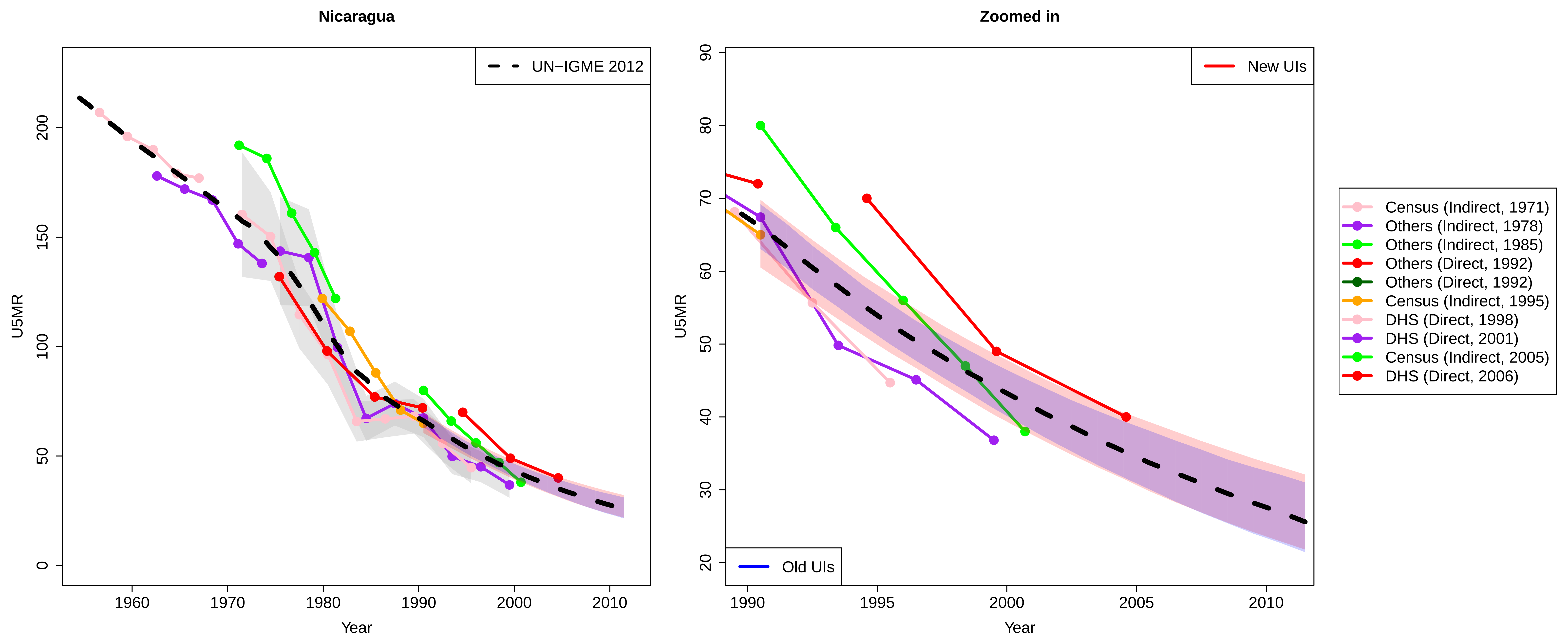

Nicaragua
UN−IGME 2012
U5MR
Year
Zoomed in
New UIs
Old UIs
Census (Indirect, 1971)
Others (Indirect, 1978)
Others (Indirect, 1985)
Others (Direct, 1992)
Others (Direct, 1992)
Census (Indirect, 1995)
DHS (Direct, 1998)
DHS (Direct, 2001)
Census (Indirect, 2005)
DHS (Direct, 2006)

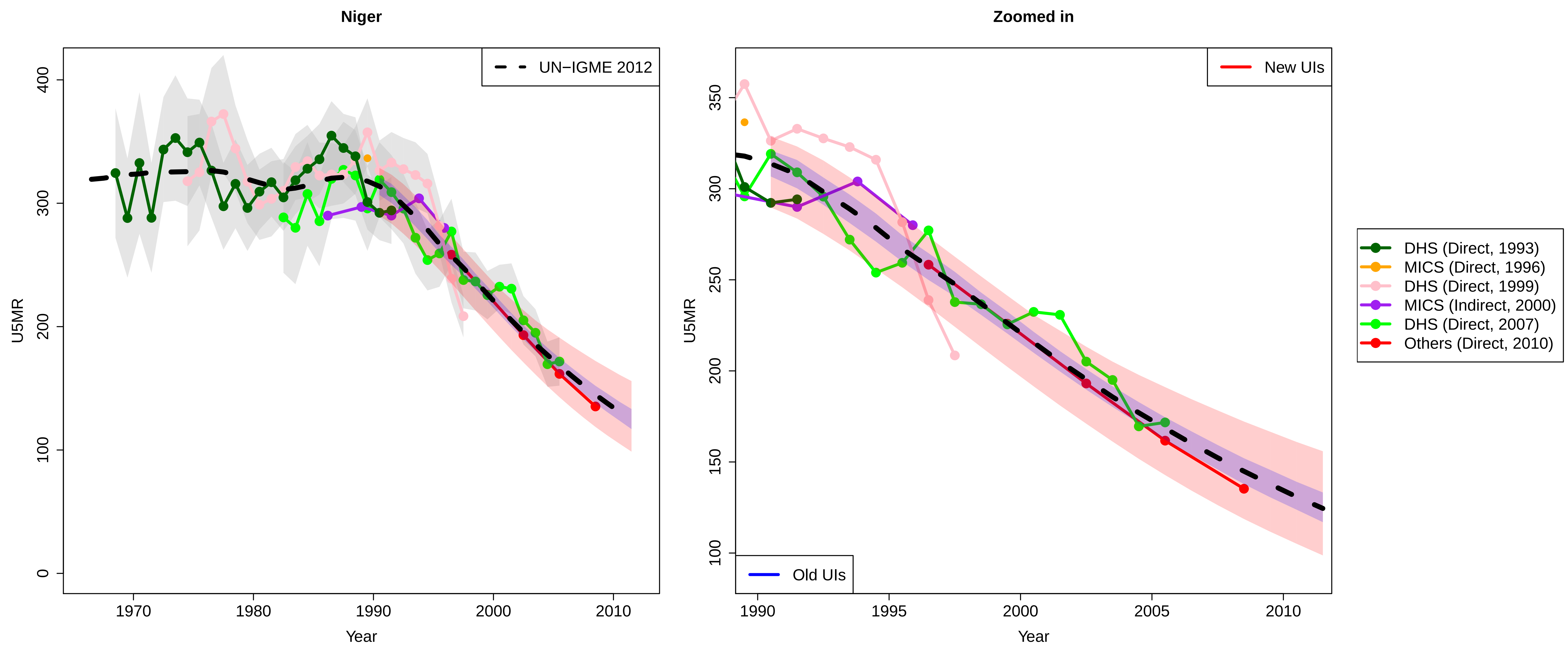
Niger
UN−IGME 2012
U5MR
Year
0
100
200
300
400
1970
1980
1990
2000
2010
Zoomed in
New UIs
Old UIs
100
150
200
250
300
350
1990
1995
2000
2005
2010
DHS (Direct, 1993)
MICS (Direct, 1996)
DHS (Direct, 1999)
MICS (Indirect, 2000)
DHS (Direct, 2007)
Others (Direct, 2010)

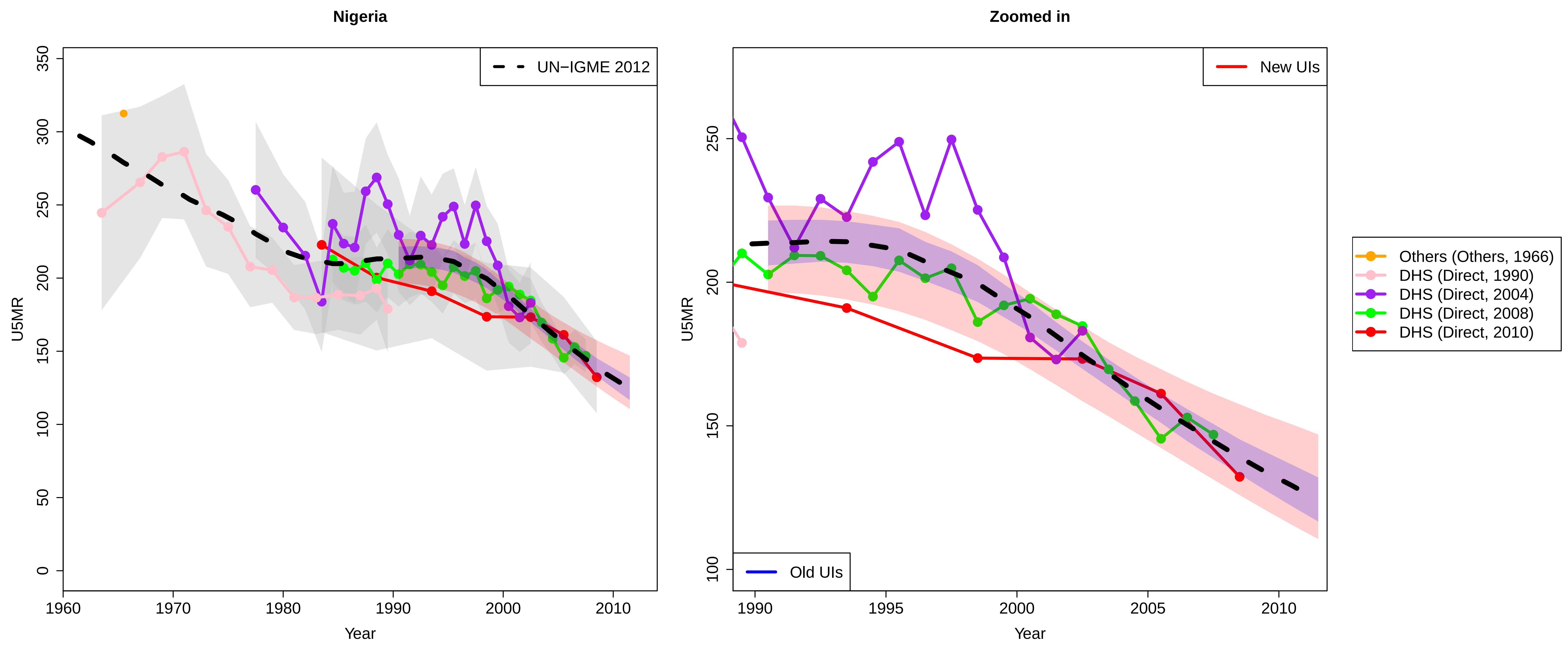

Nigeria
Zoomed in
UN−IGME 2012
New UIs
Old UIs
U5MR
Year
Others (Others, 1966)
DHS (Direct, 1990)
DHS (Direct, 2004)
DHS (Direct, 2008)
DHS (Direct, 2010)

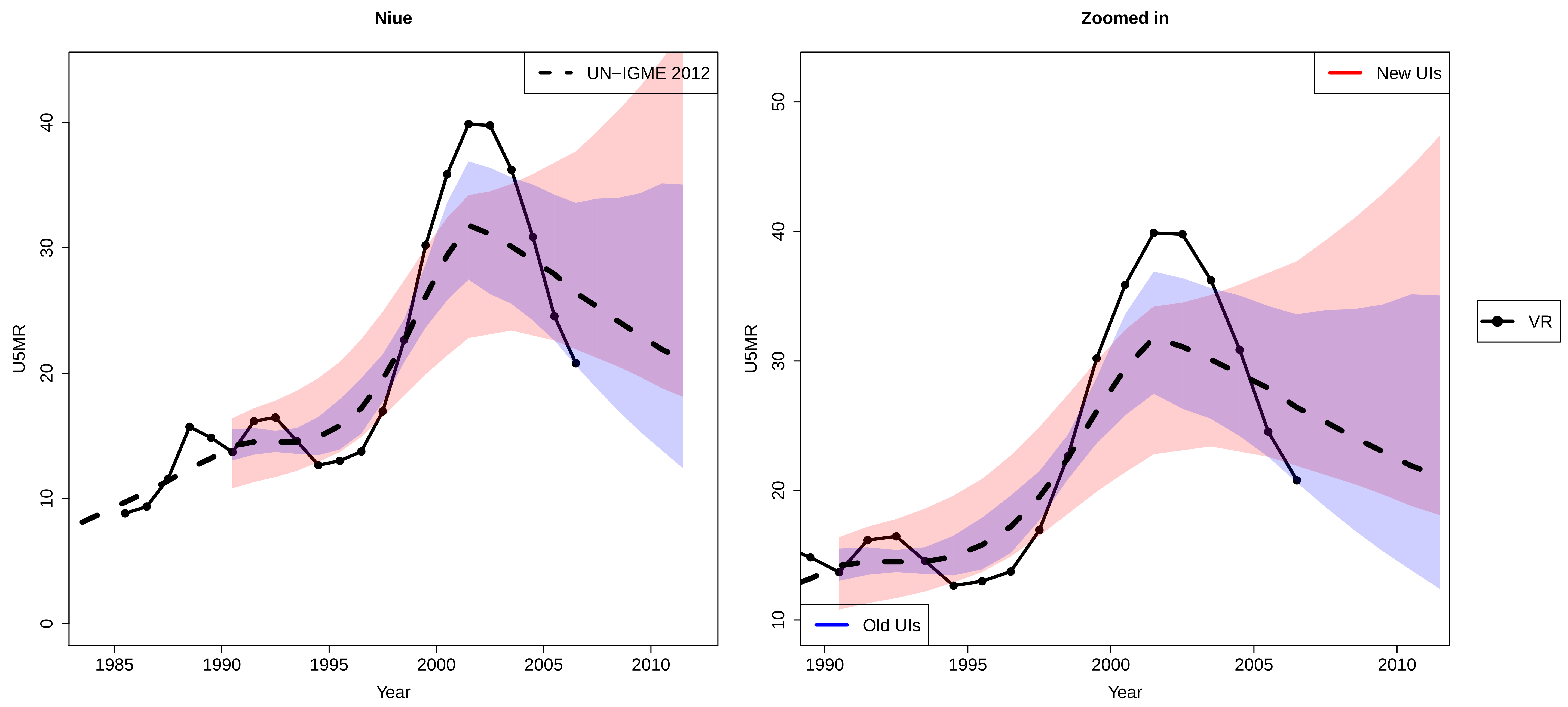

Niue
UN−IGME 2012
U5MR
Year
Zoomed in
New UIs
Old UIs
VR

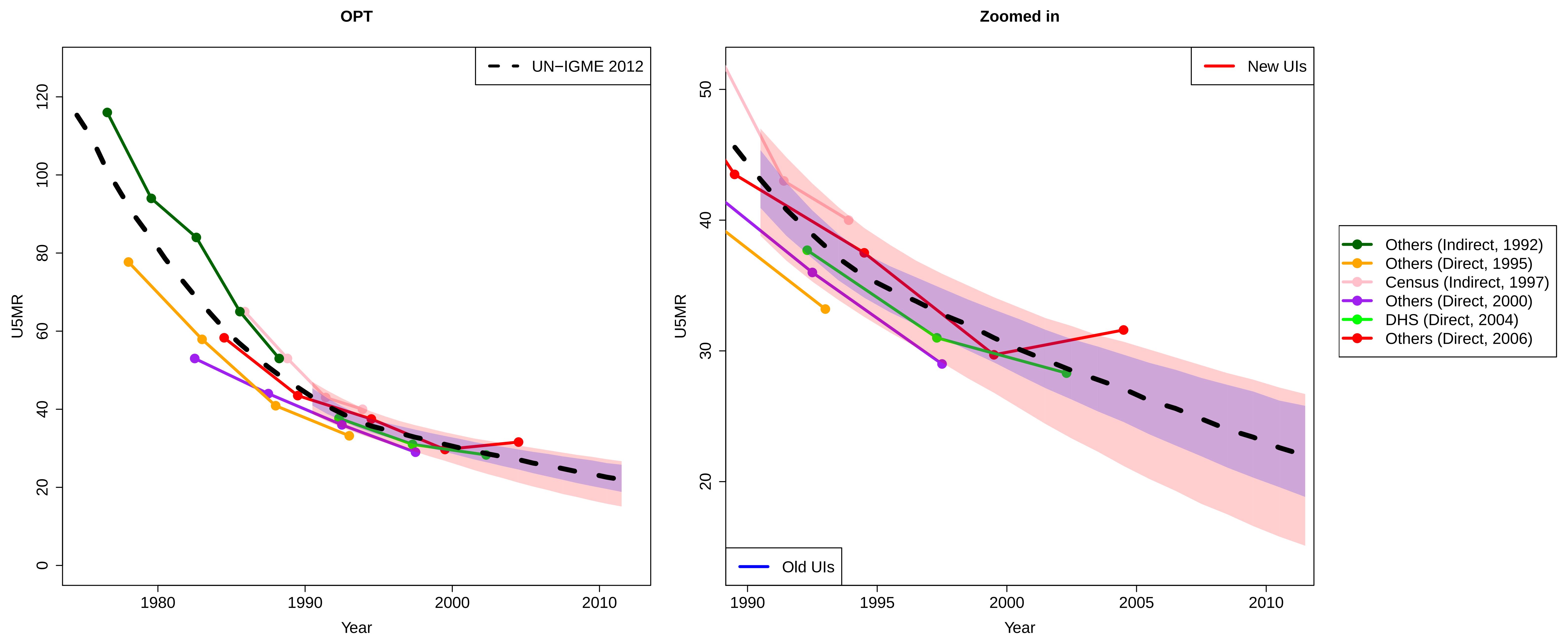

OPT
UN−IGME 2012
U5MR
Year
Zoomed in
New UIs
Old UIs
Others (Indirect, 1992)
Others (Direct, 1995)
Census (Indirect, 1997)
Others (Direct, 2000)
DHS (Direct, 2004)
Others (Direct, 2006)

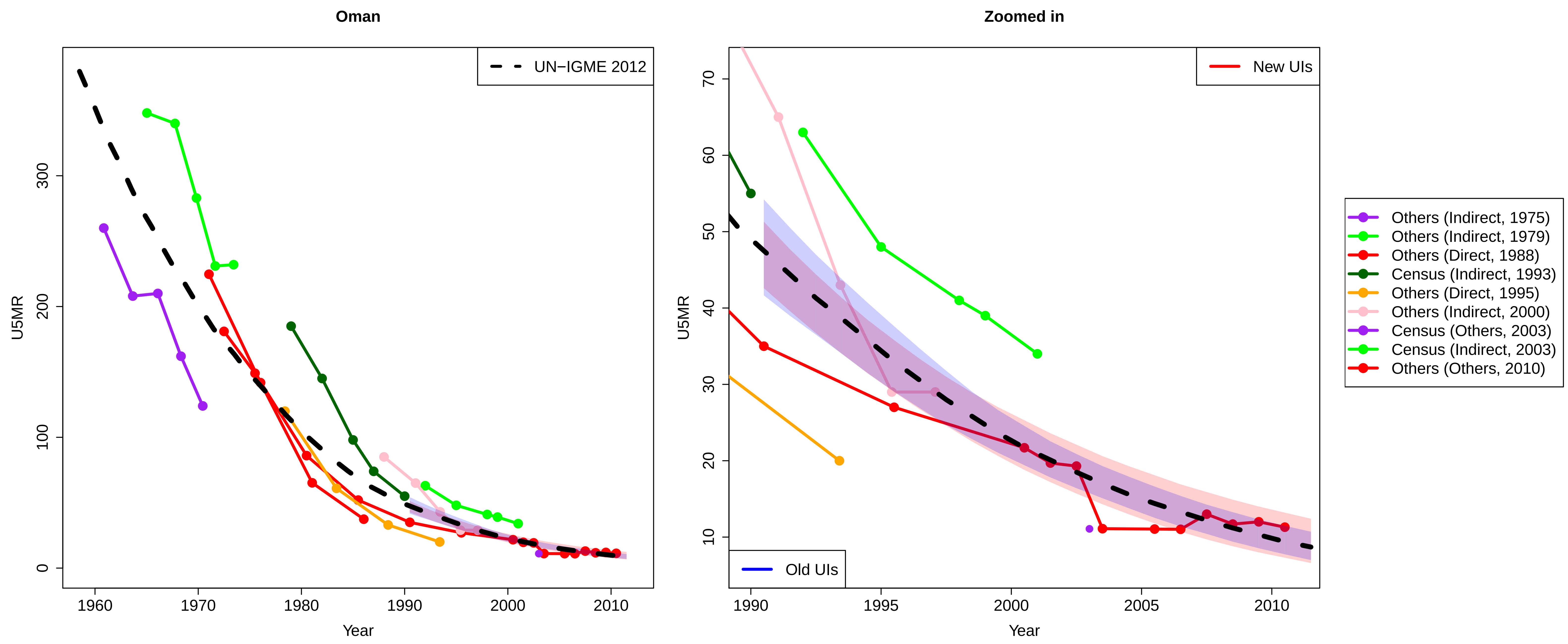
Oman
UN−IGME 2012
U5MR
Year
Zoomed in
New UIs
Old UIs
Others (Indirect, 1975)
Others (Indirect, 1979)
Others (Direct, 1988)
Census (Indirect, 1993)
Others (Direct, 1995)
Others (Indirect, 2000)
Census (Others, 2003)
Census (Indirect, 2003)
Others (Others, 2010)

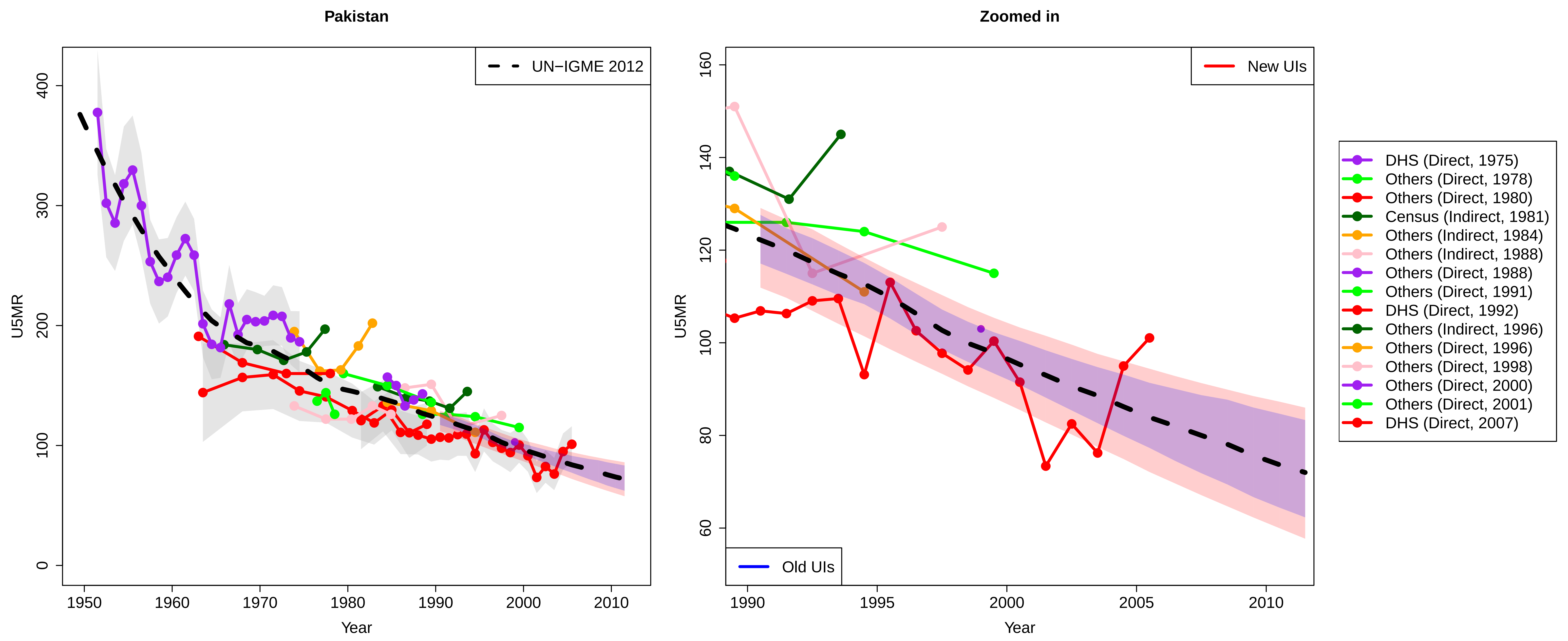

Pakistan
Zoomed in
UN−IGME 2012
New UIs
Old UIs
U5MR
Year
DHS (Direct, 1975)
Others (Direct, 1978)
Others (Direct, 1980)
Census (Indirect, 1981)
Others (Indirect, 1984)
Others (Indirect, 1988)
Others (Direct, 1988)
Others (Direct, 1991)
DHS (Direct, 1992)
Others (Indirect, 1996)
Others (Direct, 1996)
Others (Direct, 1998)
Others (Direct, 2000)
Others (Direct, 2001)
DHS (Direct, 2007)

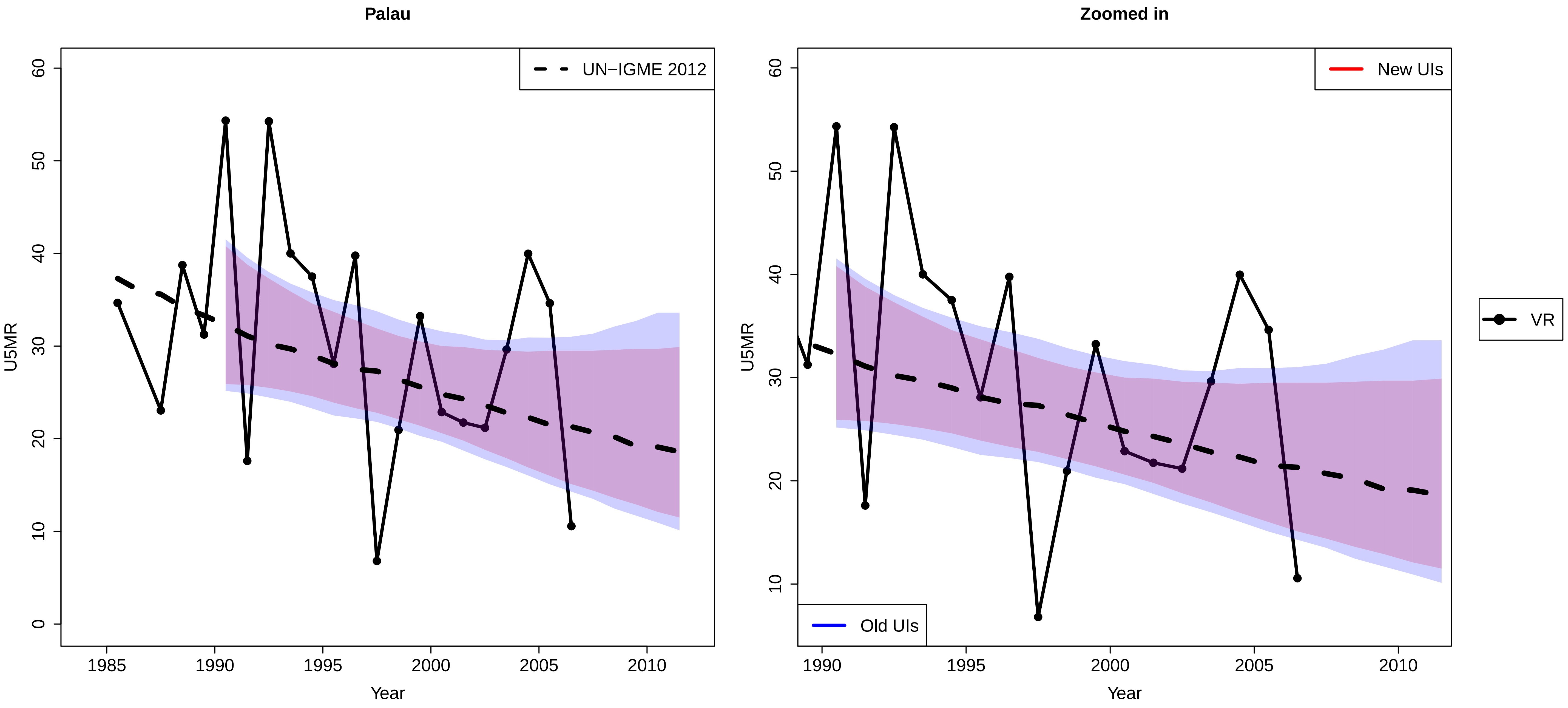
Palau
UN−IGME 2012
U5MR
Year
Zoomed in
New UIs
Old UIs
VR

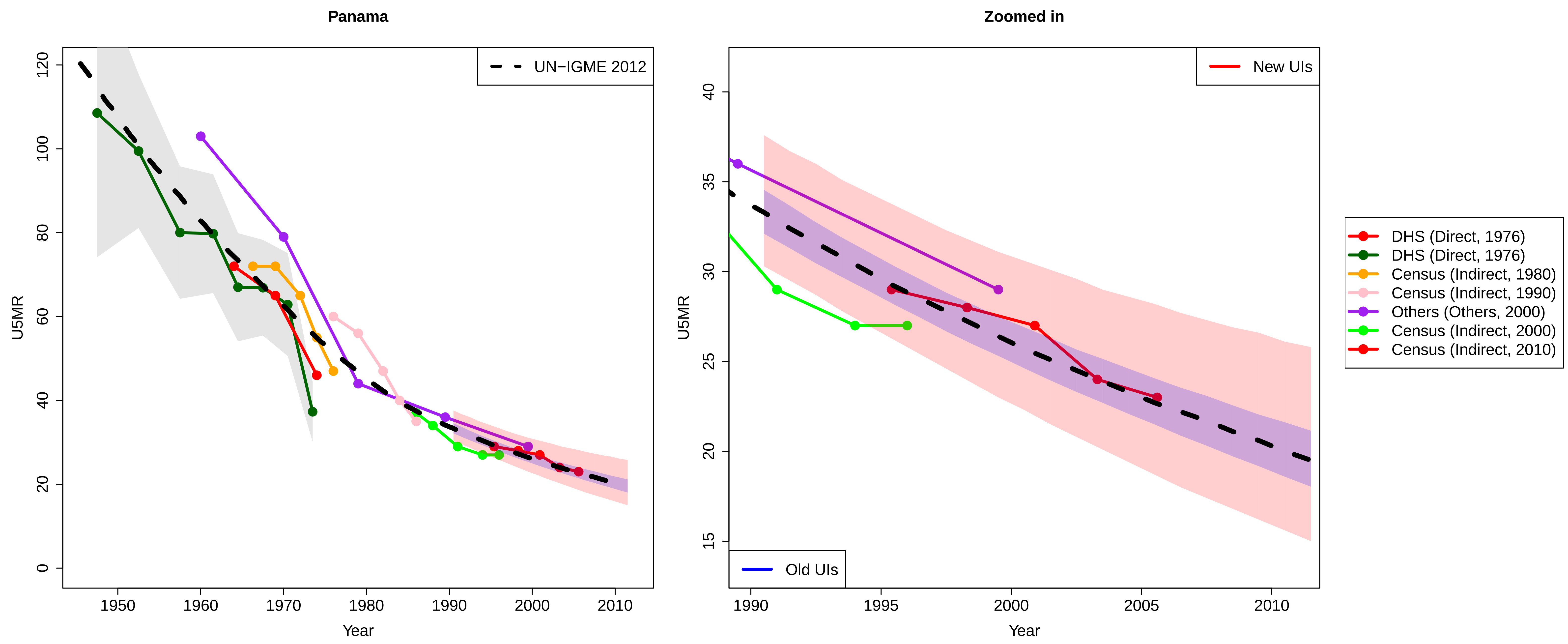

Panama
UN−IGME 2012
U5MR
Year
Zoomed in
New UIs
Old UIs
DHS (Direct, 1976)
DHS (Direct, 1976)
Census (Indirect, 1980)
Census (Indirect, 1990)
Others (Others, 2000)
Census (Indirect, 2000)
Census (Indirect, 2010)

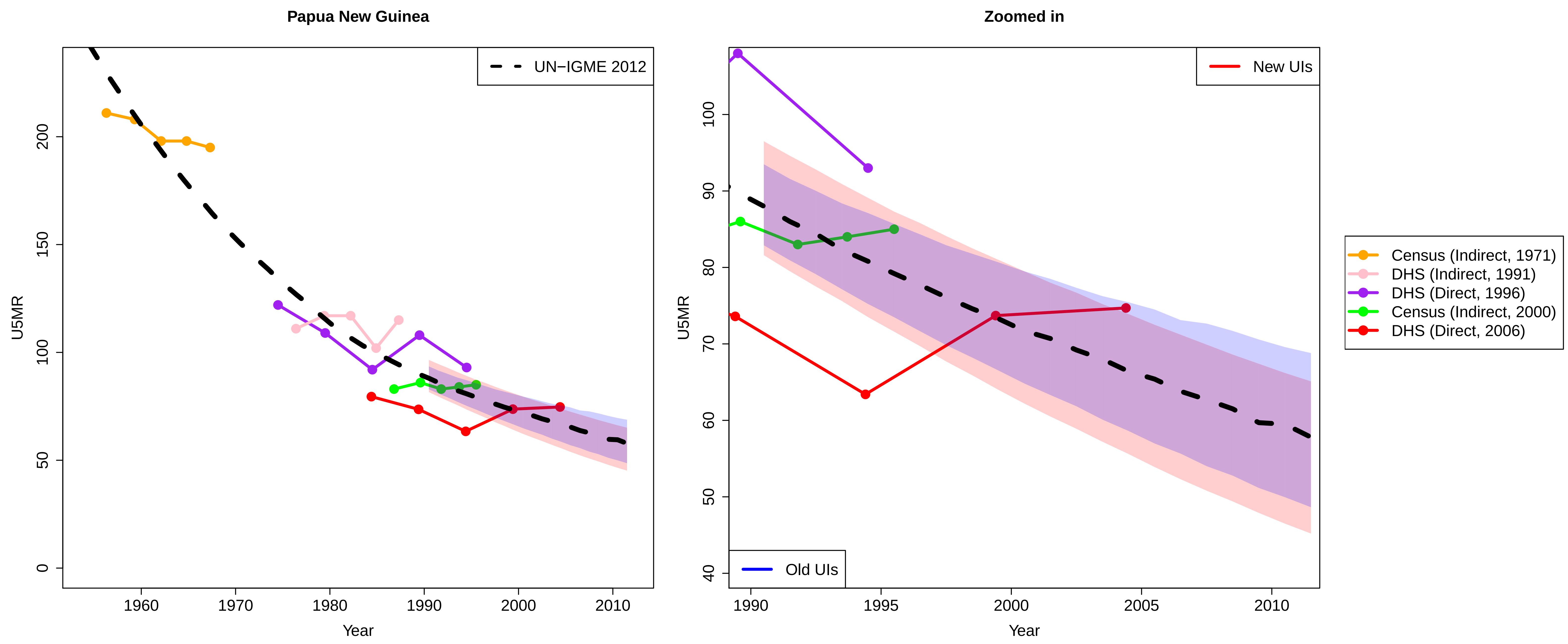

Papua New Guinea
Zoomed in
UN−IGME 2012
New UIs
Old UIs
U5MR
Year
Census (Indirect, 1971)
DHS (Indirect, 1991)
DHS (Direct, 1996)
Census (Indirect, 2000)
DHS (Direct, 2006)

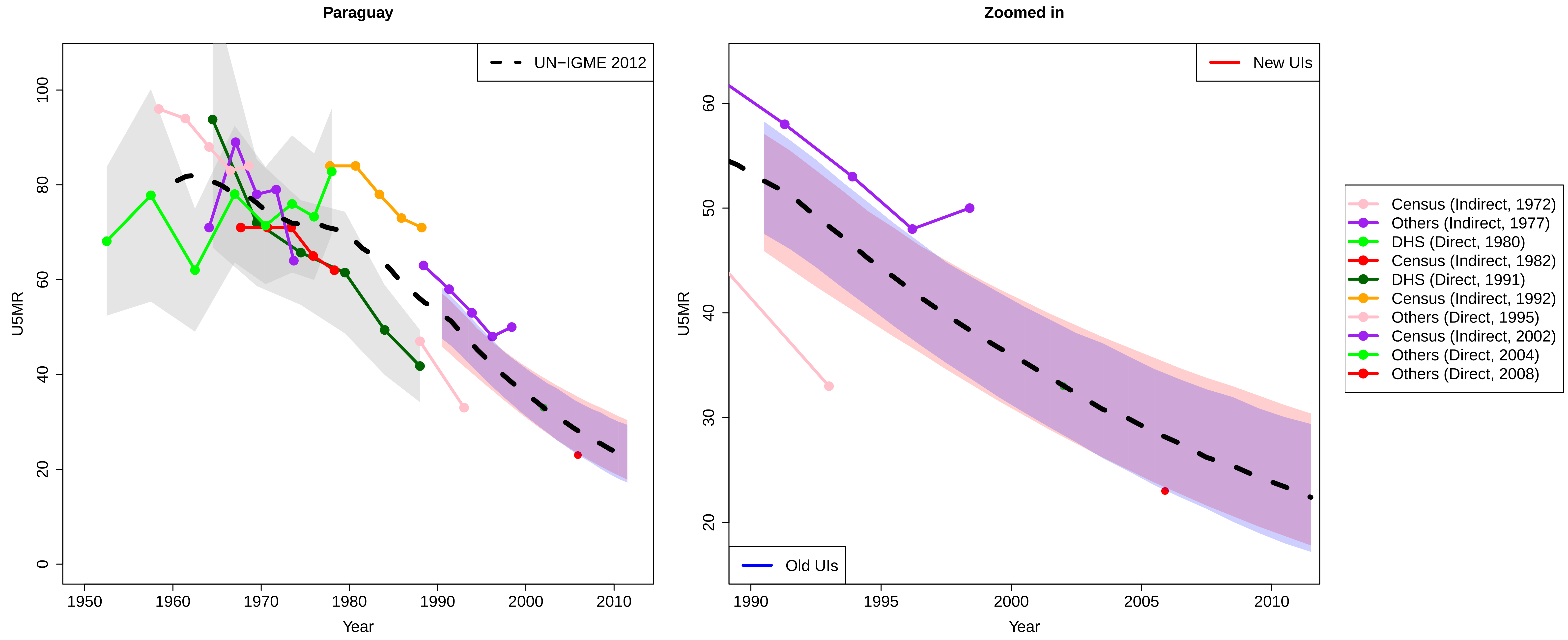

Paraguay
Zoomed in
UN−IGME 2012
New UIs
Old UIs
U5MR
Year
Census (Indirect, 1972)
Others (Indirect, 1977)
DHS (Direct, 1980)
Census (Indirect, 1982)
DHS (Direct, 1991)
Census (Indirect, 1992)
Others (Direct, 1995)
Census (Indirect, 2002)
Others (Direct, 2004)
Others (Direct, 2008)

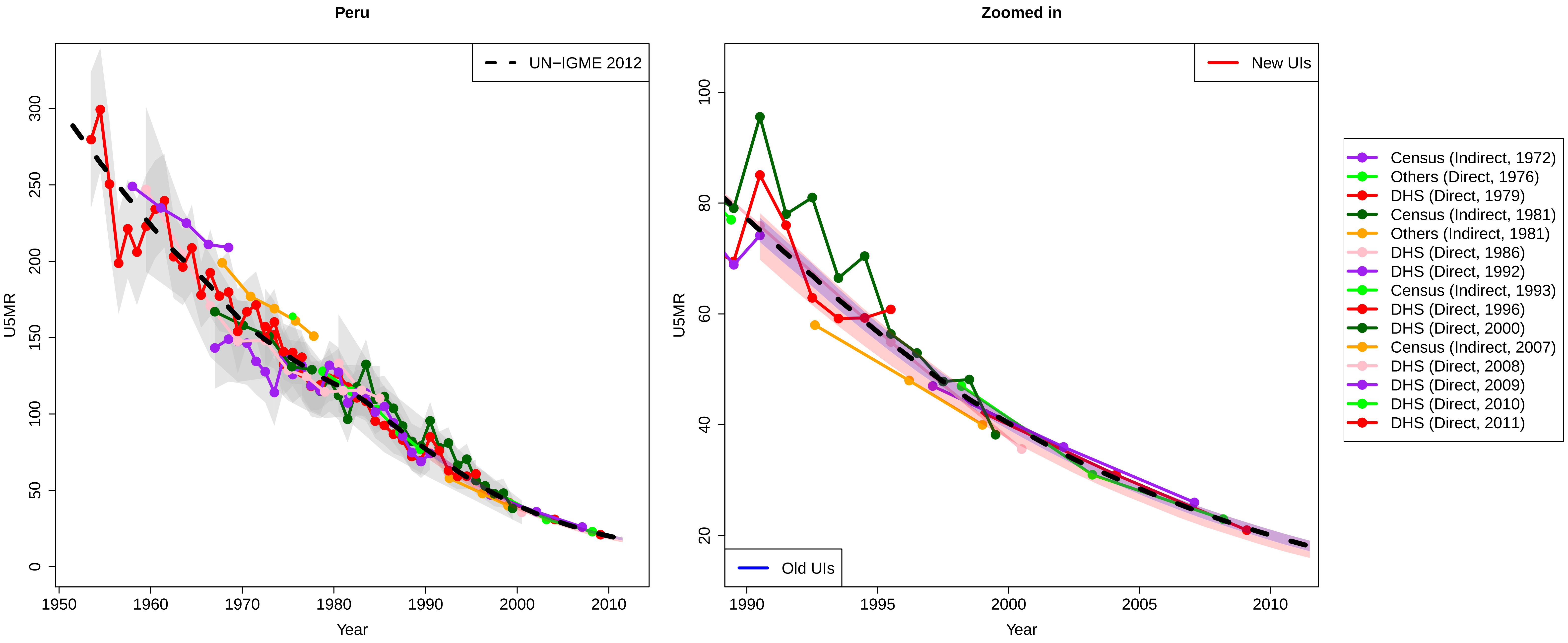

Peru
Zoomed in
UN−IGME 2012
New UIs
Old UIs
U5MR
Year
Census (Indirect, 1972)
Others (Direct, 1976)
DHS (Direct, 1979)
Census (Indirect, 1981)
Others (Indirect, 1981)
DHS (Direct, 1986)
DHS (Direct, 1992)
Census (Indirect, 1993)
DHS (Direct, 1996)
DHS (Direct, 2000)
Census (Indirect, 2007)
DHS (Direct, 2008)
DHS (Direct, 2009)
DHS (Direct, 2010)
DHS (Direct, 2011)

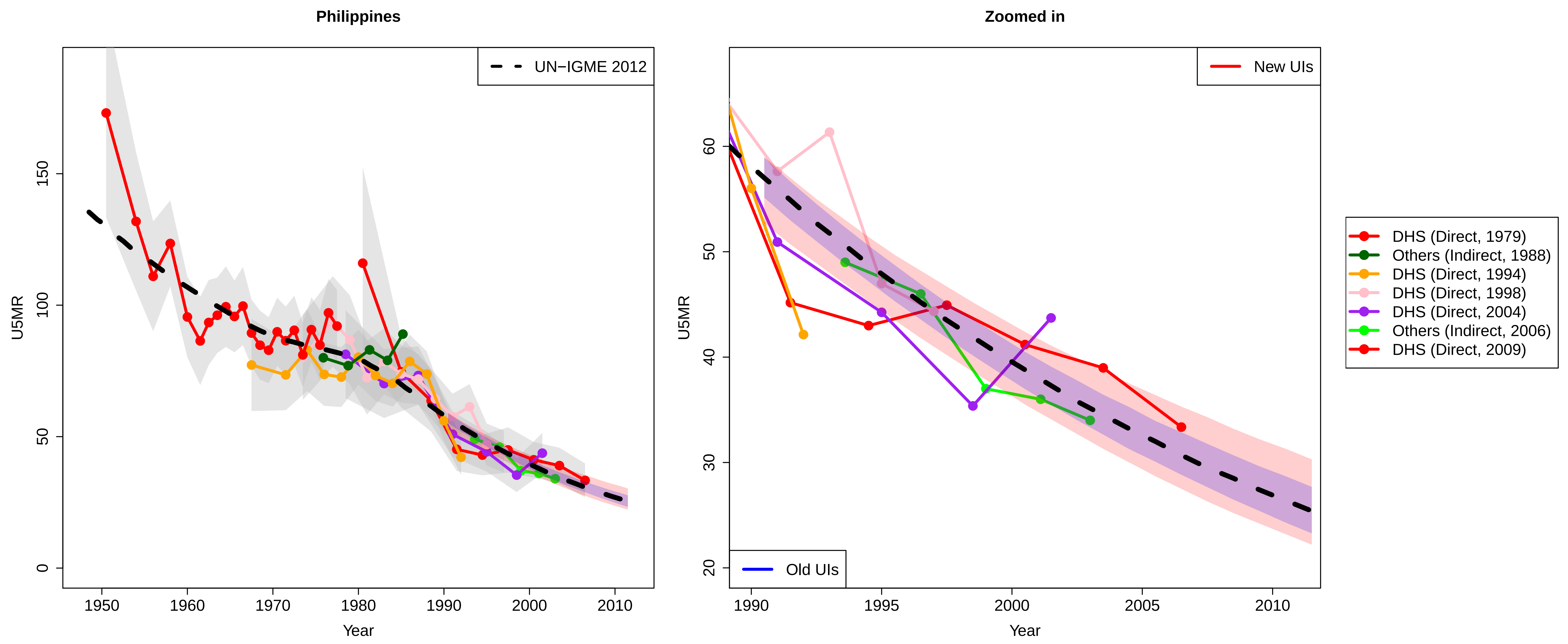
Philippines
UN−IGME 2012
U5MR
Year
Zoomed in
New UIs
Old UIs
DHS (Direct, 1979)
Others (Indirect, 1988)
DHS (Direct, 1994)
DHS (Direct, 1998)
DHS (Direct, 2004)
Others (Indirect, 2006)
DHS (Direct, 2009)

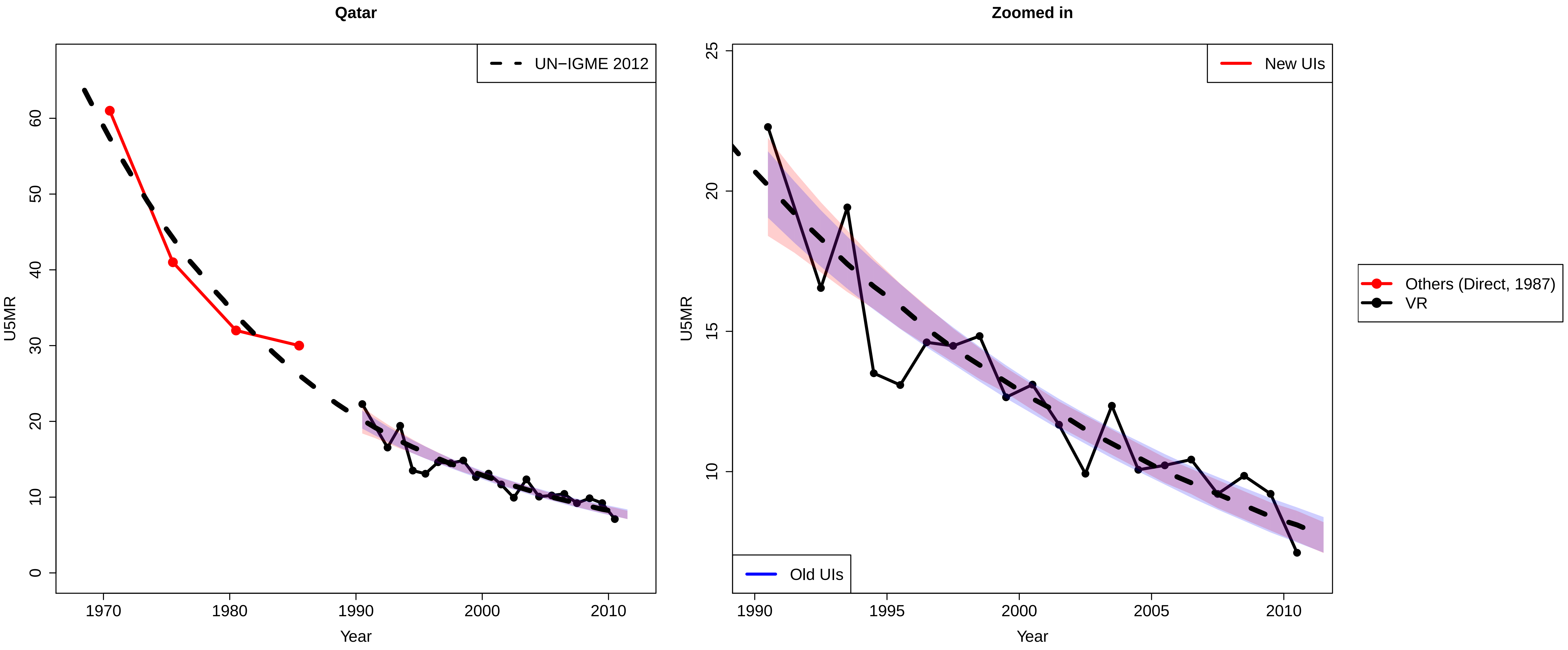
Qatar
Zoomed in
UN−IGME 2012
New UIs
Old UIs
Others (Direct, 1987)
VR
U5MR
Year
0
10
20
30
40
50
60
1970
1980
1990
2000
2010
25
20
15
10
1990
1995
2000
2005
2010

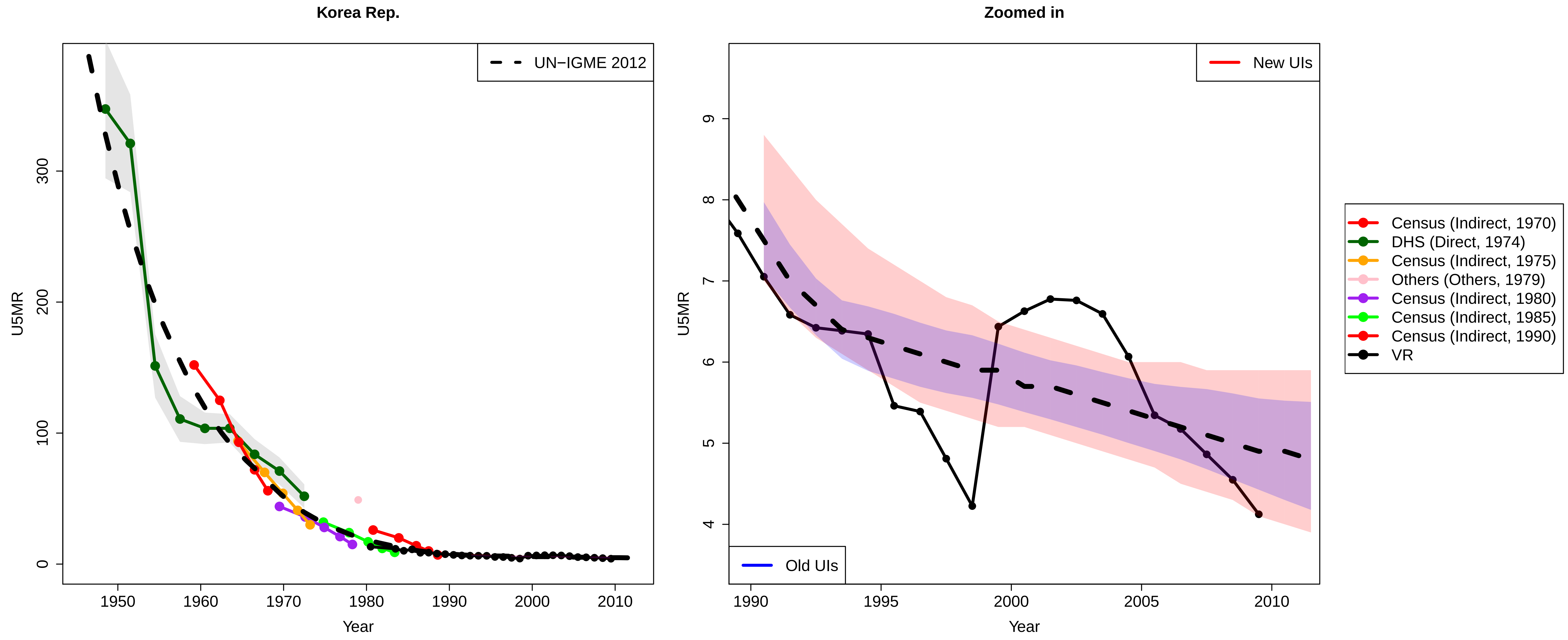

Korea Rep.
UN−IGME 2012
U5MR
Year
Zoomed in
New UIs
Old UIs
U5MR
Year
Census (Indirect, 1970)
DHS (Direct, 1974)
Census (Indirect, 1975)
Others (Others, 1979)
Census (Indirect, 1980)
Census (Indirect, 1985)
Census (Indirect, 1990)
VR

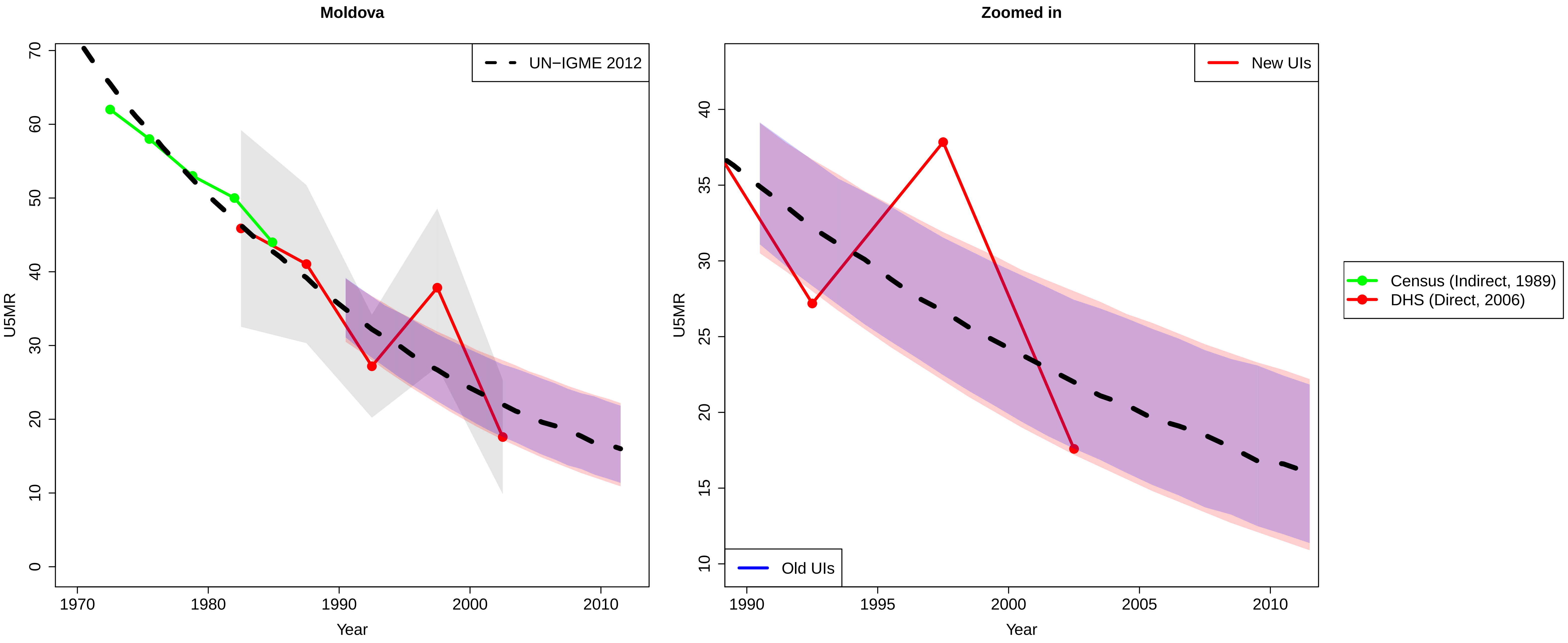
Moldova
Zoomed in
UN–IGME 2012
New UIs
Old UIs
Census (Indirect, 1989)
DHS (Direct, 2006)
U5MR
Year
0
10
20
30
40
50
60
70
1970
1980
1990
2000
2010
15
25
35
1995
2005

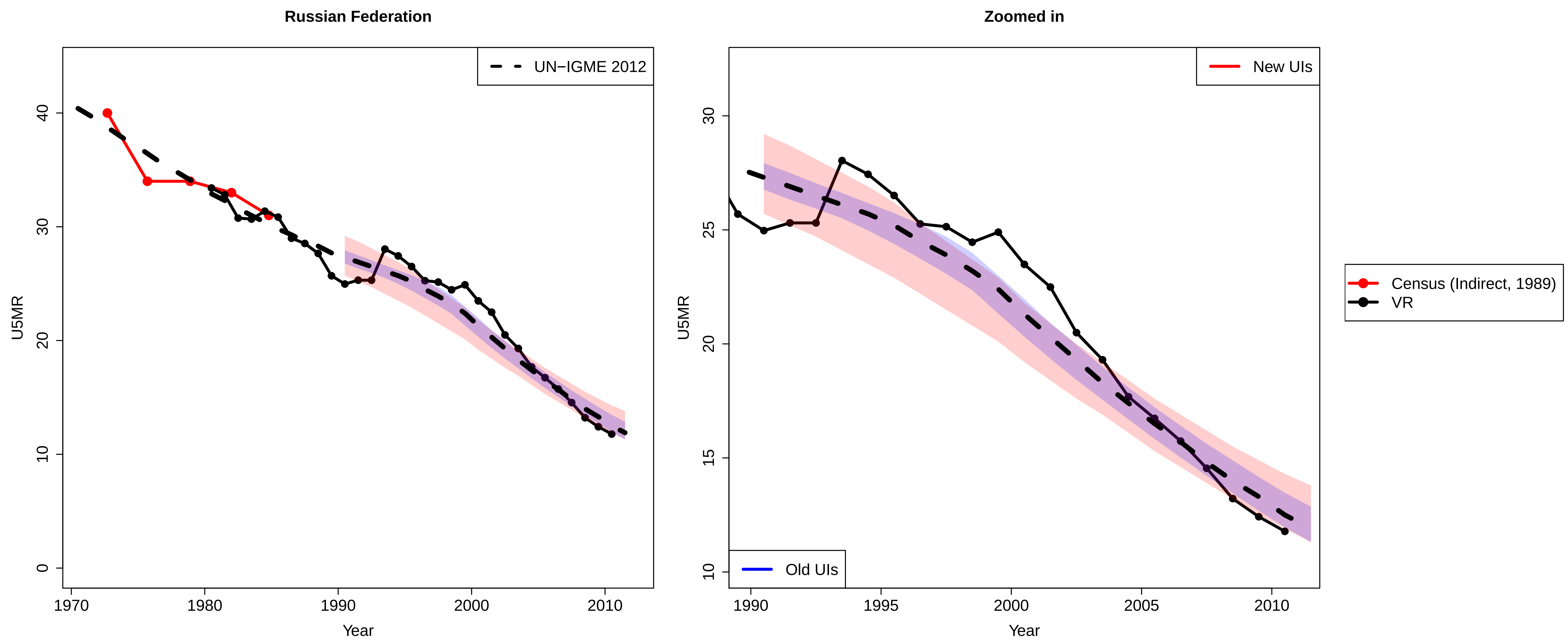
Russian Federation
Zoomed in
UN−IGME 2012
New UIs
Old UIs
Census (Indirect, 1989)
VR
U5MR
Year
0
10
20
30
40
15
25
1970
1980
1990
1995
2000
2005
2010

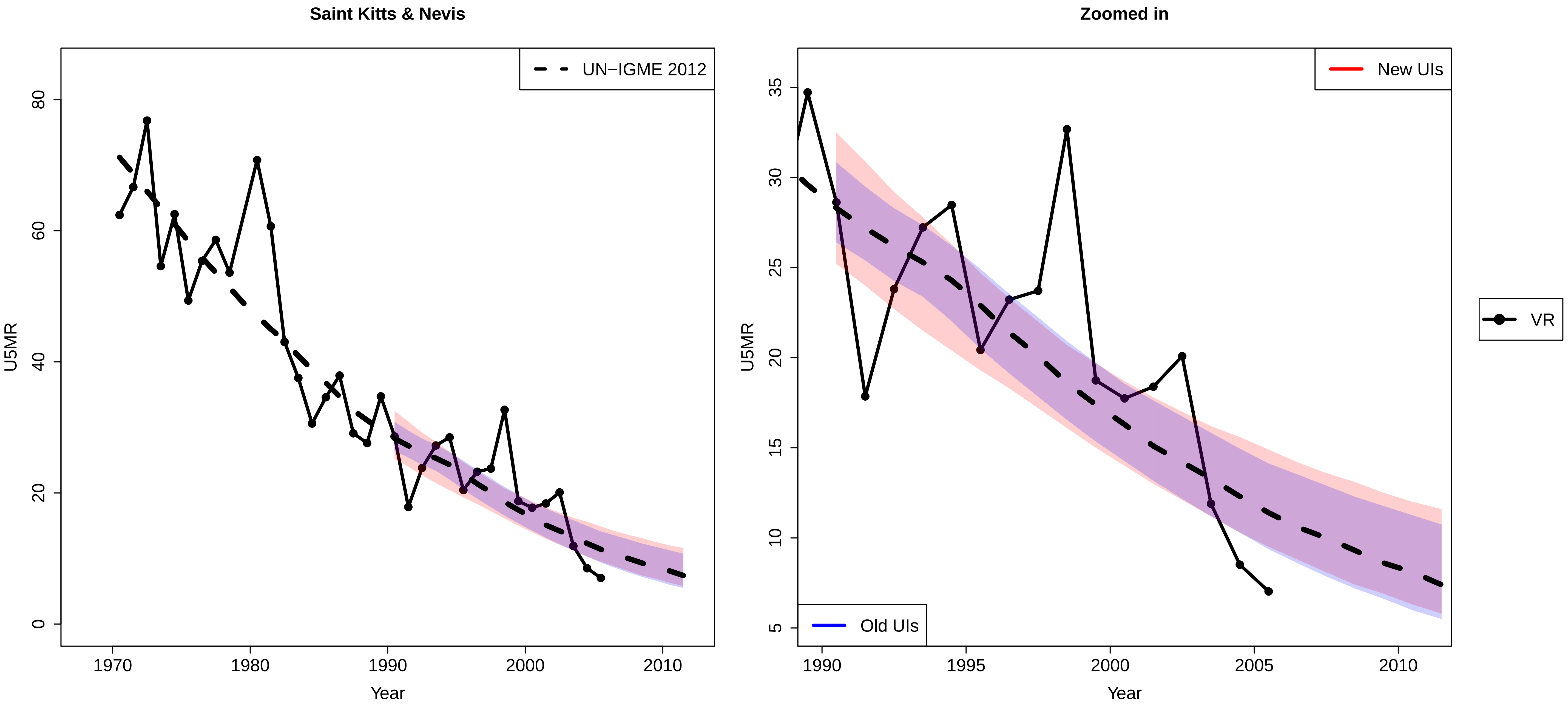
Saint Kitts & Nevis
UN−IGME 2012
U5MR
Year
Zoomed in
New UIs
Old UIs
VR

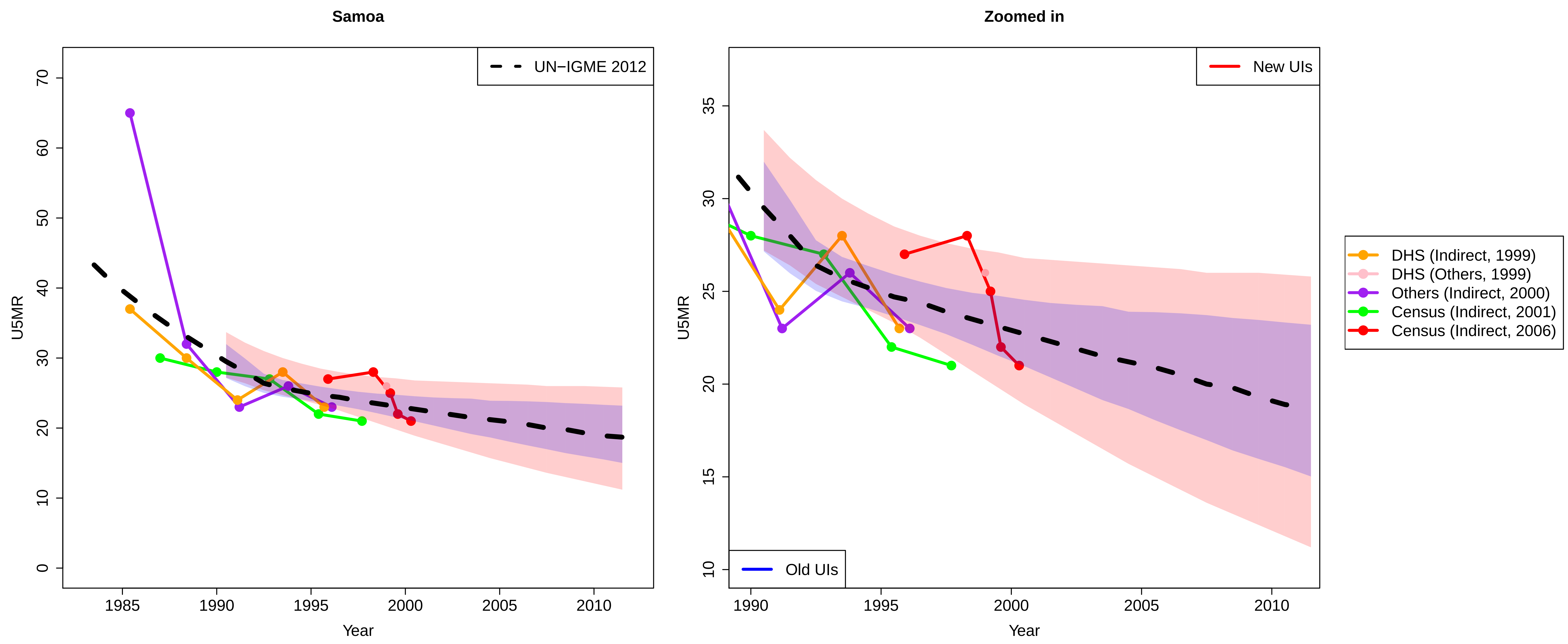

Samoa
Zoomed in
UN–IGME 2012
New UIs
Old UIs
DHS (Indirect, 1999)
DHS (Others, 1999)
Others (Indirect, 2000)
Census (Indirect, 2001)
Census (Indirect, 2006)
U5MR
Year

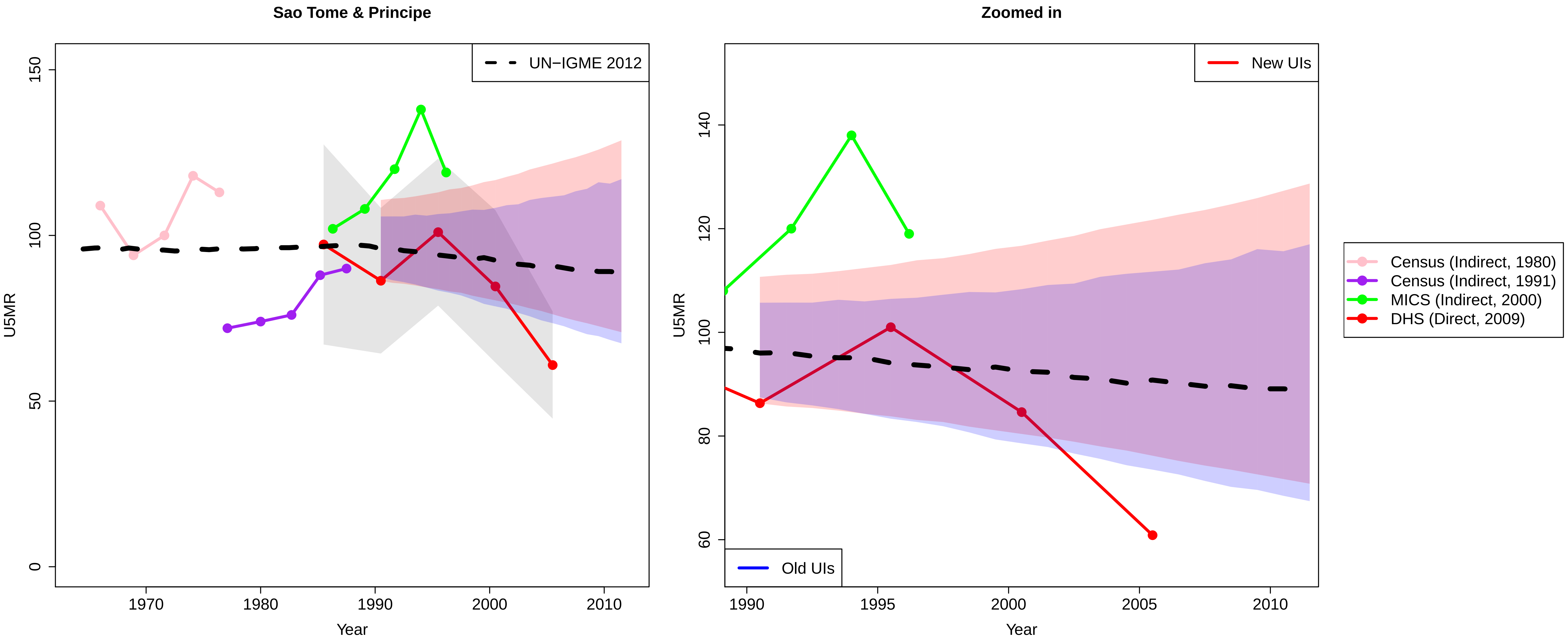

Sao Tome & Principe
UN−IGME 2012
U5MR
Year
Zoomed in
New UIs
Old UIs
Census (Indirect, 1980)
Census (Indirect, 1991)
MICS (Indirect, 2000)
DHS (Direct, 2009)

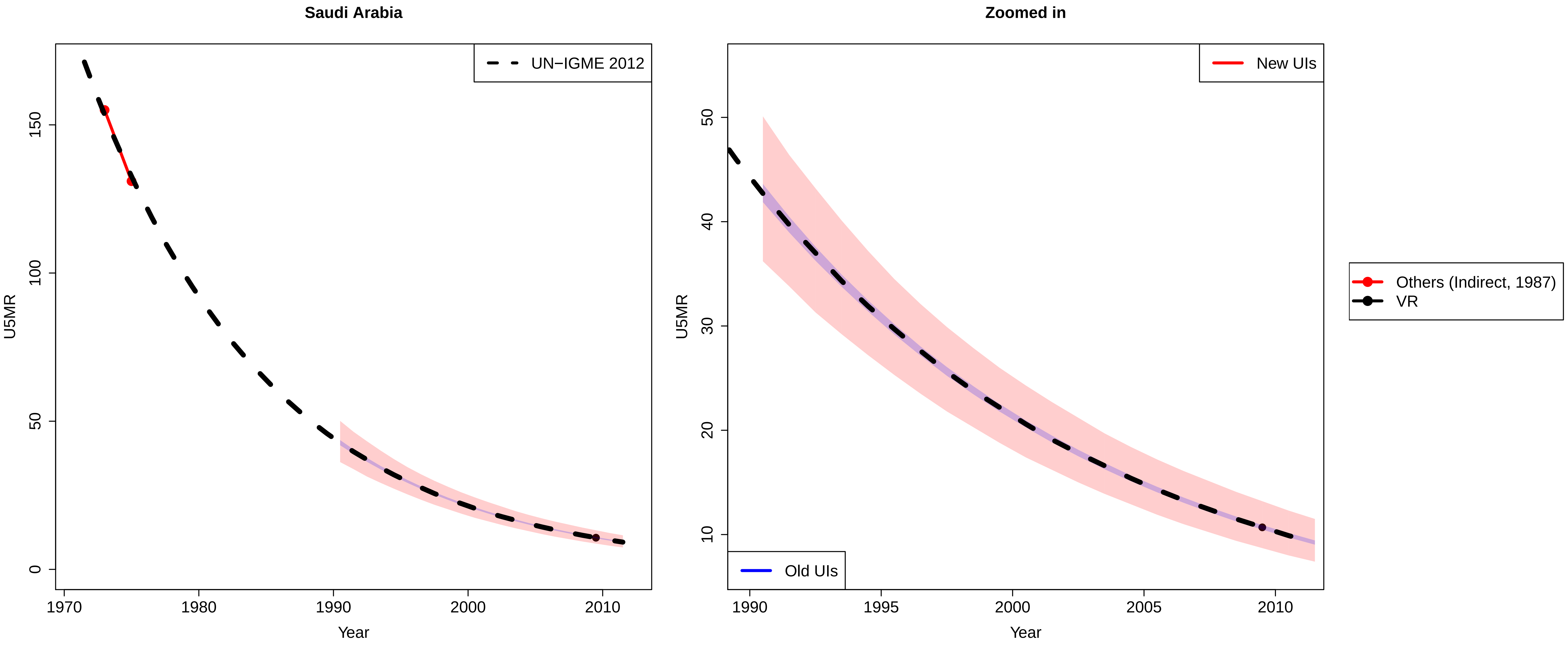

Saudi Arabia
UN−IGME 2012
U5MR
0
50
100
150
1970
1980
1990
2000
2010
Year
Zoomed in
New UIs
Old UIs
U5MR
10
20
30
40
50
1990
1995
2000
2005
2010
Year
Others (Indirect, 1987)
VR

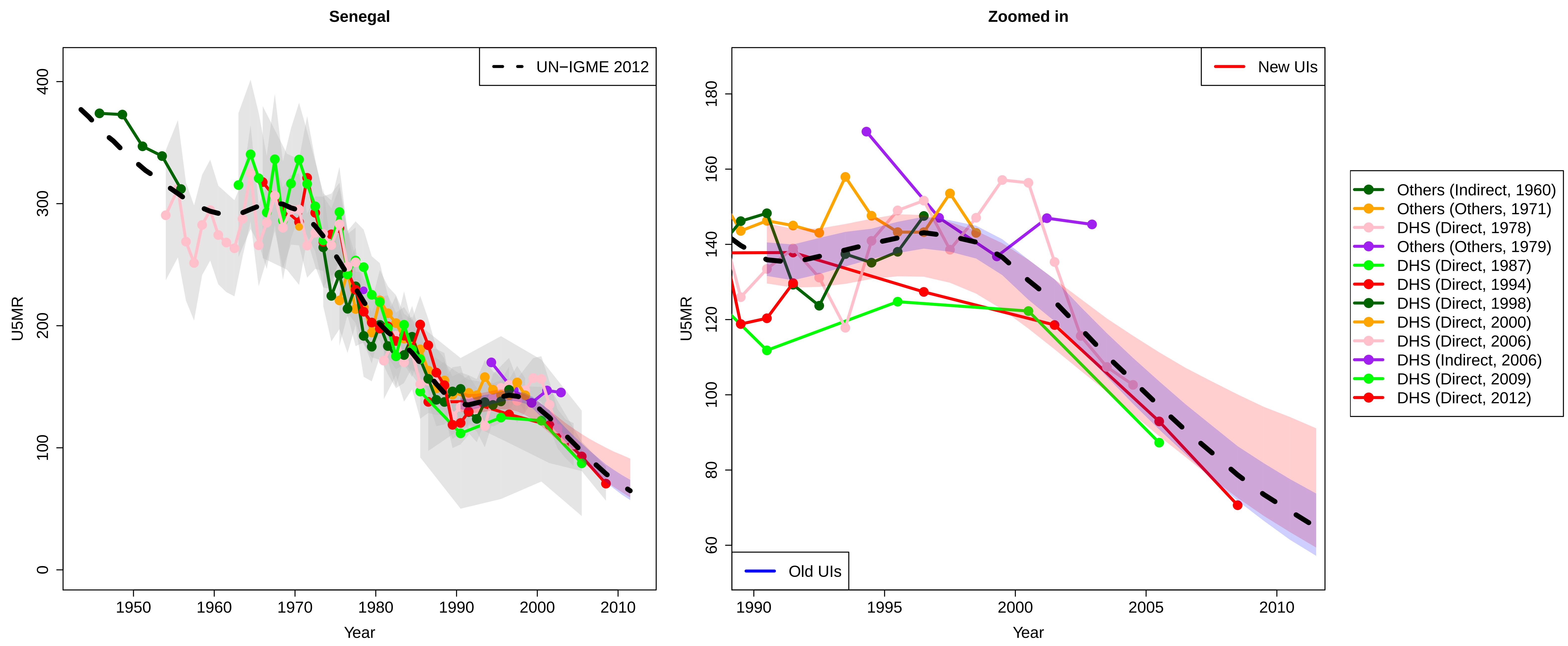

Senegal
UN−IGME 2012
U5MR
Year
Zoomed in
New UIs
Old UIs
Others (Indirect, 1960)
Others (Others, 1971)
DHS (Direct, 1978)
Others (Others, 1979)
DHS (Direct, 1987)
DHS (Direct, 1994)
DHS (Direct, 1998)
DHS (Direct, 2000)
DHS (Direct, 2006)
DHS (Indirect, 2006)
DHS (Direct, 2009)
DHS (Direct, 2012)

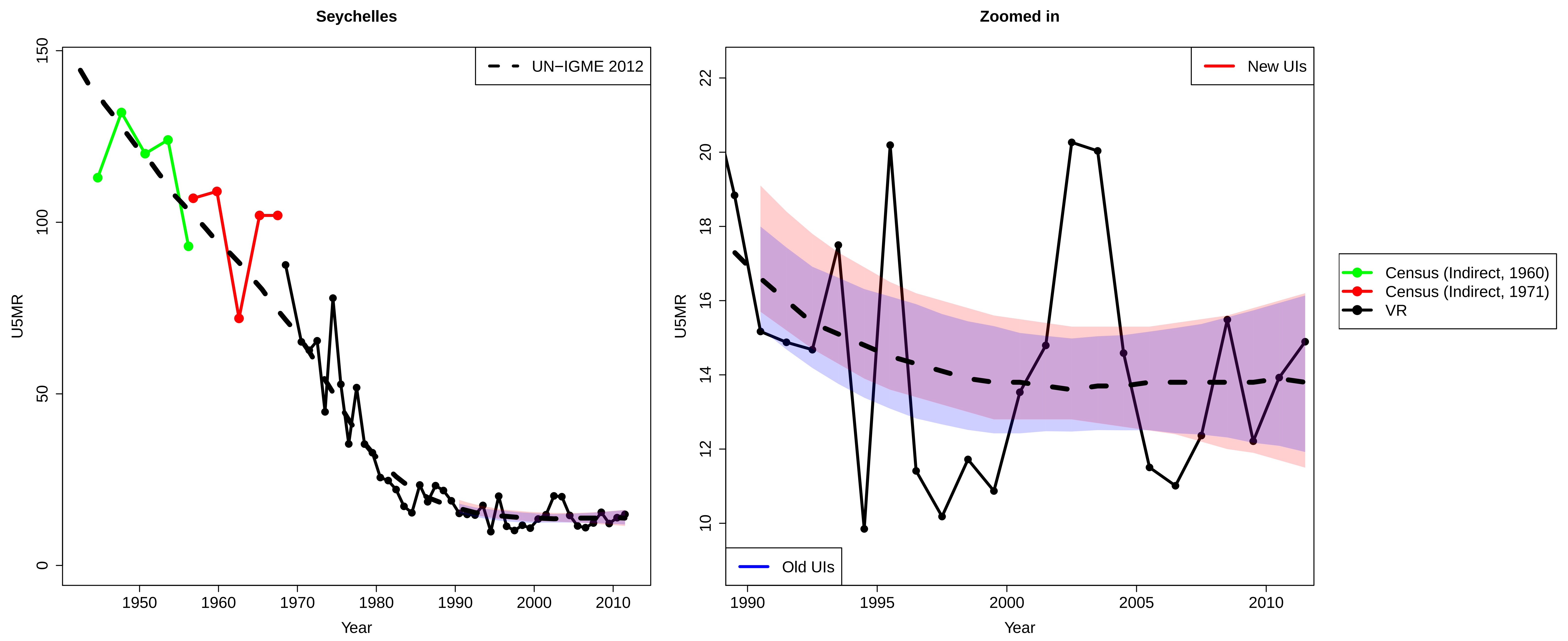
Seychelles
UN−IGME 2012
U5MR
Year
Zoomed in
New UIs
Old UIs
Census (Indirect, 1960)
Census (Indirect, 1971)
VR

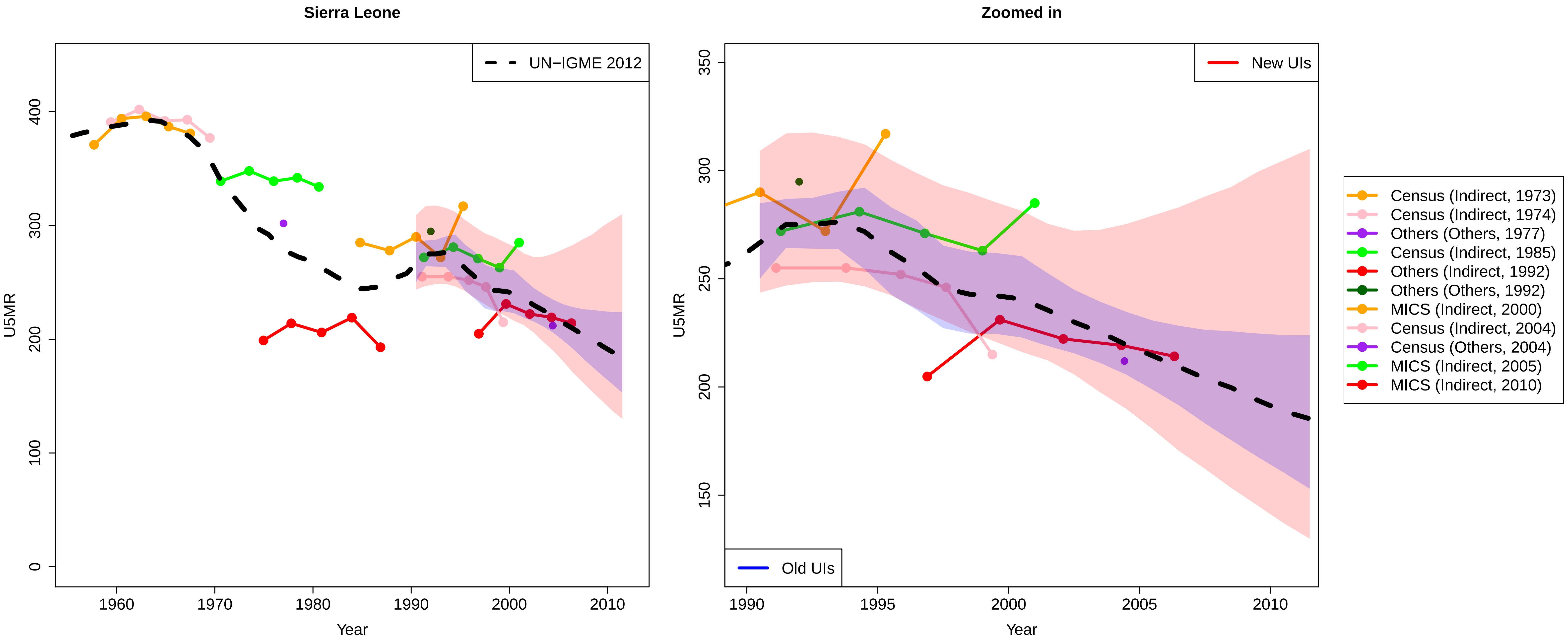
Sierra Leone
UN−IGME 2012
U5MR
Year
0
100
200
300
400
1960
1970
1980
1990
2000
2010
Zoomed in
New UIs
Old UIs
U5MR
Year
150
200
250
300
350
1990
1995
2000
2005
2010
Census (Indirect, 1973)
Census (Indirect, 1974)
Others (Others, 1977)
Census (Indirect, 1985)
Others (Indirect, 1992)
Others (Others, 1992)
MICS (Indirect, 2000)
Census (Indirect, 2004)
Census (Others, 2004)
MICS (Indirect, 2005)
MICS (Indirect, 2010)

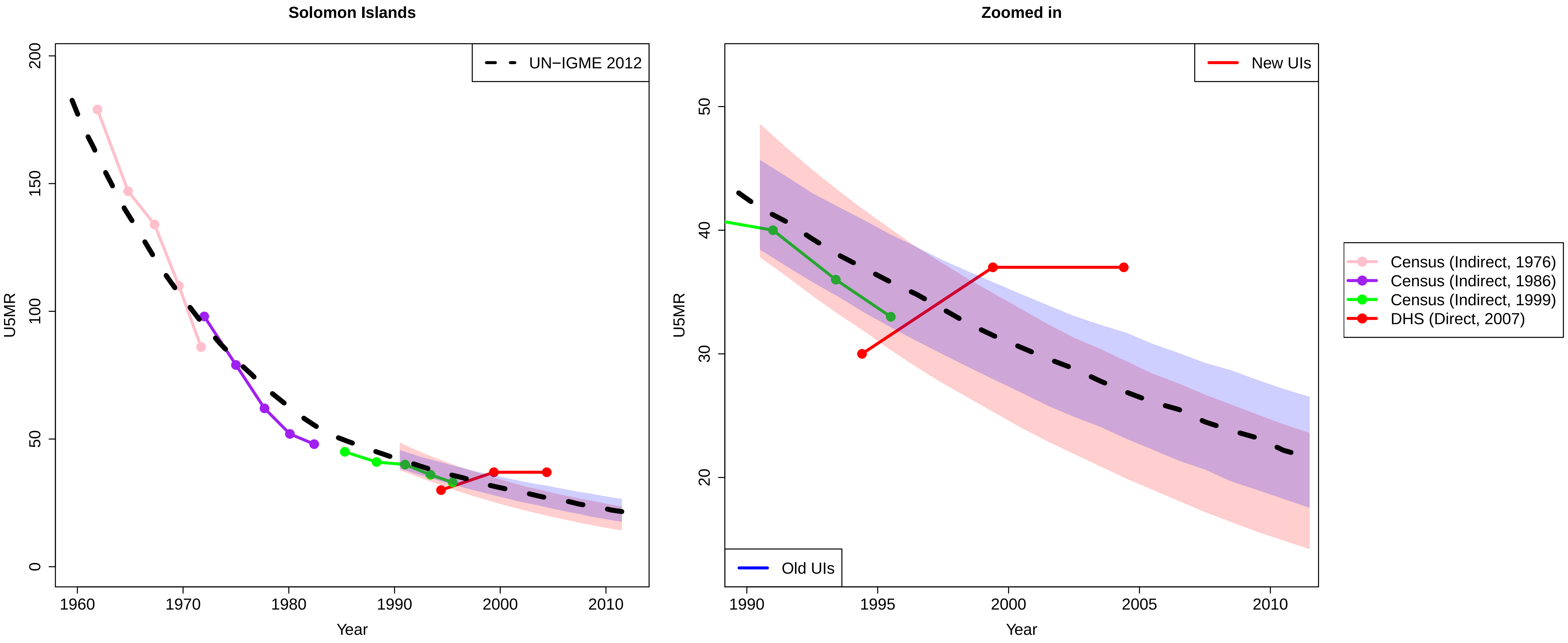

Solomon Islands
Zoomed in
UN−IGME 2012
New UIs
Old UIs
Census (Indirect, 1976)
Census (Indirect, 1986)
Census (Indirect, 1999)
DHS (Direct, 2007)
U5MR
Year
0
50
100
150
200
1960
1970
1980
1990
2000
2010
20
30
40
50
1990
1995
2000
2005
2010

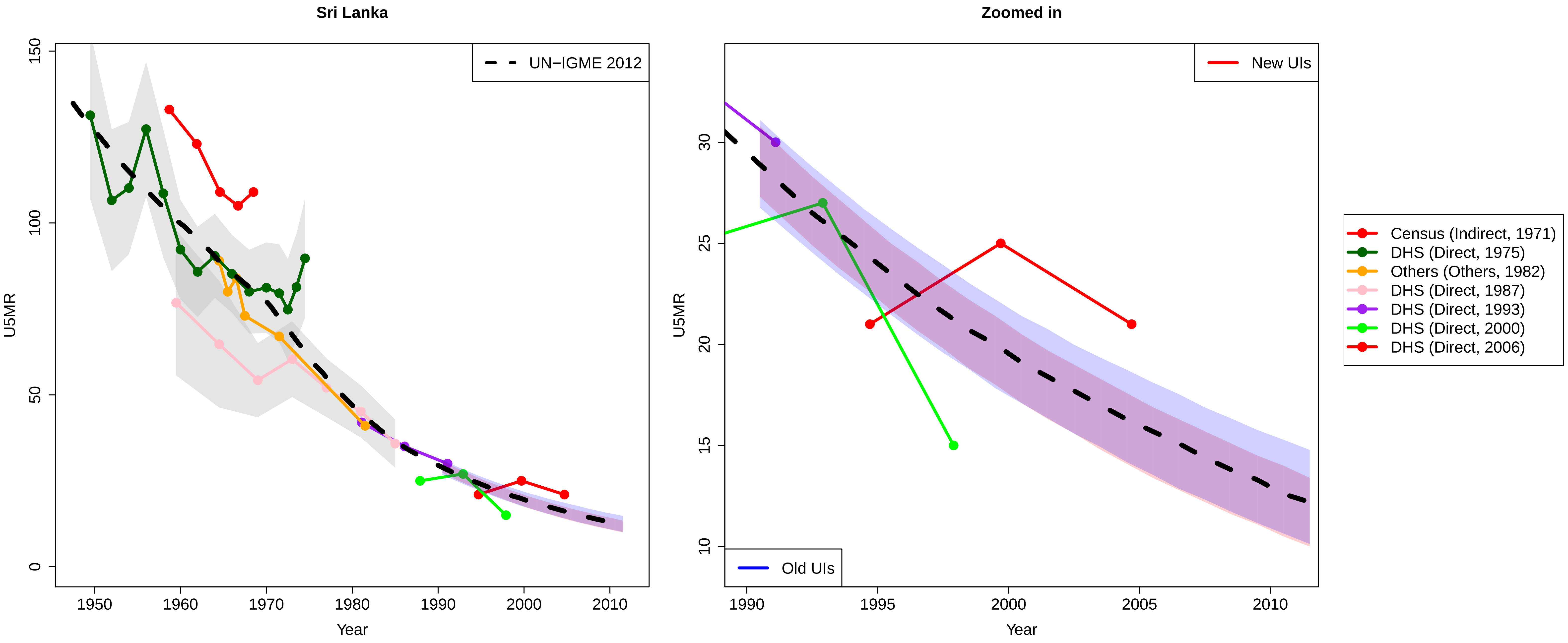

Sri Lanka
Zoomed in
UN–IGME 2012
New UIs
Old UIs
U5MR
Year
0
50
100
150
1950
1960
1970
1980
1990
2000
2010
10
15
20
25
30
1995
2005
Census (Indirect, 1971)
DHS (Direct, 1975)
Others (Others, 1982)
DHS (Direct, 1987)
DHS (Direct, 1993)
DHS (Direct, 2000)
DHS (Direct, 2006)

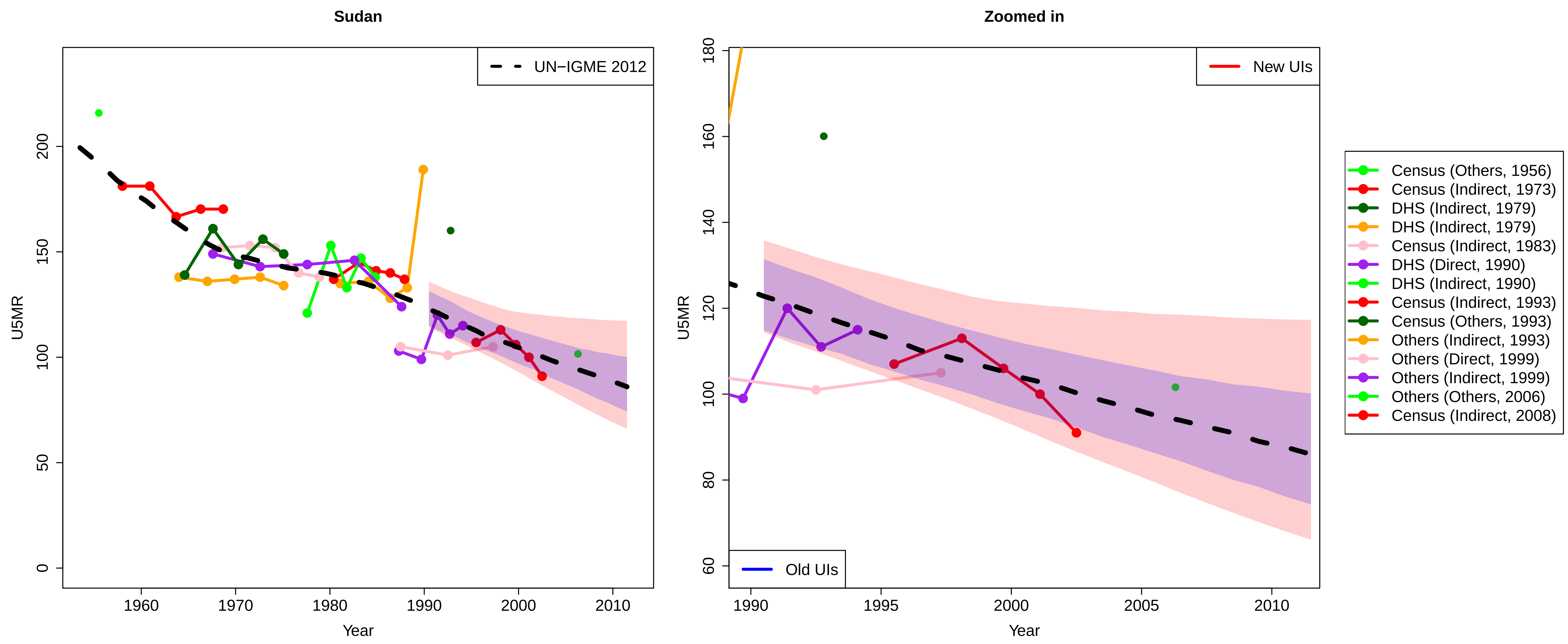
Sudan
UN−IGME 2012
U5MR
Year
Zoomed in
New UIs
Old UIs
Census (Others, 1956)
Census (Indirect, 1973)
DHS (Indirect, 1979)
DHS (Indirect, 1979)
Census (Indirect, 1983)
DHS (Direct, 1990)
DHS (Indirect, 1990)
Census (Indirect, 1993)
Census (Others, 1993)
Others (Indirect, 1993)
Others (Direct, 1999)
Others (Indirect, 1999)
Others (Others, 2006)
Census (Indirect, 2008)

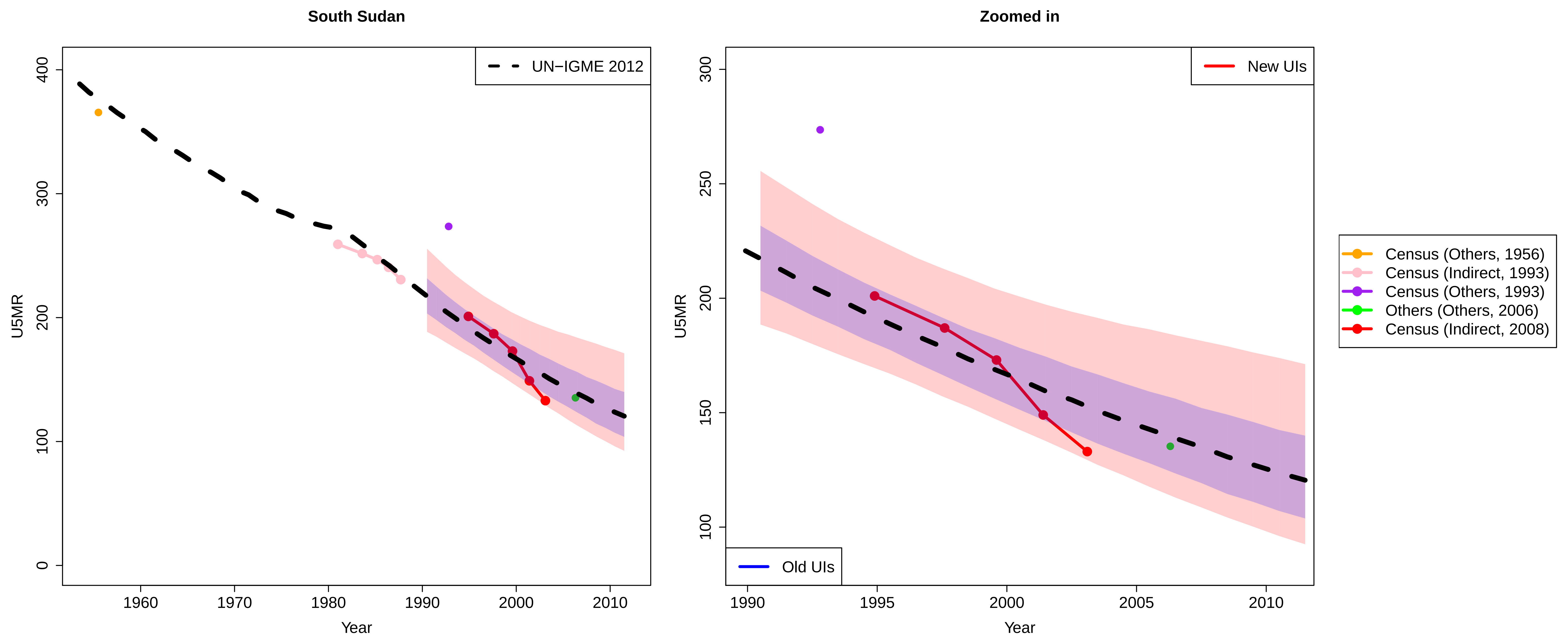

South Sudan
Zoomed in
UN−IGME 2012
New UIs
Old UIs
U5MR
Year
Census (Others, 1956)
Census (Indirect, 1993)
Census (Others, 1993)
Others (Others, 2006)
Census (Indirect, 2008)

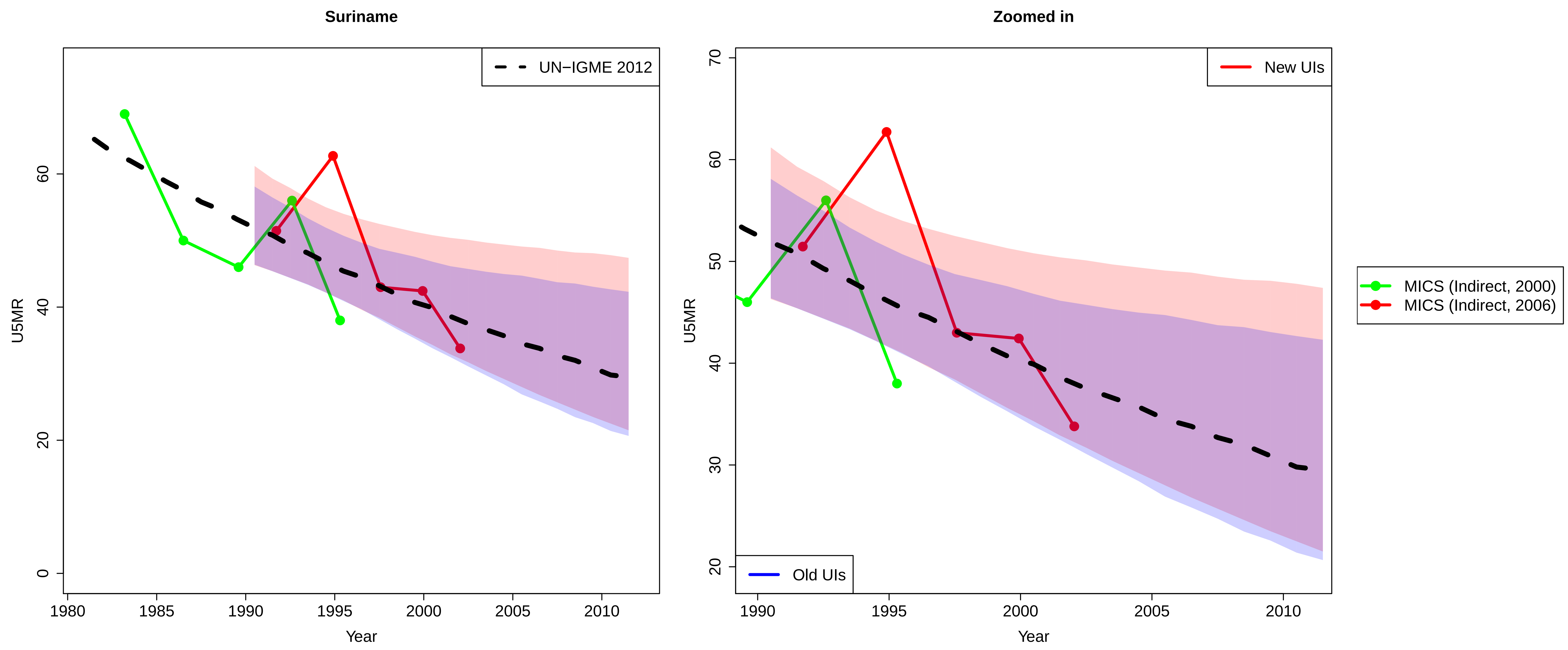

Suriname
Zoomed in
UN−IGME 2012
New UIs
Old UIs
MICS (Indirect, 2000)
MICS (Indirect, 2006)
U5MR
Year
0
20
40
60
30
50
70
1980
1985
1990
1995
2000
2005
2010

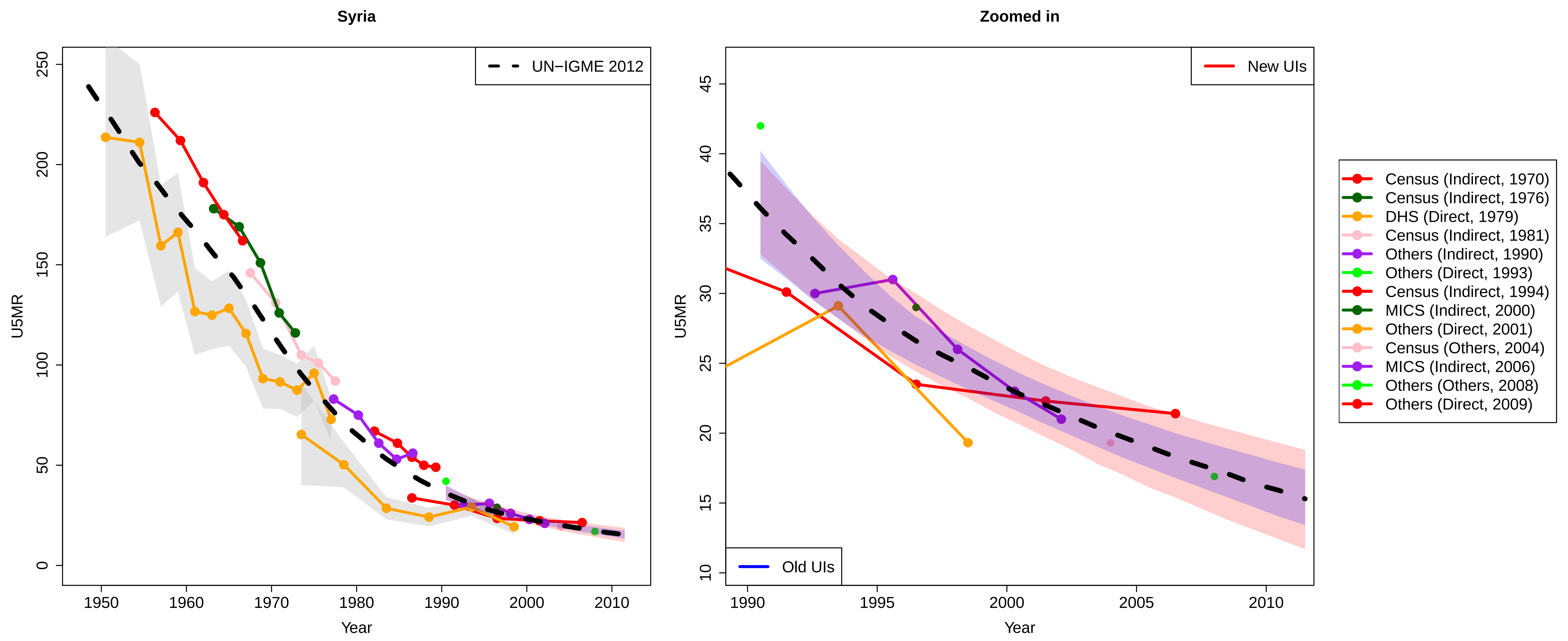

Syria
Zoomed in
UN–IGME 2012
New UIs
Old UIs
U5MR
Year
Census (Indirect, 1970)
Census (Indirect, 1976)
DHS (Direct, 1979)
Census (Indirect, 1981)
Others (Indirect, 1990)
Others (Direct, 1993)
Census (Indirect, 1994)
MICS (Indirect, 2000)
Others (Direct, 2001)
Census (Others, 2004)
MICS (Indirect, 2006)
Others (Others, 2008)
Others (Direct, 2009)

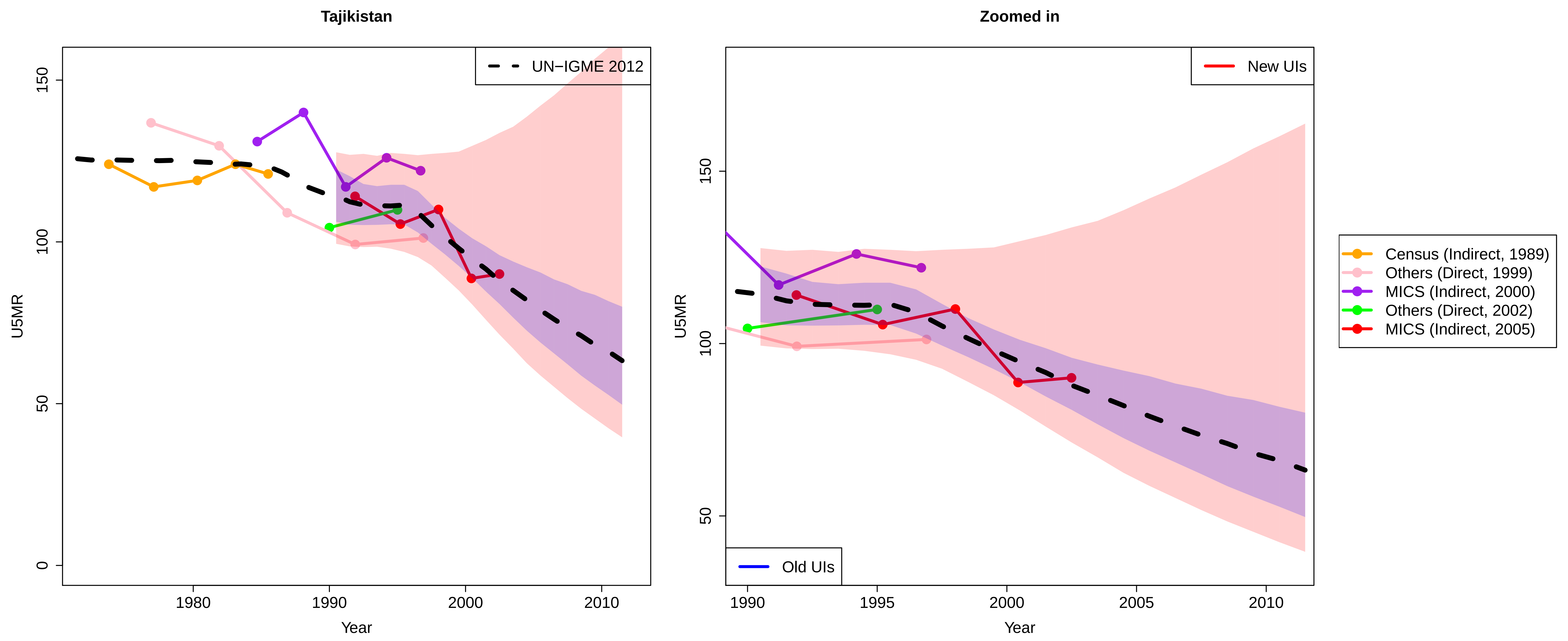
Tajikistan
UN−IGME 2012
U5MR
Year
Zoomed in
New UIs
Old UIs
Census (Indirect, 1989)
Others (Direct, 1999)
MICS (Indirect, 2000)
Others (Direct, 2002)
MICS (Indirect, 2005)

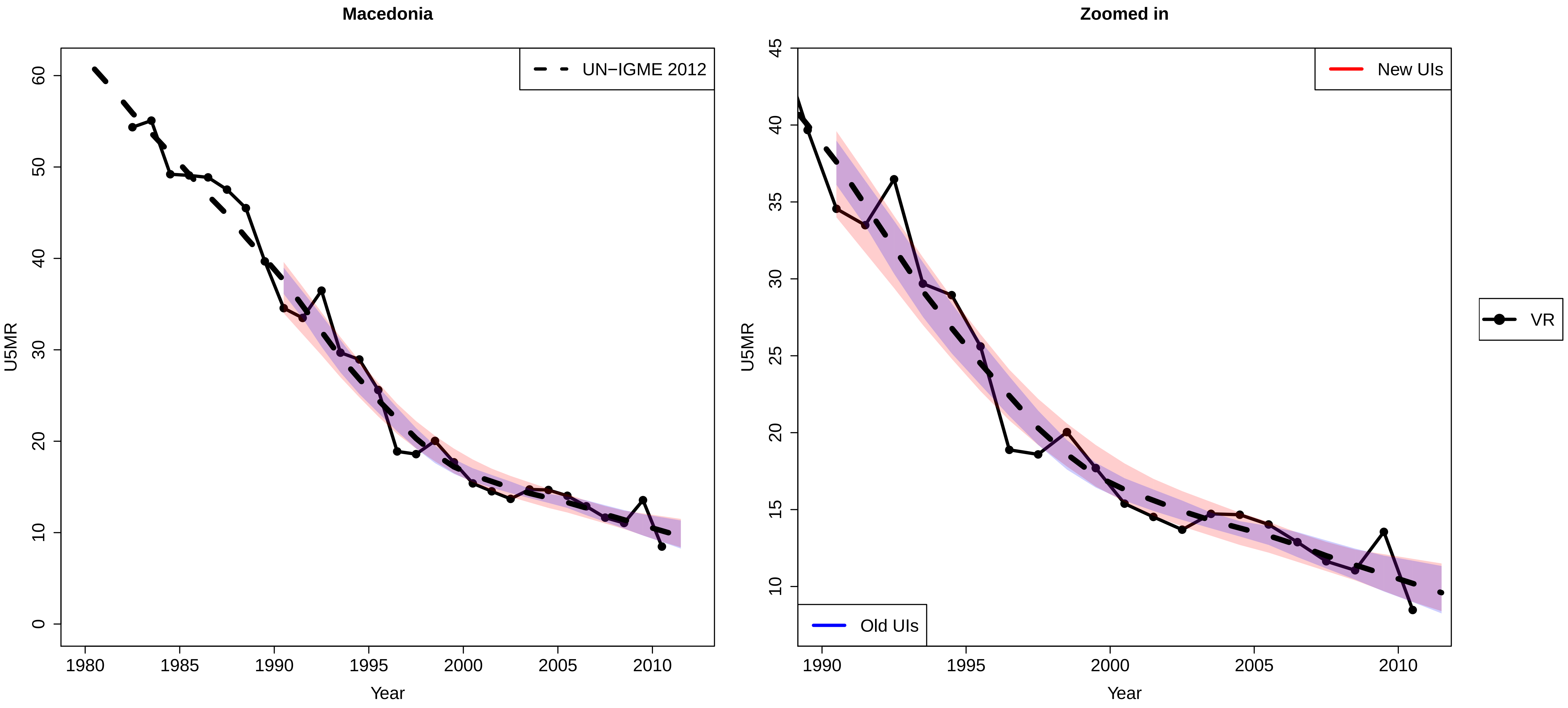
Macedonia
UN−IGME 2012
U5MR
Year
Zoomed in
New UIs
Old UIs
VR

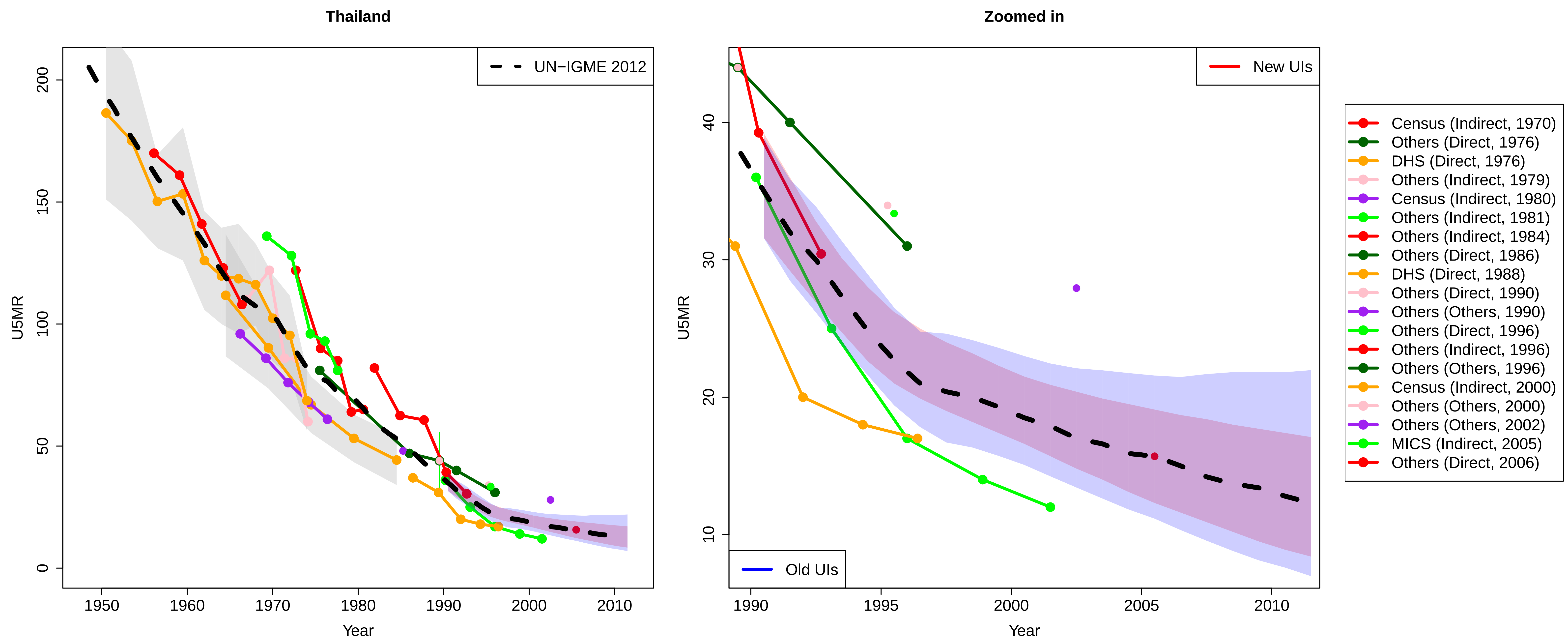
Thailand
UN−IGME 2012
U5MR
Year
Zoomed in
New UIs
Old UIs
Census (Indirect, 1970)
Others (Direct, 1976)
DHS (Direct, 1976)
Others (Indirect, 1979)
Census (Indirect, 1980)
Others (Indirect, 1981)
Others (Indirect, 1984)
Others (Direct, 1986)
DHS (Direct, 1988)
Others (Direct, 1990)
Others (Others, 1990)
Others (Direct, 1996)
Others (Indirect, 1996)
Others (Others, 1996)
Census (Indirect, 2000)
Others (Others, 2000)
Others (Others, 2002)
MICS (Indirect, 2005)
Others (Direct, 2006)

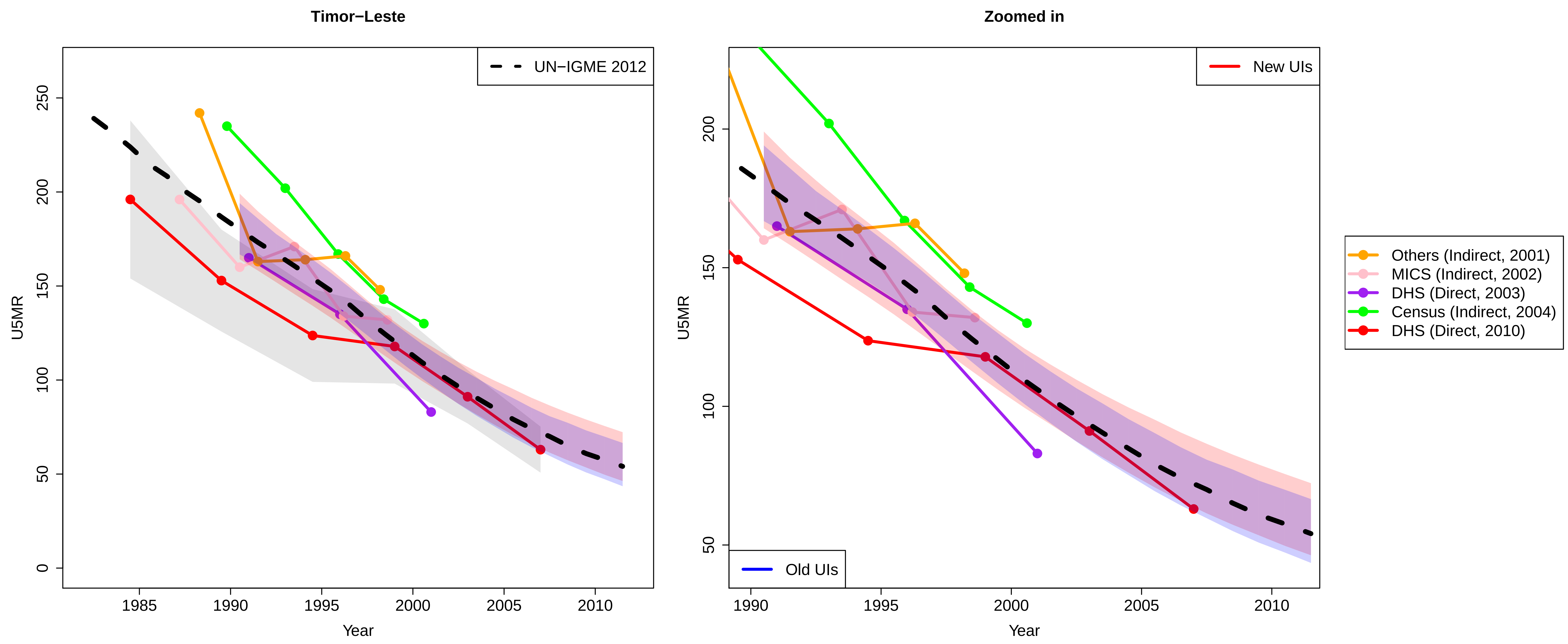

Timor−Leste
Zoomed in
UN−IGME 2012
New UIs
Old UIs
Others (Indirect, 2001)
MICS (Indirect, 2002)
DHS (Direct, 2003)
Census (Indirect, 2004)
DHS (Direct, 2010)
U5MR
Year

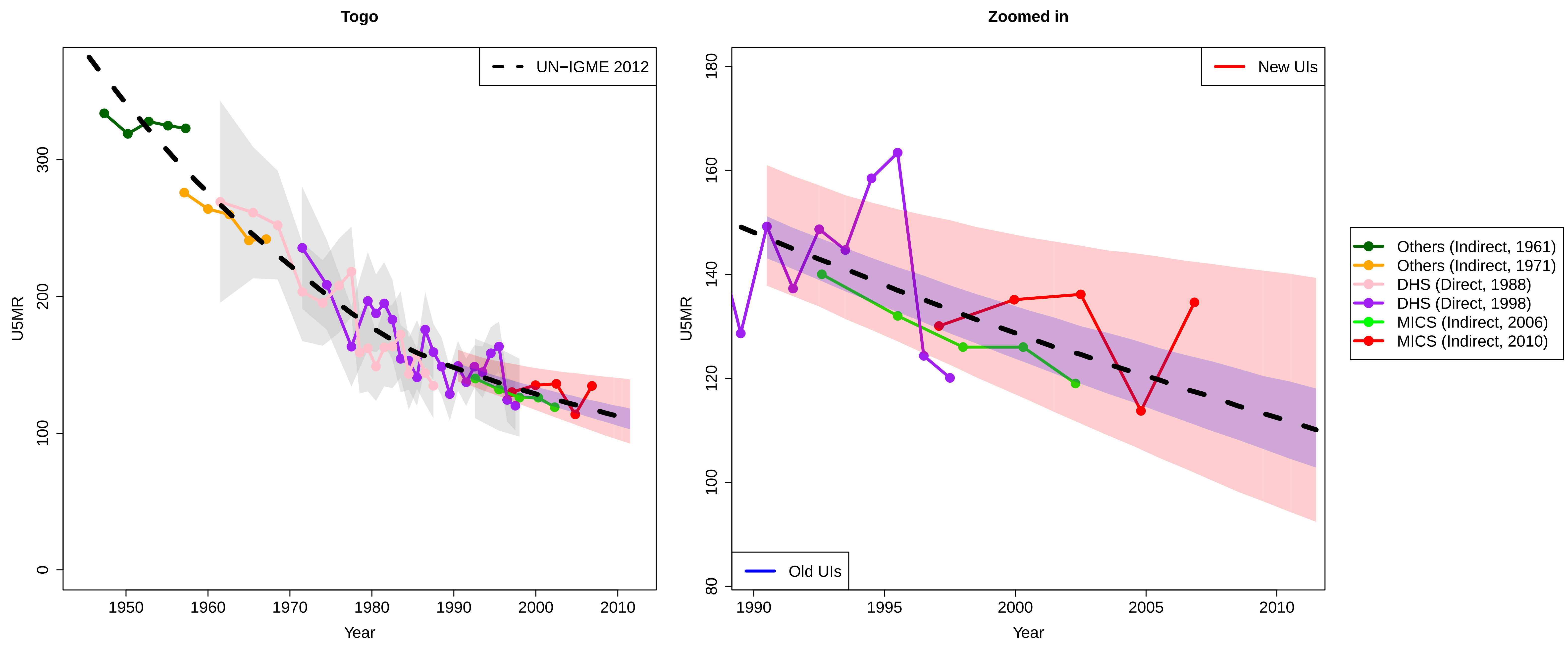
Togo
UN−IGME 2012
U5MR
Year
Zoomed in
New UIs
Old UIs
Others (Indirect, 1961)
Others (Indirect, 1971)
DHS (Direct, 1988)
DHS (Direct, 1998)
MICS (Indirect, 2006)
MICS (Indirect, 2010)

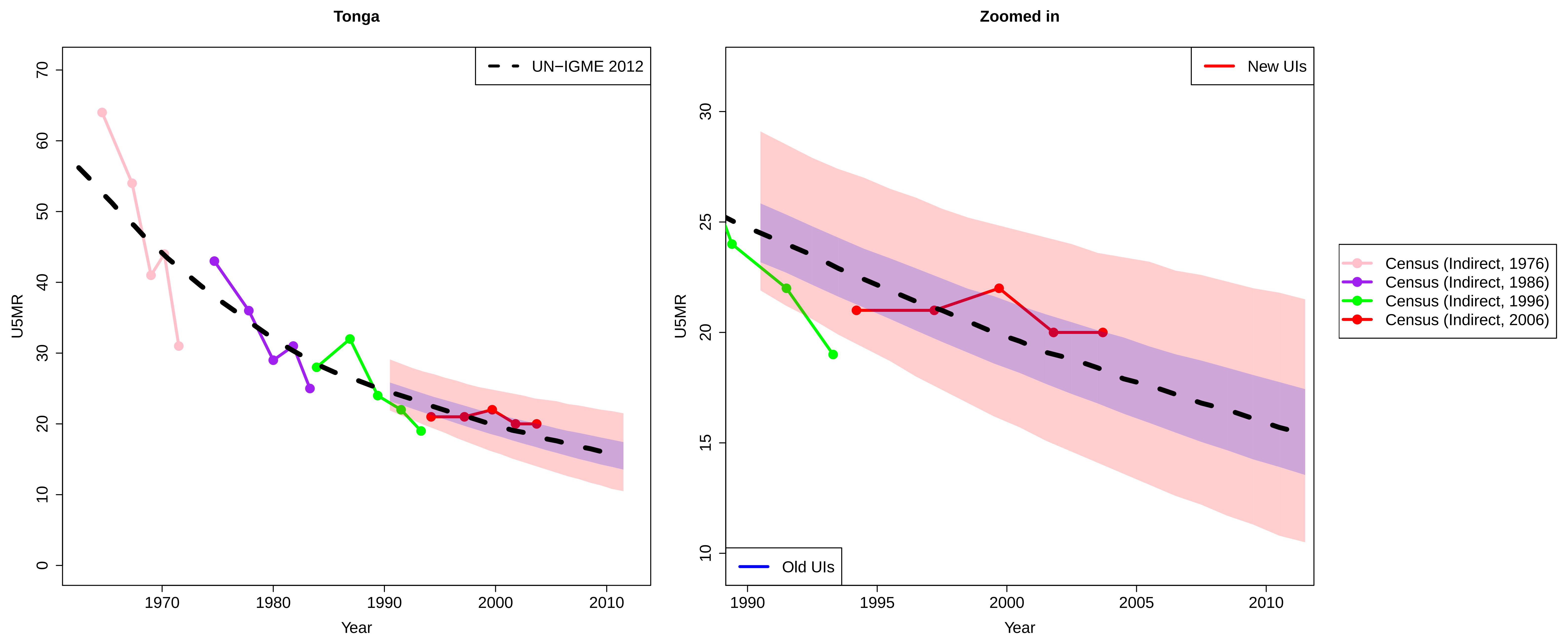

Tonga
Zoomed in
UN−IGME 2012
New UIs
Old UIs
Census (Indirect, 1976)
Census (Indirect, 1986)
Census (Indirect, 1996)
Census (Indirect, 2006)
U5MR
Year

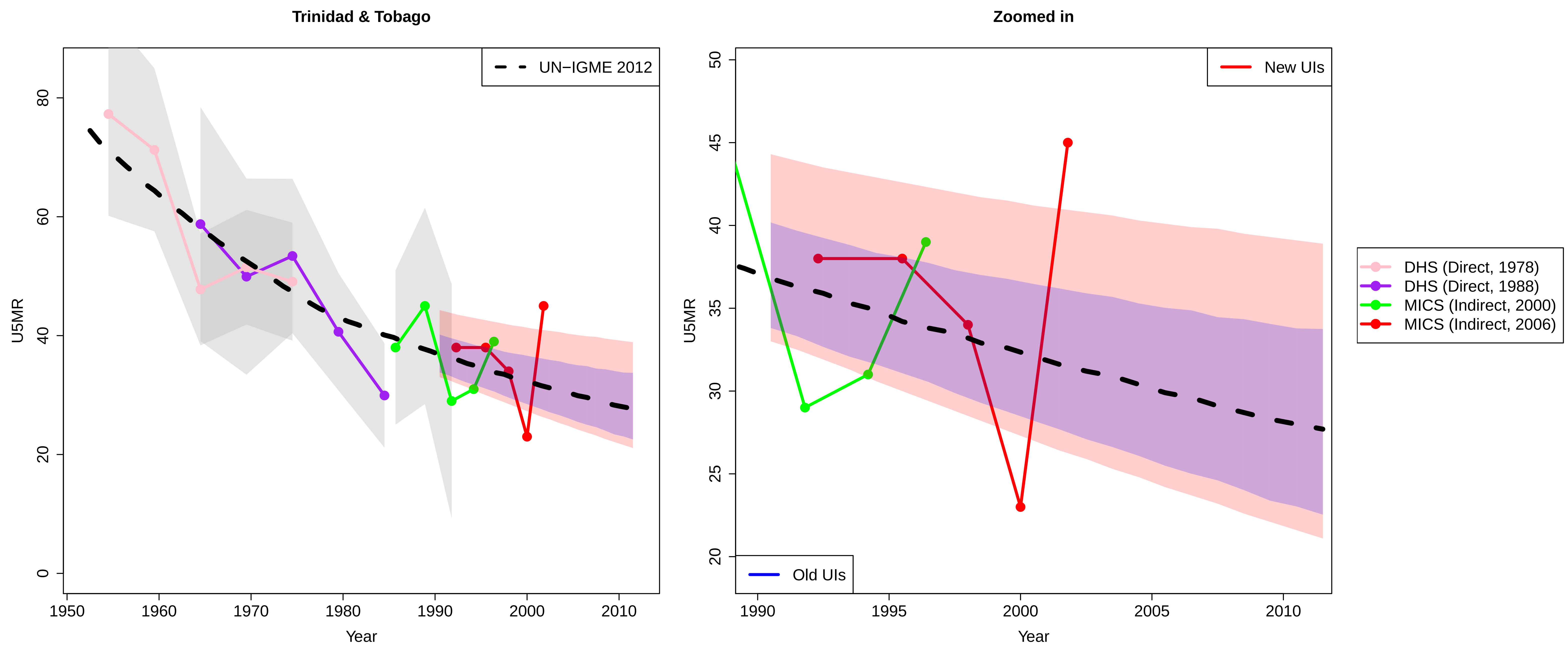

Trinidad & Tobago
Zoomed in
UN−IGME 2012
New UIs
Old UIs
DHS (Direct, 1978)
DHS (Direct, 1988)
MICS (Indirect, 2000)
MICS (Indirect, 2006)
U5MR
Year
0
20
40
60
80
1950
1960
1970
1980
1990
2000
2010
20
25
30
35
40
45
50
1995
2005

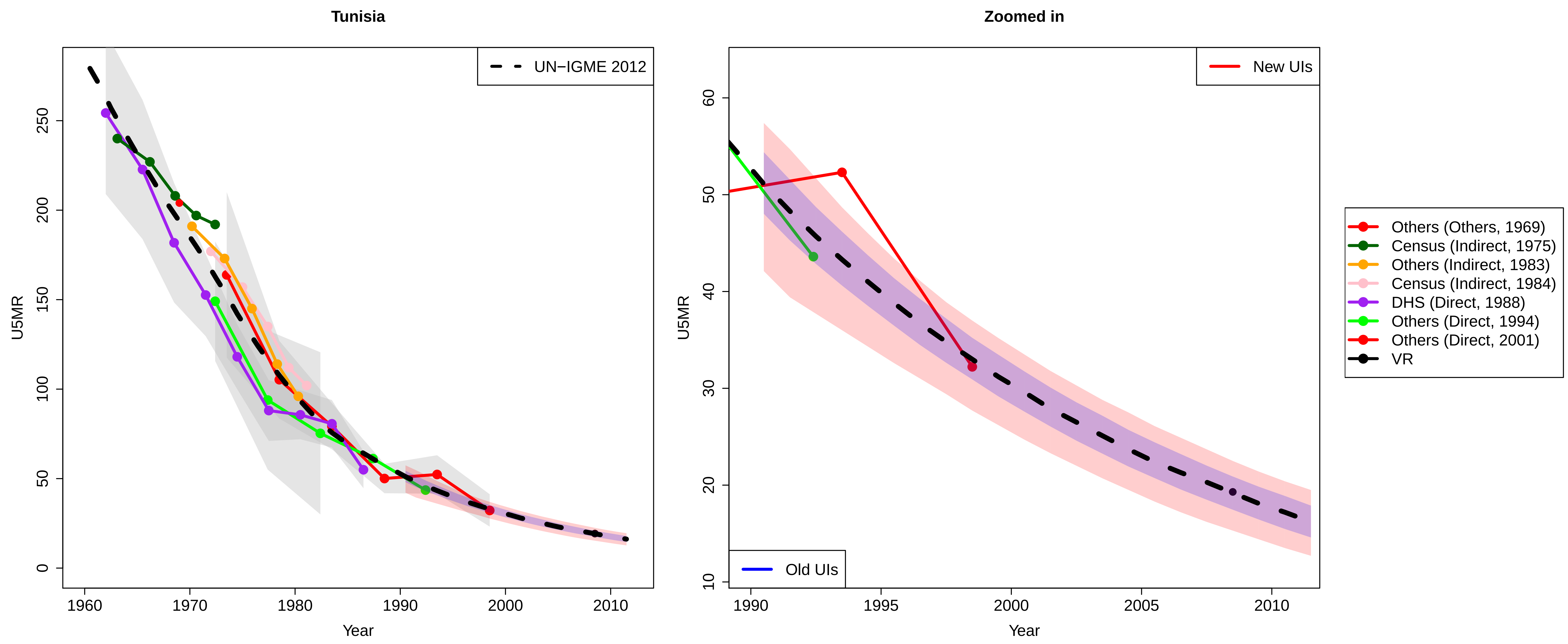

Tunisia
Zoomed in
UN−IGME 2012
New UIs
Old UIs
Others (Others, 1969)
Census (Indirect, 1975)
Others (Indirect, 1983)
Census (Indirect, 1984)
DHS (Direct, 1988)
Others (Direct, 1994)
Others (Direct, 2001)
VR
U5MR
Year

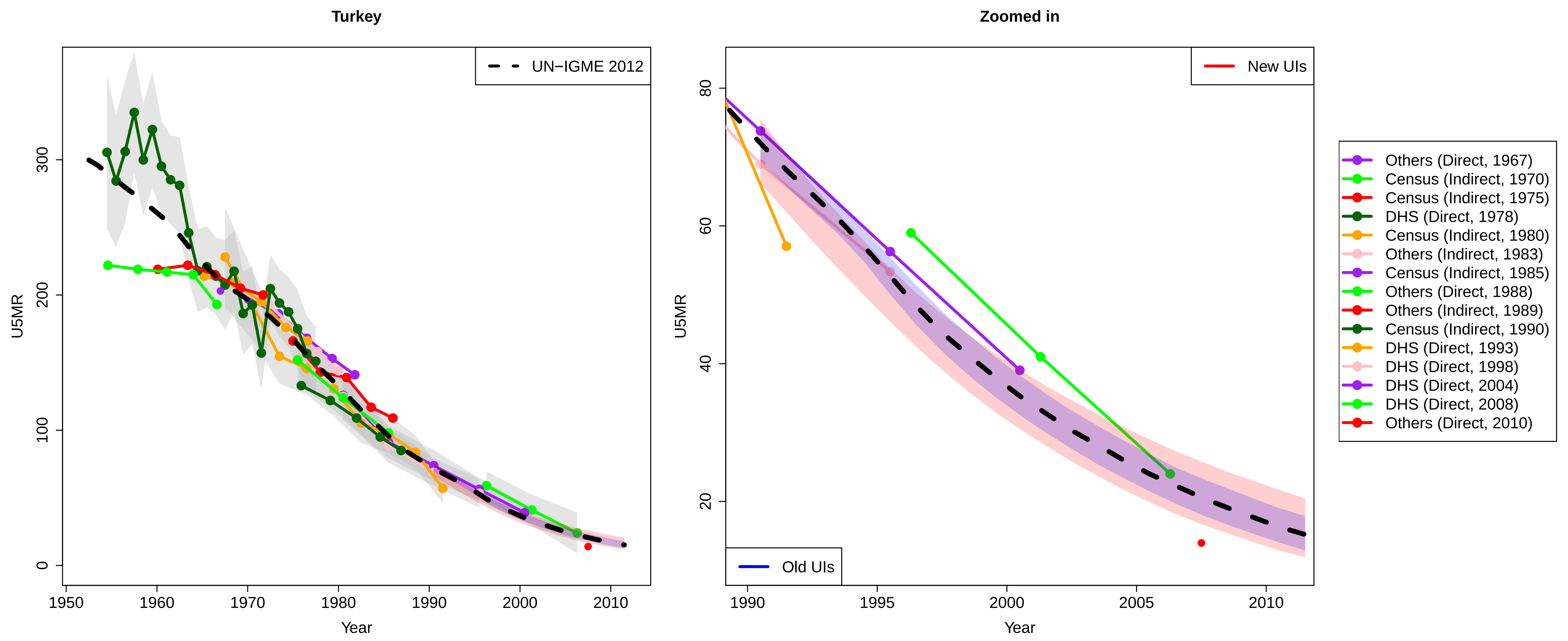
Turkey
UN−IGME 2012
U5MR
Year
Zoomed in
New UIs
Old UIs
Others (Direct, 1967)
Census (Indirect, 1970)
Census (Indirect, 1975)
DHS (Direct, 1978)
Census (Indirect, 1980)
Others (Indirect, 1983)
Census (Indirect, 1985)
Others (Direct, 1988)
Others (Indirect, 1989)
Census (Indirect, 1990)
DHS (Direct, 1993)
DHS (Direct, 1998)
DHS (Direct, 2004)
DHS (Direct, 2008)
Others (Direct, 2010)

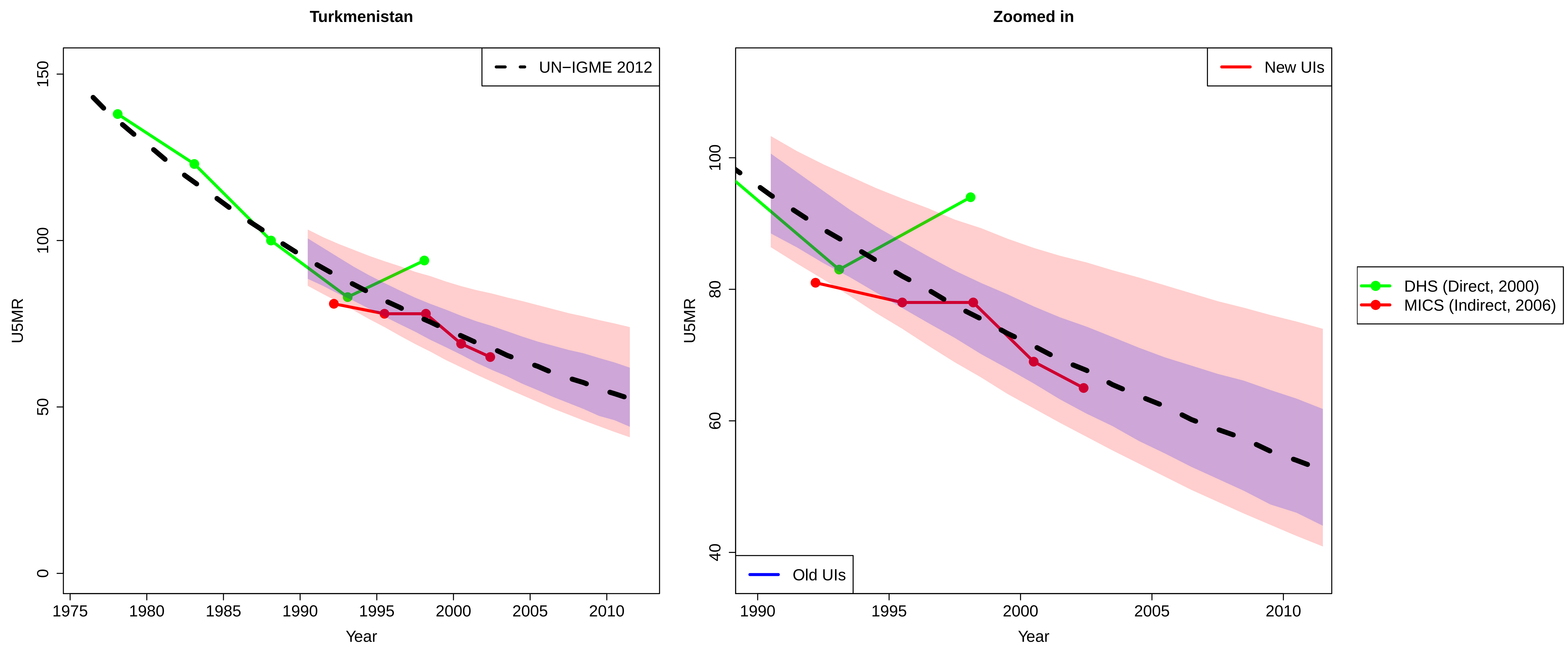

Turkmenistan
Zoomed in
UN−IGME 2012
New UIs
Old UIs
DHS (Direct, 2000)
MICS (Indirect, 2006)
U5MR
Year
0
50
100
150
40
60
80
100
1975
1980
1985
1990
1995
2000
2005
2010

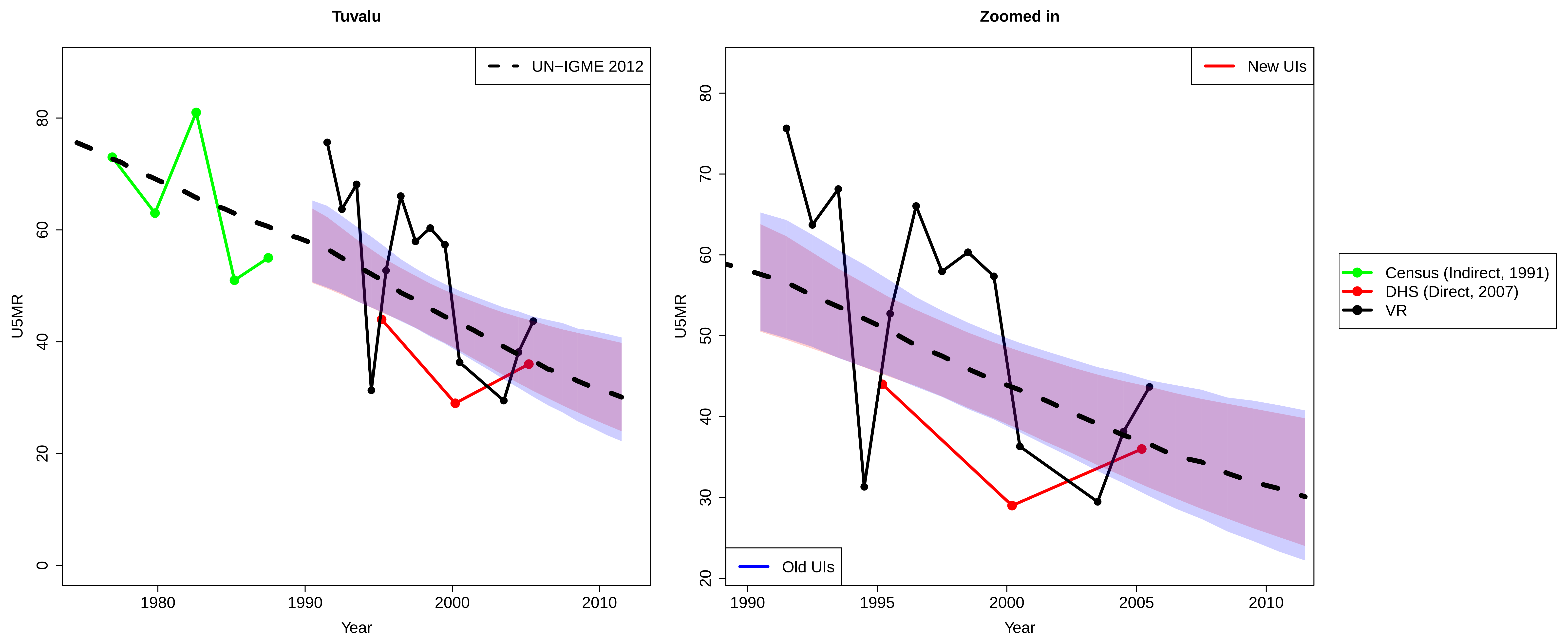

Tuvalu
Zoomed in
UN−IGME 2012
New UIs
Old UIs
Census (Indirect, 1991)
DHS (Direct, 2007)
VR
U5MR
Year
0
20
40
60
80
30
50
70
1980
1990
1995
2000
2005
2010

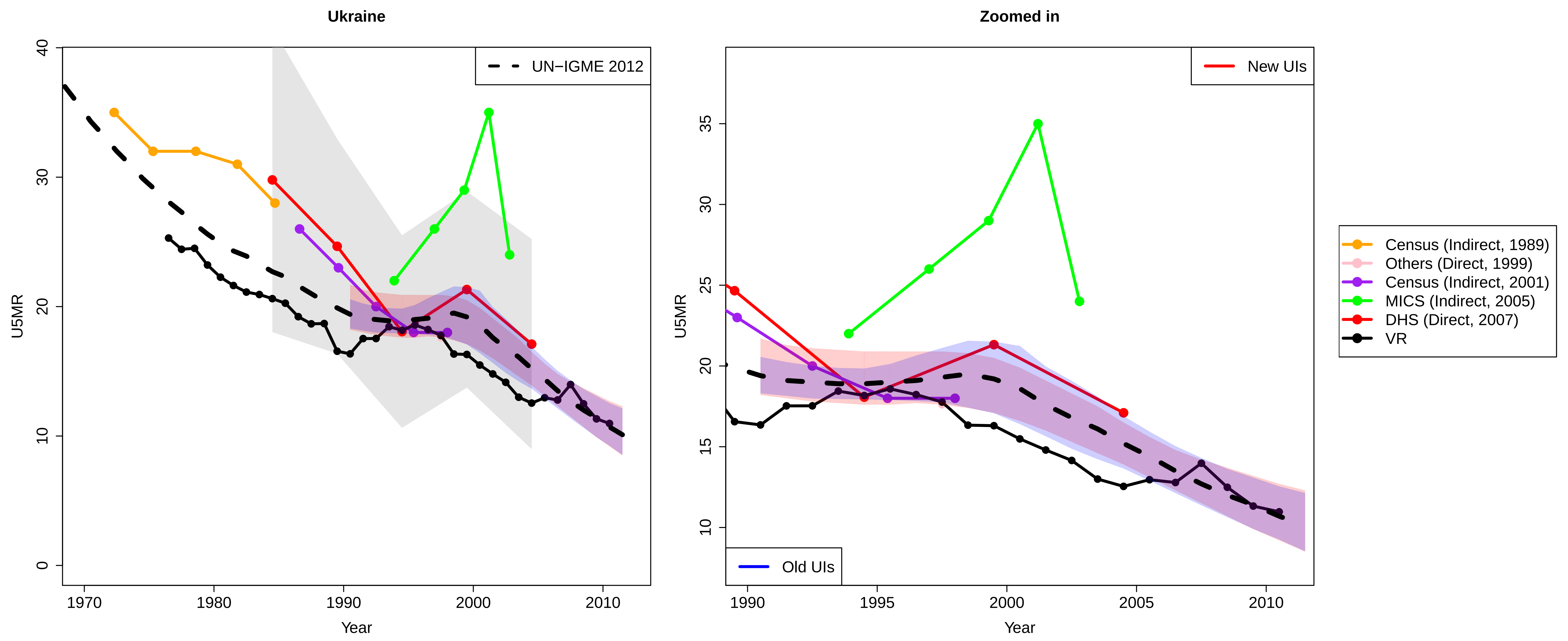

Ukraine
Zoomed in
UN−IGME 2012
New UIs
Old UIs
Census (Indirect, 1989)
Others (Direct, 1999)
Census (Indirect, 2001)
MICS (Indirect, 2005)
DHS (Direct, 2007)
VR
U5MR
Year

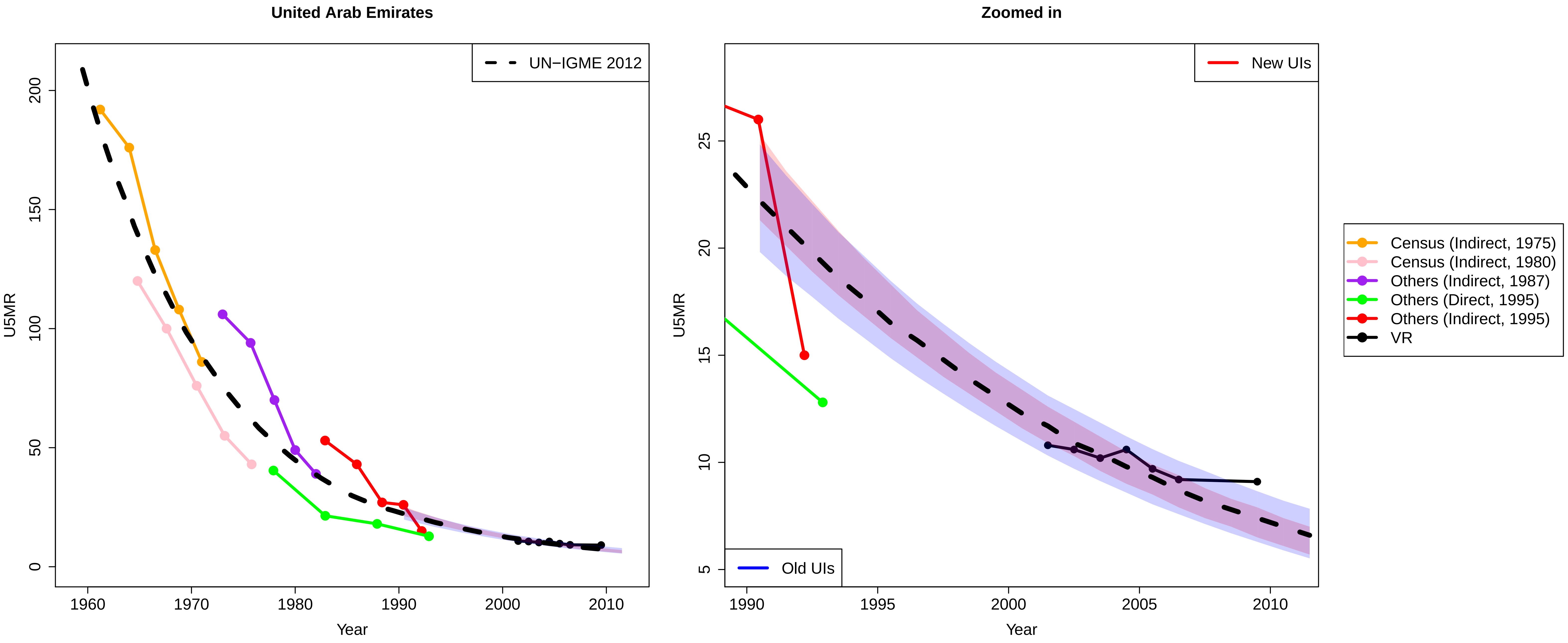
United Arab Emirates
UN−IGME 2012
U5MR
Year
Zoomed in
New UIs
Old UIs
Census (Indirect, 1975)
Census (Indirect, 1980)
Others (Indirect, 1987)
Others (Direct, 1995)
Others (Indirect, 1995)
VR

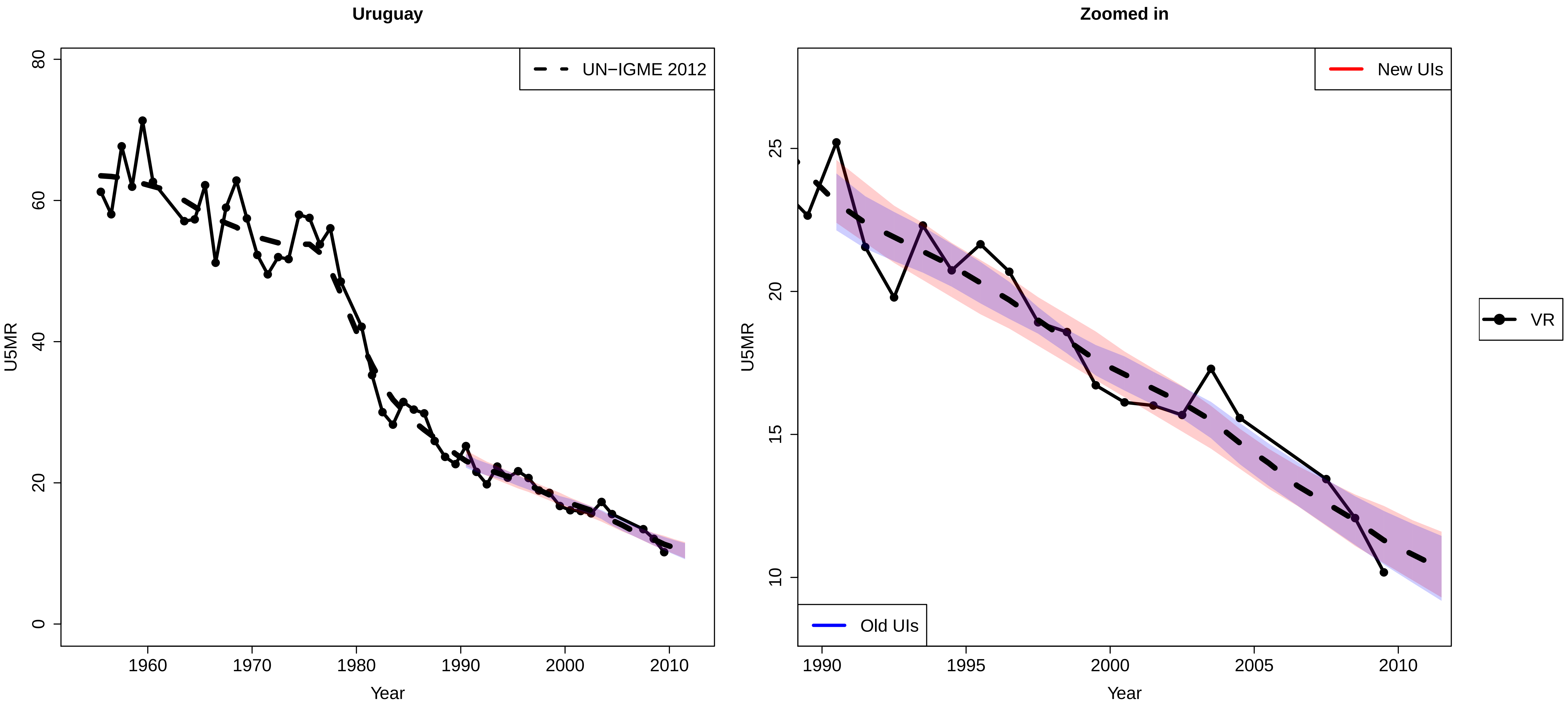

Uruguay
Zoomed in
UN−IGME 2012
New UIs
Old UIs
VR
U5MR
Year
0
20
40
60
80
1960
1970
1980
1990
2000
2010
10
15
20
25
1995
2005

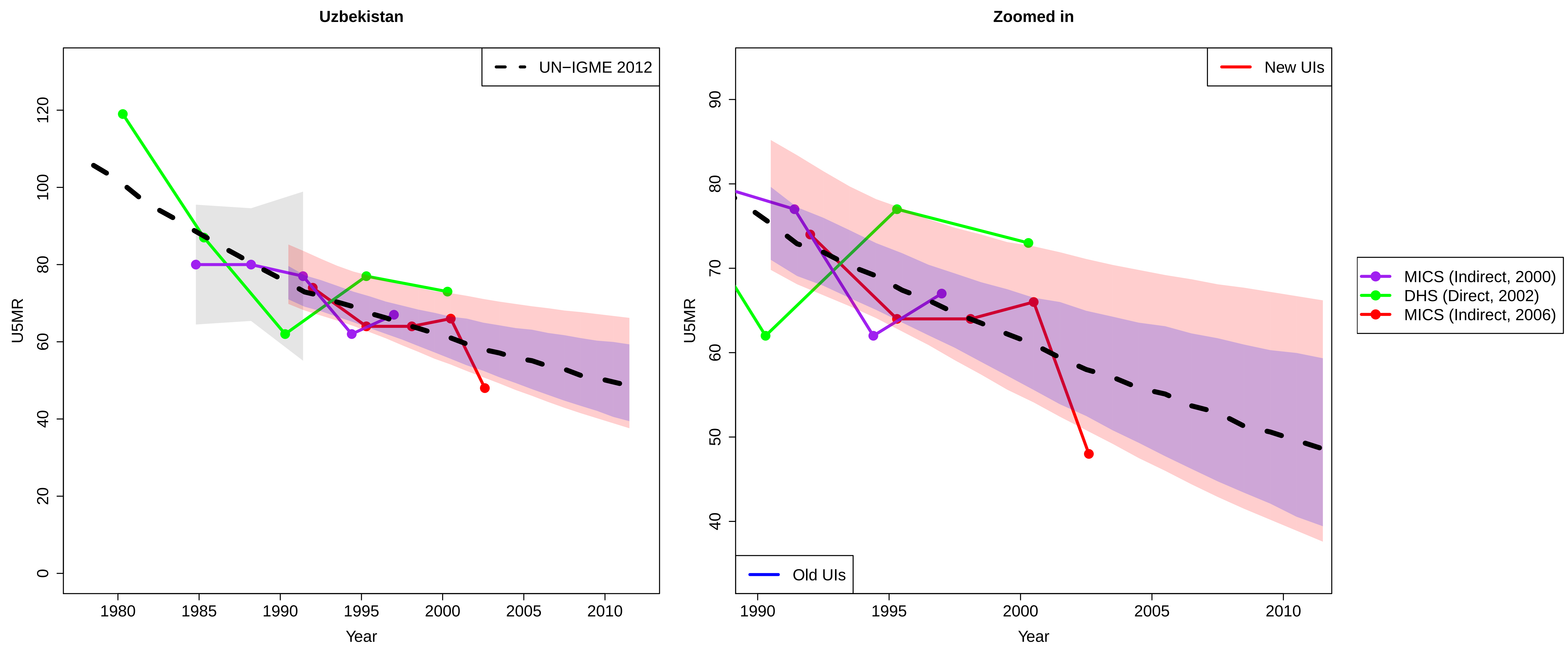
Uzbekistan
Zoomed in
UN−IGME 2012
New UIs
Old UIs
MICS (Indirect, 2000)
DHS (Direct, 2002)
MICS (Indirect, 2006)
U5MR
Year
0
20
40
60
80
100
120
1980
1985
1990
1995
2000
2005
2010
40
50
60
70
80
90

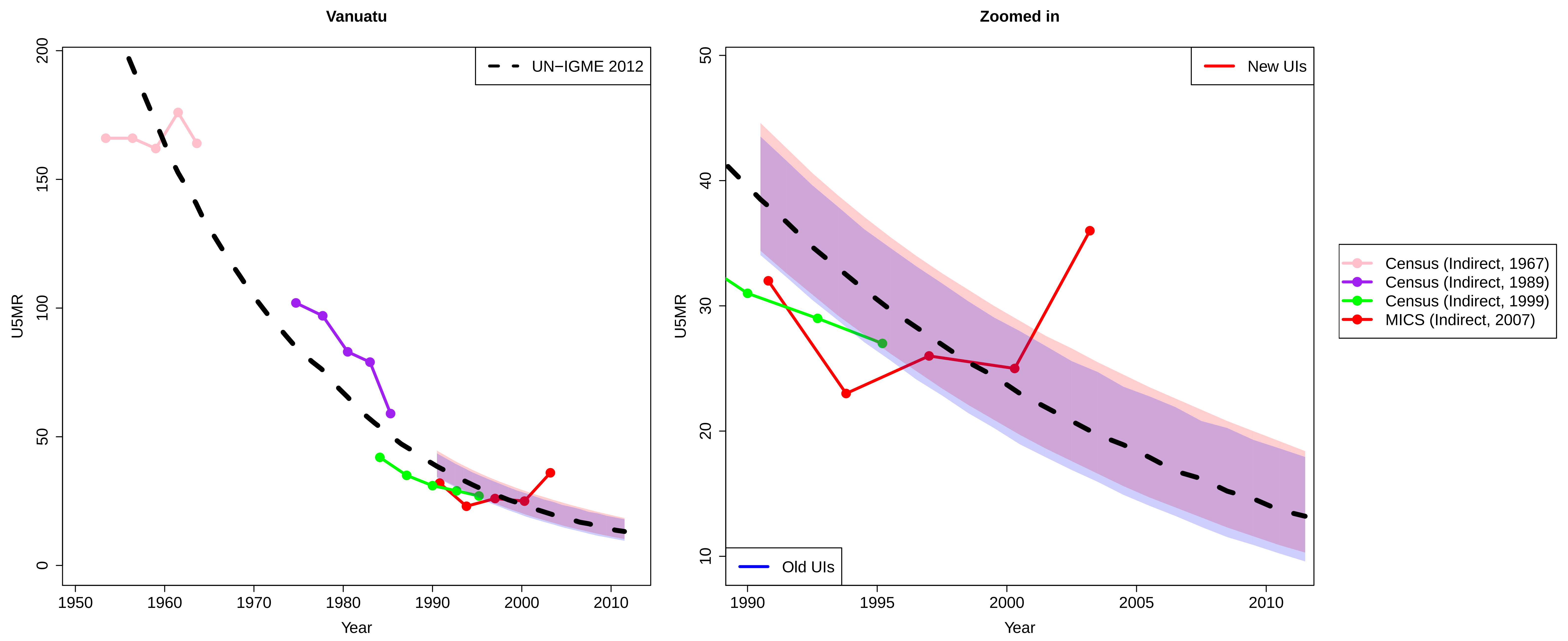

Vanuatu
Zoomed in
UN−IGME 2012
New UIs
Old UIs
Census (Indirect, 1967)
Census (Indirect, 1989)
Census (Indirect, 1999)
MICS (Indirect, 2007)
U5MR
Year
0
50
100
150
200
1950
1960
1970
1980
1990
2000
2010
10
20
30
40
50
1995
2005

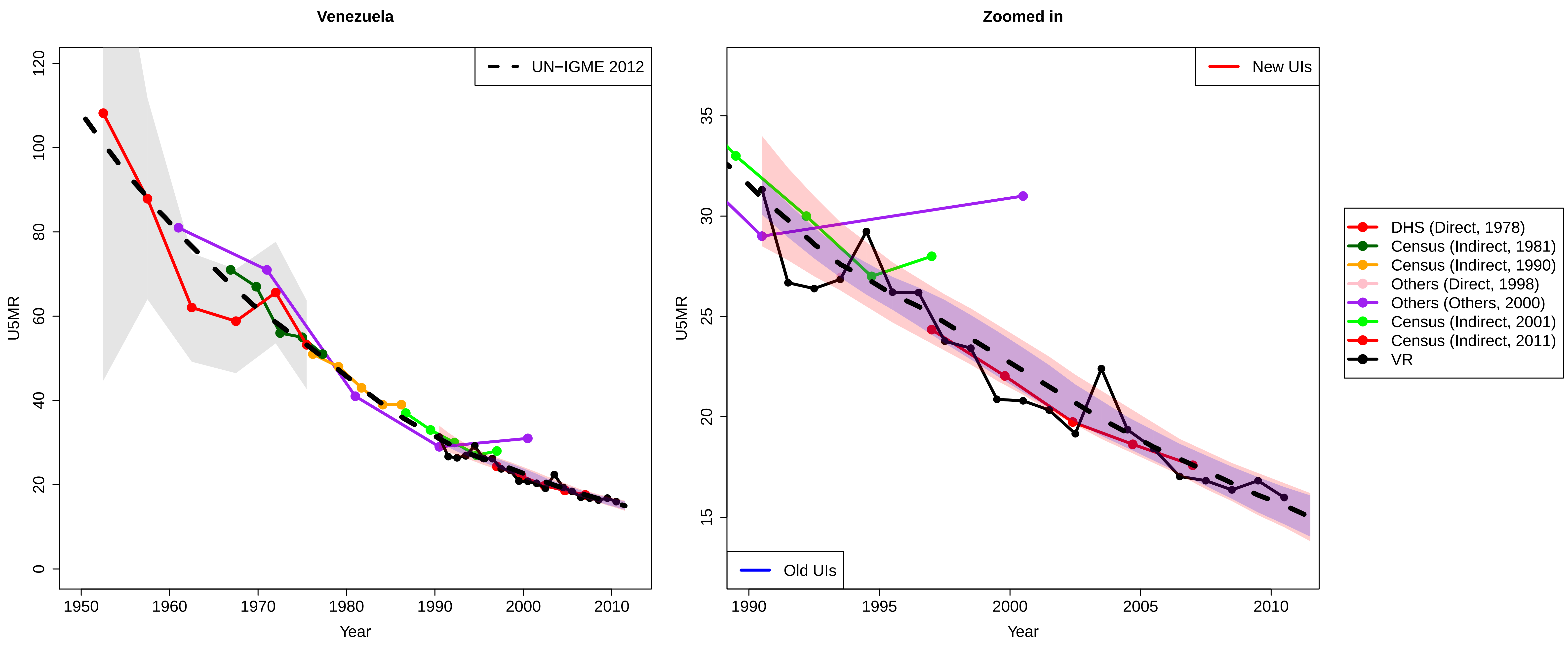

Venezuela
Zoomed in
UN−IGME 2012
New UIs
Old UIs
U5MR
Year
DHS (Direct, 1978)
Census (Indirect, 1981)
Census (Indirect, 1990)
Others (Direct, 1998)
Others (Others, 2000)
Census (Indirect, 2001)
Census (Indirect, 2011)
VR

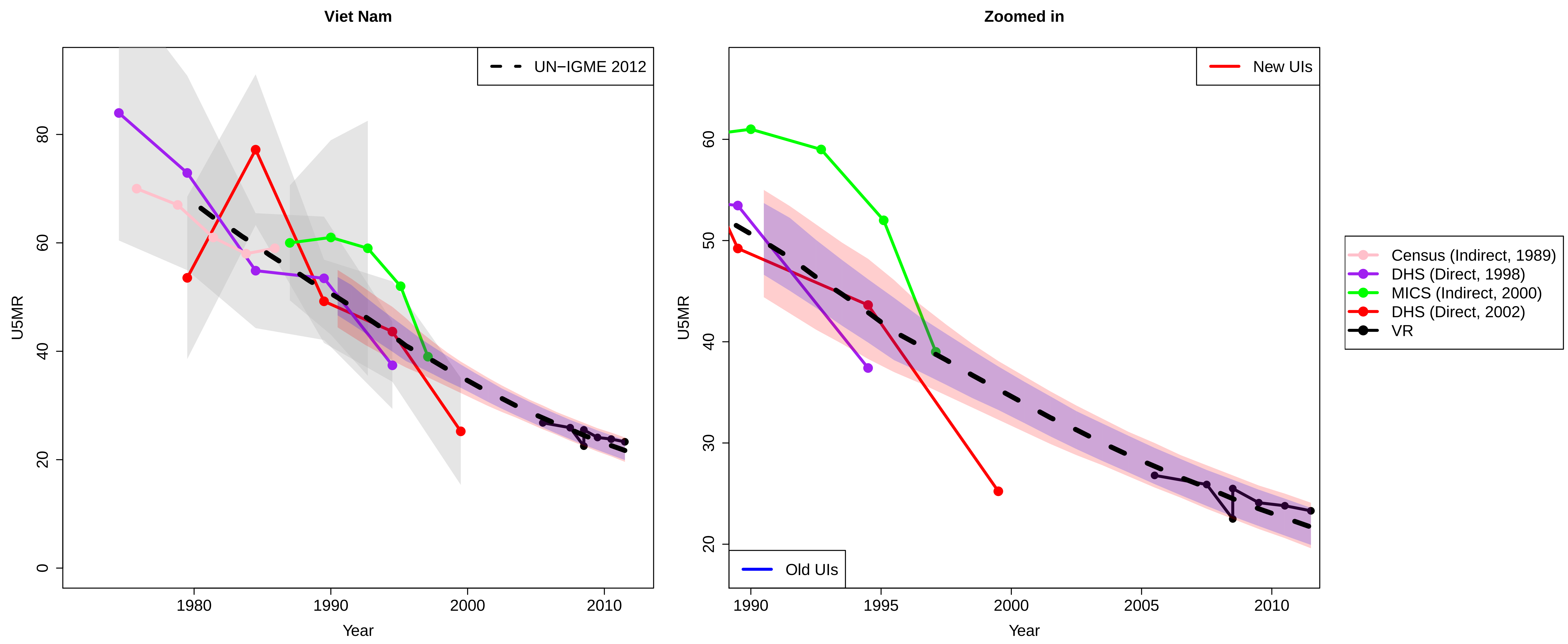
Viet Nam
Zoomed in
UN−IGME 2012
New UIs
Old UIs
Census (Indirect, 1989)
DHS (Direct, 1998)
MICS (Indirect, 2000)
DHS (Direct, 2002)
VR
U5MR
Year
0
20
40
60
80
30
50
1980
1990
2000
2010
1995
2005

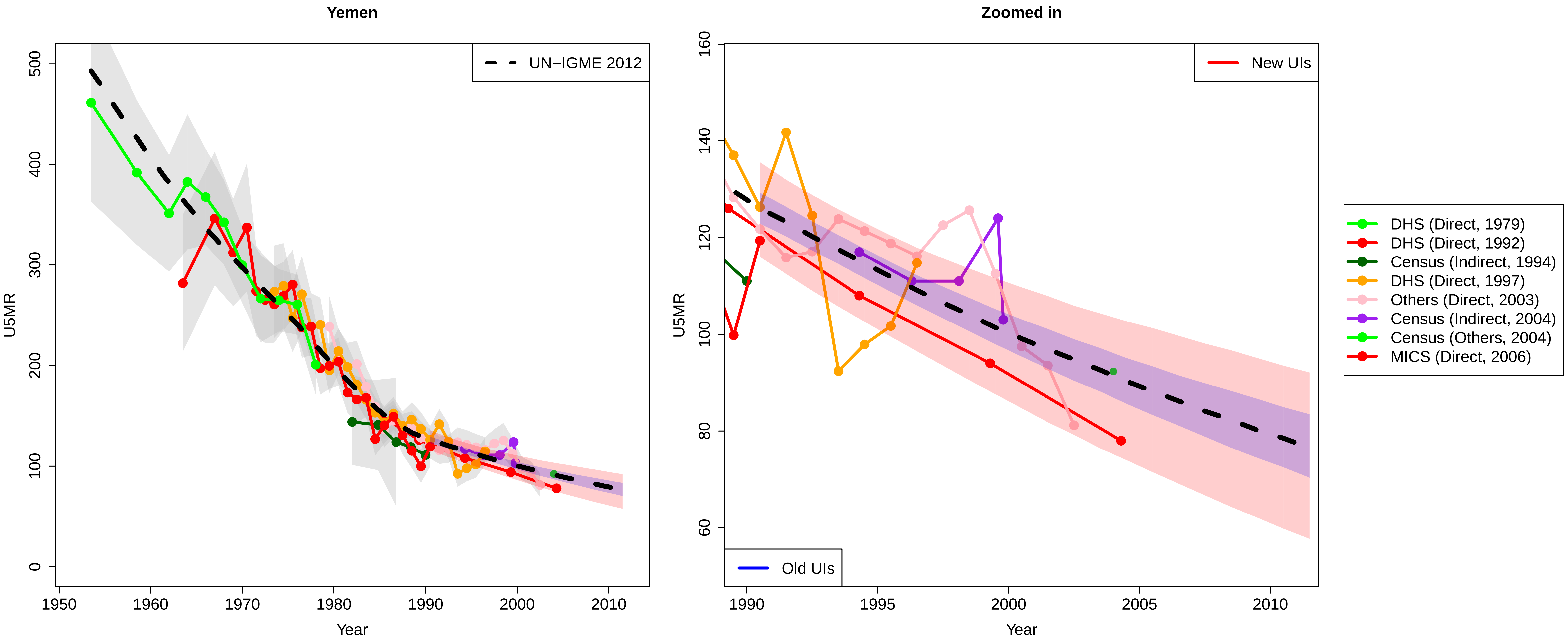
Yemen
UN−IGME 2012
U5MR
0
100
200
300
400
500
1950
1960
1970
1980
1990
2000
2010
Year
Zoomed in
New UIs
Old UIs
U5MR
60
80
100
120
140
160
1990
1995
2000
2005
2010
Year
DHS (Direct, 1979)
DHS (Direct, 1992)
Census (Indirect, 1994)
DHS (Direct, 1997)
Others (Direct, 2003)
Census (Indirect, 2004)
Census (Others, 2004)
MICS (Direct, 2006)

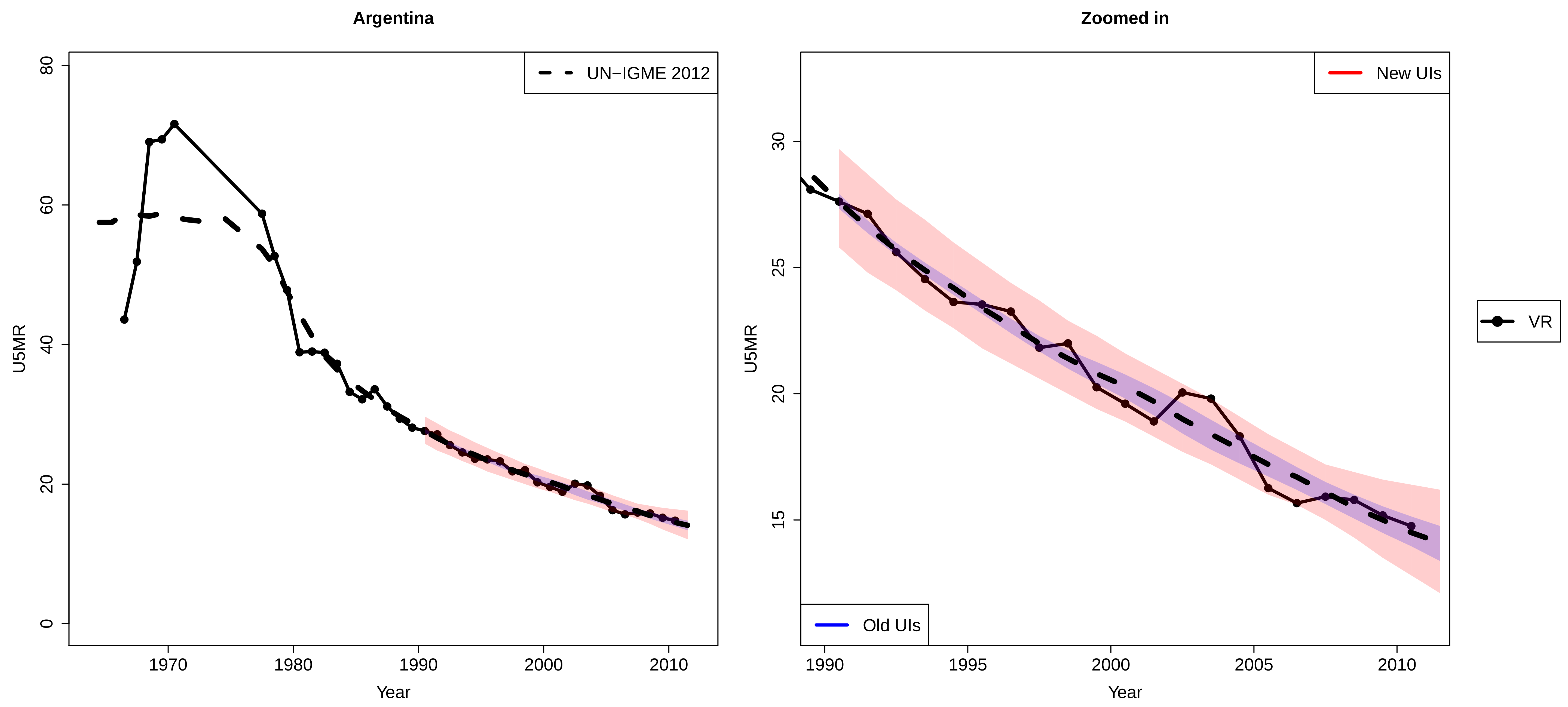
Argentina
Zoomed in
UN−IGME 2012
New UIs
Old UIs
VR
U5MR
Year
0
20
40
60
80
1970
1980
1990
2000
2010
15
20
25
30
1990
1995
2000
2005
2010

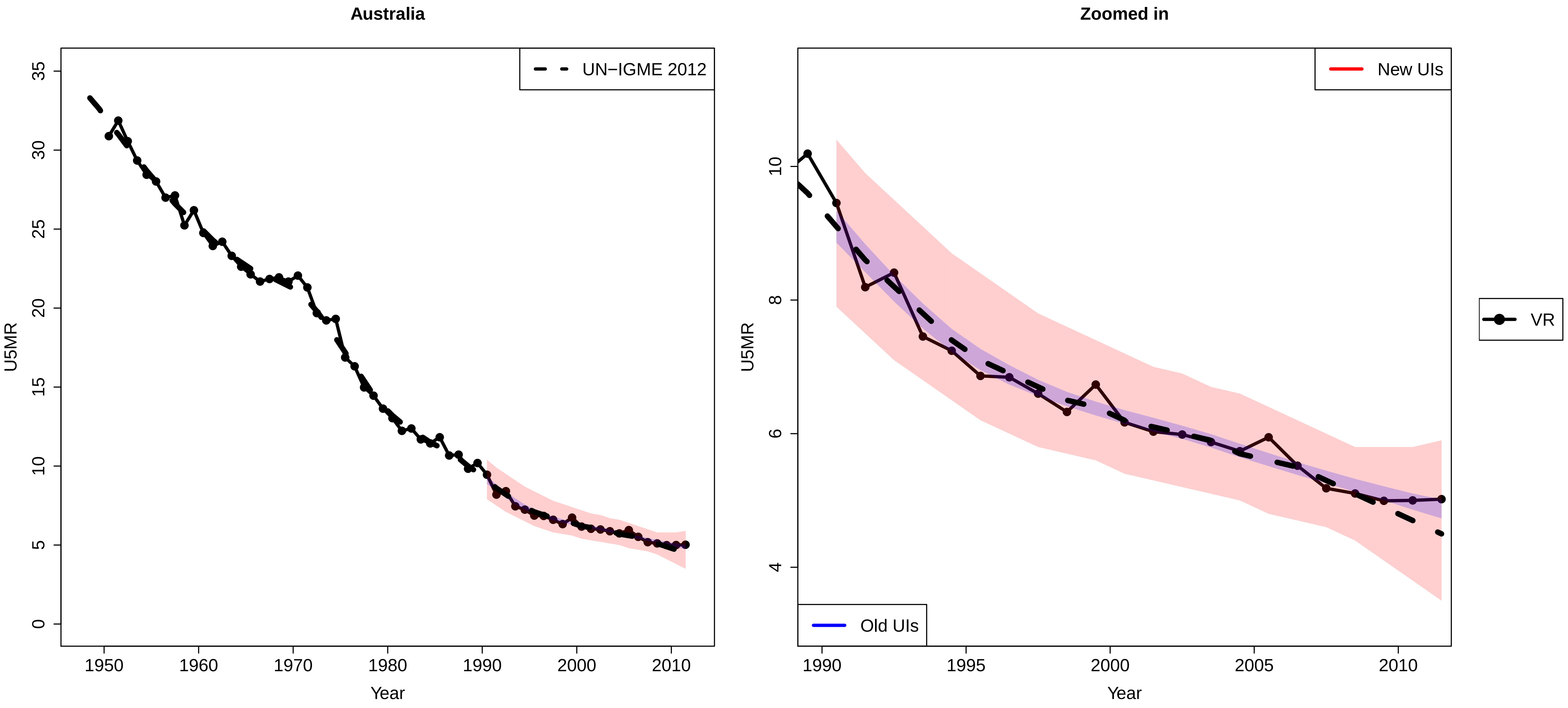

Australia
UN−IGME 2012
U5MR
Year
Zoomed in
New UIs
Old UIs
VR

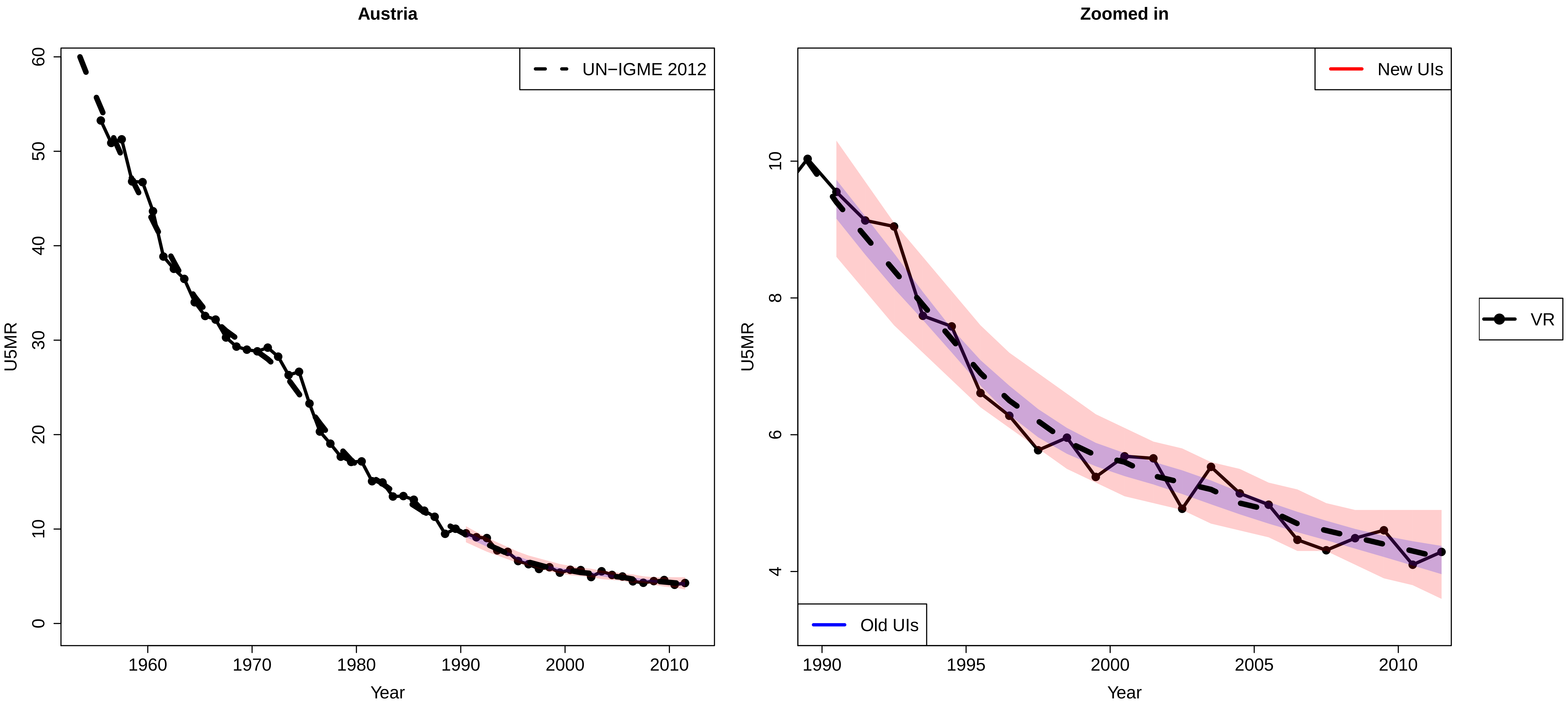

Austria
UN−IGME 2012
U5MR
Year
0
10
20
30
40
50
60
1960
1970
1980
1990
2000
2010
Zoomed in
New UIs
Old UIs
4
6
8
10
1990
1995
2000
2005
2010
VR

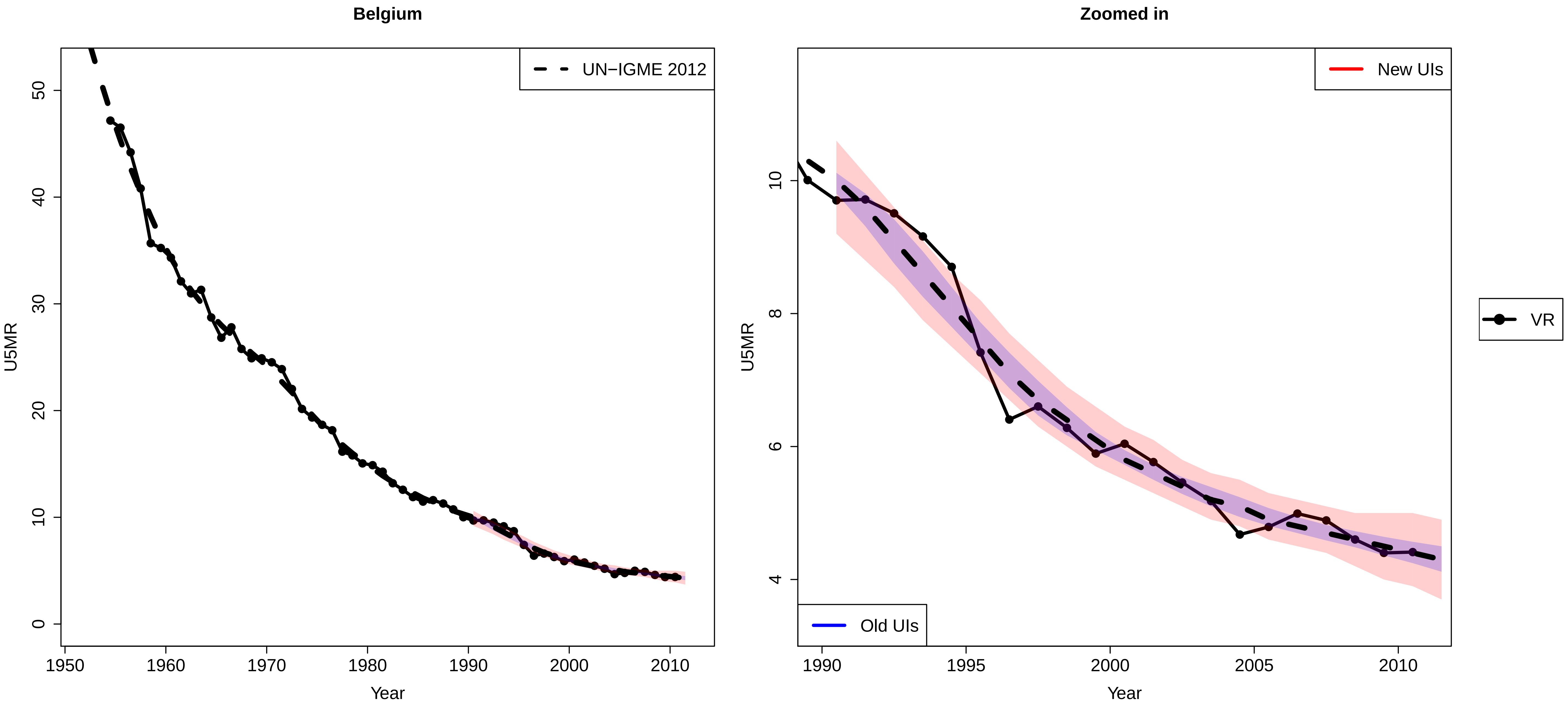

Belgium
UN−IGME 2012
U5MR
Year
Zoomed in
New UIs
Old UIs
VR

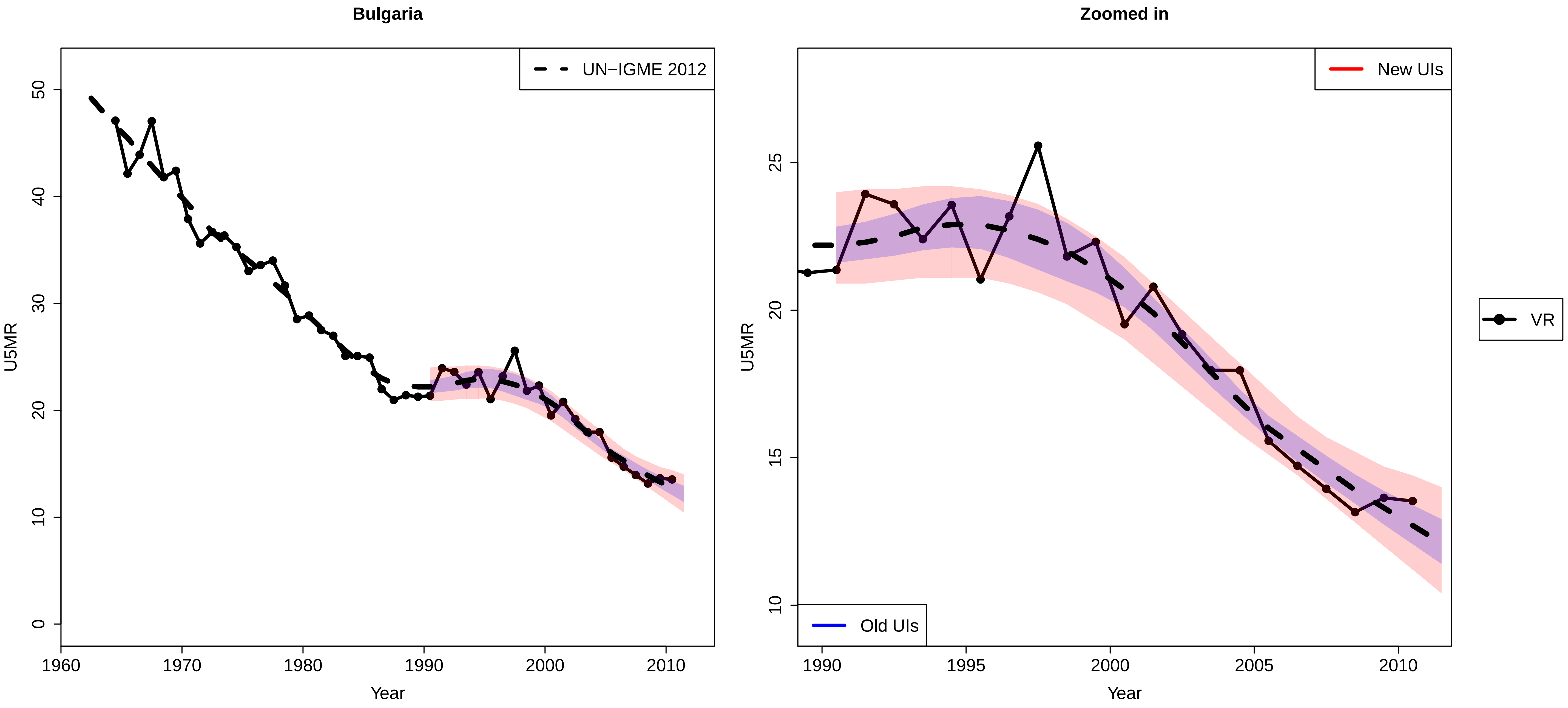

Bulgaria
Zoomed in
UN−IGME 2012
New UIs
Old UIs
VR
U5MR
Year
0
10
20
30
40
50
1960
1970
1980
1990
2000
2010
15
25
1995
2005

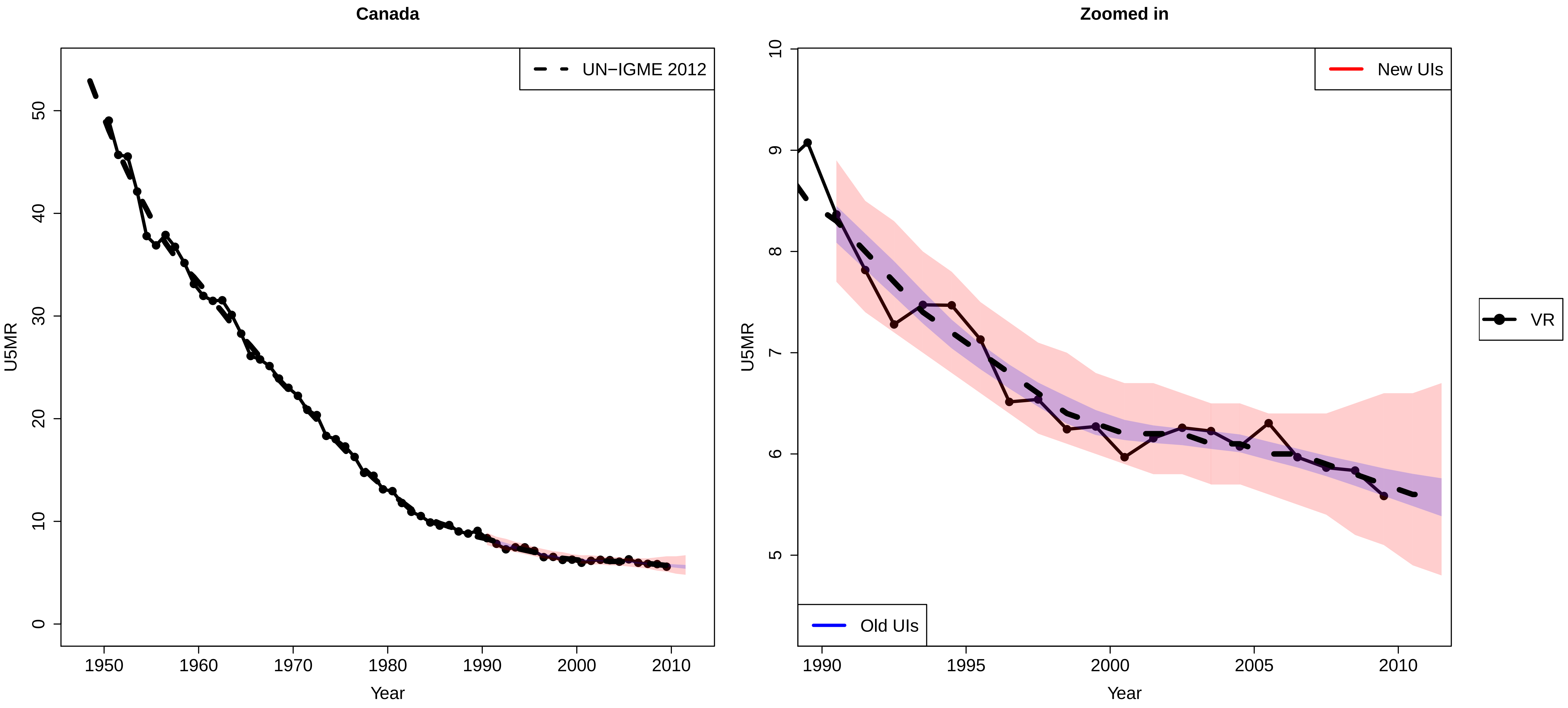

Canada
UN−IGME 2012
U5MR
Year
Zoomed in
New UIs
Old UIs
VR

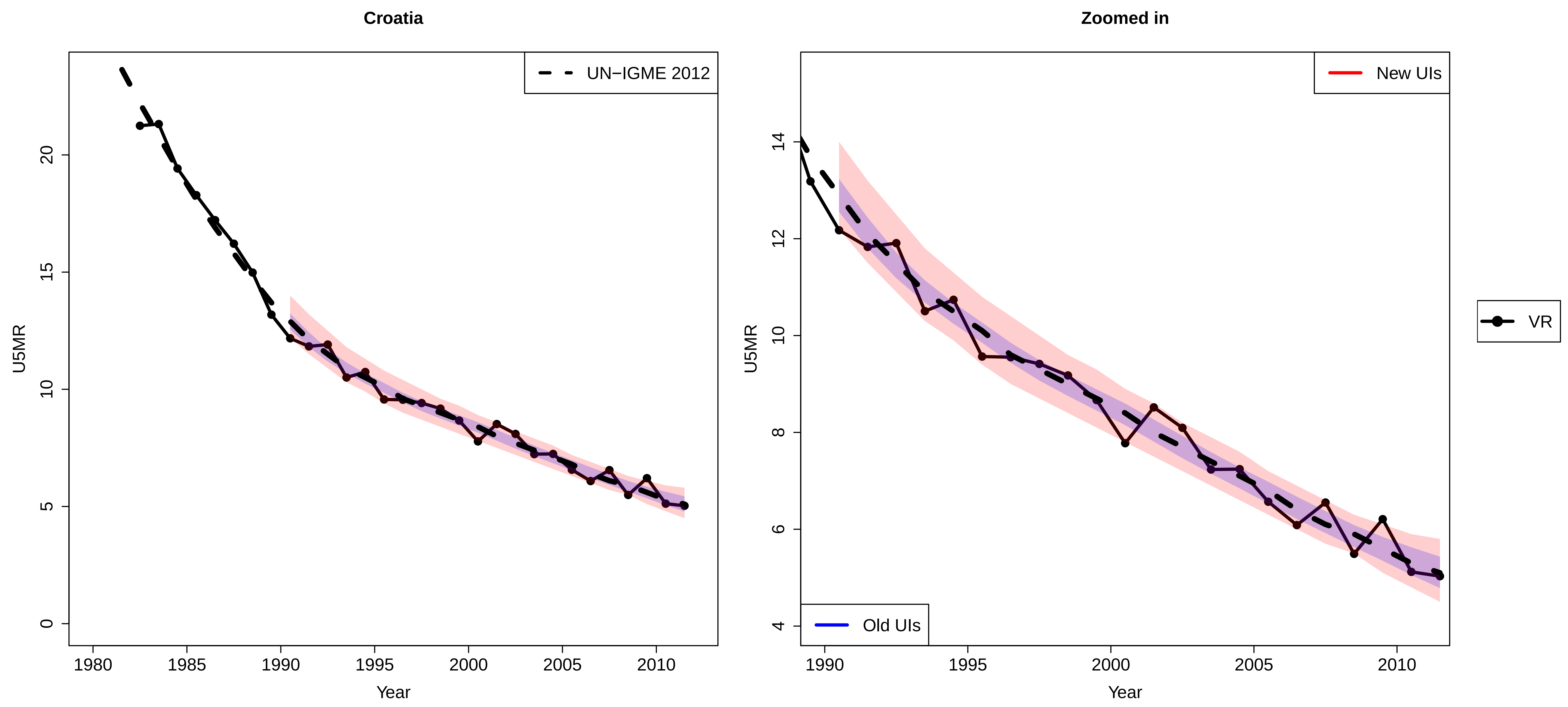

Croatia
UN−IGME 2012
U5MR
Year
Zoomed in
New UIs
Old UIs
VR

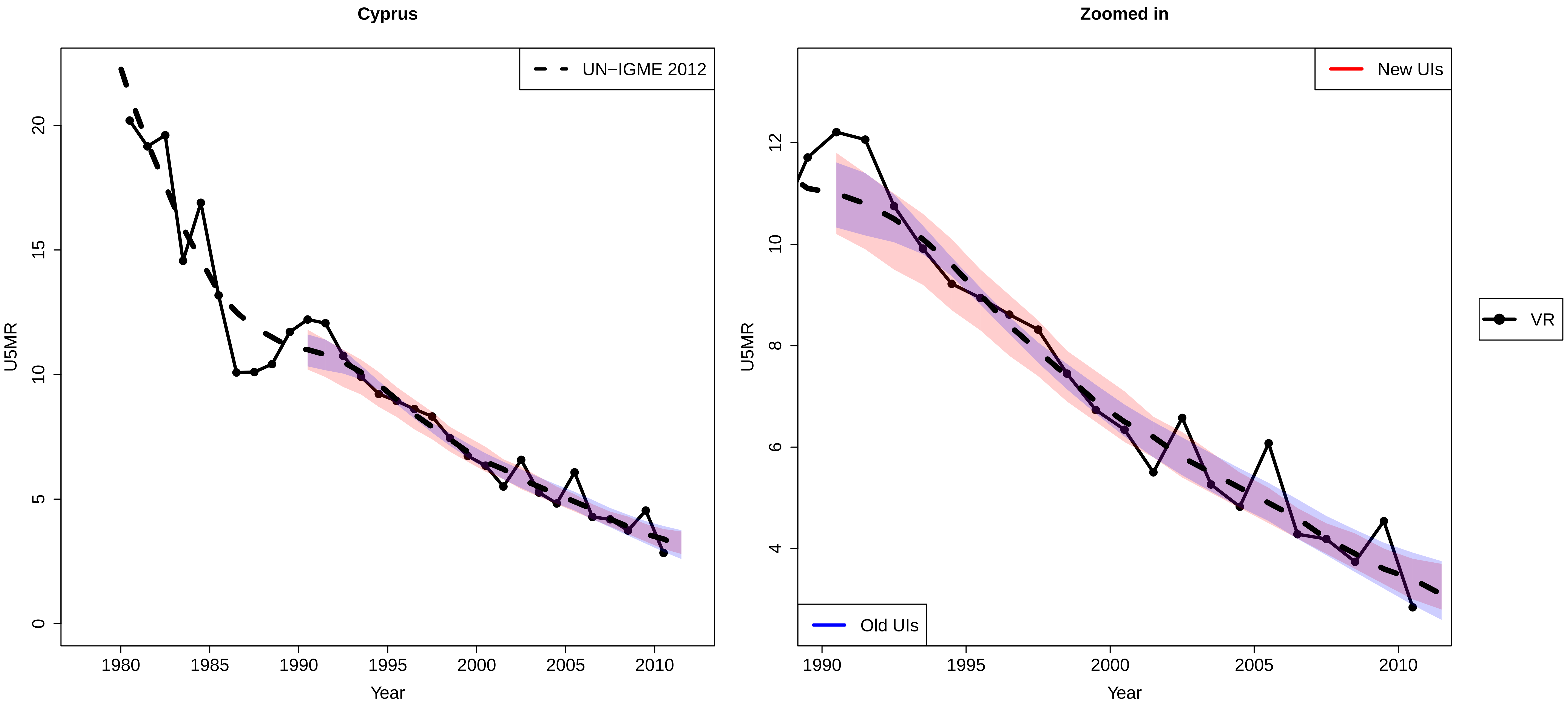

Cyprus
UN−IGME 2012
U5MR
Year
Zoomed in
New UIs
Old UIs
VR

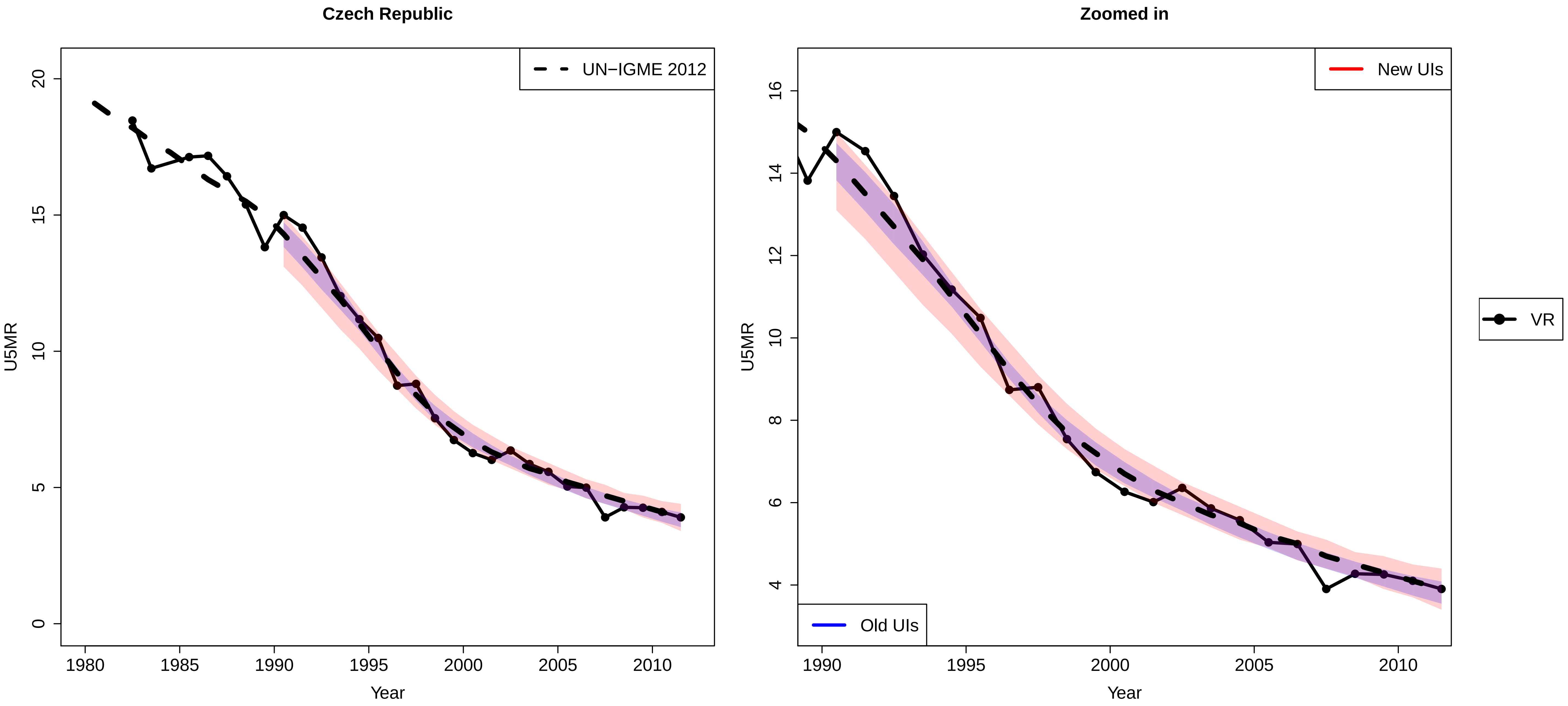

Czech Republic
Zoomed in
UN−IGME 2012
New UIs
Old UIs
VR
U5MR
Year
0
5
10
15
20
4
6
8
10
12
14
16
1980
1985
1990
1995
2000
2005
2010

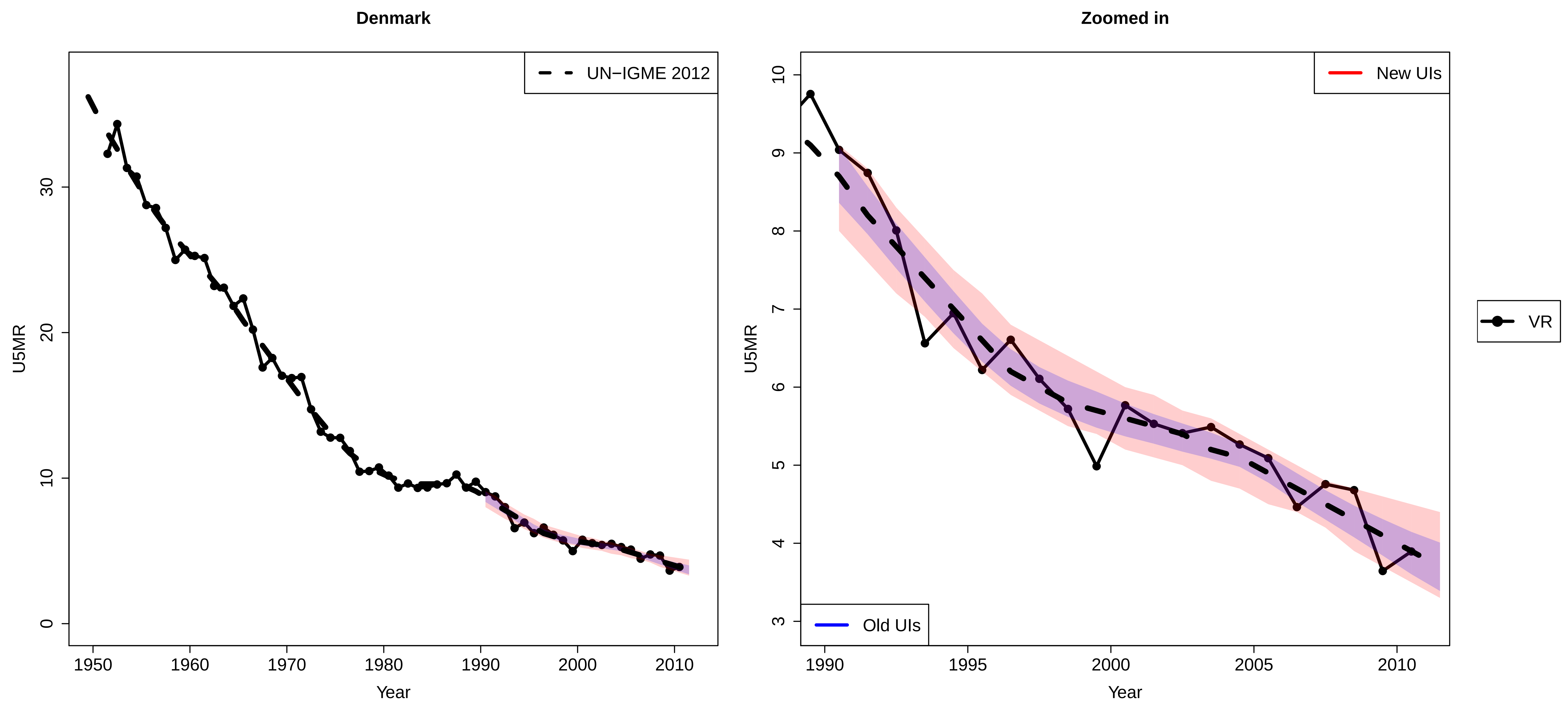

Denmark
UN−IGME 2012
U5MR
Year
Zoomed in
New UIs
Old UIs
VR

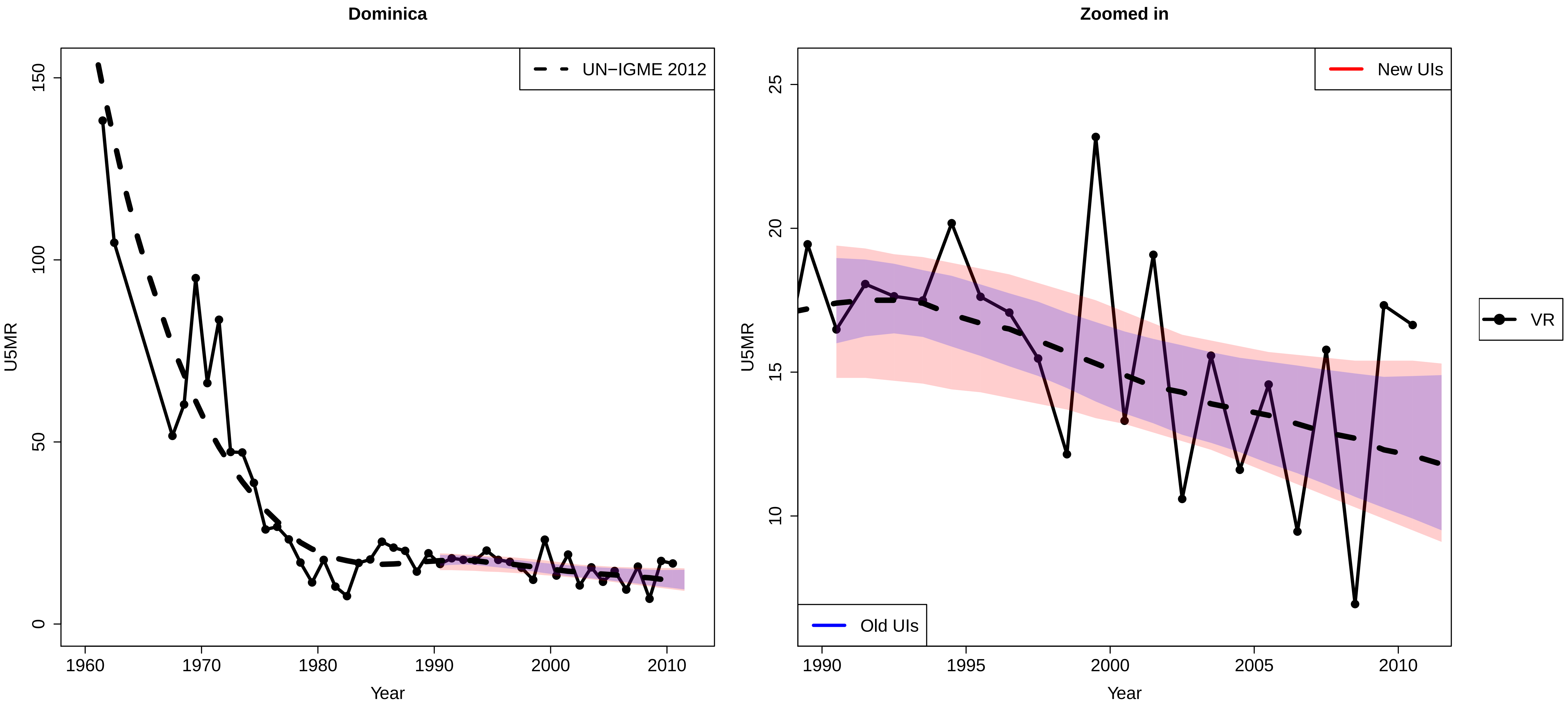

Dominica
UN−IGME 2012
U5MR
Year
Zoomed in
New UIs
Old UIs
VR

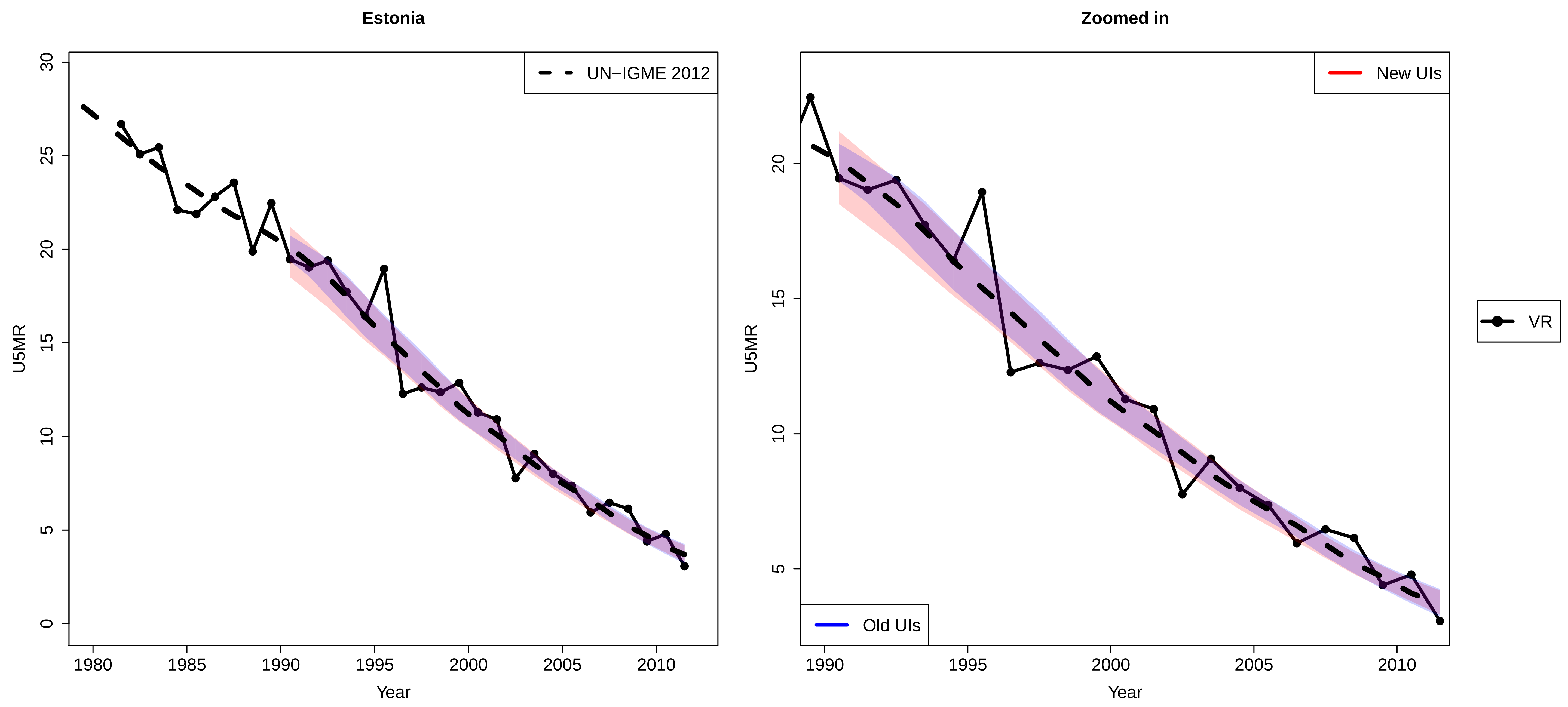
Estonia
Zoomed in
UN−IGME 2012
New UIs
Old UIs
VR
U5MR
Year

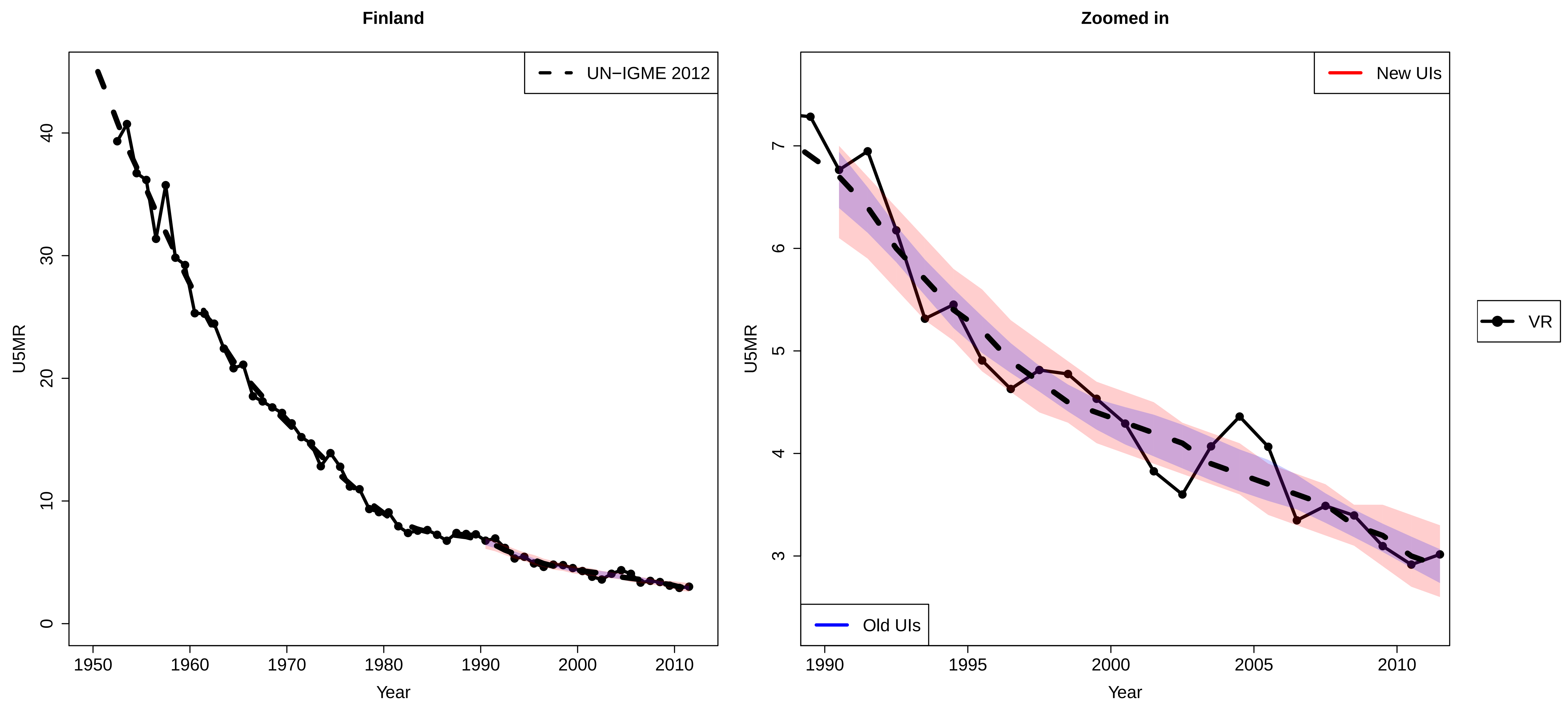

Finland
UN−IGME 2012
U5MR
Year
Zoomed in
New UIs
Old UIs
VR

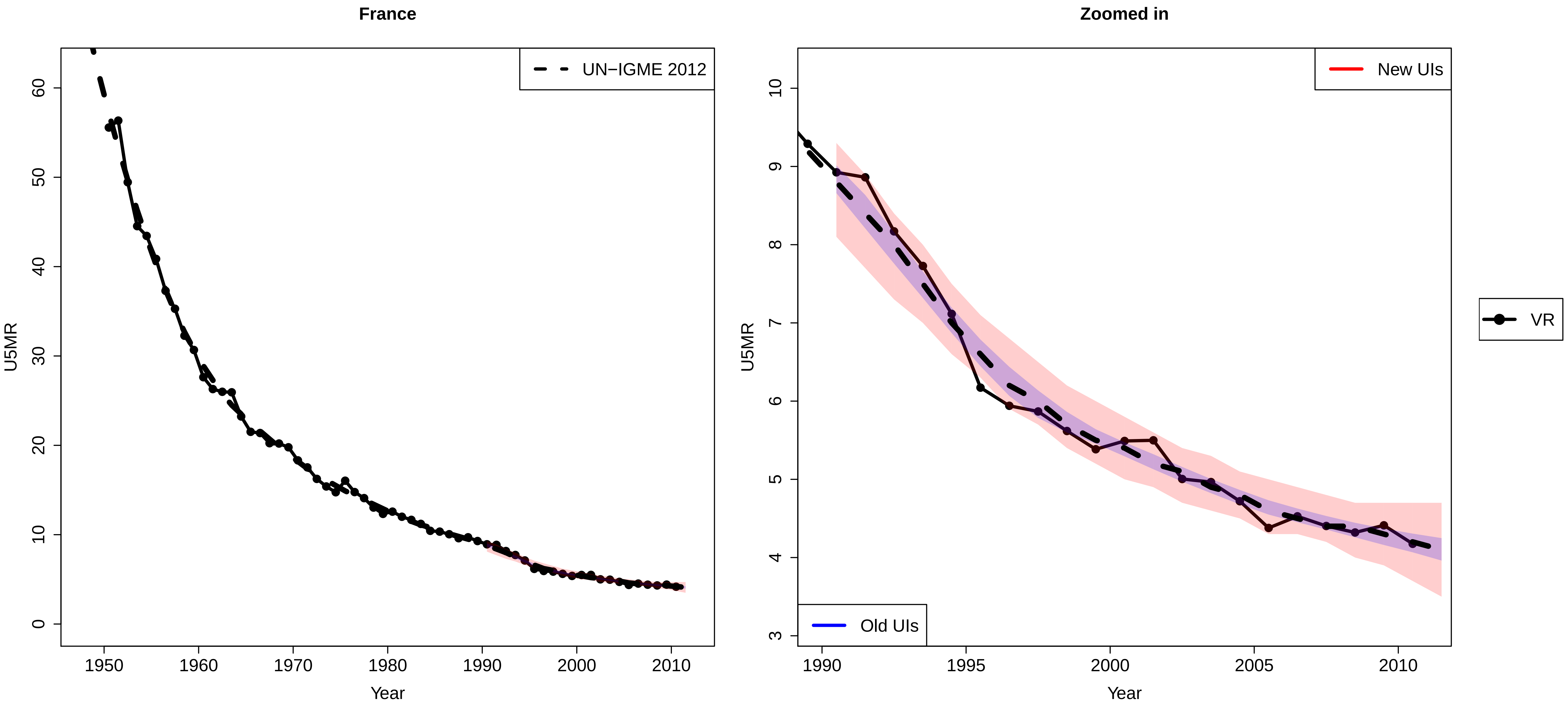
France
UN−IGME 2012
U5MR
Year
0
10
20
30
40
50
60
1950
1960
1970
1980
1990
2000
2010
Zoomed in
New UIs
Old UIs
VR
3
4
5
6
7
8
9
10
1990
1995
2000
2005
2010

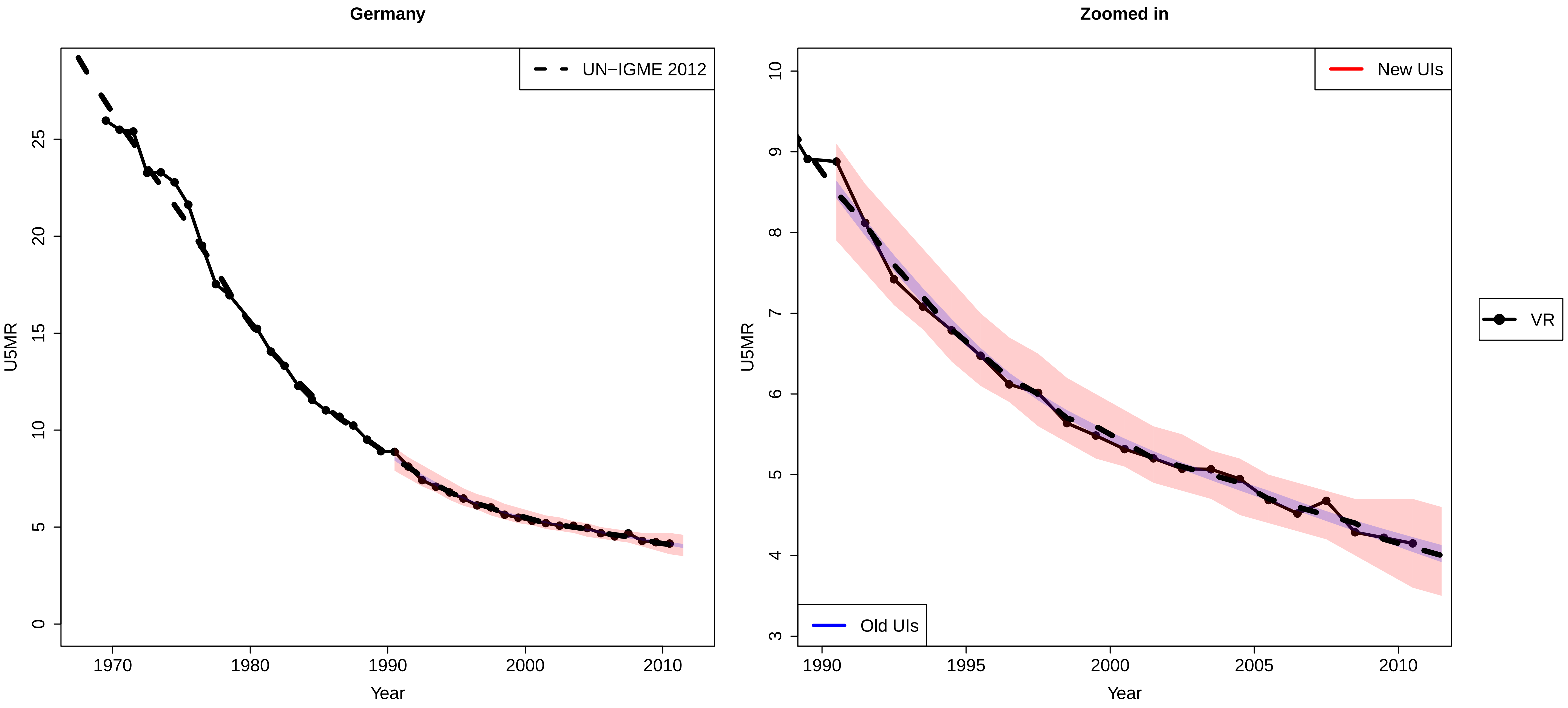
Germany
UN−IGME 2012
U5MR
Year
0
5
10
15
20
25
1970
1980
1990
2000
2010
Zoomed in
New UIs
Old UIs
3
4
5
6
7
8
9
10
1990
1995
2000
2005
2010
VR

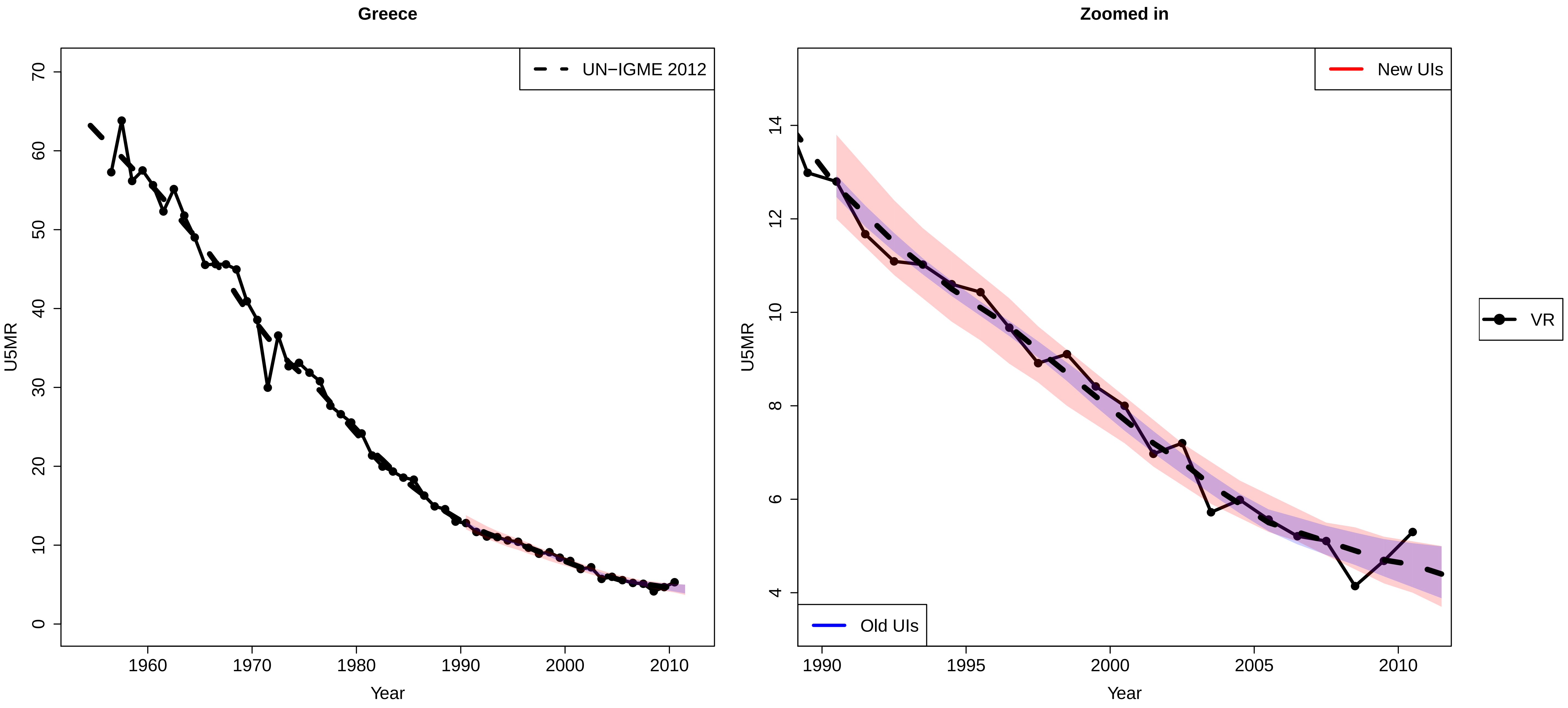

Greece
Zoomed in
UN−IGME 2012
New UIs
Old UIs
VR
U5MR
Year
0
10
20
30
40
50
60
70
1960
1970
1980
1990
2000
2010
4
6
8
10
12
14
1990
1995
2000
2005
2010

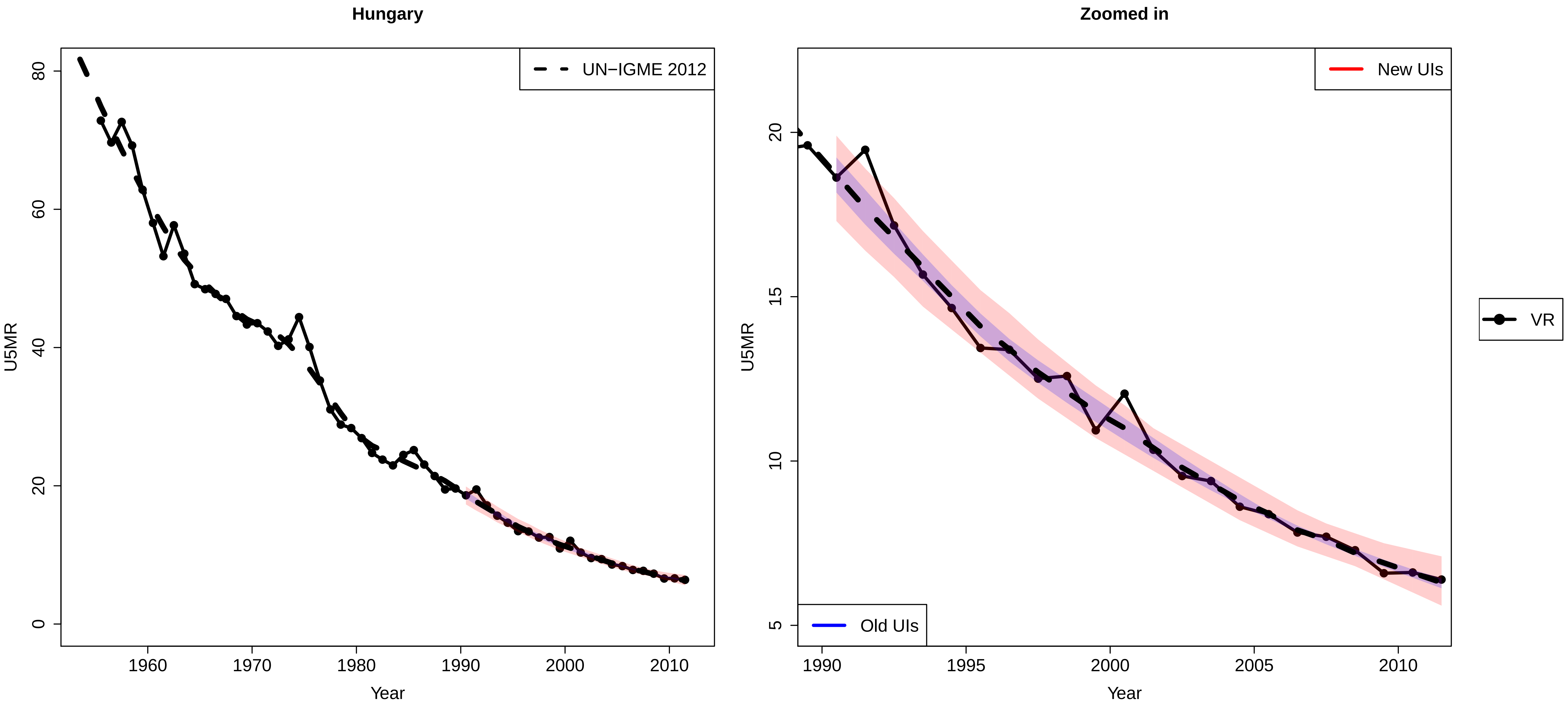
Hungary
UN−IGME 2012
U5MR
Year
Zoomed in
New UIs
Old UIs
VR
U5MR
Year

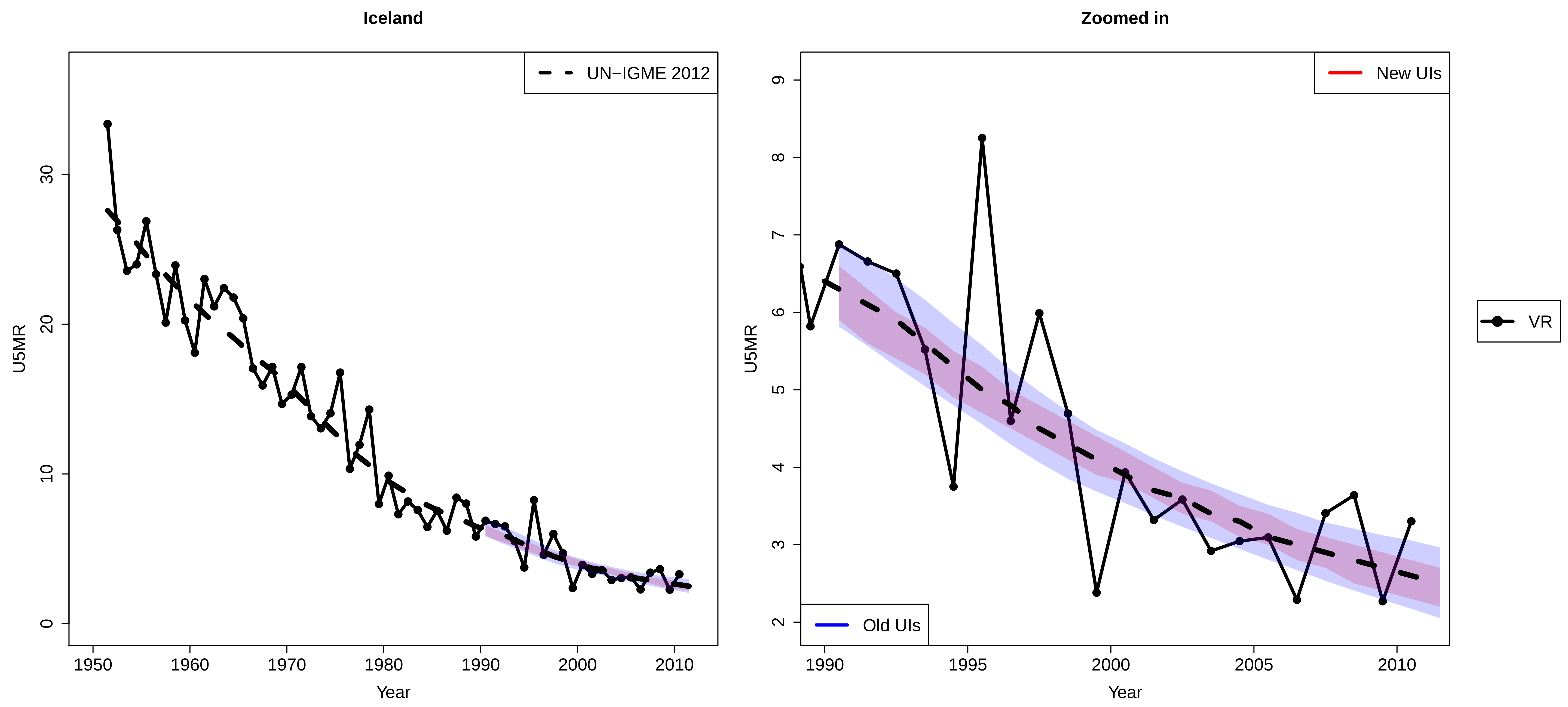

Iceland
UN−IGME 2012
U5MR
Year
Zoomed in
New UIs
Old UIs
VR

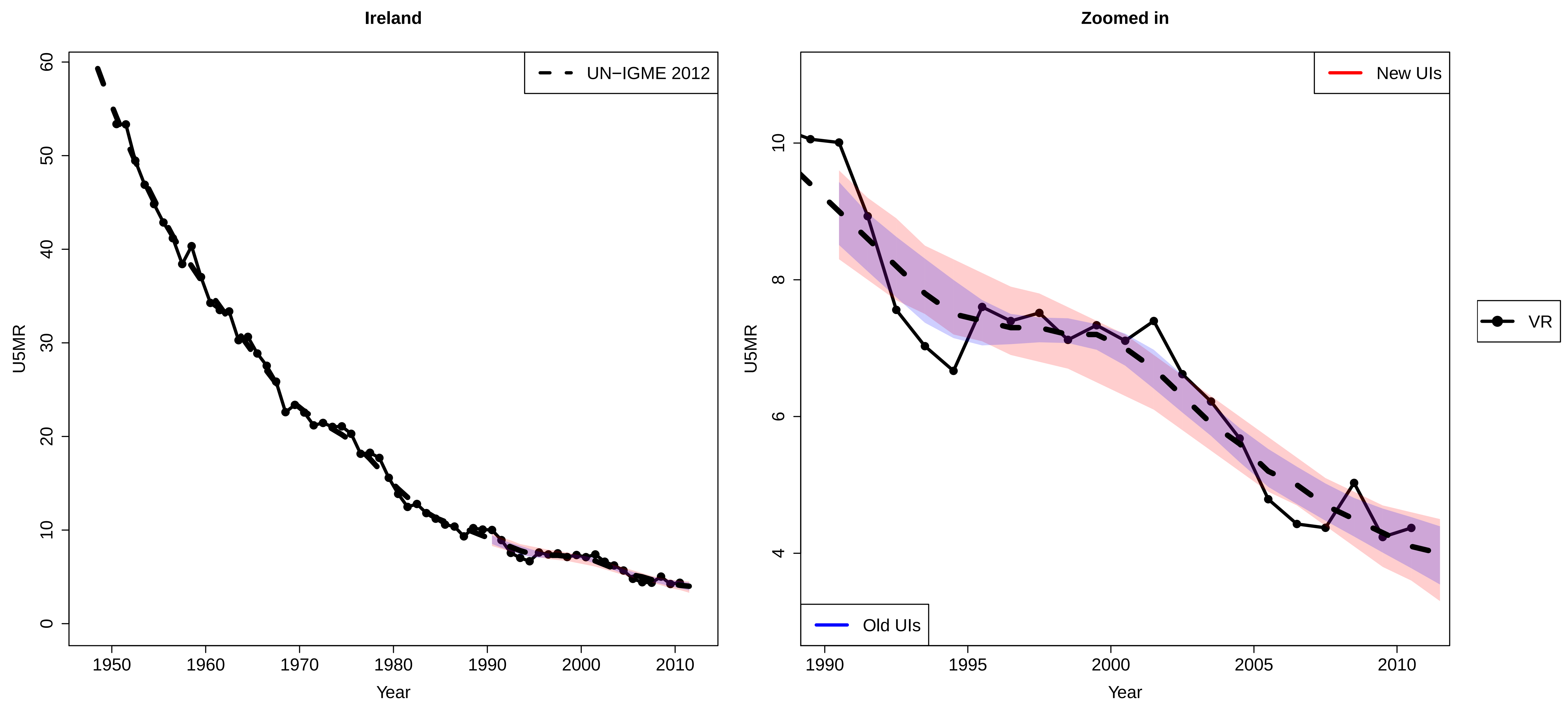

Ireland
UN−IGME 2012
U5MR
Year
Zoomed in
New UIs
Old UIs
VR

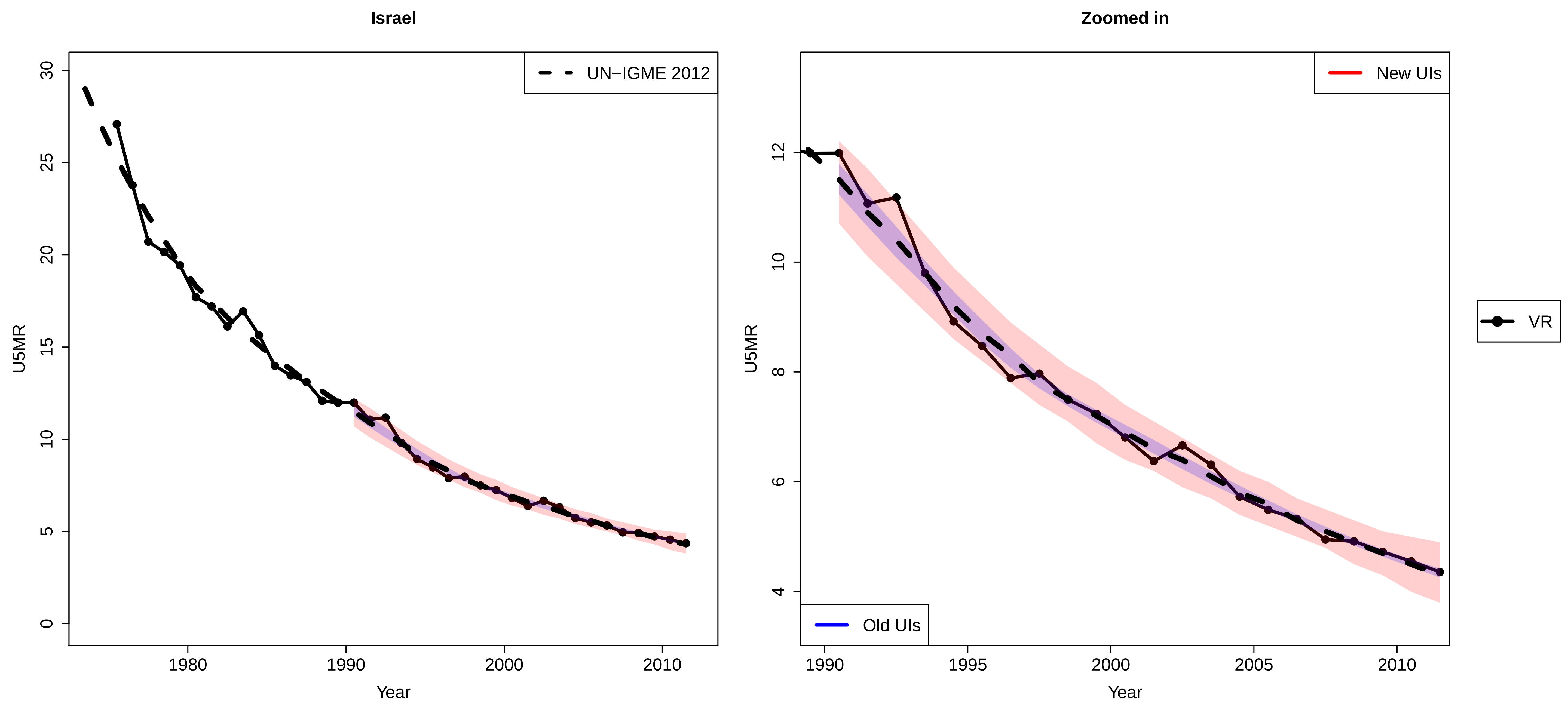

Israel
UN−IGME 2012
U5MR
Year
Zoomed in
New UIs
Old UIs
VR

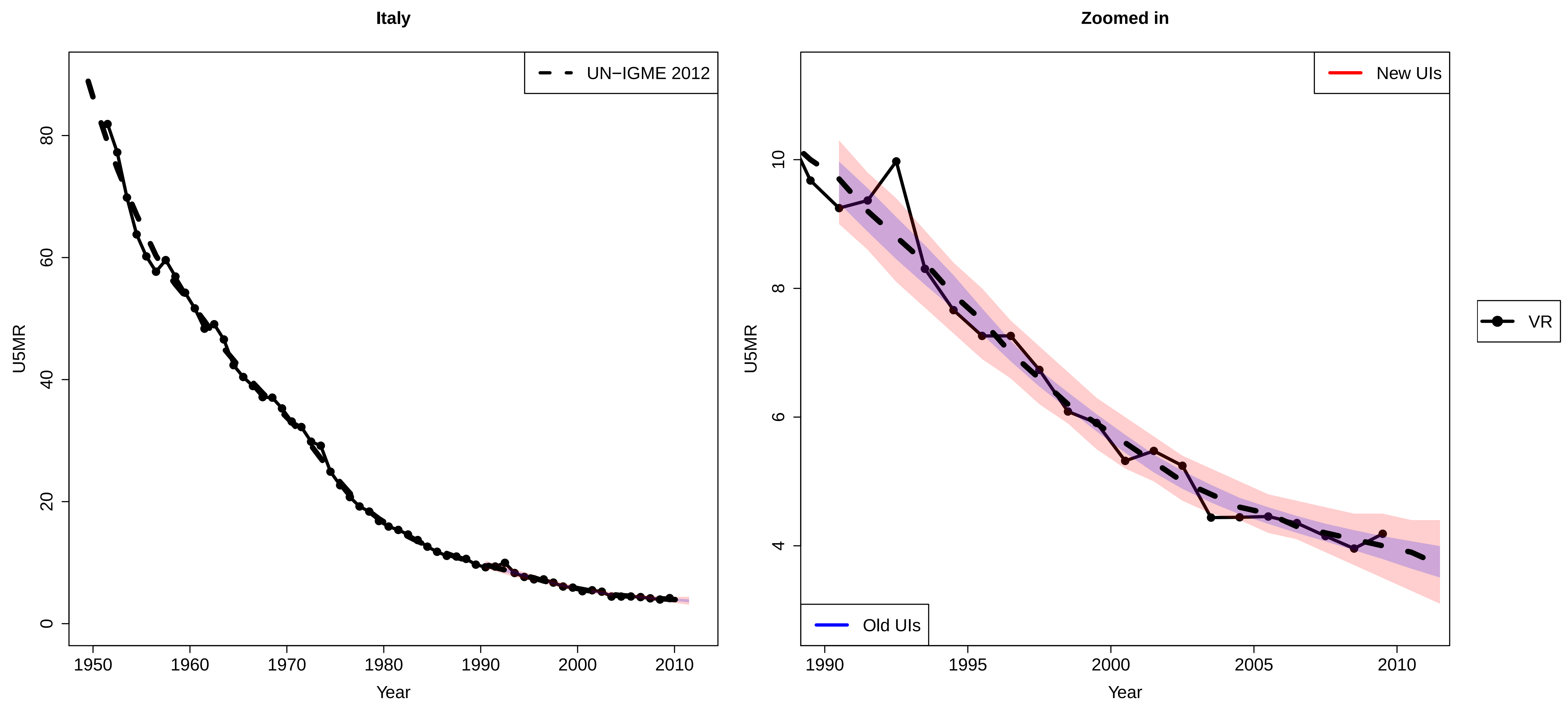

Italy
UN−IGME 2012
U5MR
Year
Zoomed in
New UIs
Old UIs
VR

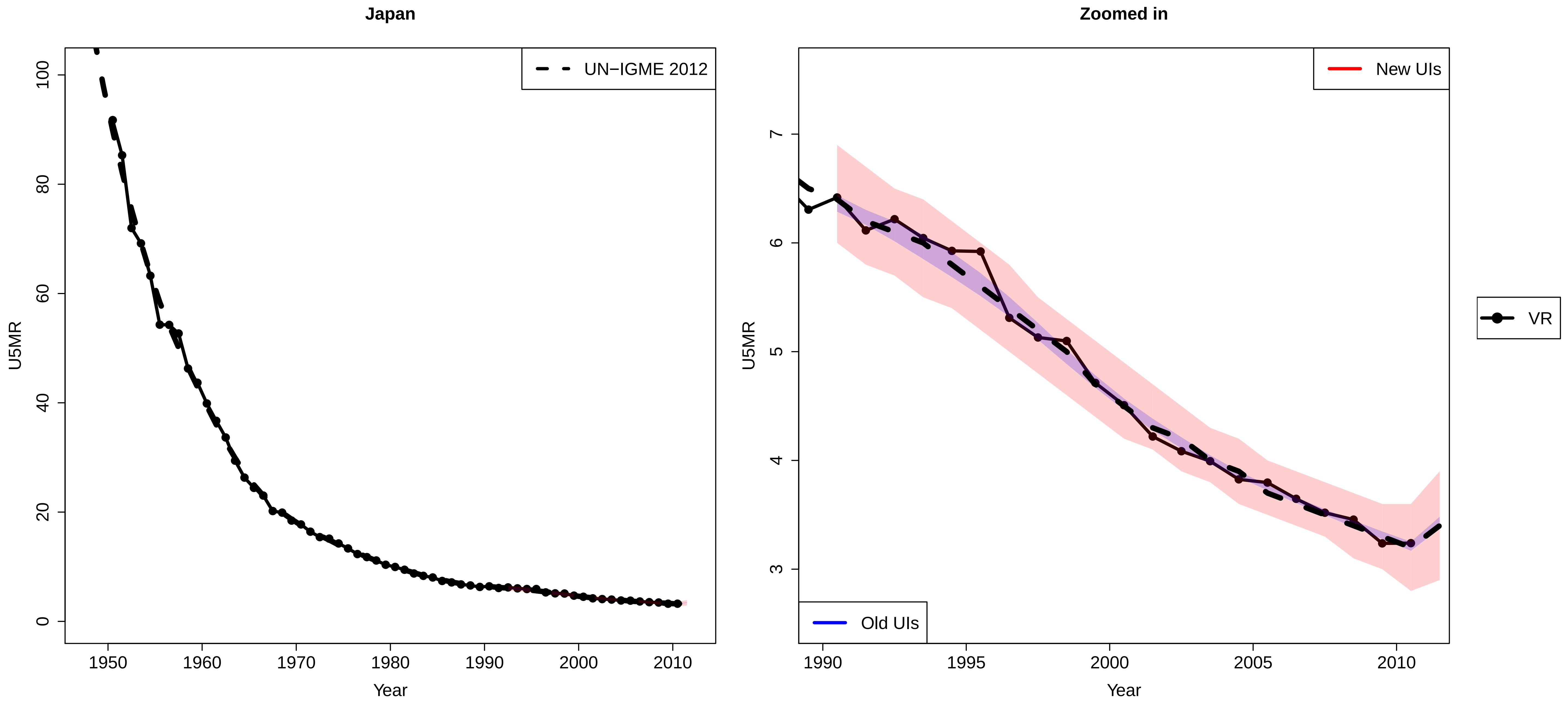
Japan
UN−IGME 2012
U5MR
Year
Zoomed in
New UIs
Old UIs
VR

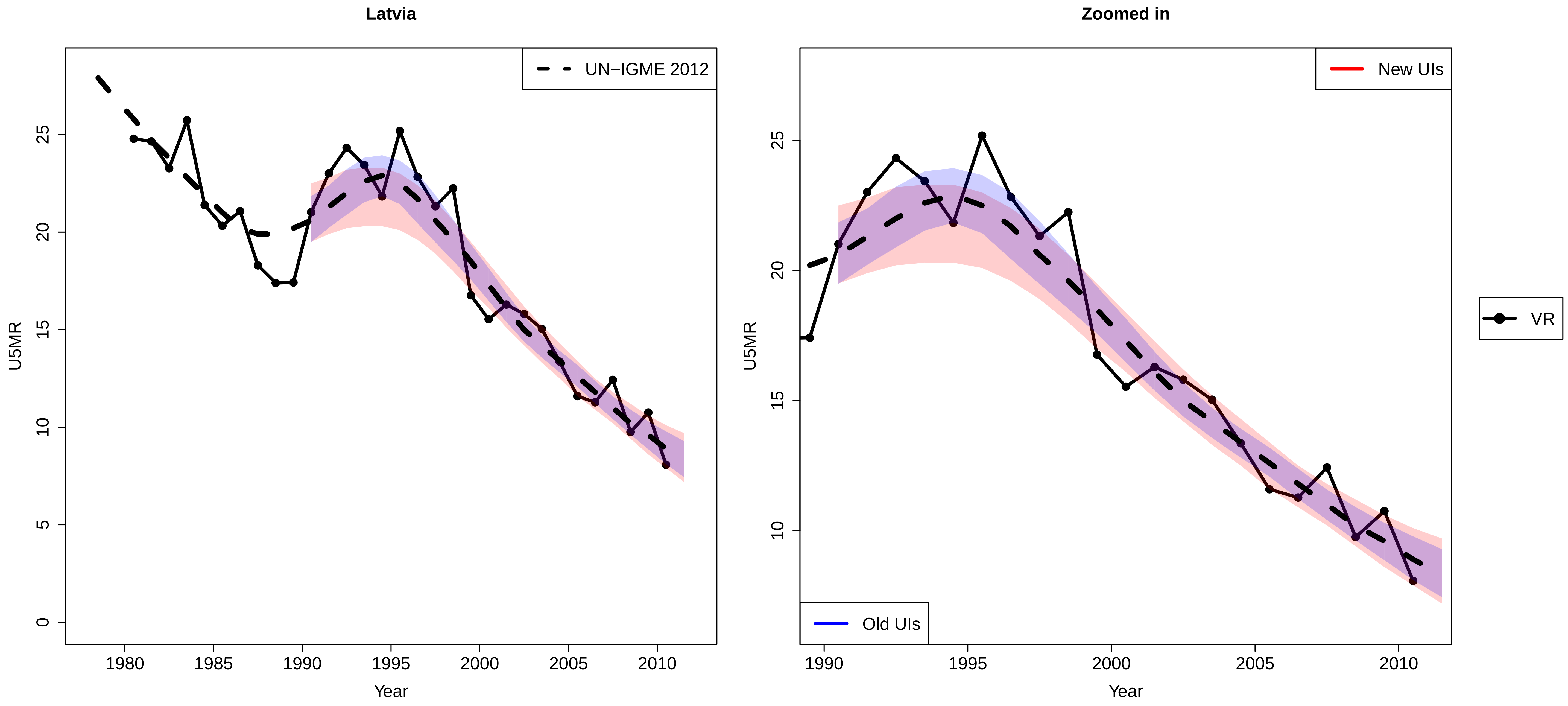

Latvia
Zoomed in
UN−IGME 2012
New UIs
Old UIs
VR
U5MR
Year

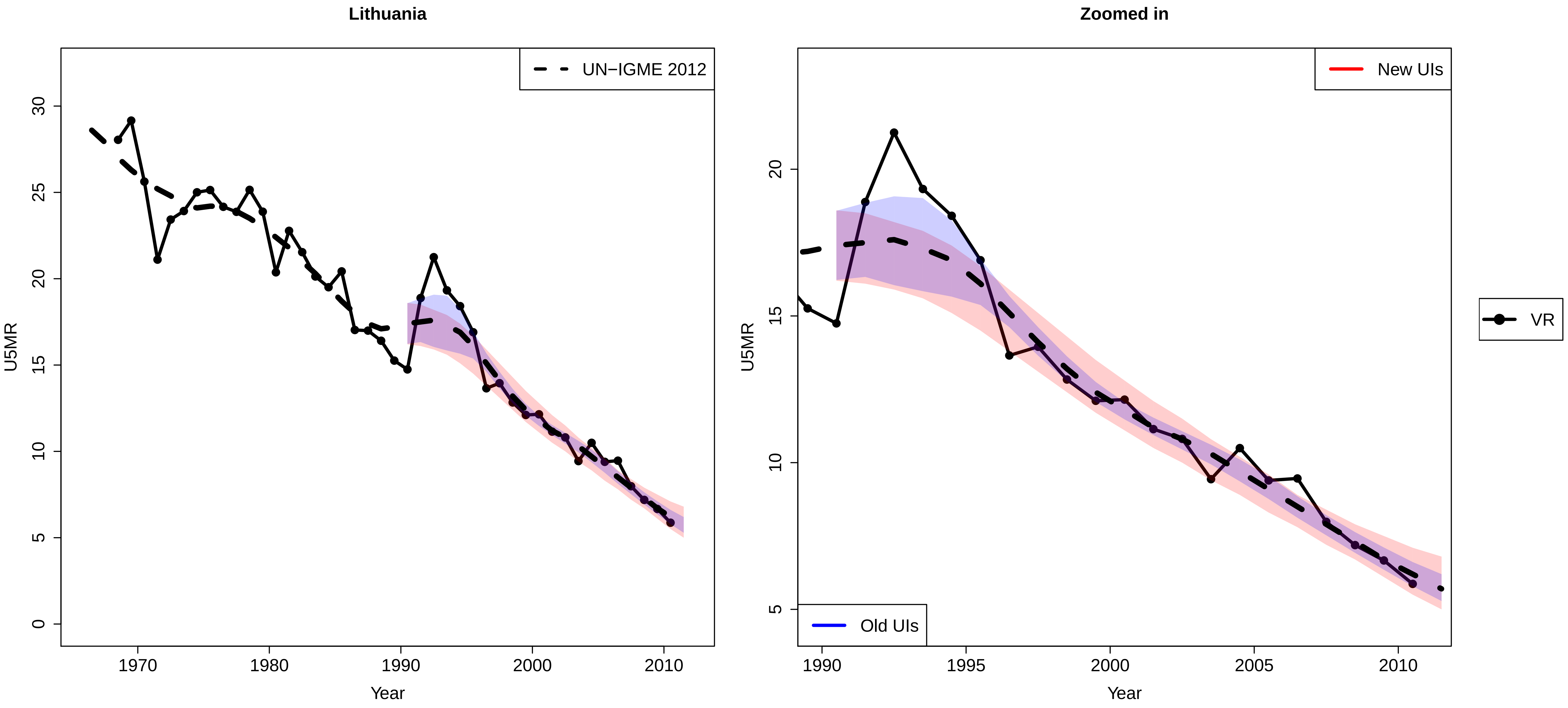
Lithuania
UN−IGME 2012
U5MR
Year
Zoomed in
New UIs
Old UIs
VR

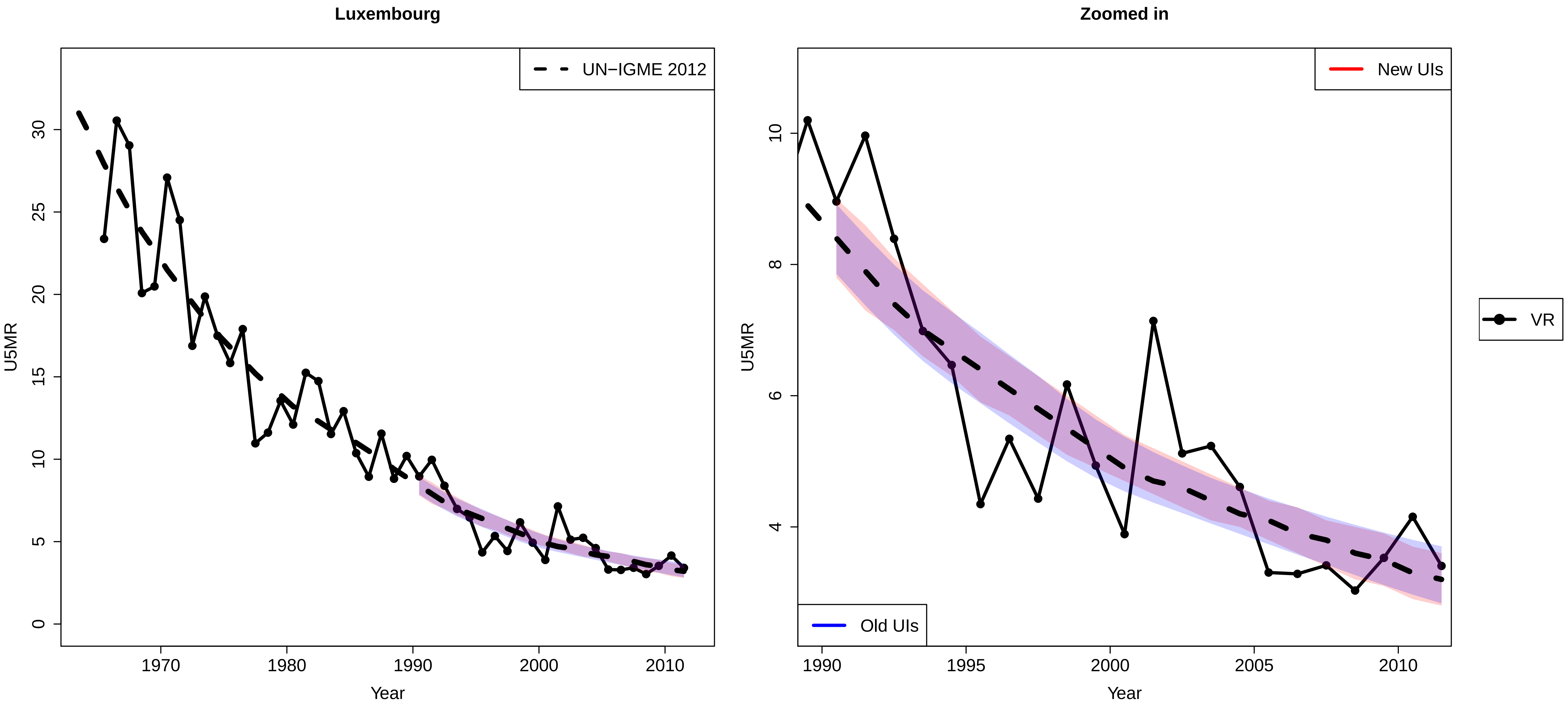

Luxembourg
UN−IGME 2012
U5MR
0
5
10
15
20
25
30
1970
1980
1990
2000
2010
Year
Zoomed in
New UIs
Old UIs
VR
4
6
8
10
1990
1995
2000
2005
2010

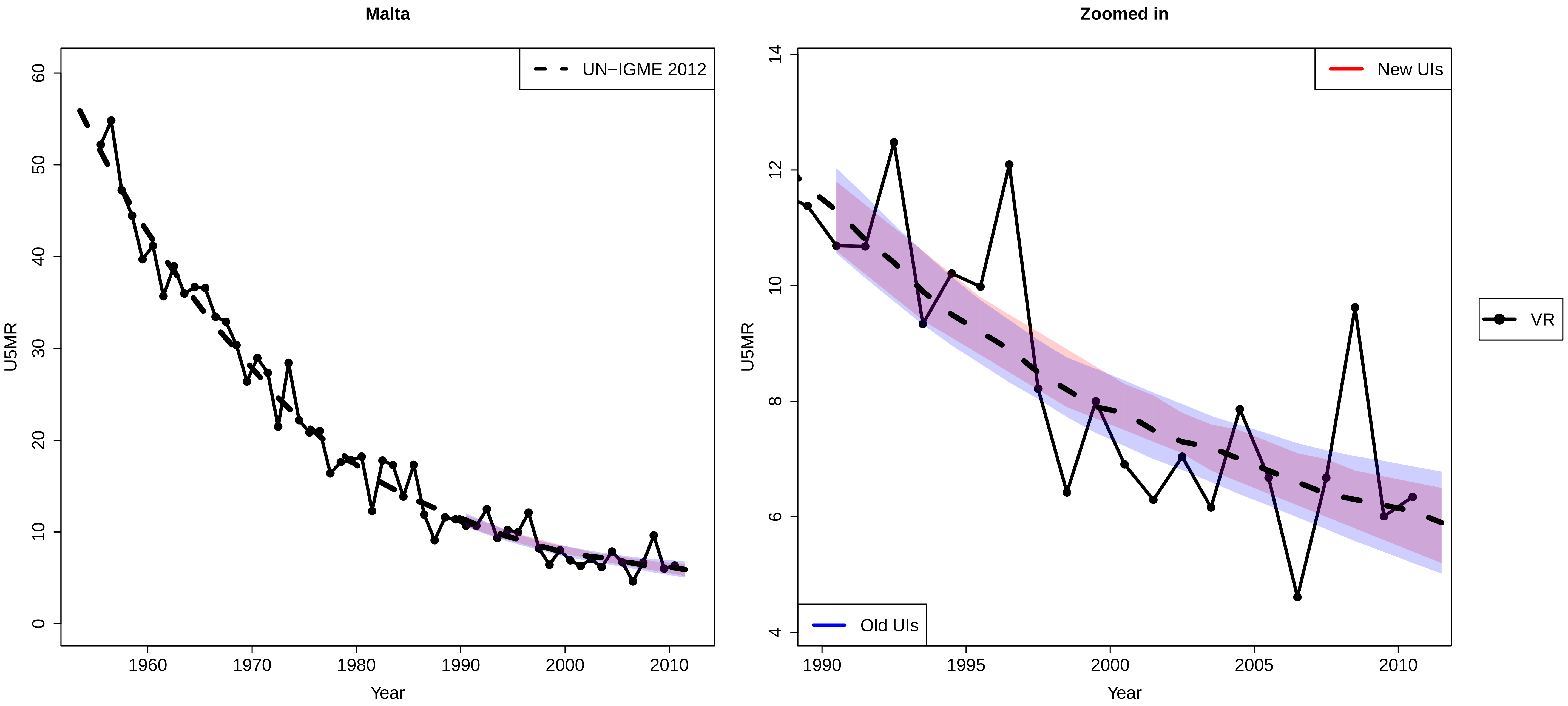

Malta
UN−IGME 2012
U5MR
Year
Zoomed in
New UIs
Old UIs
VR

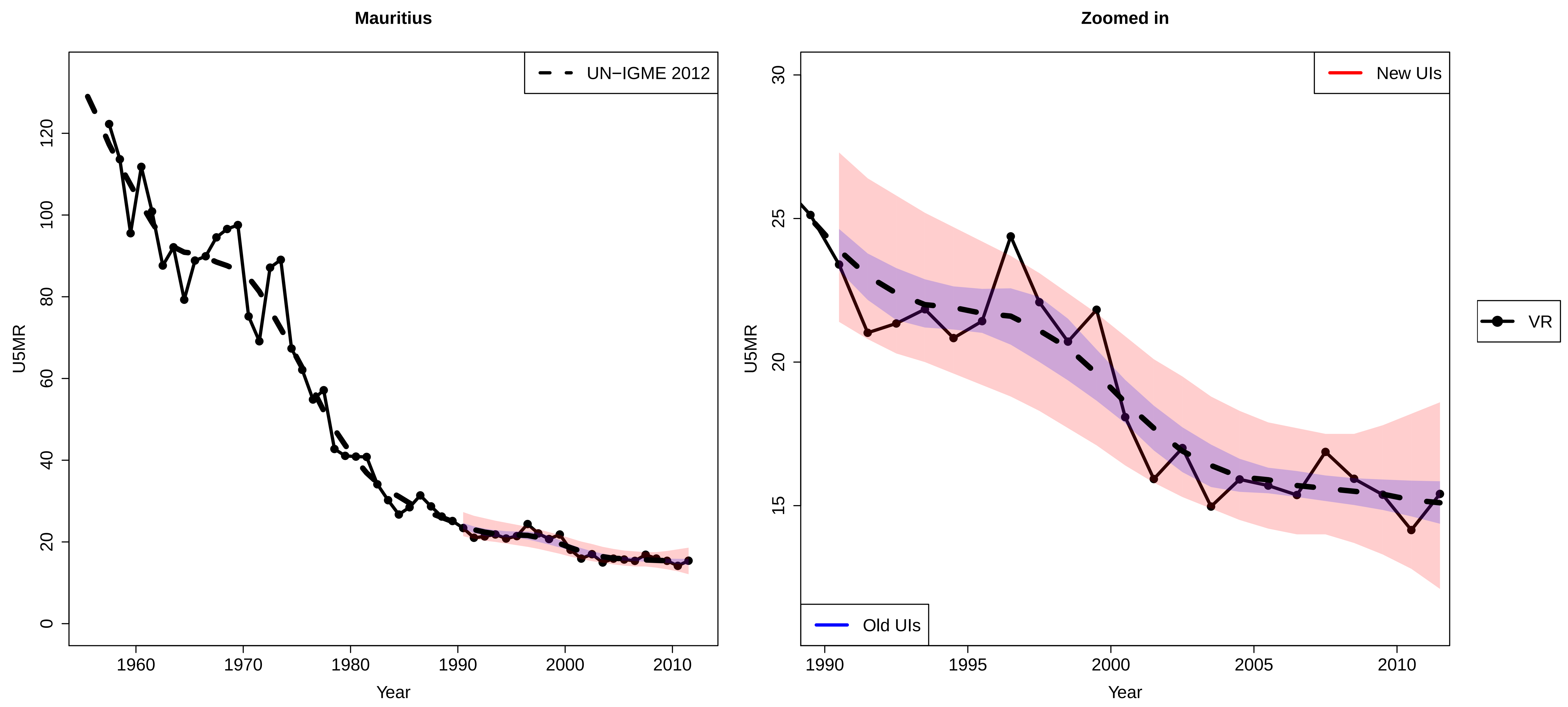

Mauritius
UN−IGME 2012
U5MR
Year
Zoomed in
New UIs
Old UIs
VR

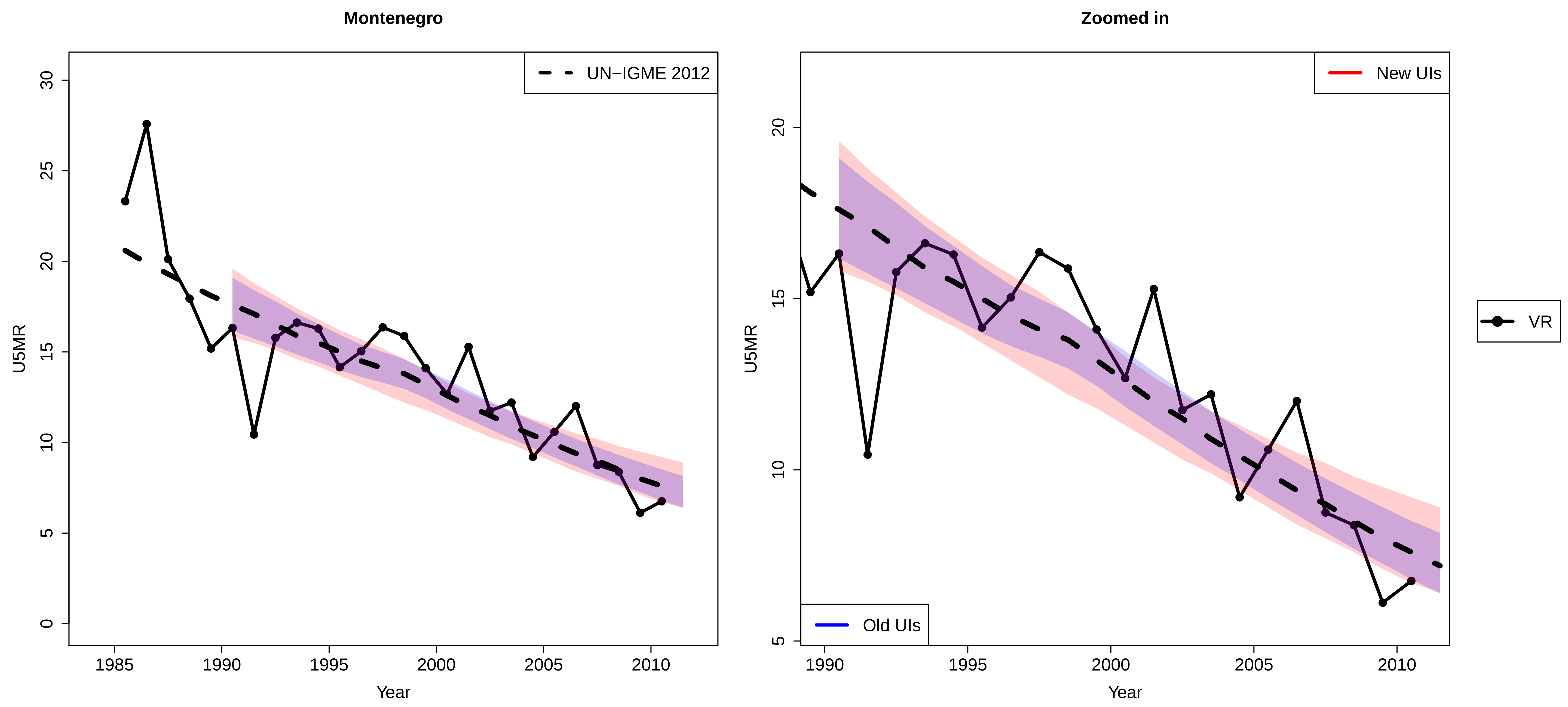
Montenegro
UN−IGME 2012
U5MR
Year
Zoomed in
New UIs
Old UIs
VR

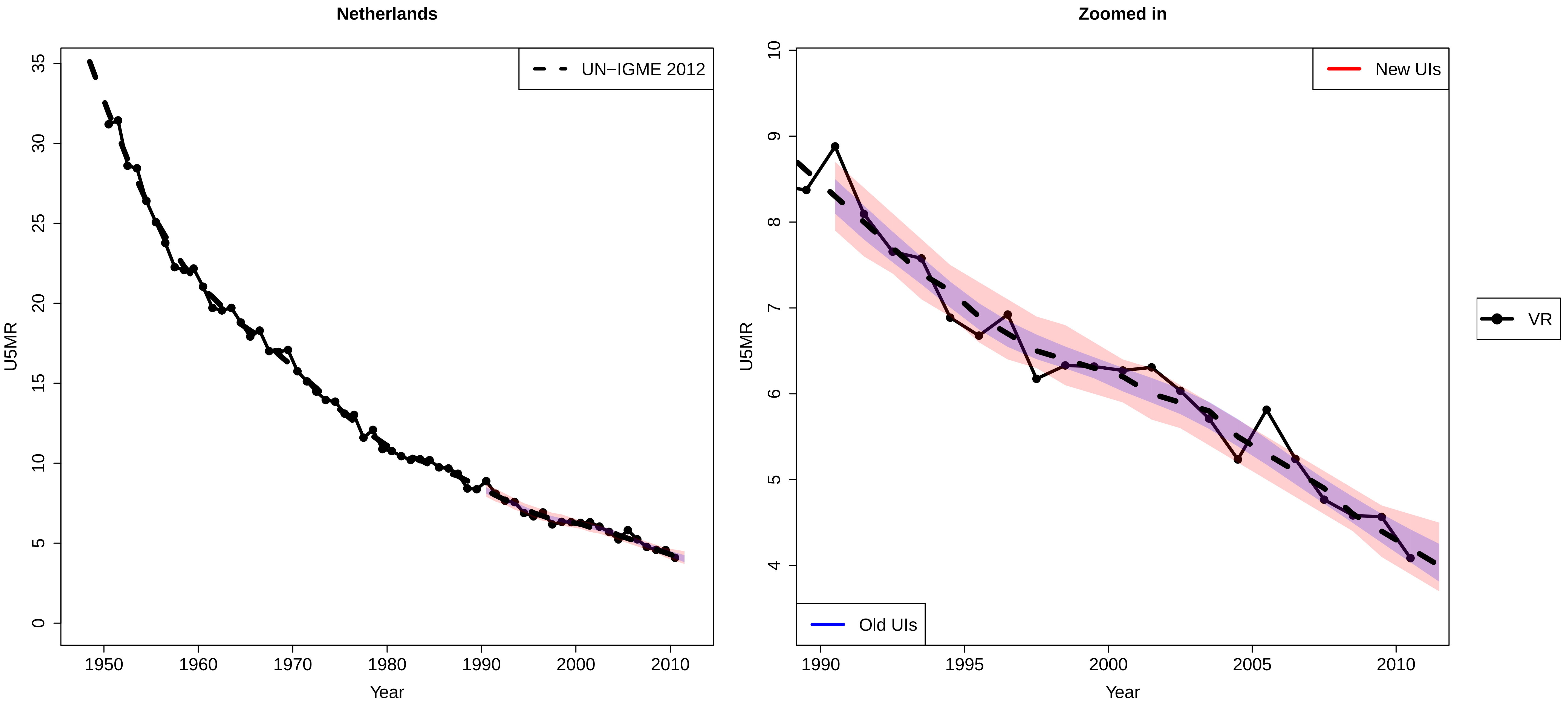
Netherlands
UN−IGME 2012
U5MR
Year
Zoomed in
New UIs
Old UIs
VR
U5MR
Year

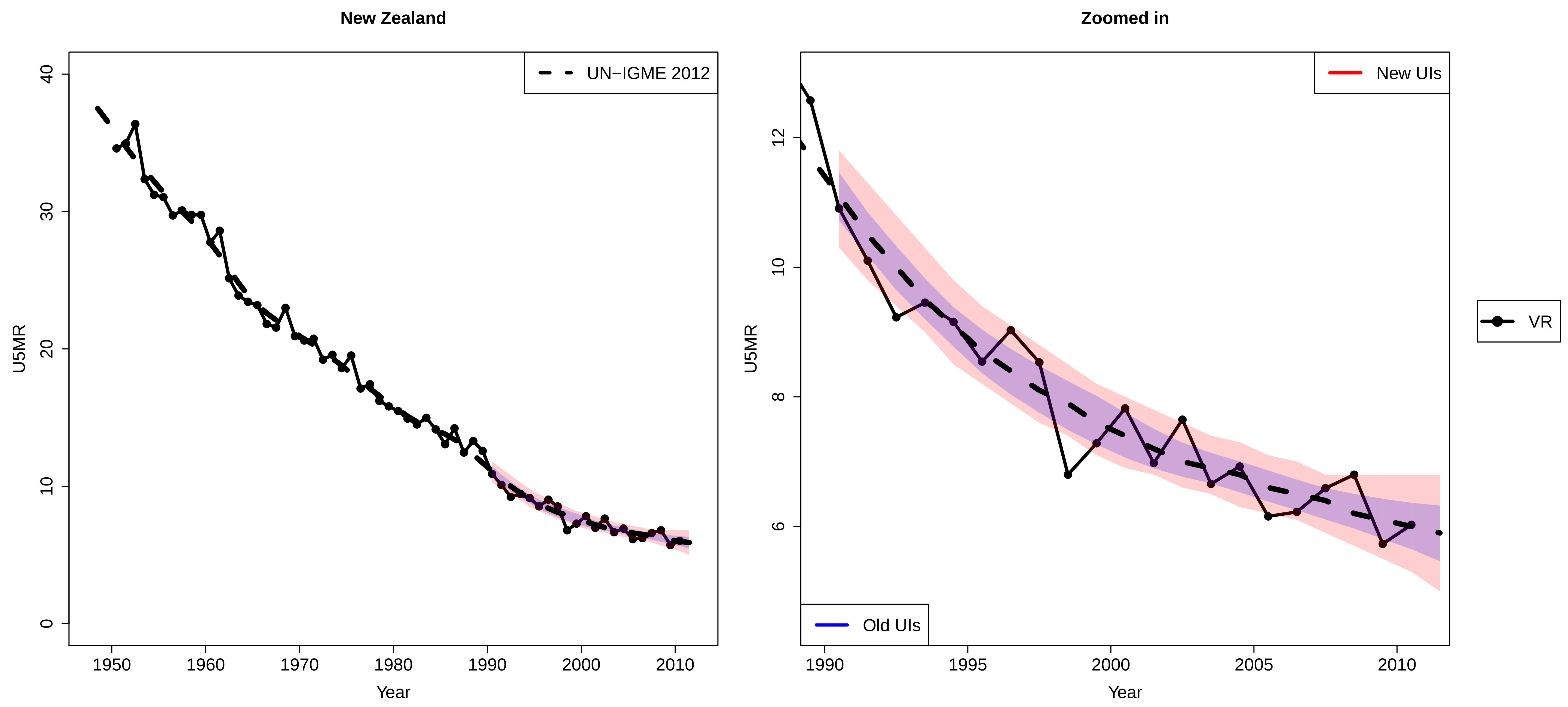

New Zealand
Zoomed in
UN−IGME 2012
New UIs
Old UIs
VR
U5MR
Year

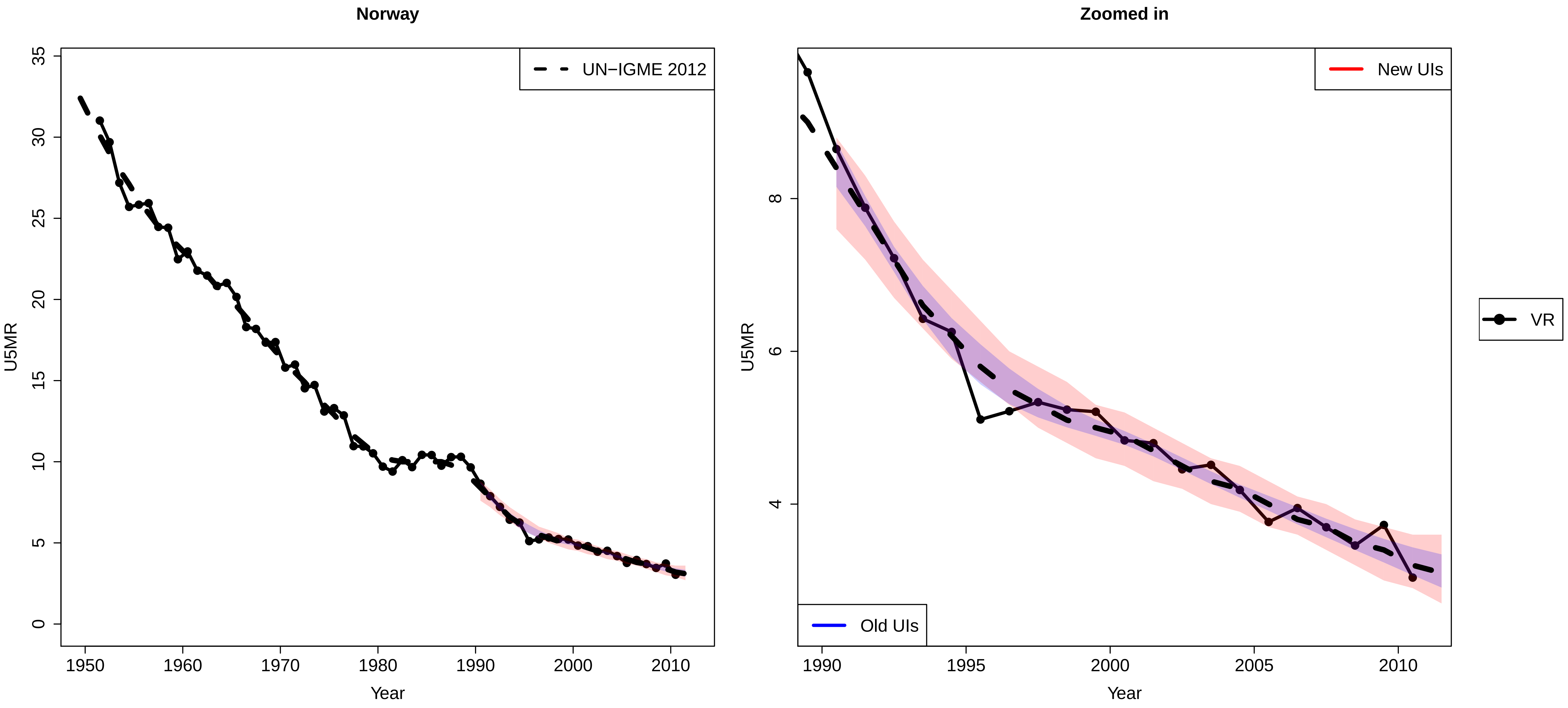

Norway
UN−IGME 2012
U5MR
Year
Zoomed in
New UIs
Old UIs
VR

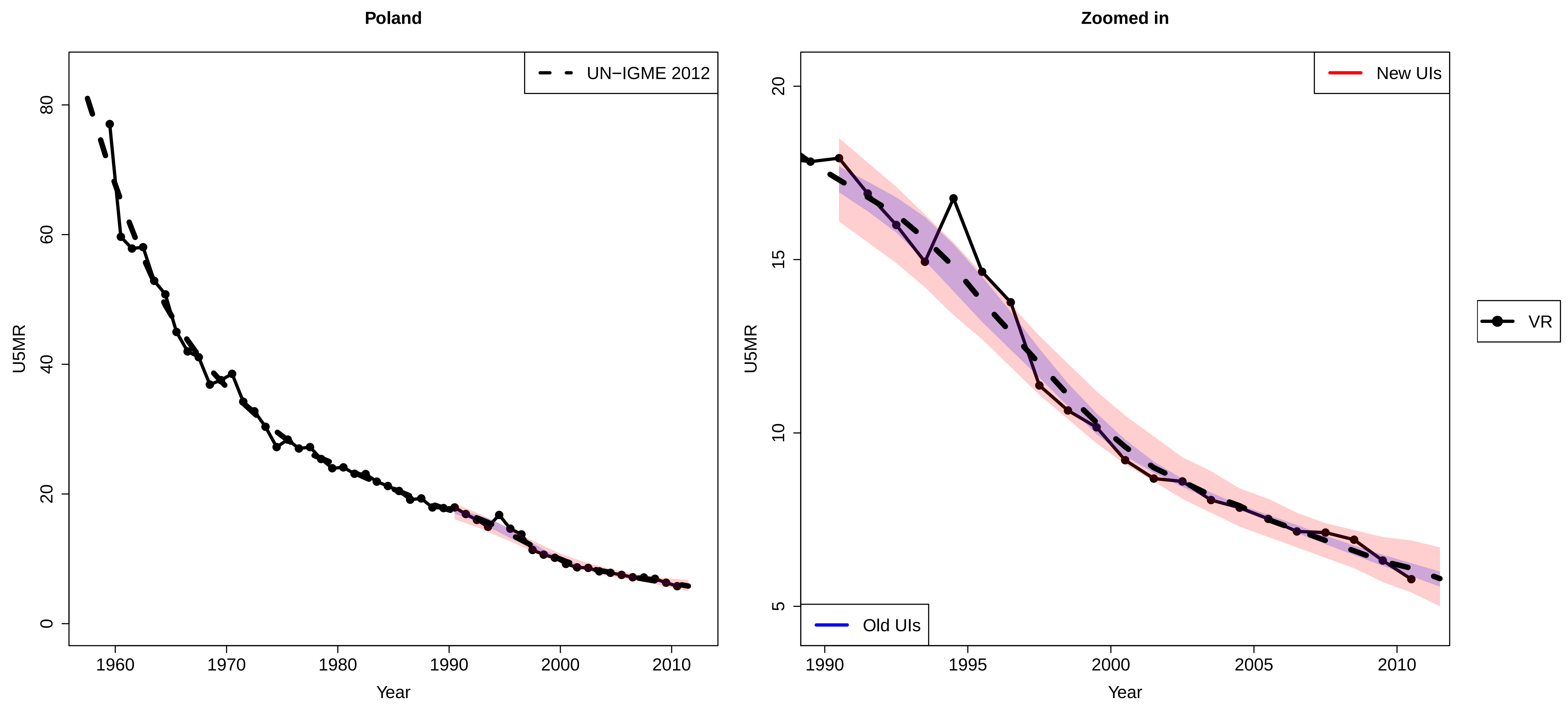

Poland
Zoomed in
UN−IGME 2012
New UIs
Old UIs
VR
U5MR
Year

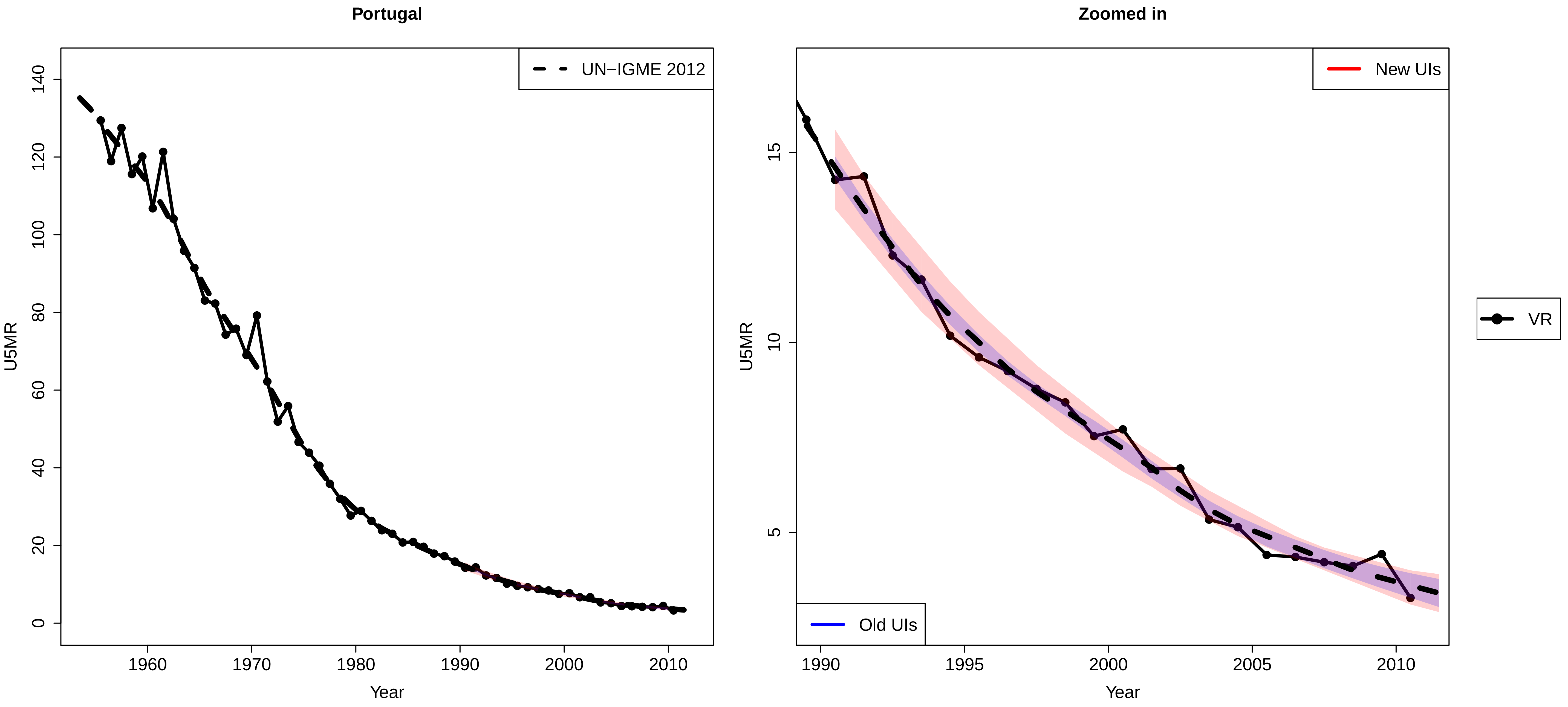
Portugal
Zoomed in
UN−IGME 2012
New UIs
Old UIs
VR
U5MR
Year
0
20
40
60
80
100
120
140
1960
1970
1980
1990
2000
2010
5
10
15
1990
1995
2000
2005
2010

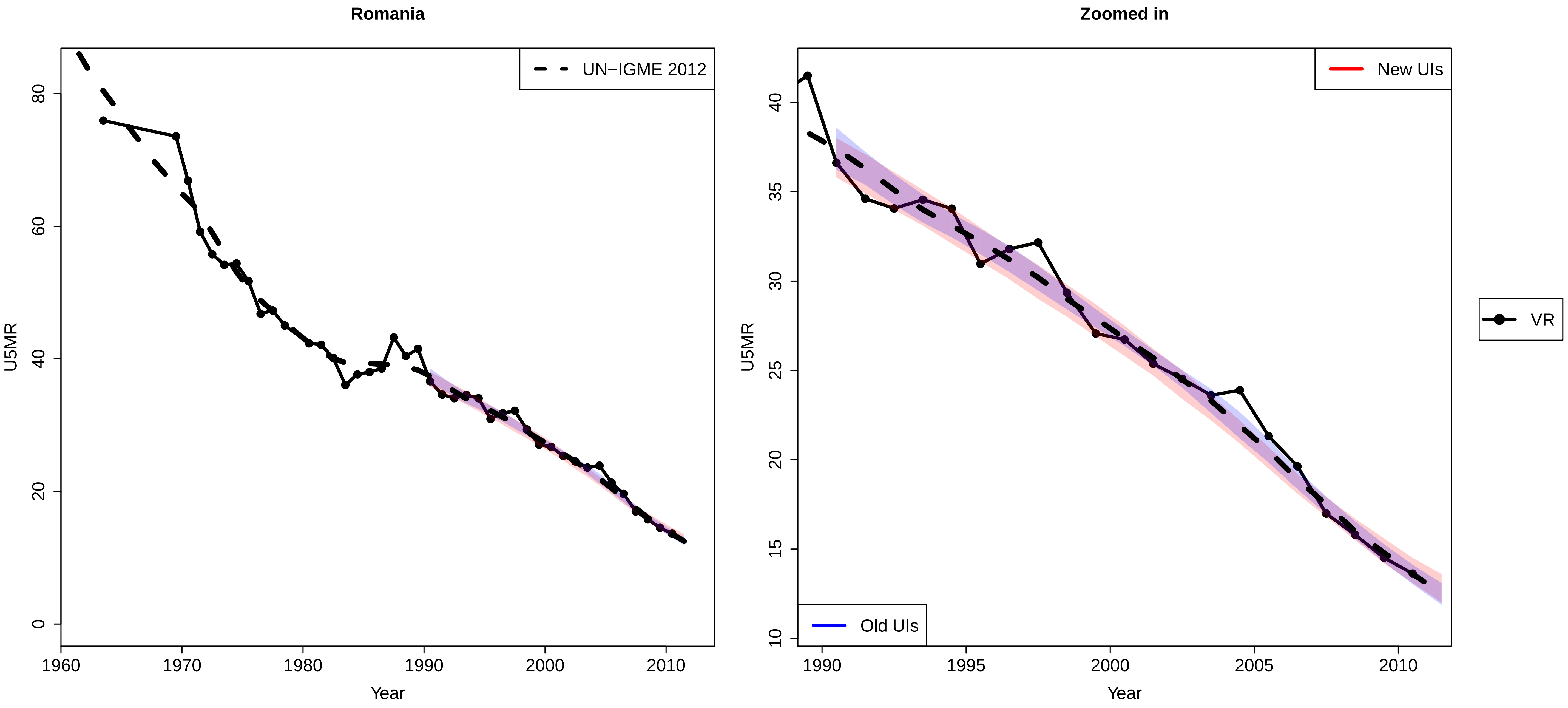
Romania
UN−IGME 2012
U5MR
Year
Zoomed in
New UIs
Old UIs
VR

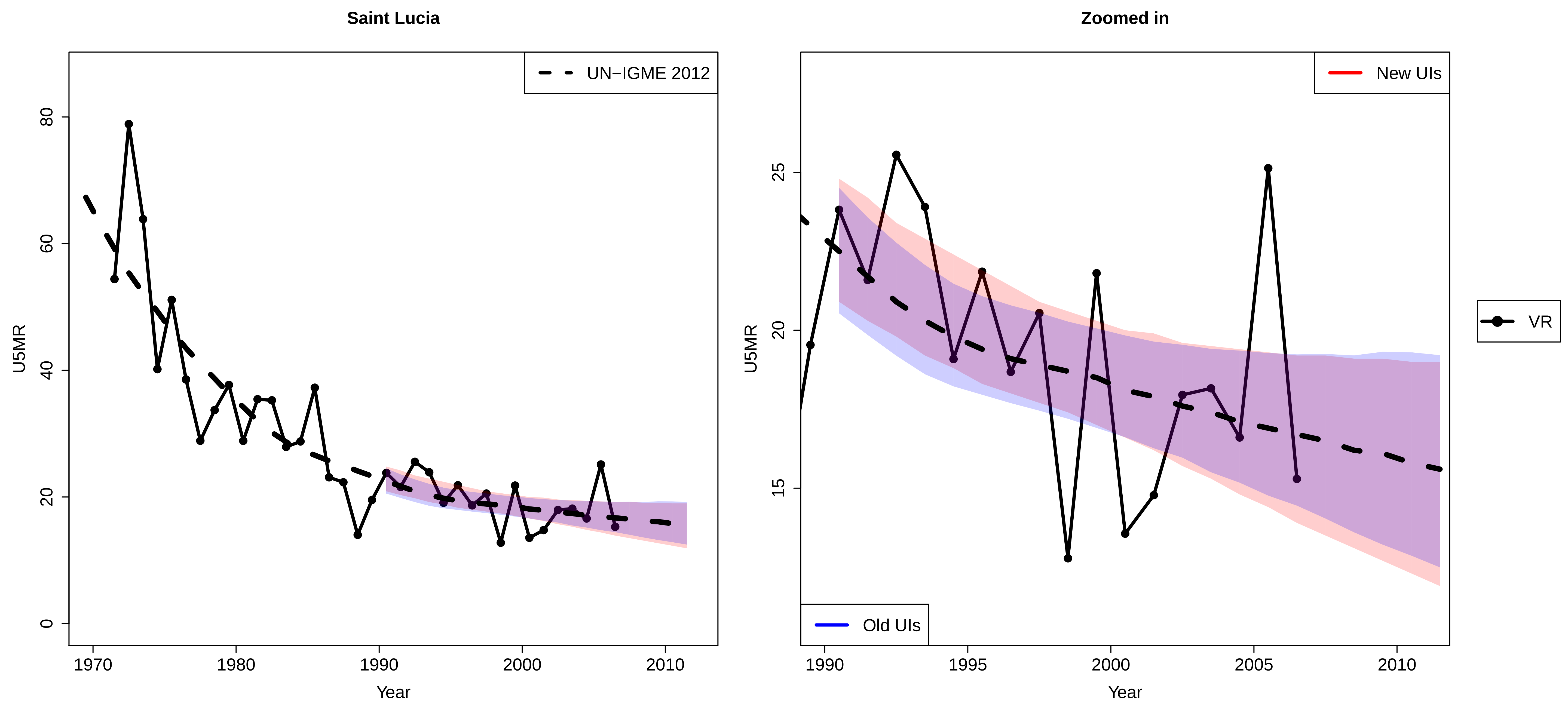

Saint Lucia
UN−IGME 2012
U5MR
0
20
40
60
80
1970
1980
1990
2000
2010
Year
Zoomed in
New UIs
Old UIs
15
20
25
1990
1995
2000
2005
2010
VR

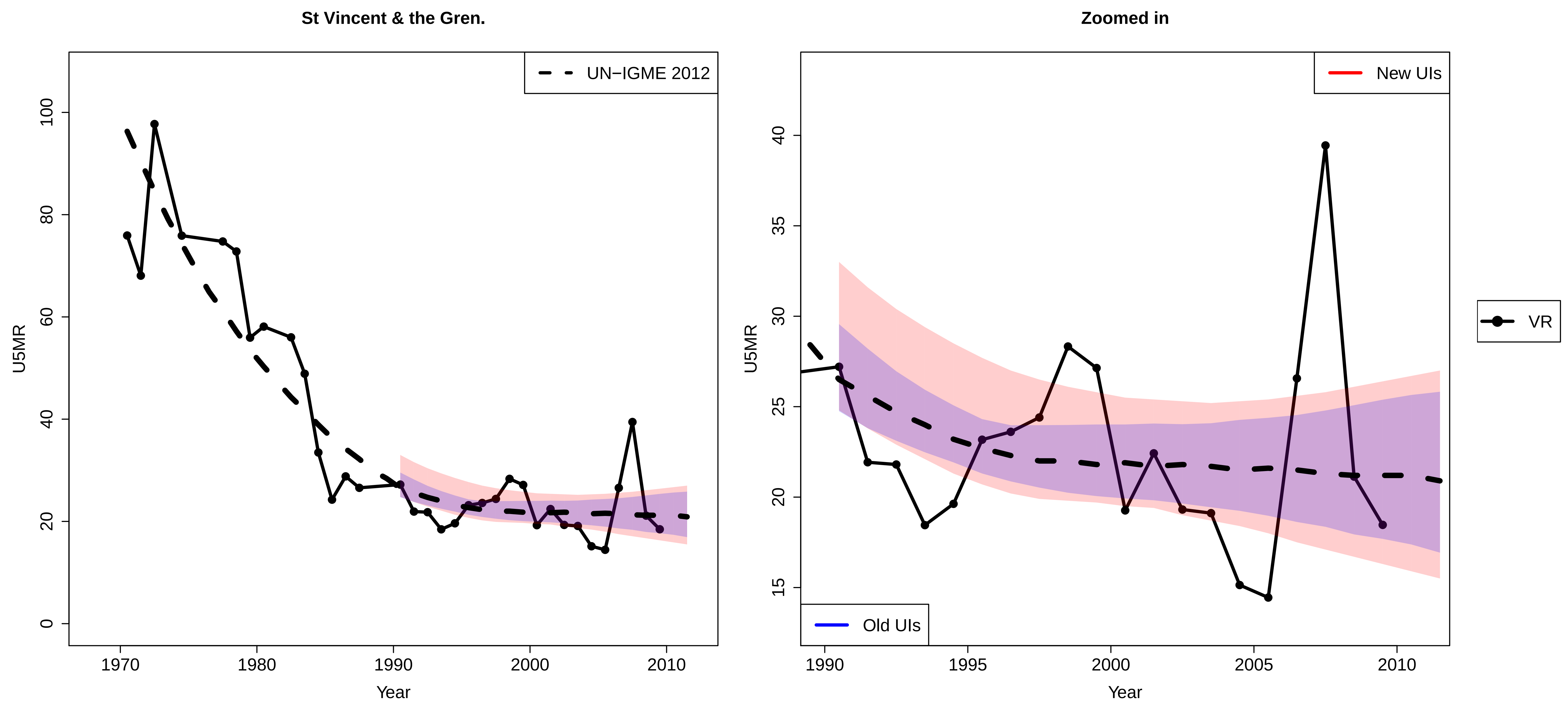
St Vincent & the Gren.
UN−IGME 2012
U5MR
Year
Zoomed in
New UIs
Old UIs
VR

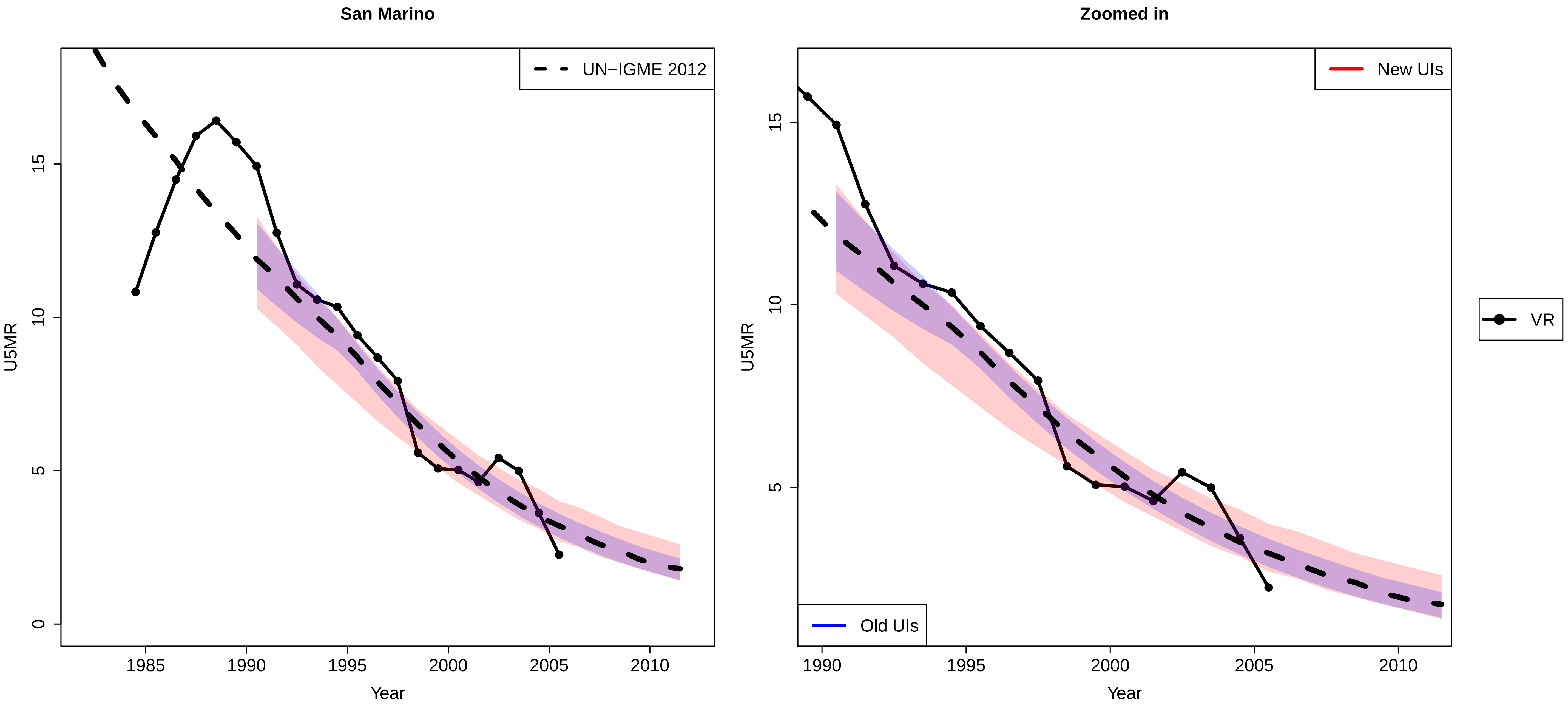
San Marino
Zoomed in
UN−IGME 2012
New UIs
Old UIs
VR
U5MR
Year
0
5
10
15
1985
1990
1995
2000
2005
2010

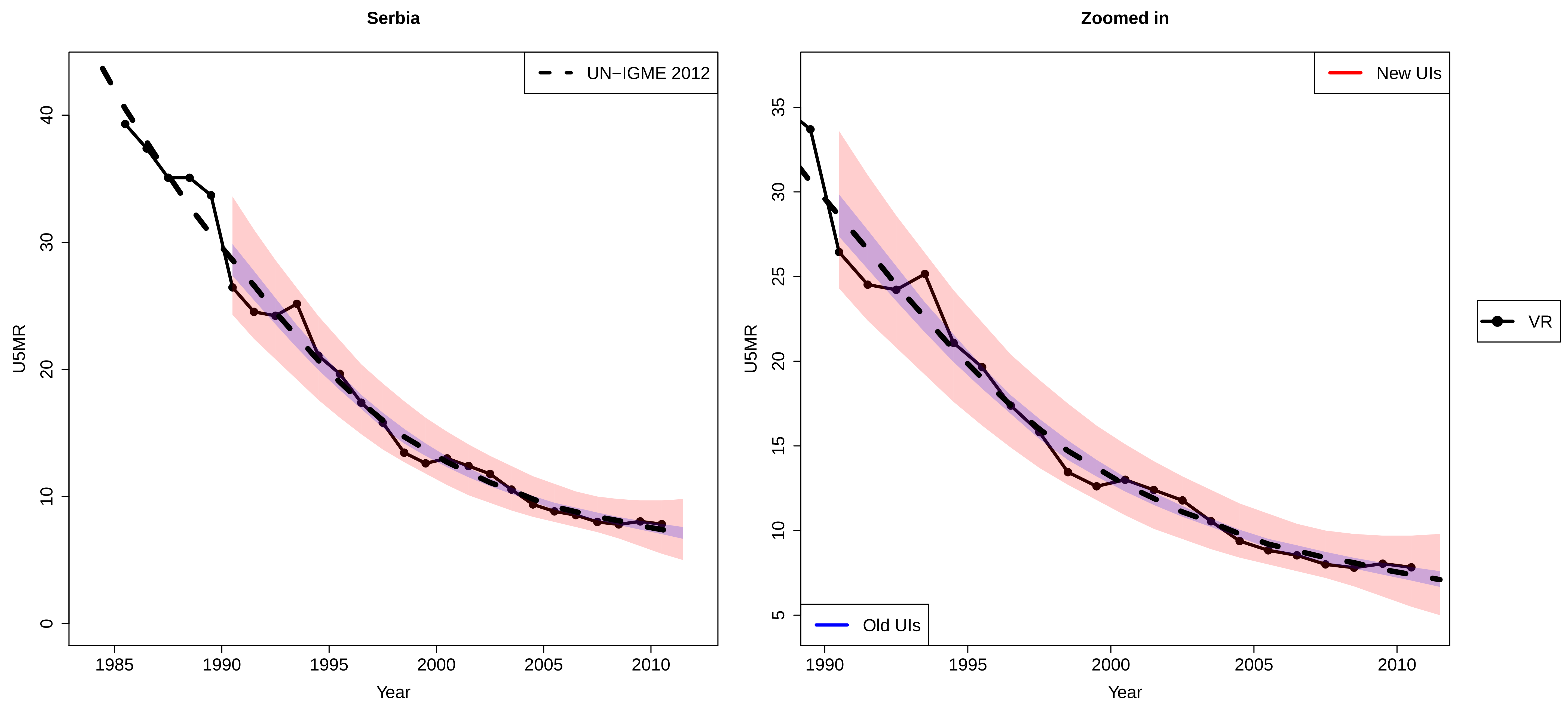

Serbia
UN−IGME 2012
U5MR
0
10
20
30
40
1985
1990
1995
2000
2005
2010
Year
Zoomed in
New UIs
Old UIs
5
10
15
20
25
30
35
VR

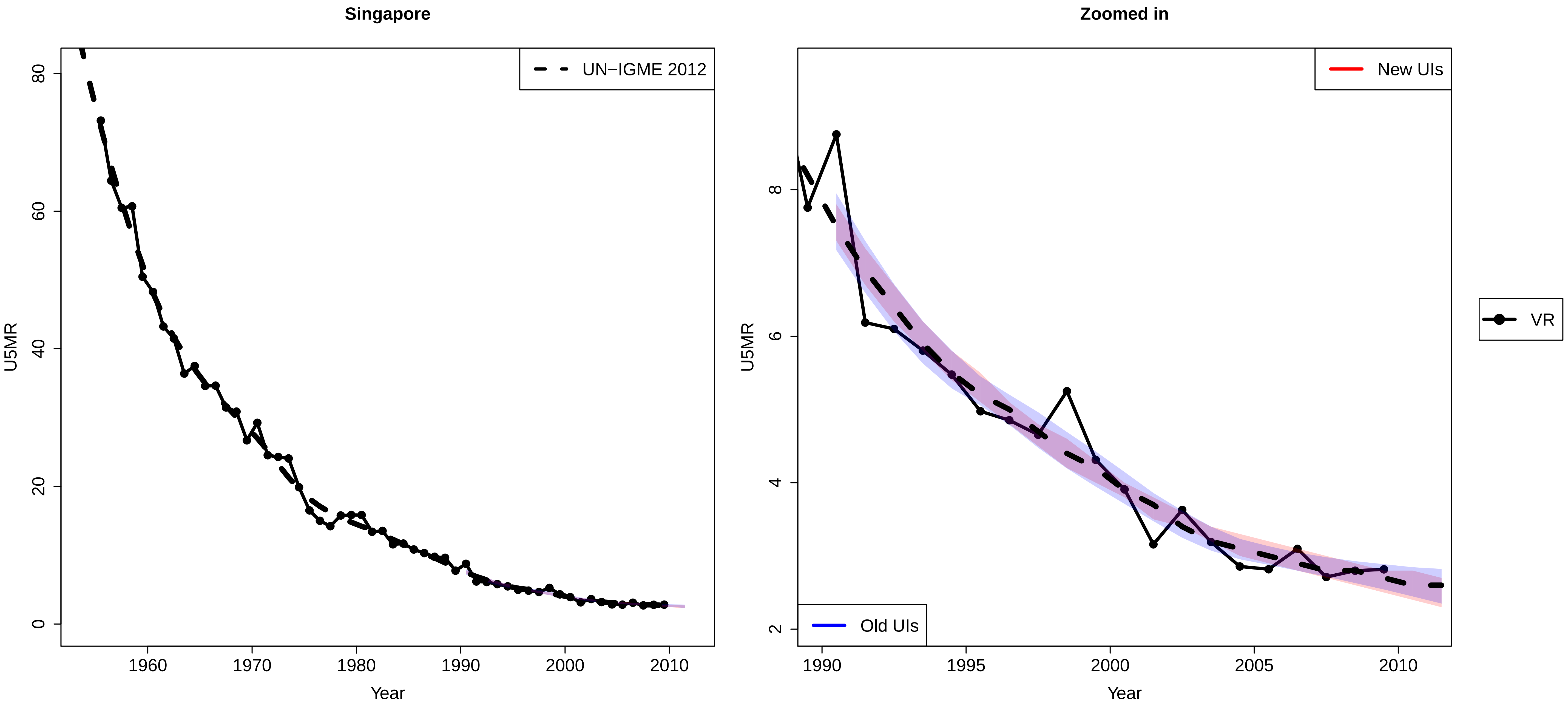

Singapore
Zoomed in
UN−IGME 2012
New UIs
Old UIs
VR
U5MR
Year

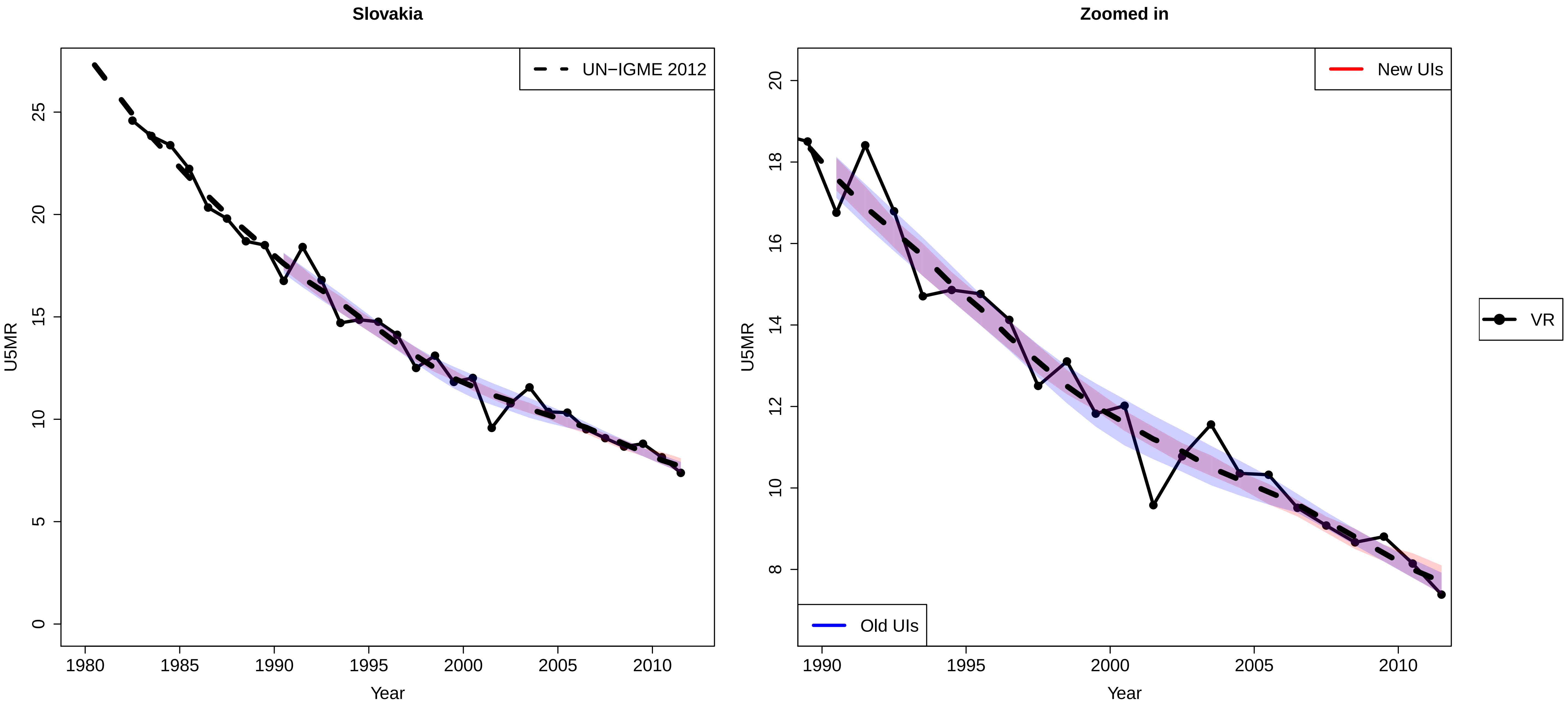

Slovakia
UN−IGME 2012
U5MR
Year
Zoomed in
New UIs
Old UIs
VR

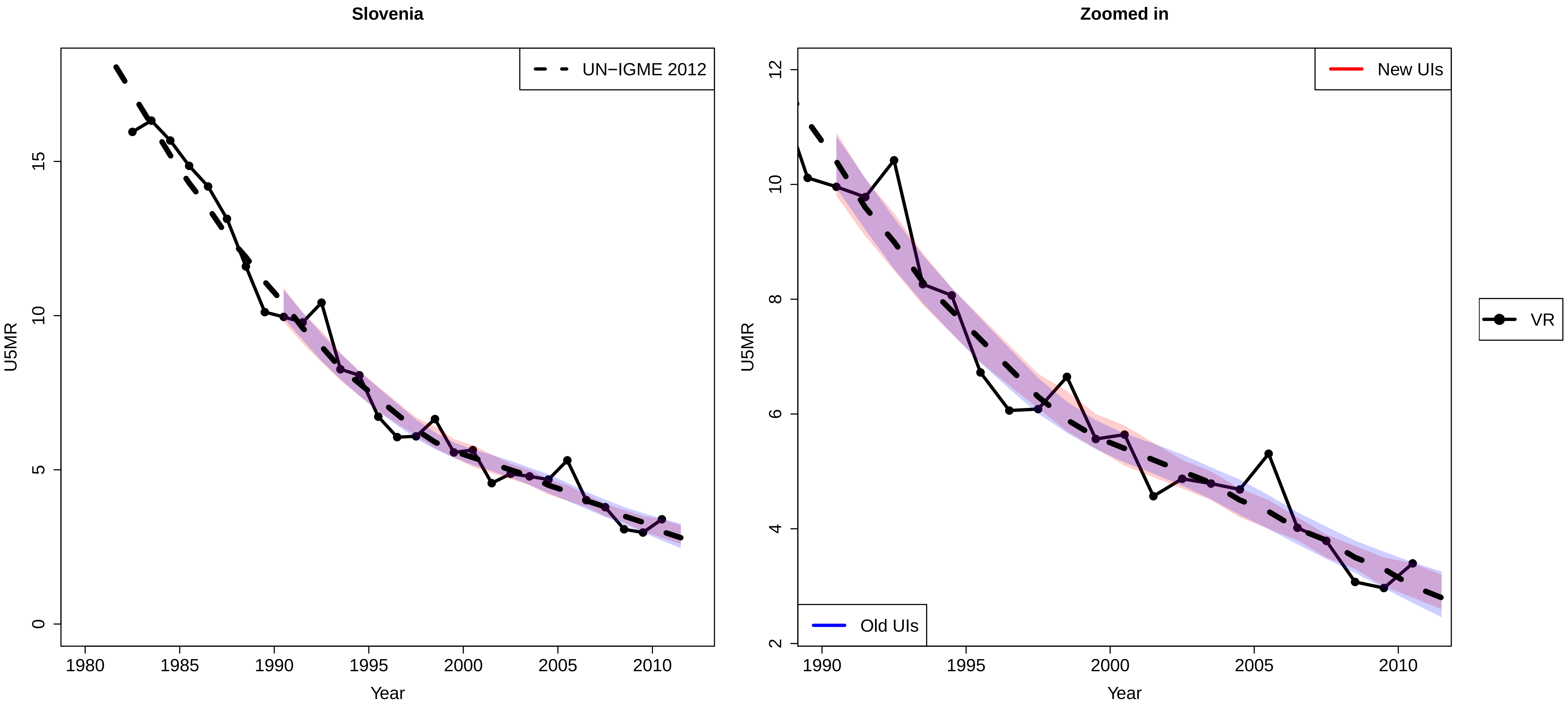

Slovenia
UN−IGME 2012
U5MR
Year
Zoomed in
New UIs
Old UIs
VR

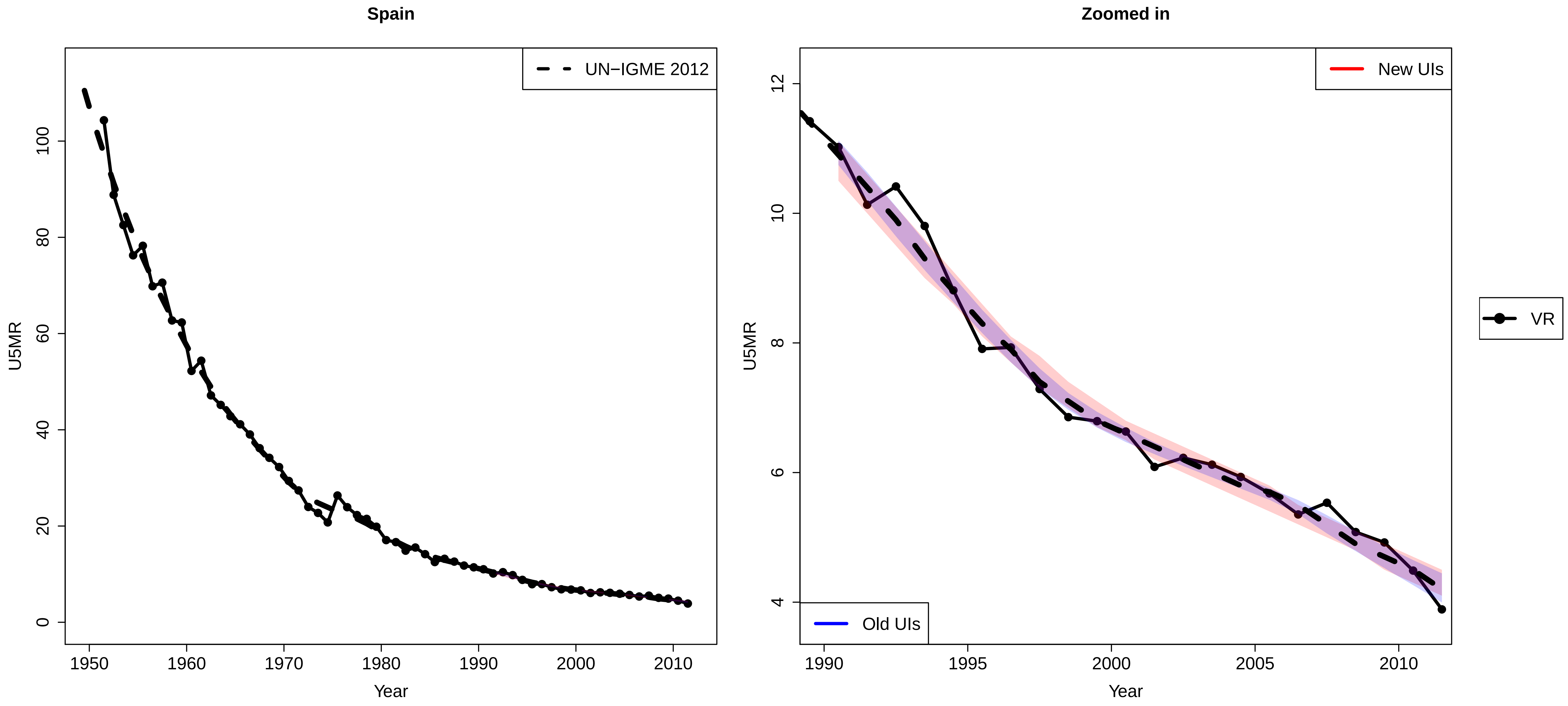

Spain
UN−IGME 2012
U5MR
Year
Zoomed in
New UIs
Old UIs
VR

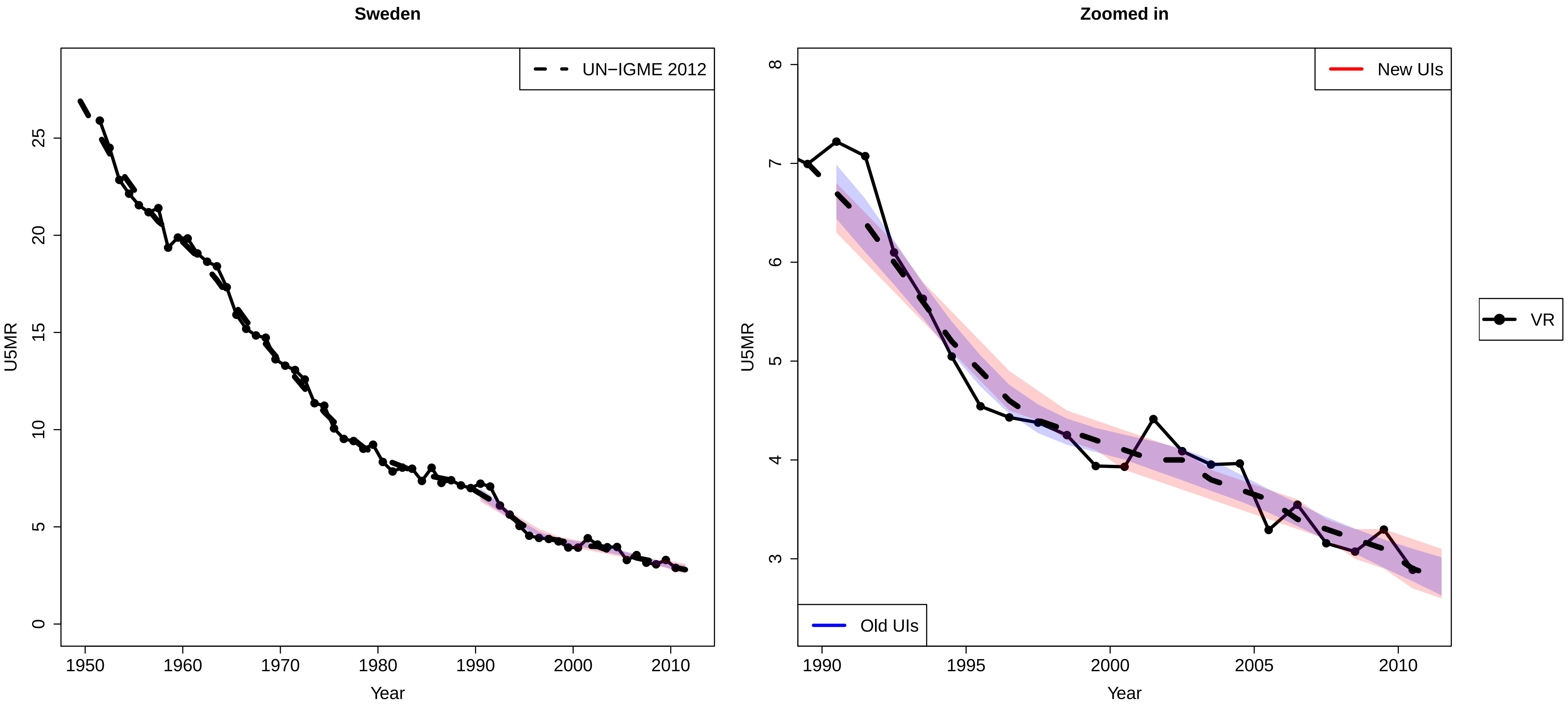

Sweden
UN−IGME 2012
U5MR
Year
Zoomed in
New UIs
Old UIs
VR

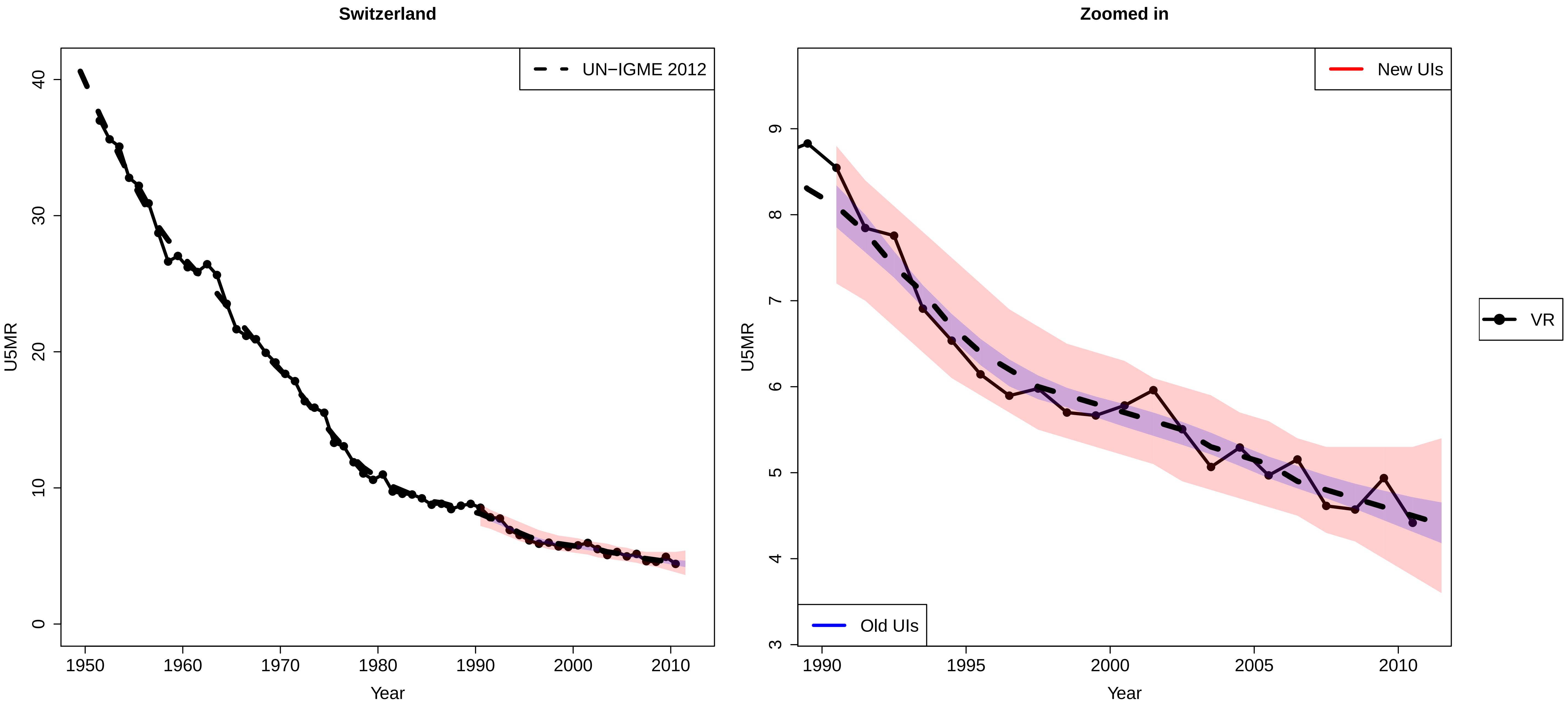

Switzerland
UN−IGME 2012
U5MR
Year
0
10
20
30
40
1950
1960
1970
1980
1990
2000
2010
Zoomed in
New UIs
Old UIs
VR
U5MR
Year
3
4
5
6
7
8
9
1990
1995
2000
2005
2010

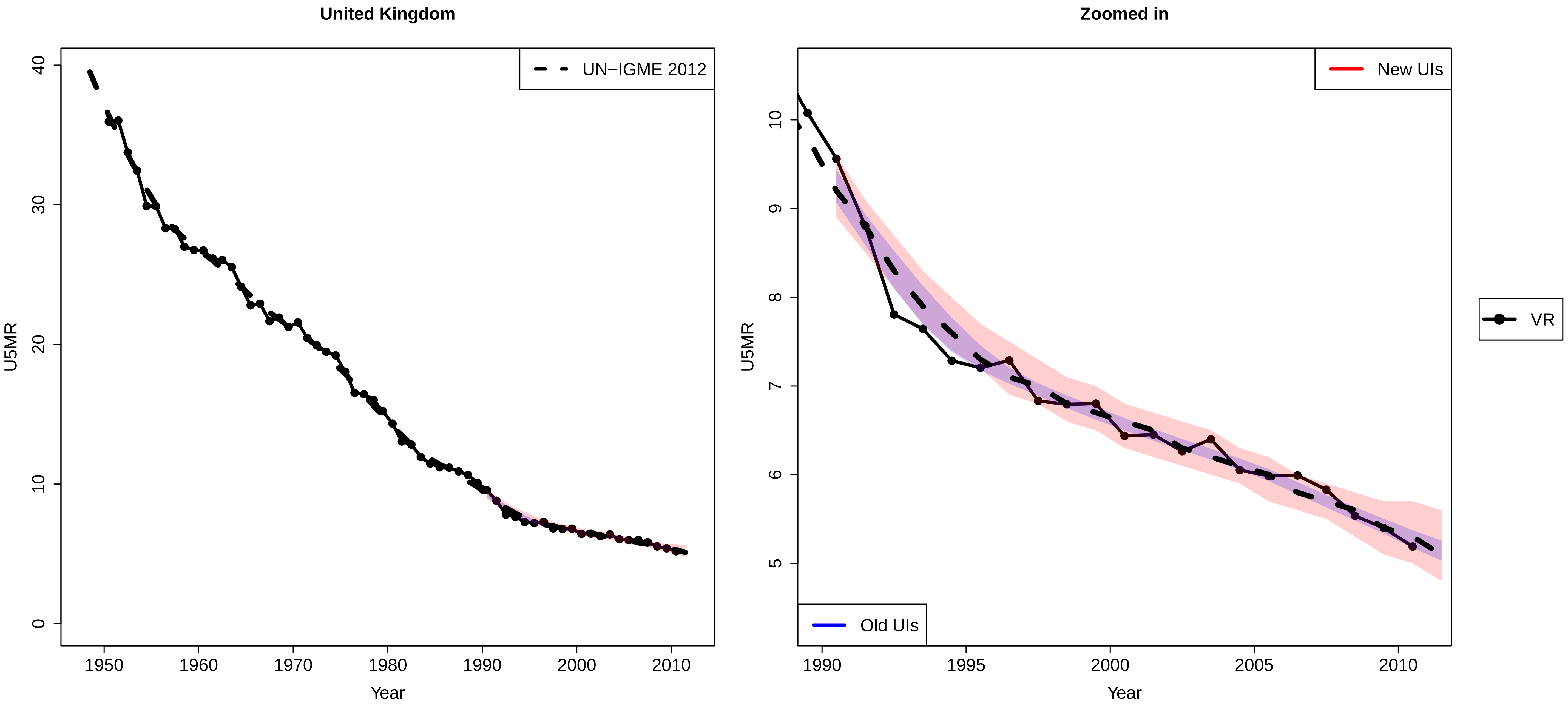
United Kingdom
UN−IGME 2012
U5MR
0
10
20
30
40
1950
1960
1970
1980
1990
2000
2010
Year
Zoomed in
New UIs
Old UIs
VR
5
6
7
8
9
10
1995
2005

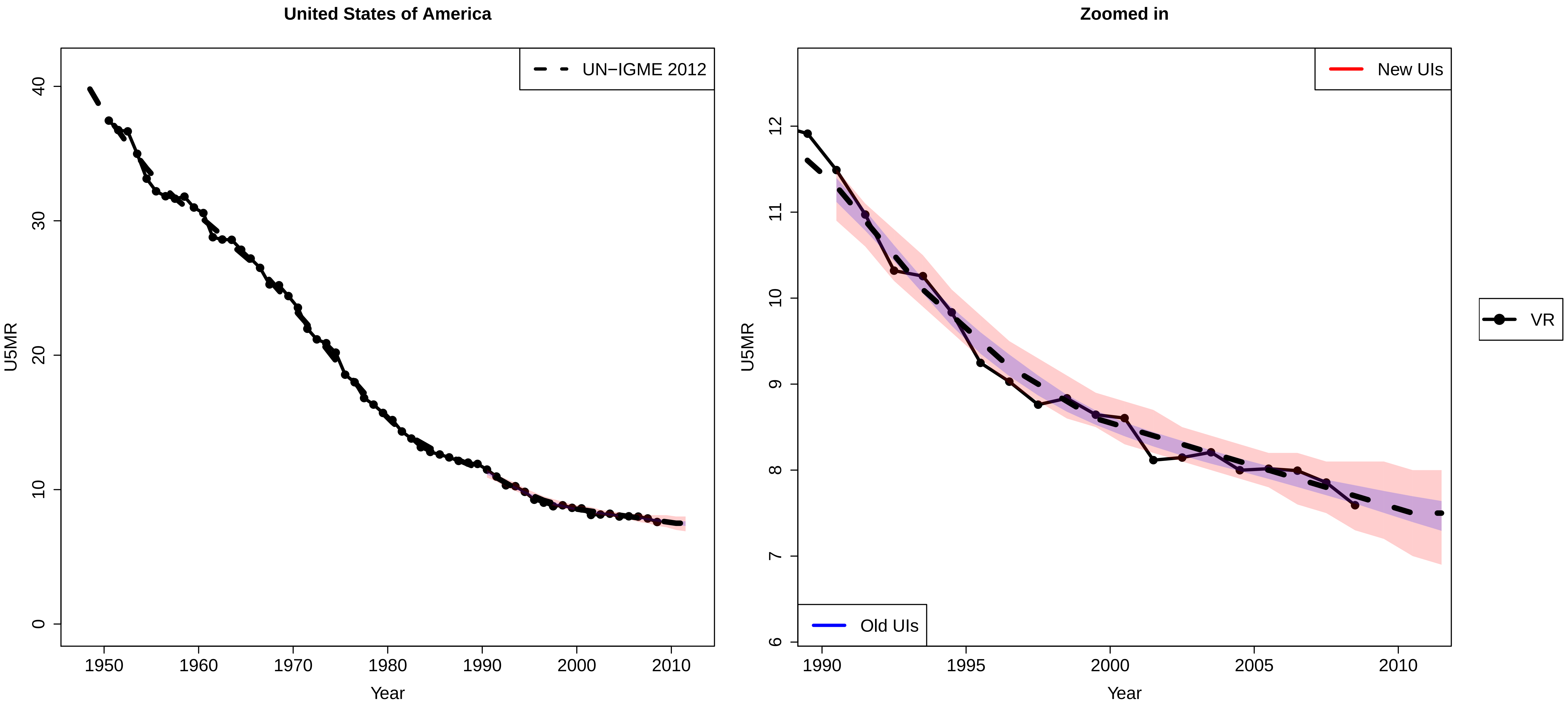

United States of America
UN−IGME 2012
U5MR
Year
0
10
20
30
40
1950
1960
1970
1980
1990
2000
2010
Zoomed in
New UIs
Old UIs
VR
6
7
8
9
10
11
12
1990
1995
2000
2005
2010
