# Supplementary material for: Progress toward Global Reduction in Under-Five Mortality: A Bootstrap Analysis of Uncertainty in Millennium Development Goal 4 Estimates
Source: PLoS Med. 2012 Dec 11;9(12):e1001355. doi: 10.1371/journal.pmed.1001355 (PMC3519895; doi:10.1371/journal.pmed.1001355)
Supplement: Table S1 — Estimates and uncertainty intervals for all countries for the U5MR in 1990, 2000, and 2011, and the ARR from 1990 to 2011. (DOCX) [file pmed.1001355.s002.docx]

| **Country** | **ISO-Code** | **U5MR 1990** | **U5MR 2000** | **U5MR 2011** | **ARR**  **1990-2011** |
| --- | --- | --- | --- | --- | --- |
| Afghanistan | AFG | 192 (159.7,210) | 136.2 (125,150.8) | 101.1 (83.8,125.9) | 3.1 (1.6,3.9) |
| Albania | ALB | 41.2 (36.1,48.1) | 26.3 (23,31) | 14.3 (10.7,22) | 5 (2.8,6.7) |
| Algeria | DZA | 65.6 (59.7,73) | 45.7 (40.9,53.7) | 29.8 (22.4,46.5) | 3.8 (1.5,5.3) |
| Andorra | AND | 8.3 (7.8,8.6) | 5 (5,5.2) | 3.3 (3.1,3.5) | 4.4 (4,4.7) |
| Angola | AGO | 243.2 (218.7,285) | 199.3 (180,241) | 157.6 (123.6,231) | 2.1 (0.1,3.6) |
| Antigua & Barbuda | ATG | 26.8 (20.6,34) | 14.8 (13.5,16.2) | 7.6 (6.1,9.6) | 6 (4,7.9) |
| Argentina | ARG | 27.6 (25.8,29.7) | 20.3 (18.9,21.6) | 14.1 (12.1,16.2) | 3.2 (2.5,4) |
| Armenia | ARM | 47.2 (42.4,51.2) | 29.8 (25.7,34.4) | 17.5 (13.3,24.2) | 4.7 (3.1,6) |
| Australia | AUS | 9.1 (7.9,10.4) | 6.2 (5.4,7.2) | 4.5 (3.5,5.9) | 3.4 (1.9,4.7) |
| Austria | AUT | 9.4 (8.6,10.3) | 5.6 (5.1,6.1) | 4.2 (3.6,4.9) | 3.8 (3,4.7) |
| Azerbaijan | AZE | 94.5 (84,104.5) | 68.6 (61.6,80.3) | 44.7 (38.4,64.7) | 3.6 (1.7,4.3) |
| Bahamas | BHS | 21.9 (19.5,24.2) | 16.9 (15.5,19.3) | 16.2 (11.7,19.4) | 1.4 (0.3,3.1) |
| Bahrain | BHR | 20.5 (18,21.7) | 12.4 (11.6,14.3) | 10 (7.7,12) | 3.4 (2.3,4.6) |
| Bangladesh | BGD | 138.8 (130.2,143.3) | 84.4 (79.5,88.6) | 46 (41.3,51.4) | 5.3 (4.6,5.7) |
| Barbados | BRB | 17.9 (17.3,20.6) | 17.2 (16.1,19.1) | 19.7 (15.3,22.4) | -0.5 (-1,1.2) |
| Belarus | BLR | 17.2 (16.3,18.7) | 13.7 (12.6,14.4) | 5.6 (4.8,6.8) | 5.3 (4.4,6.2) |
| Belgium | BEL | 10 (9.2,10.6) | 5.8 (5.5,6.3) | 4.3 (3.7,4.9) | 4 (3.2,4.8) |
| Belize | BLZ | 43.9 (40,49.9) | 26.3 (25,29.4) | 16.9 (14,19.1) | 4.5 (3.8,5.8) |
| Benin | BEN | 177.3 (164.5,185.3) | 139.7 (128.1,150.9) | 106 (93.8,123.9) | 2.4 (1.7,2.9) |
| Bhutan | BTN | 138.4 (122.4,152) | 89.1 (80.4,103) | 53.7 (46.5,73) | 4.5 (2.9,5.2) |
| Bolivia | BOL | 119.5 (112.6,125.3) | 80.8 (73.5,85.7) | 50.6 (42.9,58.2) | 4.1 (3.4,4.9) |
| Bosnia & Herzegovina | BIH | 18.8 (17.4,20.1) | 9.6 (9,10.7) | 7.7 (6.8,9.1) | 4.3 (3.4,4.9) |
| Brazil | BRA | 58 (54.2,63.7) | 35.7 (33.6,39.7) | 15.6 (13.7,18.3) | 6.3 (5.5,7) |
| Brunei Darussalam | BRN | 12.3 (11.3,13.7) | 9.6 (8.7,10.5) | 7.2 (6.1,8.8) | 2.6 (1.5,3.6) |
| Bulgaria | BGR | 22.2 (20.9,24) | 20.7 (19,21.8) | 12.1 (10.4,14) | 2.9 (2.2,3.7) |
| Burkina Faso | BFA | 208.4 (194.4,220.2) | 181.5 (168.4,198.5) | 146.4 (133.3,183) | 1.7 (0.5,2.2) |
| Burundi | BDI | 182.6 (168.8,204.5) | 164.6 (147.2,196.2) | 139.1 (116.4,198.8) | 1.3 (-0.3,2.3) |
| Cambodia | KHM | 116.7 (109.5,125.5) | 101.5 (91.5,108) | 42.5 (35.8,60.7) | 4.8 (3.1,5.7) |
| Canada | CAN | 8.3 (7.7,8.9) | 6.2 (5.9,6.7) | 5.6 (4.8,6.7) | 1.9 (0.9,2.7) |
| Cape Verde | CPV | 58 (49.7,66.3) | 38.9 (33.5,41.5) | 21.3 (18.1,25.2) | 4.8 (3.6,5.8) |
| Chad | TCD | 208.3 (193.4,224.9) | 188.5 (173,211.1) | 169 (146.3,206.3) | 1 (0.1,1.7) |
| Chile | CHL | 18.7 (17.7,19) | 10.8 (10.5,11.3) | 8.7 (7.8,9.3) | 3.6 (3.2,4.1) |
| China | CHN | 48.9 (44.4,54.4) | 35 (31.7,36.7) | 14.6 (13.3,16.5) | 5.8 (5,6.4) |
| Colombia | COL | 34.3 (32,36.3) | 25 (22.7,26.8) | 17.7 (14.9,20.5) | 3.2 (2.4,4) |
| Comoros | COM | 121.7 (104.4,145.8) | 99.6 (78,123.1) | 79.3 (50.6,117.3) | 2 (0.2,4.3) |
| Congo | COG | 118.8 (111.4,129.2) | 108.8 (98.5,116.4) | 98.8 (84.1,107.3) | 0.9 (0.6,1.7) |
| Cook Islands | COK | 19.1 (16.4,24) | 17.1 (12.9,20.4) | 9.5 (5.8,17.1) | 3.3 (0.6,6.1) |
| Costa Rica | CRI | 17.2 (16.2,18.7) | 12.9 (12,13.7) | 10.1 (8.5,11.4) | 2.5 (1.9,3.5) |
| Croatia | HRV | 12.9 (12.2,14) | 8.4 (7.8,8.9) | 5.1 (4.5,5.8) | 4.4 (3.8,5.2) |
| Cuba | CUB | 13.3 (12.6,14.5) | 8.5 (7.9,9.1) | 5.8 (4.9,6.6) | 4 (3.3,4.9) |
| Cyprus | CYP | 11 (10.2,11.8) | 6.5 (6.1,7.1) | 3.1 (2.8,3.7) | 6 (5.1,6.7) |
| Czech Republic | CZE | 14.3 (13.1,15) | 6.7 (6.4,7.3) | 3.9 (3.4,4.4) | 6.2 (5.4,6.8) |
| Denmark | DNK | 8.7 (8,9.1) | 5.6 (5.2,6) | 3.7 (3.3,4.4) | 4.1 (3.1,4.7) |
| Djibouti | DJI | 121.6 (110.7,138.2) | 105.7 (91,126.8) | 89.5 (68,123.4) | 1.5 (0.1,2.8) |
| Dominica | DMA | 17.4 (14.8,19.4) | 14.9 (13.2,17.1) | 11.8 (9.1,15.3) | 1.8 (0.3,3.2) |
| Dominican Republic | DOM | 58.3 (52.8,59.9) | 38.5 (33.3,41) | 24.7 (19.2,28.2) | 4.1 (3.3,5.1) |
| Ecuador | ECU | 52.4 (47.6,58.8) | 34.2 (30,39.5) | 22.8 (16.5,30) | 4 (2.5,5.7) |
| Egypt | EGY | 85.7 (80.7,87.9) | 44.4 (41.7,46) | 21.1 (19.2,23.1) | 6.7 (6.1,7.1) |
| El Salvador | SLV | 60.2 (53.4,65) | 33.9 (29.4,38.1) | 15.2 (12.5,22.1) | 6.6 (4.6,7.5) |
| Equatorial Guinea | GNQ | 189.6 (156.1,252.3) | 152.2 (117.3,211.1) | 118.1 (62.9,234.8) | 2.3 (-1.3,6) |
| Eritrea | ERI | 137.7 (124.9,145.2) | 98.2 (89,109.8) | 67.8 (59.9,82.3) | 3.4 (2.5,3.7) |
| Estonia | EST | 20.1 (18.5,21.2) | 10.8 (10.1,11.6) | 3.7 (3.3,4.2) | 8.1 (7.3,8.6) |
| Ethiopia | ETH | 198.3 (178.9,209.4) | 138.6 (124.3,144.6) | 77 (65.1,92.5) | 4.5 (3.4,5.2) |
| Federated States of Micronesia | FSM | 56.4 (50,66.1) | 48.6 (37.4,69) | 41.5 (25.4,77.4) | 1.5 (-1.2,3.7) |
| Fiji | FJI | 29.6 (27.1,32.7) | 22.3 (20,24.9) | 16.4 (14.1,18.6) | 2.8 (2.5,3.3) |
| Finland | FIN | 6.7 (6.1,7) | 4.3 (4,4.6) | 2.9 (2.6,3.3) | 4 (3.1,4.5) |
| France | FRA | 8.8 (8.1,9.3) | 5.4 (5,5.8) | 4.1 (3.5,4.7) | 3.6 (2.9,4.4) |
| Gambia | GMB | 164.6 (154,188.8) | 130.3 (118.2,157.6) | 100.6 (81.6,139) | 2.3 (1,3.5) |
| Georgia | GEO | 46.9 (38.7,58) | 32.6 (28.4,39.2) | 20.5 (15,32.8) | 3.9 (1.2,5.9) |
| Germany | DEU | 8.5 (7.9,9.1) | 5.4 (5.1,5.8) | 4 (3.5,4.6) | 3.6 (2.8,4.4) |
| Ghana | GHA | 120.9 (114,127.4) | 98.7 (90.6,106.6) | 77.6 (65.6,94.6) | 2.1 (1.1,2.9) |
| Greece | GRC | 12.7 (12,13.8) | 7.7 (7.2,8.2) | 4.4 (3.7,5) | 5 (4.4,6) |
| Grenada | GRD | 21 (18.4,23.8) | 15.7 (14.9,17.4) | 12.8 (10.6,15) | 2.4 (1.3,3.5) |
| Guatemala | GTM | 78 (71.7,81.2) | 48.2 (41.5,53.2) | 30.4 (20.8,38) | 4.5 (3.3,6.2) |
| Guinea | GIN | 228.2 (211.7,241) | 174.5 (160.7,194.4) | 125.8 (112.4,159.8) | 2.8 (1.7,3.3) |
| Guinea-Bissau | GNB | 210.4 (183.4,237.6) | 185.8 (163.8,222) | 160.6 (131.9,215.5) | 1.3 (0.2,2.2) |
| Guyana | GUY | 63 (56.4,67.8) | 49.1 (45.1,56.9) | 35.9 (31,49.8) | 2.7 (1,3.4) |
| Haiti | HTI | 143 (129.6,148) | 102 (91,111.5) | 70 (59.1,84) | 3.4 (2.5,4) |
| Honduras | HND | 55 (50.1,60.2) | 35 (30.4,37.9) | 21.4 (16.1,25.1) | 4.5 (3.7,5.9) |
| Hungary | HUN | 18.7 (17.3,19.9) | 11 (10.2,11.7) | 6.3 (5.6,7.1) | 5.2 (4.5,5.8) |
| Iceland | ISL | 6.3 (5.9,6.6) | 3.9 (3.8,4.2) | 2.5 (2.2,2.7) | 4.4 (3.9,5.1) |
| India | IND | 114.2 (107.1,119.7) | 87.7 (82.6,91.1) | 61.3 (56.4,68.4) | 3 (2.3,3.4) |
| Indonesia | IDN | 81.6 (76.7,85) | 52.5 (50.5,56.6) | 31.8 (28.3,39.8) | 4.5 (3.3,5.1) |
| Iran | IRN | 61.1 (54.4,70.1) | 44 (37.2,49.6) | 25 (21,33) | 4.3 (2.7,5.4) |
| Iraq | IRQ | 46 (41.1,54.1) | 42.8 (37.9,49.9) | 37.9 (30,52.6) | 0.9 (-0.5,2.1) |
| Ireland | IRL | 9 (8.3,9.6) | 7 (6.3,7.2) | 4 (3.3,4.5) | 3.9 (3.2,4.8) |
| Israel | ISR | 11.5 (10.7,12.2) | 6.9 (6.4,7.4) | 4.3 (3.8,4.9) | 4.7 (4,5.3) |
| Italy | ITA | 9.7 (9,10.3) | 5.6 (5.2,6) | 3.7 (3.1,4.4) | 4.6 (3.7,5.4) |
| Jamaica | JAM | 34.5 (30.8,39.6) | 25.5 (21.6,30.7) | 18.3 (14.4,23.6) | 3 (2.3,3.8) |
| Japan | JPN | 6.4 (6,6.9) | 4.5 (4.2,4.9) | 3.4 (2.9,3.9) | 3 (2.3,3.8) |
| Jordan | JOR | 36.7 (35,40.2) | 28 (25.3,30.4) | 20.7 (16.7,24.4) | 2.7 (2,3.9) |
| Kazakhstan | KAZ | 57 (52.1,61.4) | 42.3 (38.2,50.5) | 28.3 (25.6,42.9) | 3.3 (1.4,3.7) |
| Kiribati | KIR | 87.6 (77.7,102.6) | 65.3 (53.1,84.4) | 47.4 (33.7,70.2) | 2.9 (1.5,4.2) |
| Korea Rep. | KOR | 7.5 (7,8.8) | 5.7 (5.2,6.4) | 4.8 (3.9,5.9) | 2.1 (1.2,3.5) |
| Kuwait | KWT | 16.9 (16.6,18.5) | 12.7 (12.2,13.5) | 10.9 (9.7,12) | 2.1 (1.7,2.9) |
| Kyrgyzstan | KGZ | 70.3 (63.1,78) | 47.4 (41.2,58.6) | 30.6 (24.8,44.4) | 4 (2.4,4.8) |
| Lao PDR | LAO | 147.7 (127.8,159.4) | 81.3 (67.5,95.1) | 41.9 (28.8,59.4) | 6 (4,7.7) |
| Latvia | LVA | 20.6 (19.5,22.5) | 17.3 (16.1,18.4) | 8.3 (7.2,9.7) | 4.3 (3.6,5.2) |
| Lebanon | LBN | 33.1 (27.9,35.1) | 19 (16.5,23.3) | 9.3 (8.2,15.9) | 6 (3.2,6.4) |
| Liberia | LBR | 241.2 (209.4,255.9) | 163.8 (140.3,171.6) | 78.3 (63.3,102.1) | 5.4 (3.8,6.3) |
| Libya | LBY | 44.1 (40.5,49.1) | 27.1 (24.8,28.9) | 16.2 (13.9,17.1) | 4.8 (4.5,5.6) |
| Lithuania | LTU | 17.4 (16.2,18.6) | 11.8 (11.1,12.8) | 5.7 (5,6.8) | 5.3 (4.4,6) |
| Luxembourg | LUX | 8.4 (7.8,9) | 4.9 (4.7,5.4) | 3.2 (2.8,3.6) | 4.6 (3.8,5.4) |
| Macedonia | MKD | 37.6 (34,39.6) | 16.3 (15.5,18) | 9.6 (8.4,11.5) | 6.5 (5.4,7.1) |
| Madagascar | MDG | 161.2 (145,164.7) | 104.1 (97.2,118.6) | 61.6 (57.3,86.1) | 4.6 (2.8,4.7) |
| Malaysia | MYS | 17.2 (15.7,20.1) | 10.6 (10,11.5) | 6.5 (5.5,7.2) | 4.6 (3.9,6) |
| Maldives | MDV | 105.2 (98.2,116.1) | 52.7 (44.5,54.6) | 10.7 (9.3,13) | 10.9 (9.9,11.7) |
| Mali | MLI | 257.3 (239.8,269.9) | 214.4 (194.6,230.6) | 175.6 (152.5,196.8) | 1.8 (1.4,2.3) |
| Malta | MLT | 11.3 (10.6,11.8) | 7.8 (7.5,8.3) | 5.9 (5.2,6.5) | 3.1 (2.5,3.8) |
| Marshall Islands | MHL | 51.9 (46.1,59.2) | 37.5 (32.5,44.2) | 26.2 (21.2,33.4) | 3.3 (2.3,4.2) |
| Mauritania | MRT | 124.7 (115.7,136.5) | 117.9 (102.7,134.3) | 112.1 (86.7,143.3) | 0.5 (-0.6,1.7) |
| Mauritius | MUS | 23.9 (21.4,27.3) | 18.6 (16.4,20.9) | 15.1 (12.1,18.6) | 2.2 (1.1,3.4) |
| Mexico | MEX | 48.8 (44.1,53.5) | 29.1 (27,32.5) | 15.7 (14,17.5) | 5.4 (4.6,6.2) |
| Moldova | MDA | 34.9 (30.5,39.1) | 23.8 (19,29.4) | 16 (10.9,22.2) | 3.7 (2.4,5.2) |
| Monaco | MCO | 7.8 (6.9,8.4) | 5.1 (5,5.5) | 3.9 (3.4,4.2) | 3.3 (2.6,4) |
| Mongolia | MNG | 106.5 (97.1,117.1) | 63.4 (59.1,72.7) | 30.7 (25,45.8) | 5.9 (3.9,7) |
| Montenegro | MNE | 17.6 (15.8,19.6) | 12.6 (11.3,13.3) | 7.2 (6.4,8.9) | 4.3 (3.1,5.1) |
| Morocco | MAR | 81.3 (73.7,84.6) | 52.7 (46.9,56.7) | 32.8 (26.8,39.2) | 4.3 (3.3,5.2) |
| Myanmar | MMR | 107.4 (92.3,114.9) | 83.5 (66.7,96.8) | 62.4 (44.9,84) | 2.6 (1.2,3.9) |
| Nepal | NPL | 134.6 (121.3,136.4) | 82.9 (77,89.2) | 48 (44.5,57.1) | 4.9 (3.9,5.1) |
| Netherlands | NLD | 8.3 (7.9,8.7) | 6.2 (5.9,6.4) | 4 (3.7,4.5) | 3.5 (2.9,3.9) |
| New Zealand | NZL | 11.1 (10.3,11.8) | 7.4 (6.9,8) | 5.9 (5,6.8) | 3 (2.3,3.8) |
| Nicaragua | NIC | 66.1 (60.5,69.8) | 42.3 (38.4,46.9) | 25.6 (21.8,32.1) | 4.5 (3.3,5.3) |
| Niger | NER | 313.7 (289.7,328.6) | 215.6 (191.5,230.8) | 124.5 (98.7,155.9) | 4.4 (3.2,5.5) |
| Nigeria | NGA | 213.6 (196.3,226.7) | 187.9 (169.7,196.4) | 124.1 (110.6,147) | 2.6 (1.7,3.1) |
| Niue | NIU | 14.2 (10.8,16.4) | 29.4 (21.4,32.4) | 21.1 (18.1,47.4) | -1.9 (-6.3,-1.1) |
| Norway | NOR | 8.4 (7.6,8.8) | 4.9 (4.5,5.2) | 3.1 (2.7,3.6) | 4.7 (3.9,5.4) |
| Oman | OMN | 47.5 (42.6,51.3) | 21.7 (18.8,25.3) | 8.7 (6.6,12.4) | 8.1 (6.3,9.3) |
| OPT | PSE | 43.1 (38.8,47) | 30.1 (25.6,33.3) | 22 (15.1,26.7) | 3.2 (2.2,5) |
| Pakistan | PAK | 122.2 (111.9,129.1) | 95.3 (85.5,103.3) | 72 (57.7,86) | 2.5 (1.5,3.5) |
| Palau | PLW | 32.3 (25.9,40.8) | 24.8 (20.6,30) | 18.6 (11.5,29.9) | 2.6 (-0.1,5.5) |
| Panama | PAN | 33.3 (30.3,37.6) | 25.7 (22.3,30.6) | 19.5 (15,25.8) | 2.5 (1.4,3.8) |
| Papua New Guinea | PNG | 88 (81.6,96.5) | 71.7 (62.2,79.5) | 57.8 (45.2,65.1) | 2 (1.7,3) |
| Paraguay | PRY | 52.6 (45.9,57.1) | 35.3 (30.2,41.1) | 22.4 (17.8,30.4) | 4.1 (2.5,5) |
| Peru | PER | 75.1 (69.8,78.2) | 38.9 (36.1,40) | 18.1 (16,19.1) | 6.8 (6.3,7.4) |
| Philippines | PHL | 57 (52.7,59) | 38.8 (35.5,42.4) | 25.4 (22.2,30.3) | 3.8 (3,4.3) |
| Poland | POL | 17.3 (16.1,18.5) | 9.6 (9.1,10.5) | 5.8 (5,6.7) | 5.2 (4.4,6) |
| Portugal | PRT | 14.6 (13.5,15.6) | 7.2 (6.6,7.6) | 3.4 (2.9,3.9) | 6.9 (6.2,7.8) |
| Qatar | QAT | 20.2 (18.4,21.9) | 12.6 (12.2,13.1) | 7.7 (7.1,8.2) | 4.6 (4,5.2) |
| Romania | ROU | 37.4 (35.8,38) | 26.8 (25.8,27.5) | 12.5 (12,13.6) | 5.2 (4.7,5.4) |
| Russian Federation | RUS | 27.3 (25.7,29.2) | 21.3 (19.2,21.8) | 11.9 (11.3,13.8) | 4 (3.2,4.3) |
| Saint Kitts & Nevis | KNA | 28.3 (25.2,32.5) | 16.3 (14,18.7) | 7.4 (5.8,11.6) | 6.4 (4,7.8) |
| Saint Lucia | LCA | 22.5 (20.9,24.8) | 18.1 (16.6,20) | 15.6 (11.9,19) | 1.7 (0.7,3.2) |
| Samoa | WSM | 29.5 (27.2,33.7) | 22.7 (18.9,26.8) | 18.7 (11.2,25.8) | 2.2 (0.6,4.9) |
| San Marino | SMR | 11.9 (10.3,13.3) | 5.3 (4.6,6) | 1.8 (1.4,2.6) | 9 (6.9,10.2) |
| Sao Tome & Principe | STP | 96 (86.3,110.7) | 92.5 (80.4,116.7) | 88.8 (70.8,128.7) | 0.4 (-1,1.4) |
| Saudi Arabia | SAU | 42.7 (36.2,50.1) | 20.6 (17.4,24.3) | 9.2 (7.4,11.5) | 7.3 (6.4,8.2) |
| Senegal | SEN | 135.9 (129.6,145.6) | 130.4 (117.6,135.9) | 64.8 (59.4,91.1) | 3.5 (1.9,4) |
| Serbia | SRB | 28.6 (24.3,33.6) | 12.7 (10.9,15.1) | 7.1 (5,9.8) | 6.6 (4.9,8.4) |
| Seychelles | SYC | 16.6 (15.7,19.1) | 13.8 (12.8,15.5) | 13.8 (11.5,16.2) | 0.9 (0.2,2.1) |
| Sierra Leone | SLE | 266.7 (243.6,309.1) | 240.6 (216.2,281.4) | 185.3 (129.9,310) | 1.7 (-0.7,3.7) |
| Singapore | SGP | 7.5 (7.3,7.8) | 3.9 (3.8,4) | 2.6 (2.3,2.7) | 5 (4.8,5.7) |
| Slovakia | SVK | 17.6 (17.3,18.1) | 11.6 (11.4,11.9) | 7.7 (7.4,8.1) | 3.9 (3.7,4.2) |
| Slovenia | SVN | 10.4 (9.8,10.9) | 5.4 (5.1,5.8) | 2.8 (2.6,3.2) | 6.2 (5.5,6.7) |
| Solomon Islands | SLB | 41.8 (37.8,48.6) | 30.5 (24,33.6) | 21.6 (14.2,23.6) | 3.1 (2.9,4.9) |
| South Sudan | SSD | 217.3 (188.5,255.6) | 164.5 (142.5,200.7) | 120.5 (92.5,171.2) | 2.8 (1,4.3) |
| Spain | ESP | 10.9 (10.5,11.1) | 6.6 (6.5,6.8) | 4.2 (4.1,4.5) | 4.5 (4.1,4.7) |
| Sri Lanka | LKA | 28.9 (27.3,30.8) | 19.1 (17.1,20.5) | 12.2 (10,13.4) | 4.1 (3.8,5) |
| Saint Vincent & the Grenadines | VCT | 26.5 (24.8,33) | 21.9 (19.5,25.5) | 20.9 (15.5,27) | 1.1 (0,3.2) |
| Sudan | SDN | 122.8 (114.4,135.8) | 103.7 (91.7,121.1) | 86 (66.1,117.3) | 1.7 (0.3,3.1) |
| Suriname | SUR | 51.9 (46.3,61.2) | 39.9 (34.3,50.8) | 29.5 (21.5,47.4) | 2.7 (0.4,4.5) |
| Sweden | SWE | 6.7 (6.3,6.8) | 4.1 (3.9,4.3) | 2.8 (2.6,3.1) | 4.2 (3.5,4.5) |
| Switzerland | CHE | 8.1 (7.2,8.8) | 5.7 (5.2,6.3) | 4.4 (3.6,5.4) | 2.9 (1.8,3.9) |
| Syria | SYR | 36.1 (32.8,39.5) | 22.8 (20.6,25.7) | 15.3 (11.7,18.8) | 4.1 (3,5.4) |
| Tajikistan | TJK | 114.3 (99.4,127.7) | 94.7 (80.6,129.7) | 63.3 (39.6,163.8) | 2.8 (-1.8,5) |
| Thailand | THA | 35 (31.6,39.2) | 18.5 (16.6,21.5) | 12.3 (8.4,17.1) | 5 (3.3,7) |
| Timor-Leste | TLS | 180 (164.2,199.1) | 109.4 (99.5,120.9) | 54.1 (46.3,72.3) | 5.7 (4.2,6.7) |
| Togo | TGO | 147 (137.8,161) | 127.8 (115.8,147.1) | 110.1 (92.4,139.3) | 1.4 (0.4,2.1) |
| Tonga | TON | 24.5 (21.9,29.1) | 19.6 (15.7,24.6) | 15.4 (10.5,21.5) | 2.2 (1.1,3.8) |
| Trinidad & Tobago | TTO | 36.8 (33,44.3) | 32.1 (27,41.2) | 27.7 (21.1,38.9) | 1.4 (0.4,2.3) |
| Tunisia | TUN | 51.1 (42.1,57.4) | 29.6 (24.7,33.5) | 16.2 (12.7,19.5) | 5.5 (4.5,6.4) |
| Turkey | TUR | 72 (66.2,75.5) | 35.3 (30.5,39.1) | 15.2 (11.9,20.4) | 7.4 (5.9,8.6) |
| Turkmenistan | TKM | 94.3 (86.4,103.3) | 71.4 (61.9,86.3) | 52.5 (40.9,74) | 2.8 (1.3,3.8) |
| Tuvalu | TUV | 57.6 (50.5,63.8) | 43.3 (38.4,48.1) | 30.1 (24,39.8) | 3.1 (1.4,4.4) |
| Ukraine | UKR | 19.4 (18.2,21.7) | 18.6 (16.6,19.9) | 10.1 (8.5,12.3) | 3.1 (2.2,4.2) |
| United Arab Emirates | ARE | 22.2 (21.3,25.3) | 12.3 (11.6,13.4) | 6.6 (5.7,7) | 5.8 (5.5,6.8) |
| United Kingdom | GBR | 9.2 (8.9,9.6) | 6.6 (6.3,6.8) | 5.1 (4.8,5.6) | 2.8 (2.3,3.1) |
| United States of America | USA | 11.3 (10.9,11.5) | 8.5 (8.3,8.8) | 7.5 (6.9,8) | 2 (1.6,2.3) |
| Uruguay | URY | 23.1 (22.4,24.6) | 17.1 (16.3,17.9) | 10.3 (9.3,11.6) | 3.8 (3.3,4.4) |
| Uzbekistan | UZB | 75.3 (69.8,85.2) | 61 (54.1,72.6) | 48.6 (37.6,66.2) | 2.1 (0.7,3.5) |
| Vanuatu | VUT | 38.5 (34.4,44.6) | 23 (19.7,28.8) | 13.2 (10.3,18.4) | 5.1 (3.9,6) |
| Venezuela | VEN | 30.9 (28.5,34) | 22.3 (21.1,23.8) | 15 (13.8,16.2) | 3.4 (2.9,4) |
| Viet Nam | VNM | 49.9 (44.4,55) | 33.9 (31.1,36.6) | 21.7 (19.6,24.1) | 4 (3.2,4.7) |
| Yemen | YEM | 126 (116,135.6) | 99.1 (84.7,109.7) | 76.5 (57.7,92.1) | 2.4 (1.5,3.6) |
