# Supplementary material for: Progress toward Global Reduction in Under-Five Mortality: A Bootstrap Analysis of Uncertainty in Millennium Development Goal 4 Estimates
Source: PLoS Med. 2012 Dec 11;9(12):e1001355. doi: 10.1371/journal.pmed.1001355 (PMC3519895; doi:10.1371/journal.pmed.1001355)
Supplement: Text S1 — Supplementary information on the construction of the uncertainty intervals. (PDF) [file pmed.1001355.s003.pdf]

Progress Toward Global Reduction in Under-5 Mortality: A Bootstrap Analysis of Uncertainty in Millennium Development Goal 4 Estimates

Text S1: Supplementary information

Leontine Alkema and Jin Rou New *

Contents

1 Additional details on the parametric bootstrap 2

1.1 U5MR data model for non-VR data 2

1.2 U5MR data model for VR data 2

1.3 Bootstrap procedure 3

2 Construction of UN-IGME 2012 uncertainty intervals for additional countries, country-years with adjustment and other mortality indicators 3

*Department of Statistics and Applied Probability, National University of Singapore. Contact: alkema@nus.edu.sg.

1 Additional details on the parametric bootstrap

1.1 U5MR data model for non-VR data

The U5MR data model is given as follows:

$$\log(y_i) = \log(u_{c[i],t[i]}) + \delta_i, \tag{1}$$

where y_i is observed U5MR for observation $i = 1, \ldots, n$, $c[i]$ the corresponding country, $t[i]$ the observation date, and $u_{c,t}$ is the U5MR in country c at time t.
For non-VR data, we assumed

$$\begin{aligned} \delta_i &\sim N(\beta_{0,s[i]} + \beta_{1,s[i]} \cdot \pi_i, \sigma_i^2), \\ \beta_{0,s} &\sim N(\mu_{0,j[s]}, s^2_{0,j[s]}), \\ \beta_{1,s} &\sim N(\mu_{1,j[s]}, s^2_{1,j[s]}), \end{aligned}$$

where

- $s[i]$ refers to the data series, and $\pi[i]$ the retrospective period (centered) for observation i,
- $j = 1, \ldots, 8$ refer to the non-VR source types (DHS direct with/without reported sampling errors, others direct, DHS indirect, MICS indirect, census indirect, others indirect and others respectively).
- Parameter σ_i^2 represents the error variance, a combination of sampling and non-sampling variance,

  $$\sigma_i^2 = \omega^2_{j[i]} + \tau_i^2 / y_i^2,$$

  with $\omega^2_{j[i]}$ the non-sampling variance for source type j, and sampling variance τ_i^2/y_i^2 for log(U5MR) (based on sampling variance τ_i^2 for U5MR). Sampling variance is available for DHS direct series only.
- $\mu_{k,j[s]}$ and $s^2_{k,j[s]}$ are the mean and variance of the coefficient $\beta_{k,s}$ for series s of source type j.

All data model parameters were estimated by using the interpolated UN-IGME estimates as our estimate for $u_{c,t}$, denoted by $\hat{u}_{c,t}$. The estimates of the data model parameters for all source types were obtained using open source software package `R 2.14` (R Development Core Team 2011). Additionally, for the parameter estimates for the DHS direct observations the software package `WinBUGS` (Lunn et al. 2000) and `R`-package **R2WinBUGS** (Sturtz et al. 2005) were used.

1.2 U5MR data model for VR data

For VR data, δ_i in Eq.1 was modeled as follows:

$$\delta_{c,t} \sim \begin{cases} N(0, \sigma_c^2), & \text{for } c \in A, \\ N(\rho_c \cdot d_{c,t-1}, \epsilon_c^2), & \text{for } c \in B, \end{cases}$$

such that for the countries in subset A, the $\delta_{c,t}$'s are random draws with variance σ_c^2, while for the remaining countries in subset B, the $\delta_{c,t}$ are realizations from an AR(1) process with autoregressive parameter ρ_c (with $|\rho_c| < 1$) and variance ϵ_c^2. This AR(1) model for the $\delta_{c,t}$'s is appropriate for countries where the loess smoother did not adequately capture temporal fluctuations in the VR data. Denoting $\boldsymbol{\delta}_c = (\delta_{c,t_1}, \delta_{c,t_2}, \ldots, \delta_{c,t_n})$ (where t_n refers to the n-th observation year for country c), an equivalent notation for VR data in the countries in group B is given by:

$$\begin{aligned} \boldsymbol{\delta}_c &\sim \boldsymbol{N}(\mathbf{0}, \boldsymbol{\Delta}_c), \\ \Delta_{c,n,m} &= \frac{{\epsilon_c}^2}{1 - {\rho_c}^2} \cdot {\rho_c}^{|t_n - t_m|}. \end{aligned}$$

To assign a country to group A or B, we estimated the VR parameters ($\hat{\theta}_c$, $\hat{\sigma}_c$, $\hat{\epsilon}_c$, $\hat{\rho}_c$ (using the interpolated UN-IGME estimates as our estimate for $u_{c,t}$) and assumed that the country was in group A, unless the stationary variance of the AR(1) process for that country exceeded the variance of the simple random draws, $\hat{\epsilon}_c^2/(1-\hat{\rho}_c^2) > \hat{\sigma_c}^2$. For countries with less than ten VR observations, parameter estimates were based on the $\delta_{c,t}$'s for all countries combined.

1.3 Bootstrap procedure

For non-VR data, data series were sampled in bootstrap h as follows:

$$\begin{aligned}\boldsymbol{\beta}_s^{(h)} &\sim N(\hat{\boldsymbol{\mu}}_{j[s]}, \widehat{\boldsymbol{\Sigma}}_{j[s]}),\\ \delta_i^{(h)} &\sim N(\beta_{0,s[i]}^{(h)} + \beta_{1,s[i]}^{(h)} \cdot \pi_i, \hat{\omega}_{j[i]}^2 + \tau_i^2/y_i^2),\\ y_i^{(h)} &= \hat{u}_{c[i],t[i]} \cdot \exp\left(\delta_i^{(h)}\right),\end{aligned}$$

where $\hat{\boldsymbol{\mu}}_j = (\hat{\mu}_{0,j}, \hat{\mu}_{1,j})$, $\widehat{\boldsymbol{\Sigma}}_j$ and $\hat{\omega}_j^2$ follow from the multilevel model estimates ($\widehat{\boldsymbol{\Sigma}}_j$ is the sum of the estimated covariance matrix of the $\boldsymbol{\beta_s}$'s for source type j and the estimated covariance matrix of $\boldsymbol{\mu_j}$). $\hat{u}_{c,t}$ is the UN-IGME estimate for the U5MR in country c in year t.
For VR-data, we sampled

$$\begin{aligned}\boldsymbol{\delta}_c^{(h)} &\sim \boldsymbol{N}(\boldsymbol{0}, \widehat{\boldsymbol{\Delta}}_c),\\ \widehat{\boldsymbol{\Delta}}_c &= \begin{cases} \hat{\sigma_c}^2 \boldsymbol{I}, & \text{for } c \in A,\\ \frac{\hat{\epsilon_c}^2}{1-\hat{\rho_c}^2} \cdot \widehat{\boldsymbol{X}}_c, & \text{for } c \in B, \text{ with } \widehat{X}_{c,n,m} = \hat{\rho_c}^{|t_n - t_m|}, \end{cases}\\ y_i^{(h)} &= \hat{u}_{c[i],t[i]} \cdot \exp\left(\delta_i^{(h)}\right).\end{aligned}$$

After sampling the non-VR data series and VR data, the loess smoother was fitted to bootstrapped series $y_i^{(h)}$, resulting in $u_{c,t}^{(h)}$, the h-th sample of the U5MR in country c, year t. The lower and upper bounds were based on the 5th and 95th percentiles of all samples in a given year and denoted by $u_{c,t}^{(L)}$ and $u_{c,t}^{(U)}$. For a limited number of country-years where the loess estimate ended up below (above) the bootstrapped 90% UI, the loess estimate was used as the lower (upper) bound.

2 Construction of UN-IGME 2012 uncertainty intervals for additional countries, country-years with adjustment and other mortality indicators

U5MR for HIV countries For high HIV prevalence countries, an alternative estimation procedure is used by the UN-IGME to account for the effect of the epidemic on reporting biases the levels and trend of U5MR; U5MR is first estimated for the "non-AIDS" scenario and AIDS deaths are added in at a later step (Walker et al. 2012). The bootstrap method was used to construct UIs for these countries in the first step of the estimation method, that is, to assess the uncertainty in the "non-AIDS" U5MR estimates. To produce UIs for the total U5MR, $u_{c,t}^{(h)}$ for bootstrap $h = 1, \dots, H$, the assumption was made that the relative uncertainty in the non-AIDS U5MR is equal to the relative uncertainty in the total U5MR:

$$u_{c,t}^{(h)} = u_{c,t}^{(h),noAIDS} \frac{\hat{u}_{c,t}}{\hat{u}_{c,t}^{noAIDS}}.$$

where $u_{c,t}^{(h),noAIDS}$ refers to the h-th sample of the non-AIDS U5MR in country c, year t, and $\hat{u}_{c,t}^{noAIDS}$ refers to the UN-IGME "no-AIDS" point estimate.

U5MR for country-years with post-adjustments For a limited number of country-years, adjustments are carried out after fitting the loess curve, for example, to incorporate the increased number of deaths related to natural disasters such as the 2010 earth quake in Haiti. For these country-years, the bootstrapped U5MR estimates are given by:

$$u_{c,t}^{(h)} = u_{c,t}^{(h),noAdj} \frac{\hat{u}_{c,t}}{\hat{u}_{c,t}^{noAdj}},$$

where $u_{c,t}^{(h),noAdj}$ refers to the h-th sample of the unadjusted U5MR in country c, year t and $\hat{u}_{c,t}^{noAdj}$ to the unadjusted UN-IGME point estimate. Note that the uncertainty in the adjustment is not assessed, instead we assume that the relative uncertainty in the adjusted U5MR is equal to the relative uncertainty in the non-adjusted U5MR.

Infant mortality rate Uncertainty bounds for the infant mortality rate, ${}_1q_0$, were constructed by assuming that the relative uncertainty in ${}_1q_0$ is equal to the relative uncertainty in U5MR:

$$q_{c,t}^{(h)} = \hat{q}_{c,t}\frac{u_{c,t}^{(h)}}{\hat{u}_{c,t}}.$$

where $q_{c,t}^{(h)}$ refers to the h-th bootstrapped estimates for ${}_1q_0$ for country c, year t, and $\hat{q}_{c,t}$ to the UN-IGME point estimate.

Number of deaths The standard calculation of the number of deaths is based on the infant mortality rate as well as ${}_4q_1$, the probability of dying between ages 1 and 4. To derive UIs for the number of deaths, the h-th sample of U5MR and IMR was used to first derive the h-th sample for the ${}_4q_1$, and subsequently, the joint sample of ${}_4q_1$ and ${}_1q_0$ was used to calculate the h-th sample for the number of deaths. Uncertainty in the number of births was not accounted for.

UIs for aggregate (global and regional) estimates The standard calculation for aggregates estimates is based on the estimated number of deaths. To derive the h-th sample for an aggregate estimate, this calculation method was applied to the h-th sample for the number of deaths.

References

Lunn, D., A. Thomas, N. Best, and D. Spiegelhalter (2000). WinBUGS - a Bayesian modelling framework: concepts, structure, and extensibility. *Statistics and Computing 10*, 325–227.

R Development Core Team (2011). *R: A Language and Environment for Statistical Computing*. Vienna, Austria: R Foundation for Statistical Computing. ISBN 3-900051-07-0.

Sturtz, S., U. Ligges, and A. Gelman (2005). R2WinBUGS: A Package for Running WinBUGS from R. *Journal of Statistical Software 12*(3), 1–16.

Walker, N., K. Hill, and F. Zhao (2012). Child Mortality Estimation: Methods Used to Adjust for Bias due to AIDS in Estimating Trends in Under-Five Mortality. *PLoS Medicine 9*(8), e1001298.
